# Carbon Nanomaterial Fluorescent Probes and Their Biological Applications

**DOI:** 10.1021/acs.chemrev.3c00581

**Published:** 2024-03-13

**Authors:** Andrew T. Krasley, Eugene Li, Jesus M. Galeana, Chandima Bulumulla, Abraham G. Beyene, Gozde S. Demirer

**Affiliations:** † Janelia Research Campus, Howard Hughes Medical Institute, 19700 Helix Drive, Ashburn, Virginia 20147, United States; ‡ Division of Chemistry and Chemical Engineering, California Institute of Technology, 1200 E. California Boulevard, Pasadena, California 91125, United States

## Abstract

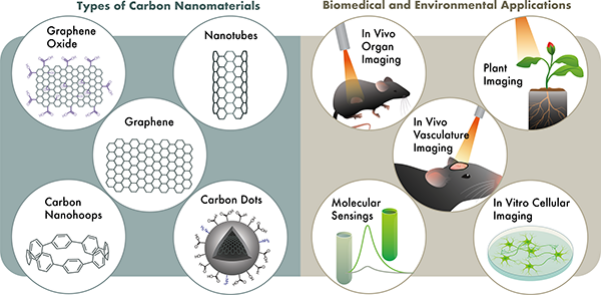

Fluorescent carbon nanomaterials have broadly useful chemical and photophysical attributes that are conducive to applications in biology. In this review, we focus on materials whose photophysics allow for the use of these materials in biomedical and environmental applications, with emphasis on imaging, biosensing, and cargo delivery. The review focuses primarily on graphitic carbon nanomaterials including graphene and its derivatives, carbon nanotubes, as well as carbon dots and carbon nanohoops. Recent advances in and future prospects of these fields are discussed at depth, and where appropriate, references to reviews pertaining to older literature are provided.

## 1 Introduction

Fluorescent carbon nanomaterials (CNMs) probes have garnered significant attention in the fields of biomedicine and environmental science due to a desirable array of optical, electrical, chemical, and material properties. These CNMs encompass several classes of nanoparticles whose primary constituent is elemental carbon. The first in the family of these materials is graphene, with a characteristic planar structure made from sp^2^ hybridized carbon atoms arranged in an extended honeycomb network, and its derivatives such as graphene oxide (GO) and graphene nanoribbons (GNR).^1^ While graphene is an allotrope of elemental carbon and has dimensions on the scale of ∼10 μm, GO and related family of graphene derivatives constitute a mix of sp^2^ and sp^3^ hybridized carbon atoms, typically contain epoxide, carbonyl, or carboxylic acid functional groups, and have dimensions that are on the order of ∼10 nm. Graphene is a zero-bandgap nanomaterial with metallic character and, despite a wealth of fascinating material properties, is nonfluorescent and hence not a focus of this review. However, it forms the basis for understanding GO and GNR family of nanomaterials, which can be synthesized from graphene, and can be fluorescent with demonstrated use for biological and environmental applications. We will therefore introduce graphene and discuss its properties as it enables us to explain the synthesis and material properties of GO and related derivatives. Later sections of this review, which focus on applications and use cases of CNMs, will primarily focus on GO and other fluorescent CNMs, and not graphene.

Carbon nanotubes (CNTs) constitute another important class of fluorescent CNMs included in this review. Carbon nanotubes are cylindrical nanocrystals of sp^2^ hybridized carbon atoms that can be conceptualized as rolled sheets of graphene. While the diameter of CNTs is typically on the order of single nanometers, their length could extend for up to ∼1 μm. Within CNTs, one can distinguish between single-walled CNTs (SWCNTs), and double and multiwalled CNTs (DWCNTs and MWCNTs, respectively). SWCNTs are single rolled sheets of graphene, whereas DWCNTs and MWCNTs can have two or multiple coaxial rolled sheets of graphene that are nested within each other. Quantum confinement effects give rise to a set of unique photophysical properties in semiconducting SWCNTs, including a nonphotobleaching fluorescence in the near-infrared and shortwave infrared (NIR/SWIR) regions of the electromagnetic spectrum (850–1400 nm). Despite the reported low quantum yield (QY) of SWCNTs (typically ∼1%), their stable photoemission spectra, sharp optical transitions (full width at half-maximum ∼180–200 cm^–1^, or ∼20 nm), and large absorption cross section (10^–15^–10^–17^ cm^2^/C atom) can be advantageous for biological imaging applications.^2,3^ Moreover, the fact that SWCNT photoemission emanates from surface bound (and hence environmentally sensitive) excitons make SWCNTs excellent scaffolds for biosensing with single molecule sensitivity.^4,5^ Other members of the CNT family, including DWCNTs and MWCNTs, are nonfluorescent because the coaxial geometry of nested nanotubes facilitates efficient nonradiative relaxation from otherwise fluorescent single tubes.^6,7^ Therefore, DWCNTs and MWCNTs are not a focus of this review. Carbon nanocones (CNCs, also known as carbon nanohorns) encompass another class of sp^2^ hybridized rolled graphene sheets with conical, as opposed to cylindrical, geometry. Although they are easier to synthesize than CNTs, and have been used as nanohybrids in conjunction with other fluorescent nanomaterials and dyes, CNCs do not have intrinsic fluorescence of their own and are therefore not a focus of the later sections of this review.^8^

Carbon dots (CDs) refer to a major class of CNMs that also includes carbon quantum dots (CQDs) and carbonized polymer dots (CPDs) and are an important focus of this review.^9^ CDs are quasi zero-dimensional, spherical CNMs, with diameters that are in the range of ∼1–10 nm. They can be synthesized from a wide range of precursor materials, are intrinsically fluorescent, and exhibit diverse photophysical and material properties that are functions of the carbon source and the synthetic strategy used to produce them. Indeed, the latest synthetic strategies can now furnish bright CDs with quantum yields up to 80%, and highly tunable and stable photoemission ranging from blue to NIR, from a wide range of abundant low-cost source materials. Relative to other fluorescent CNMs, CDs permit a better degree of control and ease over their synthesis and purification, which has enabled the generation of CDs exhibiting a wide range of photophysical and chemical properties. This diversity has also led to the use of CDs in a wide range of applications, including bioimaging, which we extensively explore in this review.^9^

Carbon nanohoops (CNHs) constitute the smallest and newest class of fluorescent CNMs discussed in this review. CNHs are composed of aromatic rings that are fused to generate a macrocyclic structure that resembles the smallest slice of a SWCNT. CNHs are unique among fluorescent CNMs in that they are synthesized bottom up from small molecule precursors using strategies that benefit from advances in modern synthetic organic chemistry, including precise control over molecular structure, excellent characterization, and purification to produce monodisperse products with well-behaved photophysical and chemical properties. As the newest member of fluorescent CNMs, applications of CNHs for bioimaging are still in their infancy, but early results have been highly encouraging, and weexplore these advances in the review.

Small molecule organic fluorophores and fluorescent proteins (FPs) still constitute the primary reagents of choice in scientific research where imaging or sensing is employed. There are several reasons for this. These reagents are better characterized, are monodisperse (pure), and therefore generally well behaved compared to fluorescent CNMs. They are also optically compatible with most commercially available microscopes. FPs are typically expressed through common genetic strategies widely available to experimental biologists, which facilitates their ease of use. Similarly, some organic fluorescent dye reagents are straightforward in their application. However, as we highlight in this review, there are some unique advantages that fluorescent CNMs provide that make them a rational or only choice for certain biological applications.

First, the optical properties of fluorescent CNMs can be quite advantageous for applications in biology. A commonly encountered theme in the emissive properties of all CNMs is a remarkable photostability, with some fluorescent CNMs exhibiting nonphotobleaching fluorescence. Additionally, emission is highly tunable, broadly encompassing the visible, NIR, and SWIR regions of the spectrum. A dearth of fluorophores that emit in the NIR/SWIR means that CNMs could be compelling reagents of choice for imaging and biosensing in that region of the spectrum. Second, thanks to a unique combination of their small size, and surface and mechanical properties, some CNMs are able to reach and enter cell and tissue types that otherwise are inaccessible to traditional probes, enabling applications in neural tissues and plant organelles for instance. Third, preparation of most fluorescent CNMs does not require sophisticated synthesis and purification skills, and these materials can be produced at scale and low cost compared to other laboratory reagents. Lastly and importantly, fluorescent CNMs facilitate multiplexed use cases, in which the nanomaterials can be functionalized with contrast agents that allow orthogonal imaging modalities, or can be loaded with therapeutics, drugs, or biomolecules, such as genes and proteins, for delivery into cells. Compared to fluorescent nanomaterials synthesized from heavy metals, CNMs are biocompatible, and their abundant functional handles can be ligated to fine-tune their biointerfacial properties. Although not in the scope of this review, CNMs have also found applications in a diverse range of the scientific enterprise, including electrochemical sensing, optoelectronics, catalysis, and energy storage.

In this paper, we provide a review of the synthesis, functionalization, characterization, and material properties of the CNMs that we introduced in the preceding paragraphs. Subsequently, we explore the applications of fluorescent CNMs in biological imaging, molecular sensing, and cargo delivery both in biomedical and environmental science and engineering. We conclude the review by discussing the important topics of CNM cytotoxicity, environmental accumulation, and fate, and their scale-up, economical, and regulatory considerations, all of which are critical factors for the successful translation of CNMs from the lab to clinical and field applications. This review mostly covers advancements made in the last five years, with relevant comprehensive reviews suggested for earlier studies for interested readers. However, older literature are discussed in cases where new literature is unavailable, or the earlier literature still represent the most significant advancements for the topic at hand.

## 2 Carbon Nanomaterial Synthesis and Characterization

In this section, we discuss synthesis and characterization methods for CNMs briefly introduced in the previous section. We discuss material properties in Section 3, chemical modifications in Section 4, and biological and environmental applications in Sections 5 and 6.

### 2.1 Carbon Nanotubes (CNTs)

**Figure 1:**
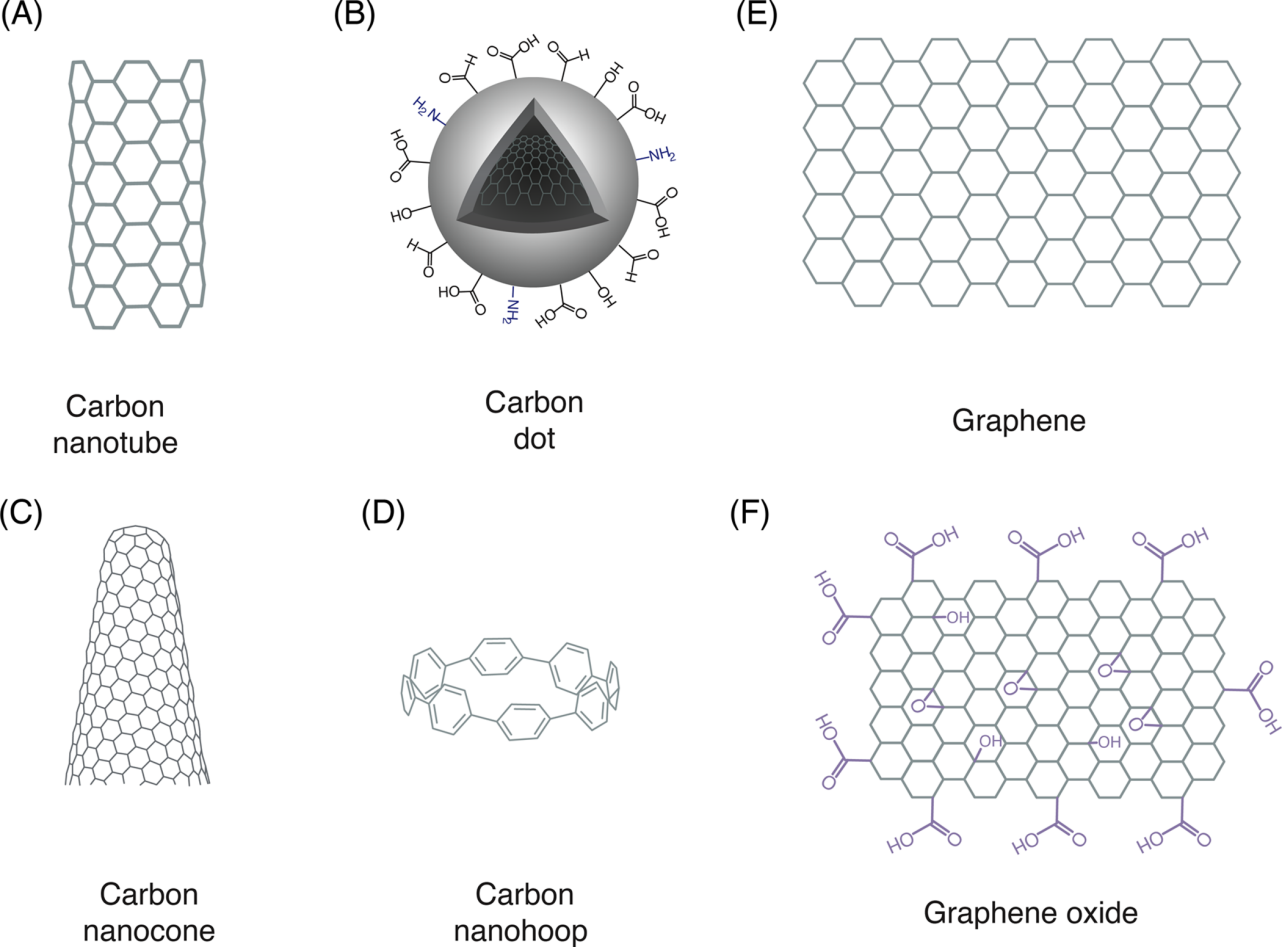
Carbon nanomaterial (CNM) types and their structures. (A) Pristine carbon nanotubes are cylindrical nanocrystals of sp^2^ hybridized carbon atoms. (B) Carbon dots are quasi-spherical nanoparticles with a mix of sp^2^ and sp^3^ carbon atoms and contain a variety of functional handles. (C) Carbon nanocones represent sp^2^ carbon atoms rolled into a conical geometry. (D) Carbon nanohoops can be conceptualized as a single slice of a carbon nanotube. (E, F) Pristine graphene is a 2-dimensional material made of sp^2^ carbon atoms in a honeycomb-like arrangement, whereas graphene oxide contains a mix of sp^2^ and sp^3^ carbon atoms and features various functional moieties.

Carbon nanotubes (CNTs) are nanocrystalline materials that are composed of a hexagonal sp^2^ hybridized network of carbon atoms that are rolled into a cylindrical form (Figure 1A). They can contain single, double, or multiple coaxial layers resulting in either single-walled (SWCNTs), double-walled (DWCNTs), or multiwalled carbon nanotubes (MWCNTs). CNTs can be synthesized through various methods, with the three primary modes of synthesis being chemical vapor deposition (CVD),^10−12^ arc discharge,^13^ and laser ablation.^14^ In contrast to DWCNTs and MWCNTs, SWCNTs possess intrinsic and unique photophysical properties, and have been extensively employed for biosensing and imaging applications and will therefore be one of the primary CNMs discussed in this review.

Since their first discovery in the late 20th century, SWCNTs have drawn interest from a wide range of scientific fields due to their mechanical, chemical, electrical, and optical properties.^15−17^ While SWCNTs typically have a diameter of 1–3 nm, DWCNTS and MWCNTs can have a broader diameter distribution ranging from 2 to 100 nm.^18^ Depending on the structure of the graphitic lattice, SWCNTs can be categorized into three groups: armchair, zigzag, or chiral.^19^ SWCNT electronic band gap structure is critical for setting their electronic and optical properties. For instance, certain SWCNTs can serve as field effect transistors,^20^ and tracking change in electrical or optical properties across the nanotube in the presence of adsorbed molecules can provide a means for molecular sensing. Also a consequence of their electronic bandgap structure, certain SWCNT chiralities exhibit photoluminescence by absorbing light in the NIR-I and emitting in the NIR-II region. This makes them excellent reagents for biological imaging^21,22^ and scaffolds for biosensing applications.^23,24^ SWCNTs have also been employed as photoinduced drug delivery vessels,^25,26^ gene and protein delivery vehicles,^27,28^ nanopores,^29,30^ adjuvant vaccines,^31^ and are used in tissue engineering applications.^32−34^

### 2.2 Carbon Dots (CDs)

Carbon dots (CD), quasi-spherical in nature, are typically smaller than 10 nm in diameter and encompass a collection of nanoparticles, such as graphene quantum dots (GQDs), carbon quantum dots (CQDs), and carbonized polymer dots (Figure 1B).^35^ CDs have been extensively used in various fields due to their tunable photoluminescent (PL) properties,^35−38^ chemical diversity,^39,40^ and biocompatibility.^41,42^

Synthesis routes consist of top-down and bottom-up approaches. For bottom-up synthesis, polymers,^43,44^ glucose,^45,46^ glycerol,^47,48^ biowaste,^49,50^ amino acids,^51,52^ among others have been used to create surface-functionalized CDs via hydrothermal methods or microwave pyrolysis. Top-down methods for CD synthesis require cleavage of larger carbon allotropes like graphite,^53−57^ GO,^58^ carbon fibers,^59^ and other carbon materials.^60,61^ Recently, there has also been a growing literature on the “green synthesis” of CDs from biological organisms.^62,63^

The popularity of CDs for bioimaging have been attributed to their unique photoluminescence properties and high quantum yields.^64,65^ Their π-conjugated system absorbs UV light and provides emission of visible light facilitated by both n → π* and π → π* transitions of C=N and C=O, and C=C bonds, respectively.^66,67^ Many studies suggest that N doping (adding nitrogen) of CDs leads to higher quantum yields.^67^ These inherent photoluminescence properties and biocompatibility of CDs make them ideal fluorescent probes, where they have been utilized to image cells,^68,69^ biomolecules,^70^ and various other biological systems.^71−73^

### 2.3 Carbon Nanocones (CNCs) and Carbon Nanohoops (CNHs)

Beyond the aforementioned CNMs, carbon nanocones and nanohoops have gained increasing attention for their distinctive physicochemical properties (Figure 1C,D). Although their applications in biological research are less developed, these materials hold a promising potential for biosensing, bioimaging, and therapeutics.

Carbon nanocones (CNCs), also known as nanohorns, are comprised of carbon atoms arranged within a highly conjugated C–C π-system akin to CNTs and graphene sheets. CNCs have a diameter of 2–5 nm and length of 40–50 nm.^74^ Diverging from CNTs and graphene, CNCs have one end enclosed and the other end open, embodying the shape of an ice cream cone (Figure 1C). They can be synthesized by various processes depending on the desired size, including cascade annulation,^75^ and laser and solar radiation ablation.^76,77^ The potential applications of CNCs include biosensing, bioimaging, therapeutics, and cargo delivery.

Carbon nanohoops (CNHs) belong to a new class of CNMs and can be thought of as singular cross sections of CNTs (Figure 1D). CNHs are composed of several aromatic rings fused together to form a closed conjugated π-system that resembles a macro-ring structure. Although nanohoops emerged theoretically in 1954, their synthesis was not feasible until 2008, when Jasti, Bertozzi, and colleagues synthesized [9], [12], and [18]-cycloparaphenylenes ([*n*]-CPPs).^78^ This groundbreaking CNHs synthesis has been followed up by various innovative approaches leveraging transition metals to execute reductive eliminations for formation of highly strained CPP macrocycles.^79−86^ Unlike many other π-conjugated CNMs, nanohoops have radially oriented π-systems yielding unique optical, electronic, and charge transport properties,^87^ making them attractive for select bioimaging applications.^88,89^ In terms of optical properties, smaller CNHs demonstrate red-shifted fluorescence due to the narrowing of the HOMO–LUMO (highest occupied molecular orbital–lowest unoccupied molecular orbital) gap.^90^ In addition to size, electron donating and accepting rings also change fluorescent emission properties via solvent-molecule interactions and improve quantum yields.^91^

### 2.4 Graphene, Graphene Oxide (GO), Reduced Graphene Oxide (RGO), and Graphene Nanoribbons (GNRs)

Graphene made its debut in 2004^92^ and the pioneering work of Geim and Novoselov in graphene physics was recognized with the 2010 Nobel Prize. Graphene has unique electronic, magnetic, optical, and thermal properties that make it suitable for a wide range of applications.^93−97^ Composed of a single layer of hexagonal sp^2^ hybridized carbon atoms arranged in a 2-dimensional (2D) sheet, graphene’s highly conjugated π-system is responsible for its electronic properties (Figure 1E). Its zero-bandgap enables effective electron conduction at relativistic speeds,^98,99^ making graphene excellent for electrochemical processes.^100−102^ Graphene is the thinnest and strongest nanomaterial to date with atomic thickness and mechanical stiffness of 1060 GPa.^103,104^

Graphene is synthesized via two main synthetic routes. Top-down approaches include mechanical and chemical exfoliation,^92,105^ unzipping of carbon nanotubes,^106−108^ and chemical synthesis,^109,110^ which are typically used to synthesize smaller graphene lattices (nm up to cm length). For bottom-up approaches, CVD^111−113^ and epitaxial growth^114−116^ are preferred to synthesize larger graphene lattices (up to several cm in length).

GO is comprised of a graphene parent structure, and additionally contains hydroxyl (−OH) and epoxide functional groups (C–O–C) on the longitudinal plane, and carbonyl oxygens (=O), ethers (−O−), and carboxylic acids (O=C–OH) at the edges^117,118^ (Figure 1F). These chemical modifications contribute to GO’s solubility in polar protic and polar aprotic solvents,^119^ and give rise to photoluminescence properties that broaden its applications. GO’s decoration with oxygen-rich moieties also results in a p-doping effect and lowers its Fermi level, which facilitates development of artificial optoelectronic systems that mimic naturally occurring biological phenomena.^120,121^ Other common applications of GO include drug delivery,^122,123^ antimicrobials,^124,125^ fluorescent probes for biological sensing,^126^ and cancer biomarker detection.^127^

Until recently, synthesis and homogeneous functionalization of highly crystalline GO was not feasible, where synthesis mostly relied on the direct oxidation of graphite to produce graphite oxide followed by an exfoliation process.^128^ High degrees of crystallinity translate to decreased amounts of defect sites and thus improved electrical conductivity and resistance to oxidation. Toward this goal, a route for highly crystalline GO synthesis has recently been developed, achieving a >99% monolayer ratio with uniform epoxy modification and minimal lattice defects,^117^ advancing the robust use of GO in many applications. Even though this approach currently only works for epoxy modification, its translation to other surface modifications with high crystallinity will be highly enabling.

Another recent CNM of interest is graphene nanoribbons (GNRs). These small strips of graphene typically have width to length ratios of 1:10, and with recent advancements in GNR synthesis, widths less than 10 nm have been achieved.^129^ The GNR band gap is governed by its size, making narrow GNRs desirable. Similar to other CNMs, top-down synthesis methods involve the fragmentation of larger carbon allotropes, such as CNTs and graphene. Specifically, CNTs can undergo plasma etching, where a localized and controlled exposure to plasma induces their unzipping and facilitates the formation of smaller GNRs.^130^ Other synthesis approaches include lithographic^131^ and sonochemical^132,133^ methods for the formation of GNRs from graphene, which also requires etching for the controlled removal of atoms from higher ordered graphene. Bottom-up synthesis of GNRs has been reported. Halogenated aromatic substrates are some of the first chemical precursors to be used for bottom up GNR synthesis.^134^ These chemical moieties provide avenues for the selective polymerization of smaller aromatic building blocks via radical addition reactions at high temperatures. More recently, novel methods for the synthesis of GNRs exploited the use of transition metals for the selective polymerization of smaller building blocks,^135,136^ offering tighter control for the formation of thinner GNRs.

### 2.5 CNM Characterization Methods

In this section, we describe the most prevalent CNM characterization techniques including methods used for chemical identification, morphological, structural, optical, size, and surface charge characterization. Table 1 provides a comparative overview of these techniques and their limitations to guide readers to choose the most suitable characterization method for a given CNM and property type.

**Table 1:** Comparison of CNM Characterization Methods

Method	Typical Information Provided	Considerations for Use	Ref
FTIR	Chemical functional groups pre- and post-modification for all CNMs	• Non-destructive, real-time, simple, and fast	(1, 39, 187)
		• Availability of extensive reference spectra	
		• Requires relatively large amount of sample	
		• Does not provide quantitative information	
		• Peaks can be hard to distinguish from background in some CNMs (e.g., pristine C_60_)	
Raman	CNTs: chirality, diameter, defects	• Diverse information, simple, non-destructive	(138, 142, 188−190)
	CDs, CNHs, CNCs: band gap	• The ratio of D- and G-bands can give quantitative measure of defect density	
	Graphene: layers and defects	• Spectra can be hard to deconvolute given limited availability of reference spectra	
	GO: chemical structure, doping, band gap	• Higher spatial resolution, wider field of view, and faster scan rates are needed	
XPS	Surface elemental composition and chemical environment of surface species (e.g., CF vs CF_2_)	• Higher sensitivity compared to FTIR	(191−195)
		• Provides quantitative composition information	
		• Requires relatively large sample amount and ultrahigh vacuum conditions	
		• Deconvolution of peaks can be ambiguous and requires prior insight on functional groups present	
NMR	CNTs, CNHs, CNCs: chemical structure	• Non-destructive analysis of both solid and liquid samples	(80, 149, 196−200)
	CDs: chemical modification and purity	• Quantitative chemical composition results	
		• High resolution at the atomic level	
		• Difficult to determine structure in large and complex CNMs due to many peaks	
		• Low isotopic abundance of ^13^C limits sensitivity	
SAXS	SWCNTs: morphology, diameter	• Small sample amounts needed and fast	(153, 154, 201−203)
	MWCNTs: nanotube alignment	• Quantitative characterization of metastable systems with multiple conformations	
	Graphene, GO: molecular mass and physical properties	• Cannot reconstruct 3D structure from 1D data and only offers surface-level insights	
		• Lower resolution compared to electron microscopy	
		• Often requires use of a synchrotron facility	
SANS	Structure, morphology, porosity, total internal surface of CNMs	• Higher penetration compared to SAXS, better suited for multilayered CNMs (e.g., MWCNTs)	(15, 51, 58, 204)
		• Preserves sample integrity	
		• Higher contrast between CNM and solution	
		• Often requires use of neutron facilities that are sparsely available	
		• Measurement time can be long	
AFM	Surface morphology, size and height of CNMs pre- and post-modification, determination of cargo loading	• Analysis of both solid and liquid samples	(159, 205−209)
		• Higher resolution than SEM, providing 3D surface topography at nm lateral and sub-Å vertical resolution	
		• No need for vacuum, non-destructive	
		• Lower scanning areas (μm^2^) than electron microscopy	
		• Slow scan speeds	
SEM	Surface morphology of all CNMs	• Can scan larger area (mm^2^) than AFM and has large depth of field, suitable for imaging rough samples	(206, 207, 210−213)
		• Can be combined with other approaches to provide elemental composition analysis	
		• Has lower resolution than AFM and cannot provide 3D information	
		• CNMs typically have low contrast in electron microscopy compared to other nanoparticles	
		• Requires vacuum conditions	
STM	Surface morphology of conductive and semiconductive CNMs	• Provides sub-angstrom resolution in all three dimensions	(167, 214−216)
		• Requires conductive or semiconductive samples, problems when π-conjugation is disrupted (e.g., GO)	
		• Requires vacuum conditions	
TEM	CNTs: inner and outer tube morphology	• Can be combined with other approaches to provide elemental composition analysis	(217−220)
	CDs: size, graphene lattice spacing	• Can give crystal structure information	
	CNCs: surface morphology	• High spatial resolution of 0.05 nm	
	Graphene, GO: lattice spacing, surface morphology	• 2D image can offer insights on size and lattice spacing but cannot give 3D structure	
		• CNMs typically have low contrast in electron microscopy compared to other nanoparticles	
		• Requires vacuum conditions	
UV–vis-IR	Absorption and emission spectra, quantum yield, photophysical properties for optically active CNMs, and purity	• Non-destructive, real-time, simple, and fast	(221−226)
		• Equipment readily available for UV–vis region	
		• NIR region requires expensive equipment for characterization (e.g., SWCNTs)	
DLS and ZETA	Size and surface zeta potential of CNMs, dispersity and colloidal stability of CNMs	• Real-time, simple, and fast	(227−232)
		• Nondestructive for hydrodynamic size, but destructive for zeta potential measurement	
		• Colored or fluorescent samples may skew the results, though there are newer equipment available to overcome this	
		• Can only be used for spherical CNMs for accurate measurement, but algorithms could be adjusted for other shapes	

#### 2.5.1 Chemical Identification

**Figure 2:**
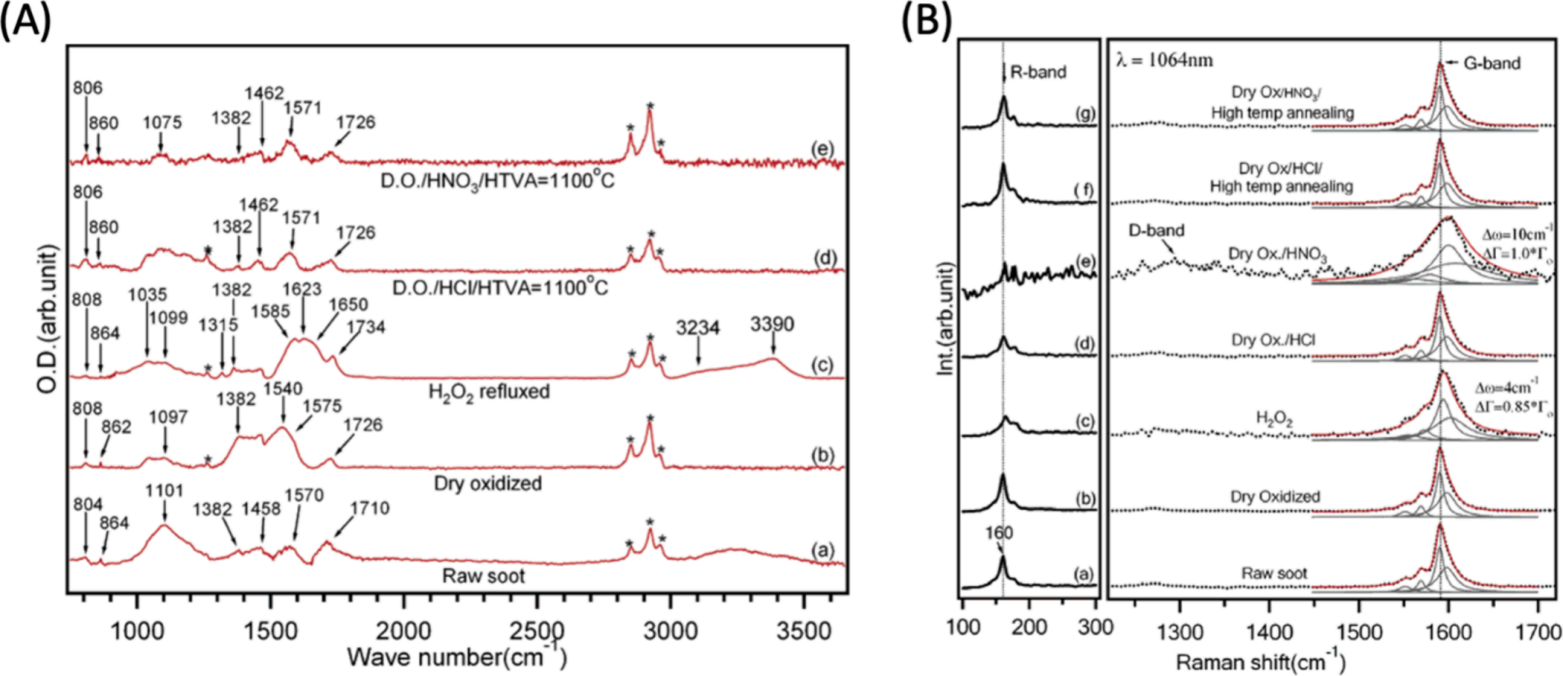
FTIR and Raman characterization of different preparations of SWCNTs. (A) FTIR spectra for raw SWCNT soot (a), dry oxidized (b), H_2_O_2_ refluxed (c), purified material via HCl and high-temperature vacuum anneal (HTVA) treatment at 1100 °C (d), and purified material via HNO_3_ and HTVA treatment at 1100 °C (e). The top two spectra on purified SWCNTs represent the cleanest material. (B) Raman spectra for the same SWCNT types from (A) showing R-band (100–300 cm^–1^) region (left panel) and D- and G-band region (1230–1750 cm^–1^) (right panel). Reproduced from ref (138). Copyright 2005 American Chemical Society.

Methods for characterizing CNM chemical identity include spectroscopic approaches such as Fourier-Transform Infrared (FTIR) and Raman. FTIR measures the vibrations of atoms and bonds when they absorb infrared light (IR) at various wavelengths providing information about the chemical functional groups present within a material (Figure 2A). Even though it is more common for the characterization of CDs,^137^ CNTs,^138^ and GO,^139^ it can also be used with other CNMs as a standard characterization tool.^140,141^ Another technique that relies on IR and vibrational frequencies to provide a molecular fingerprint is Raman spectroscopy. Raman utilizes scattering of light (instead of absorbance in FTIR) to measure intrinsic chemical properties of CNMs, and can provide information on the mass density, optical energy gap, elastic constants, doping levels, presence of defects, and other forms of crystal disorder. It also offers insights into the edge structure, strain, number of graphene layers, nanotube diameter, chirality, and curvature of CNMs (Figure 2B).^142^ FTIR and Raman distinguish different bond types, each with unique limitations and strengths. Therefore, they provide a comprehensive chemical identification of CNMs when used in combination.

**Figure 3:**
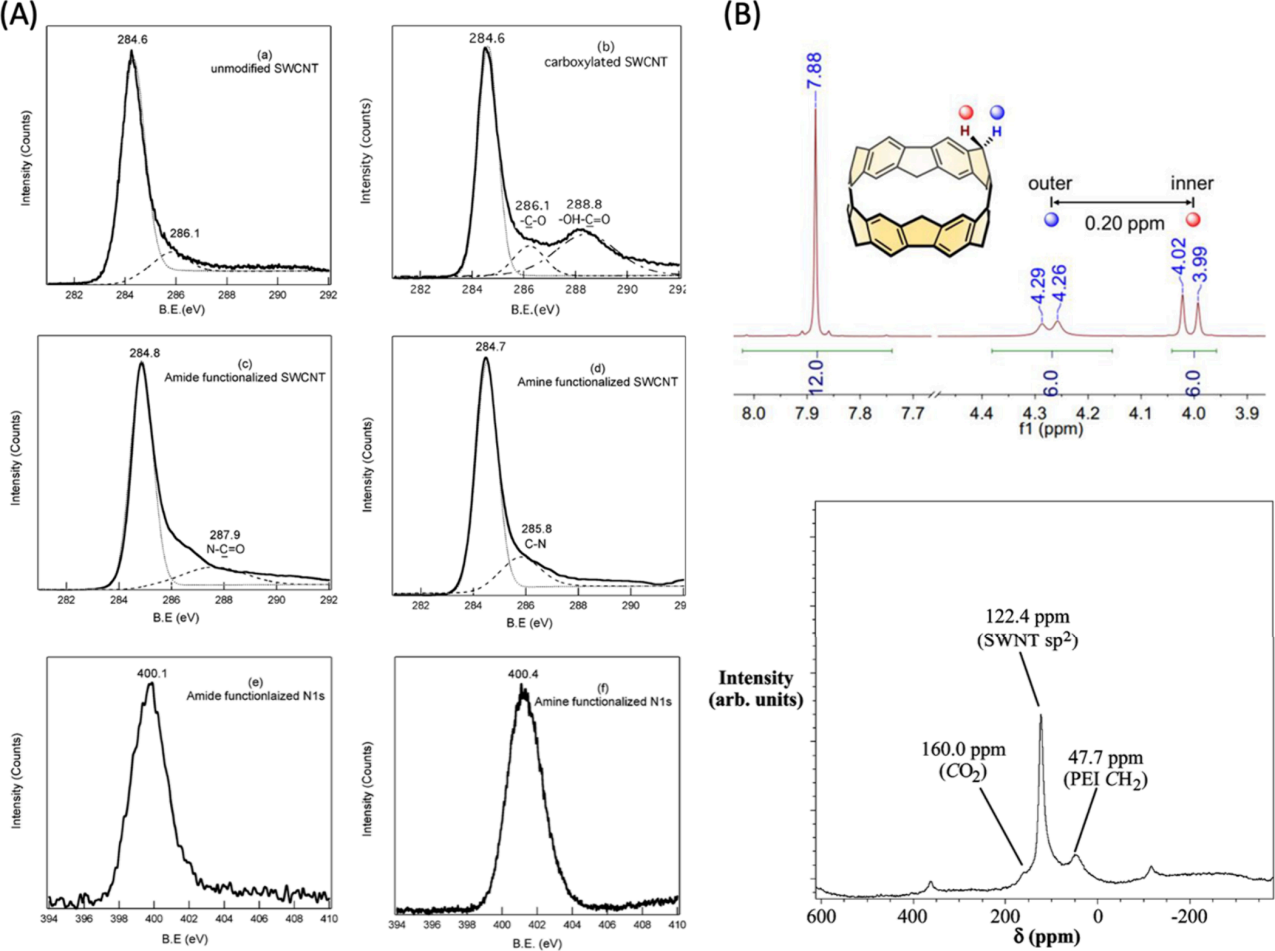
XPS and NMR characterization of CNMs. (A) XPS spectra of (a) unmodified SWCNT C 1s, (b) carboxylated C 1s, (c) amide-functionalized C 1s, (d) amine-functionalized C 1s, (e) amide-functionalized N 1s, (f) amine-functionalized N 1s. Reproduced from ref (150). Copyright 2005 American Chemical Society. (B) (Top panel) ^1^H NMR spectrum of Methylene-Bridged [6]CPP. Reproduced from ref (151). Copyright 2020 American Chemical Society. (Bottom panel) Solid-state NMR spectra of polyethylenimine (PEI)-functionalized SWCNTs via ^13^C MAS NMR spectrum with a 12 kHz spinning speed. Reproduced from ref (152). Copyright 2008 American Chemical Society.

Other common methods for chemical identity characterization of CNMs include X-ray photoelectron spectroscopy (XPS) and nuclear magnetic resonance (NMR). XPS enables quantitative analysis of elemental composition of most CNMs. It uses high energy X-ray photons to ionize electrons within an atom to provide data on electron binding energies associated with specific atoms within the specimen. For this reason, XPS is one of the standard methods of characterization of most CNMs and their surface modifications (Figure 3A).^27,143,144^ In addition to XPS, NMR spectroscopy allows for the characterization of the molecular structure, specifically of carbon and hydrogen atoms via ^13^C NMR and ^1^H NMR, and can offer information on certain surface-modified CNMs (CDs, CNTs)^145−147^ and their relative purity (Figure 3B).^148^ However, one major limitation is the difficulty of interpreting properties of the whole CNM being analyzed given their large and complex carbonaceous structures. Therefore, relatively small CNMs, such as nanohoops, are more frequently characterized via NMR.^80,149^

#### 2.5.2 Morphological and Structural Characterization

**Figure 4:**
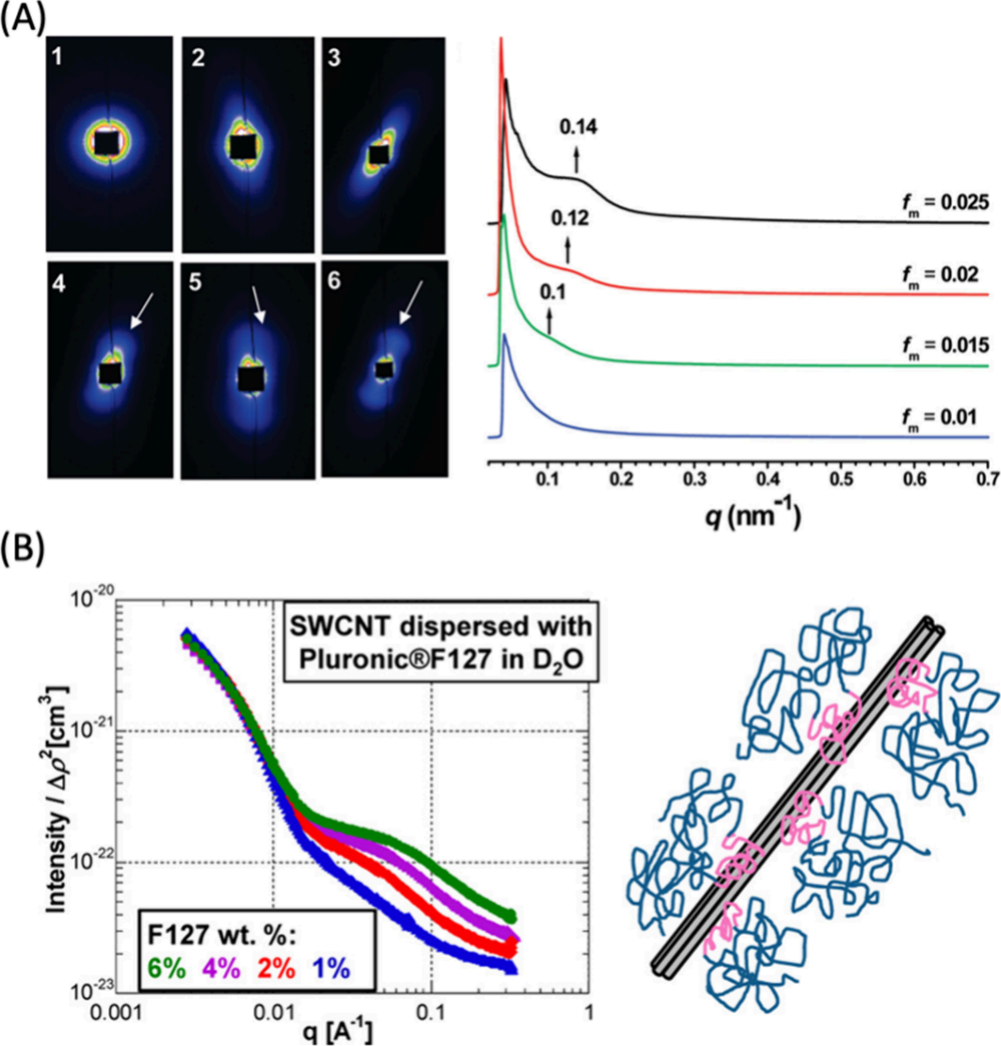
SAXS and SANS characterization of CNMs. (A) (Left panel) SAXS patterns of GO aqueous dispersions with maximum mass fraction (*f*_m_’s) of 2.5 × 10^–4^, 5 × 10^–3^, 1 × 10^–2^, 1.5 × 10^–2^, 2 × 10^–2^, and 2.5 × 10^–2^, from 1 to 6. The white arrows indicate the diffuse arc and the scattering peak. (Right panel) SAXS profiles of liquid crystals of GO with high concentrations. The spectra depict the scattering intensity as a function of scattering vector *q* (*q* = (4π sin θ)/λ, where 2θ is the scattering angle). Reproduced from ref (157). Copyright 2011 American Chemical Society. (B) (Left panel) SANS patterns of SWCNTs dispersed in Pluronic F127 in 100% D_2_O, at four different concentrations of 6, 4, 2, and 1% (%w/w). (Right panel) Schematic of dispersed SWCNTs. Reproduced from ref (158). Copyright 2012 American Chemical Society.

Small-angle neutron scattering (SANS) and small-angle X-ray scattering (SAXS) are powerful techniques that utilize the diffraction of high energy particles to investigate the atomic and magnetic structures of CNMs. Both techniques leverage the wave characteristics inherent to particles to analyze the diffraction patterns according to Bragg’s law, thereby obtaining information about the arrangement and organization of atoms in the material’s inner and outer layers. SAXS is particularly well-suited for studying the external surfaces of CNMs, making it ideal for analyzing single-layered materials, such as graphene and SWCNTs (Figure 4A).^153,154^ On the other hand, SANS, by using neutrons, possesses greater penetration capabilities and is less destructive, allowing the investigation of bulk materials and determination of internal structures without causing significant decomposition (Figure 4B). Consequently, SANS is especially useful for exploring the chemical structures deeply embedded within nanomaterials like MWCNTs.^155^ While SAXS and SANS provide distinct benefits, they also complement each other by offering insights on chemical structures in different regions of CNMs. Therefore, researchers often employ both techniques in tandem to gain a comprehensive structural understanding of CNMs. However, it must be noted that SANS does have an advantage over SAXS as it provides higher contrast between the sample and its solvent through contrast matching. In SAXS, contrast matching is also possible but requires the chemical modification of the nanoparticles making it more challenging to perform.^156^

**Figure 5:**
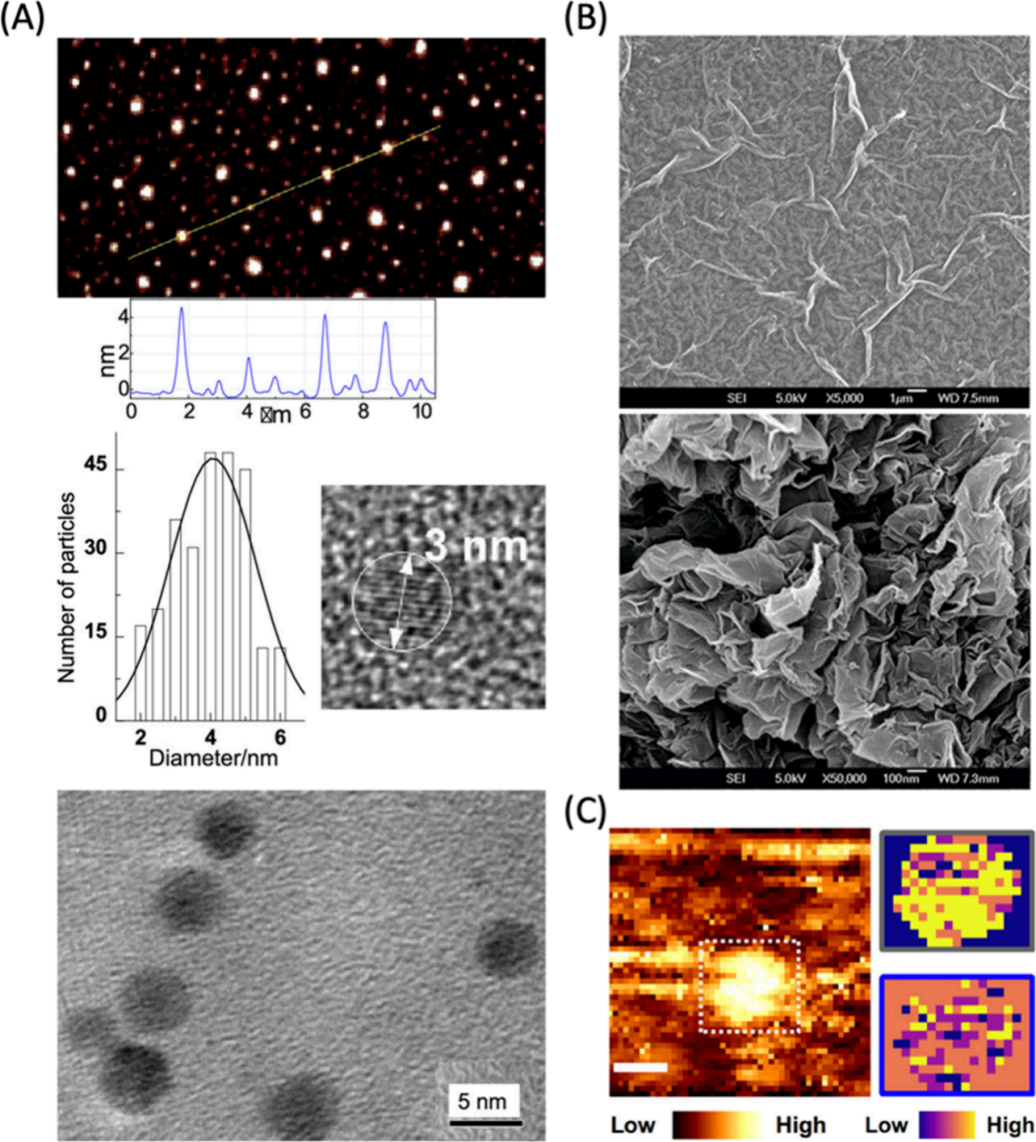
AFM, TEM, SEM, and STM characterization of CNMs. (A) (Top) AFM image of CDs with height profiles of some dots along the highlighted line. (Middle left) Size distribution based on AFM height analyses, (middle right) a high-resolution TEM image of CDs illustrating the carbon core (Bottom) TEM image of the gold-doped CDs. Reproduced from ref (173). Copyright 2014 American Chemical Society. (B) SEM images of GO and rGO nanosheets. Reproduced from ref (174). Copyright 2011 American Chemical Society. (C) (Left) Topographic STM image of a CD in the dashed white box with scale bar of 5 nm and colormap indicating STM height. (Right) PCA and k-means clustering of the tunneling spectroscopy data reveal low (blue) to high (yellow) density of states showing localized defects of about 1–2 nm in diameter. Reproduced from ref (175). Copyright 2020 American Chemical Society.

Other techniques utilized for morphological characterization of CNMs are atomic force microscopy (AFM), scanning electron microscopy (SEM), scanning tunneling microscopy (STM), and transmission electron microscopy (TEM). AFM measures the intermolecular forces between a sharp probe and a sample to afford an image of the surface of CNMs. AFM is routinely used for CNTs,^159^ CDs,^160^ graphene,^161^ and GO^162^ to provide high resolution images of CNM surface at the nanometer and even atomic scale and is commonly used to verify the loading of macromolecules (Figure 5A). SEM focuses a high energy electron beam to a sample to measure the secondary electron emissions. It is commonly employed for the characterization of CNTs,^163^ CDs,^164^ graphene,^161^ and GO^165^ and provides a detailed analysis of surface features, such as roughness, texture, and the presence of defects (Figure 5B). STM also visualizes surface characteristics of CNMs by using a sharp conductive probe positioned closely to a sample, which tunnels electrons via quantum tunneling when a bias voltage is applied. Consequently, small aberrations in the tunneling currents can be translated into height differences in sample surface, revealing atomic and molecular features of CNTs,^166^ CDs,^167^ graphene,^168^ and GO^169^ (Figure 5C). TEM, on the other hand, is routinely used for the imaging of internal and morphological structures of CNMs. It transmits high energy electrons through a thin sample to visualize internal and peripheral structural characteristics of CDs,^162,170^ CNTs,^163^ graphene,^171,172^ and GO^162,169^ (Figure 5A).

#### 2.5.3 Optical Characterization

**Figure 6:**
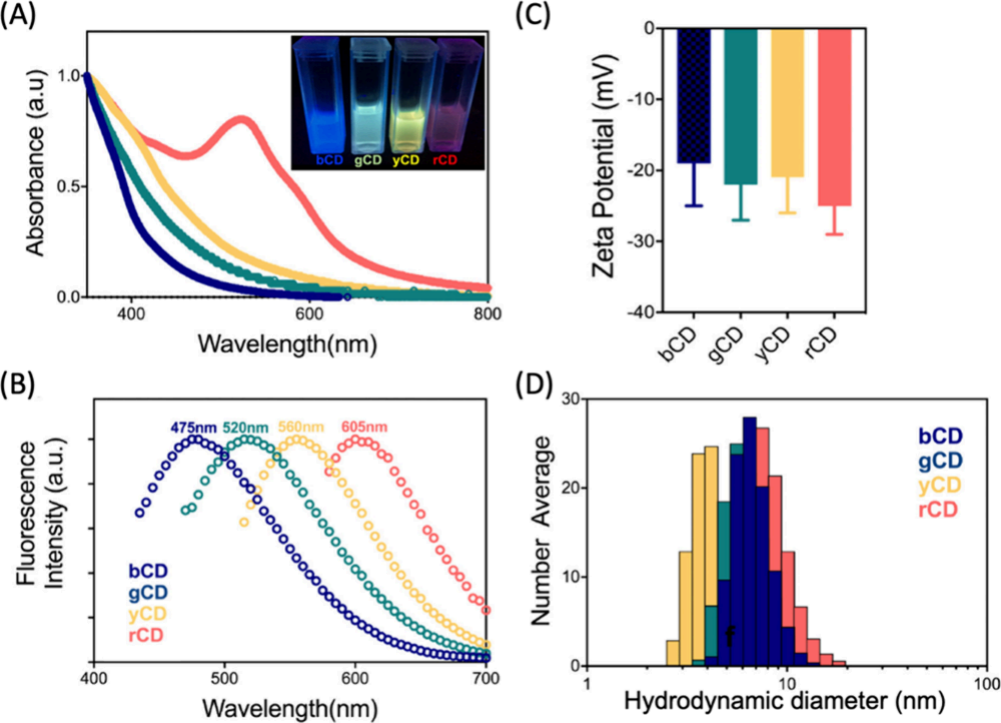
UV–vis-IR, zeta potential, and size characterization of CNMs. (A) UV–vis absorption spectroscopy of CDs with increasing number of oxygen-containing defects (blue to red CDs). (B) Fluorescence emission spectra of the four fractionated CD samples. (C) Zeta potential measurements of four CDs. (D) Hydrodynamic diameter measurements by DLS indicate no size trend of blue to red CDs. Reproduced from ref (175). Copyright 2020 American Chemical Society.

CNMs possess extraordinary optical properties that position them as highly useful fluorescent probes. Ultraviolet–visible-near infrared (UV–vis-NIR) spectroscopy is a powerful tool elucidating the diverse light absorption and fluorescent emission profiles of CDs,^45,51^ CNTs,^25,31^ nanohoops,^78,82,88^ nanoribbons,^133,135^ fullerenes,^176^ and GO.^177^ UV–vis-NIR characterization facilitates the acquisition of comprehensive chemical absorption profiles, an invaluable technique to determine quantum yields (Figure 6A). Moreover, when paired with emission profiles, it provides insights for the fine-tuning of the fluorescent properties of CNMs, particularly important for those that have undergone selective chemical modifications (Figure 6B). Specifically, UV–vis is routinely used for the quantification of quantum yields for highly fluorescent CDs.^178^

#### 2.5.4 Surface Charge and Size Characterization

Zeta potential, which is a measure of the magnitude of the electrostatic repulsion or attraction between particles, is routinely used to determine the surface charge and colloidal stability of CNMs. It is based on applying a voltage to the CNM solution and measuring the particle velocity as a function of voltage as particles move toward the electrode of opposite charge (Figure 6C). Zeta potential can be specifically useful to validate the attachment of moieties and biological cargoes if they have a charge different from the nanoparticle core. Zeta potential also indicates the stability of colloidal nanoparticles, where it is commonly accepted that particles with zeta potential <30 and >30 mV are colloidally stable.^179^

Dynamic light scattering (DLS) measures the hydrodynamic size distribution of nanoparticles. By exploiting the inherent Brownian motion of particles, DLS analyzes fluctuations in scattered light intensity, enabling accurate particle size and polydispersity estimation^180−182^ (Figure 6D). The measurements and algorithms of a typical DLS are optimized for spherical particles but they could be modified for rod-shaped CNMs. The synergic use with electron microscopy and AFM enhances the characterization by providing insights into the particle geometry, aggregation, and morphology. DLS finds broad applicability in the investigation of many CNMs, such as CDs,^183^ fullerenes,^184^ graphene,^185^ and GO.^186^

## 3 Material Properties of CNMs

In this section, we discuss important photophysical (Table 2), mechanical (Table 3), and electronic properties (Table 4) of above-mentioned CNMs, specifically focusing on how these properties affect their biological applications.

**Table 2:** Summary Table of CNM Photophysical Properties

Material	Excitation (nm)	Emission (nm)	Quantum Yield (%)	Ref
SWCNTs	250–900: 1 photon	800–1600	0.01–1	(408−416)
	1560: 2 photon			
Carbon dots	230–732: 1 photon	300–820	1–94.5	(417−427)
	690–1400: 2 photon			
Carbon nanohoops	[*n*]CPP – 340: 1 photon	450–600	0.05–83.5	(428−438)
	*m*[*n*]CPP – 328: 1 photon			
	*m*[*n*]CPP – 705–800: 2 photon			
Graphene/GO/RGO	230–300	350–800	1.7–74	(439−451)

**Table 3:** Summary Table of CNM Mechanical Properties

Material	Surface Area (m^2^ g^–1^)	Young’s Modulus	Thermal Conductivity	Ring Strain(kcal mol^–1^)	Ref
SWCNTs	1315	0.32–1.47 TPa	1750–7000 W m·K^–1^	–	(509−514)
Carbon dots	0.0667–2.5747	–	0.1–21.65%	–	(515, 516)
Carbon nanohoops	503	14–41 GPa	0.06–0.265 W m·K^–1^	67–119 kcal mol^–1^	(517−519)
Graphene/GO/RGO	Graphene: 2630	Graphene: 1.0 TPa	Graphene: 1500–5000 W m·K^–1^	–	(520−531)
	GO/RGO: 669–2391	GO/RGO: 0.25 ± 0.14 TPa	GO/RGO: 2–1000 W m·K^–1^		

**Table 4:** Summary Table of CNM Electronic Properties

Material	Carrier Mobility (cm^2^ V^–1^ s^–1^)	Current Density (mA cm^–1^)	Specific Capacitance	Resistance (Ω cm)	Ref
SWCNTs	2–100,000	4 × 10^12^	14.1–180 F g^–1^ at 1 A g^–1^	1 × 10^–6^– 1 × 10^–4^	(593−603)
Carbon dots	8.5 × 10^–5^– 9.9 × 10^–7^	5–500	21–697 F g^–1^ at 1 A g^–1^	0.069–9.92	(604−612)
Carbon nanohoops	–	–	–	–	
Graphene/GO/RGO	Graphene: 2 × 10^5^	Graphene: 1.2 × 10^7^– 4 × 10^7^	Graphene: 12.4–47.8 F g^–1^ at 0.5 A g^–1^	Graphene: 0.3–0.9	(522, 613−616)
	GO/RGO: n/a	GO/RGO: n/a	GO/RGO: 119.6–181.5 F g^–1^ at 0.5 A g^–1^	GO/RGO: 1.1–7.2	

### 3.1 Photophysical Properties

#### 3.1.1 Single-Walled Carbon Nanotubes (SWCNTs)

**Figure 7:**
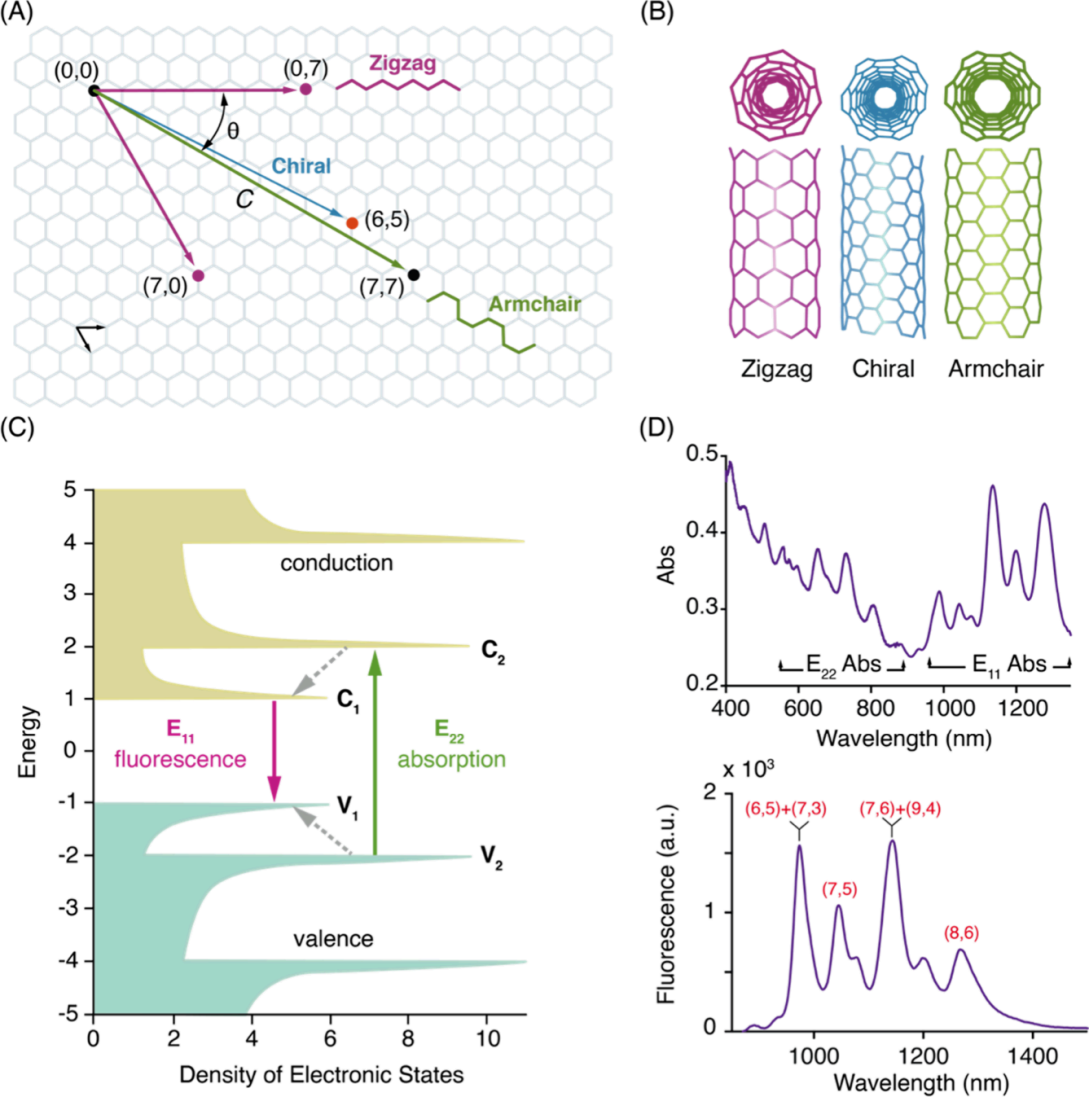
SWCNT photophysical properties. (A) CNTs can be conceptualized as graphene sheets rolled according to unique rollup vectors that determine their optoelectronic properties and give rise to a diversity of species. The direction and magnitude of the rollup vector is often denoted by a pair of indices, (*n*, *m*), which can be thought of as scalar multipliers of the unit basis vectors into which the roll up vector can be decomposed. (B) CNT species can fall within three categories depending on the “twist” of the graphitic lattice. (C) An electronic density of states for a nanotube species of the semiconducting (chiral) type, with a small but nonzero bandgap between the valence and conduction bands. Note the sharp peaks in the density of states, which gives rise to “feature-rich” spectra depicted in (D). Excitation is typically carried out using *E*_22_-lasers, and fluorescence emission is detected with Stokes shift of >100 nm from the *E*_11_ state (equivalent to the first excited stated in molecular spectroscopy). (D) Absorption (top) and fluorescence emission (bottom) spectra from a multichiral (polydisperse) dispersion of single wall carbon nanotubes synthesized by the HiPco method. λ_ext_ = 785 nm is typically used for broad resonant and off-resonance excitation of nanotubes for most imaging applications. Peak assignments for some of the prominent chiralities observed in HiPco samples are shown in red text. For a thorough treatment of optical spectroscopy of SWCNTs, the reader is invited to review Weissman et al.^250^

**Figure 8:**
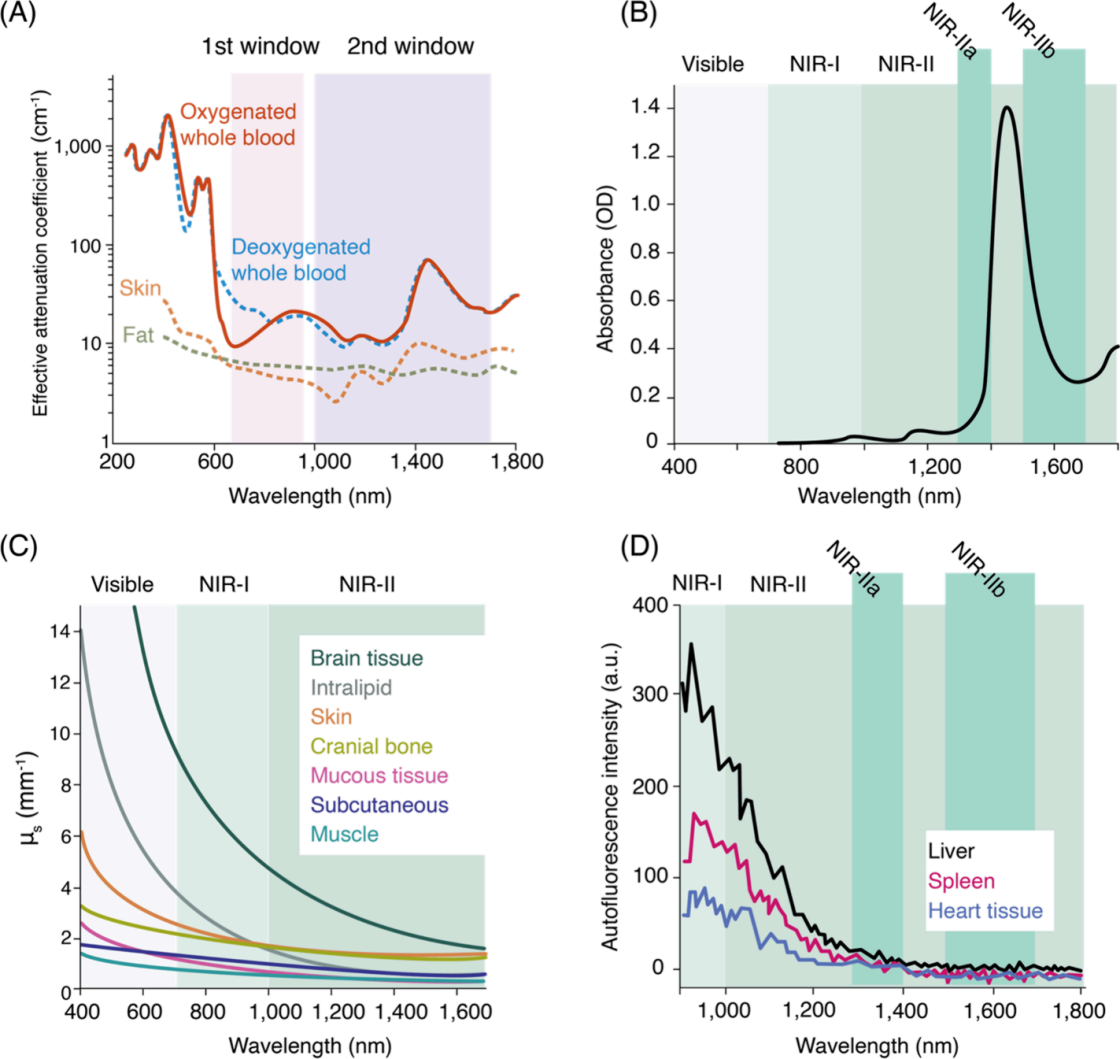
SWCNT photoluminescence in the NIR/SWIR window is coincident with reduced absorption, scattering, and autofluorescence from biological samples. (A) Effective attenuation coefficients of skin and blood in the 1^st^ and 2^nd^ NIR windows. (B) Absorption by water from 400–1800 nm. (C) Reduced scattering coefficients of various biological matrices exhibit monotonic decrease into the NIR/SWIR window. (D) Autofluorescence spectra of *ex vivo* mouse tissues at 808 nm excitation. Reproduced from ref (251). Copyright 2018 American Chemical Society.

Among all CNMs, SWCNTs possess certain unique photophysical properties. Semiconducting SWCNTs are intrinsically fluorescent in the near-infrared/shortwave infrared (NIR/SWIR) regions of the spectrum due to strongly allowed Van Hove transitions that are the main features of their electronic density of states.^233^ The excited state of a SWCNT is characterized by diffusive excitons on the graphitic lattice.^234^ Excitation typically proceeds by absorption of photons in the second conduction band, which rapidly decays to the first, allowing radiative recombination and fluorescence in the NIR region (800–1600 nm)^235,236^ (Figures 7C and 8). This emission is typically sharp and consists of multiple peaks, each corresponding to a specific chirality of a nanotube.^237^ The emission range, coupled with large Stokes shifts and the absence of photobleaching,^238^ have facilitated the use SWCNTs for fluorescence microscopy.^233,239−243^ SWCNTs can enable imaging of depths up to 3 mm^244,245^ because of the reduced absorption and scattering of their fluorescence emission by tissue, bone, water, and blood^246−249^ (Figure 8).

In order to deploy SWCNTs as fluorescence contrast agents, they often need to be functionalized. This is required to make SWCNTs soluble in aqueous mediums of interest and/or to impart biological compatibility. Perturbations to either the SWCNT directly or to the extended supramolecular corona encompassing the SWCNT can impart changes to the photophysics of the material. Covalent attachments are often introduced at the expense of lower or fully eliminated photoluminescence due to the degradation of the sp^2^ network, which can promote non-radiative decay of mobile excitons.^252−256^

**Figure 9:**
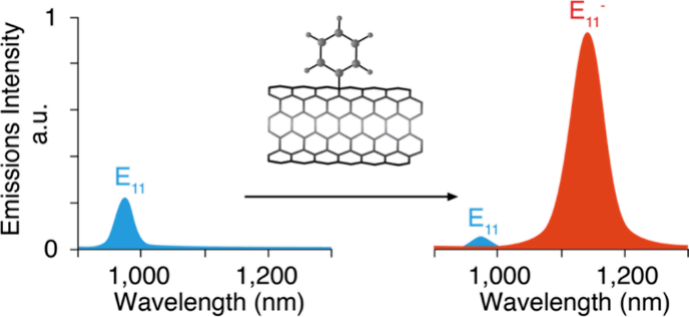
Engineered covalent adducts on SWCNTs allow for tunable fluorescence emission. Note the emergence of a brighter, red-shifted emission peak (*E*_11_^–^) after functionalization with covalent color centers.

One particular type of covalent modification, organic color centers (OCC),^257^ allows for tuning the inherent NIR/SWIR emission of SWCNTs. These centers are synthetic defects that are added to the sp^2^ lattice, causing sp^3^ centers that facilitate emission of bright photoluminescence^258^ that is chemically tunable^258,259^ and single photon in nature.^260^ These centers often trap and localize excitons and increase the probability of radiative recombination.^257^ The introduction of an OCC creates a quantum two-level system, which inherently emits single photons,^261^ with an *E*_11_ and a red-shifted new *E*_11_^–^ fluorescence peak (Figure 9).^257^ Density functional theory (DFT) calculations support that this dipole-allowed transition results from an asymmetric splitting of the frontier orbitals at the defect site.^258^ Introduction of defects has minimal effect on absorption, but can dramatically change the emission spectrum, in which *E*_11_^–^ can become more dominant than *E*_11_.^258,262^

The bright photoemission from OCC-modified SWCNTs arises from the fact that the *E*_11_^–^ optical transition lies below the *E*_11_ dark excitons.^263,264^ The newly formed state allows for these *E*_11_ dark excitons, which normally decay through non-radiative pathways, to be harvested at OCC-sites and allow SWCNT brightness to be increased as much as 28-fold through the *E*_11_^–^ emission pathway.^258,263,264^ As the density of OCCs on the SWCNT are increased, the lifetimes of both bright and dark *E*_11_ excitons become shorter; suggesting both can become trapped at the defect sites.^265^ The energy difference between the *E*_11_ and *E*_11_^–^ corresponds to the D-phonon mode (1301 cm^–1^, 161 meV) caused by the defect, suggesting an exciton–phonon coupling mechanism that can brighten dark excitons.^257^ Position and intensity of the emission can be tuned through installation of aryl OCC with electron-withdrawing or donating substituents. These groups effectively adjust the HOMO and LUMO levels at the defect sites and have been demonstrated to have a linear correlation between *E*_11_^–^ shift and the Hammett constant of the OCC group.^258,266^

Similar to OCCs, oxygen dopants have also been demonstrated to produce red-shifted emission.^267−270^ This emission is temperature dependent, suggesting low-lying dark state exists below the optically allowed states,^267^ which was supported with DFT calculations.^271^ These and other SWCNT covalent modifications are more extensively discussed in Section 4.

#### 3.1.2 Carbon Dots (CDs)

CDs are fluorescent nanoparticles that can absorb and emit photons that cover the entire UV–vis spectrum,^272^ but typically show strong absorption in the UV region (230–300 nm).^273^ Fluorescence excitation in CDs can be achieved over a broad range of excitation wavelengths^274,275^ and is tunable with a shifting emission that is a function of the excitation wavelength.^276^ Quantum yield of CDs can vary widely with reported values ranging from approximately 1%^277,278^ to 94.5%.^279^

The photoluminescence mechanism of CDs is still not well-understood and is highly debated. It is complicated by the fact that a wide range of carbon containing materials are used as precursors for CD synthesis. Multiple disciplines investigate CDs with non-harmonized techniques and measurement parameters that are often focused on discipline-specific applications. Additionally, the inherent complexity of the photoemission that likely involves multiple pathways further complicates the study of CDs.^280−282^ These have led to several contrary findings during the characterization of CDs.^280^ While several different mechanisms have been proposed for CD photoluminescence,^280,281,283^ many studies attribute the emission to surface electronic states,^280,284−288^ which can be strongly influenced by surface group functionality. CDs are inherently surface-functionalized with polar groups formed during their synthesis. Some studies claim that fluorescence originates from oxygen groups on the surface,^289^ others attribute it to the nitrogen groups,^290^ core electronics,^291^ CD crystallinity,^292^ or to the presence of fluorescent molecules on the surface^275,293,294^ or in solution^295,296^ that are inadvertently formed during synthesis.^294,297−300^

Some studies have attributed the origin of CD emission to quantum confinement effects, involving band-to-band transitions or intrinsic emission.^301−304^ In such a system, the emissive component is thought to be a nanometer sized, sp^2^-conjugated domain that is only emissive at very small sizes, possibly the core or a portion of the core.^280^ Several groups have noted emission is correlated with size, with the small CDs being blue-shifted and larger being red-shifted; as expected with the quantum confinement model.^301,302,304^ However, other groups have observed the opposite trend, with decreasing size producing red-shifting.^305,306^ On the other hand, many groups have posited that photoemission may arise from extrinsic contributions, particularly on the surface in the form of defects, charge traps,^307,308^ or molecular-like states.^302^ This position is supported by the need to appropriately passivate the surface to achieve bright CDs,^309^ and fluorescence being strongly influenced by pH,^310,311^ solvents,^312,313^ and degree of surface oxidation.^287,314^

Despite the uncertainty in mechanism of emission, CDs typically have very strong fluorescence,^274,315−318^ which is tunable^307,317,319−322^ and sensitive to its local environment (e.g., solvents,^312,323,324^ ions,^325,326^ pH,^310,327^ and other particles^328−331^). Emission bands are very broad with their full width half-maximum approximately 50–100 nm.^332^ Fluorescence often occurs only when the nanoparticles are well dispersed^330,331^ and emission efficiencies decrease at longer wavelengths.^273^ Single dots usually have narrower emission spectra.^313,333^ Gosh and co-workers demonstrated the loss of tunability at the single dot level along with loss of emission multiexponential decay that are suggestive of presence of multiple emitters.^333^ This suggests that individual dots may be individual emitters of a particular wavelength and the typical broad spectrum observed is due to an overlay of many dots emitting at once.^333,334^ This has been contradicted by other groups claiming tunability at single dot level,^272^ but it is difficult to discriminate whether or not true single dots are present or emissions arose from small number of CD nanoparticles that may be aggregating.^335^ Related to this, Kang and co-workers showed that different fractions collected from size exclusion chromatography purification of CDs display non-tunable emission of a specific color that is a function of size.^301^ Wen and co-workers used this same size exclusion protocol to isolate different sizes with the same emission, suggesting that the tunability of emission is associated with the heterogeneity of CDs produced during synthesis (i.e., differences in core and shell densities, size, surface functional groups).^305^ Similar separations with high-performance liquid chromatography (HPLC), based on surface functionality rather than size, also produced individual fractions with specific non-tunable emissions.^316^

Early reports of CDs claimed little to no photobleaching after several hours of continuous irradiation.^277,307,336,337^ However, recent data reported considerable photobleaching of CDs.^338−340^ For instance, Wang and co-workers noted an approximately 8% drop in fluorescence intensity after 17 h (365 nm, 950 μW cm^–2^) and that the quantum yield remained constant and did not decrease below a certain threshold even when irradiating longer. When purged with nitrogen before and during measurements, or treated with a reducing agent (e.g., ascorbic acid) or poly(methyl methacrylate) during drop-casting, photobleaching slowed. Interestingly, Wang et al. noted an approximately 50% fluorescence reduction when drop-cast on SiO_2_ substrates and irradiated (532 nm, 1.68 mW, 0.7–0.8 μm spot).^339^ Moreover, Zhi and co-workers synthesized CDs with different quantum yields and observed that when irradiated with UV light, CDs with the highest quantum yield showed the largest reduction in absorbance.^341^ Longo and co-workers also conducted a photobleaching study, where CDs were irradiated with a laser, with known pulse duration, repetition rate, and energy per pulse. Using 5 ns pulses, they observed an emission intensity decrease of 10% after 500 pulses. As they performed the experiment at different energies per pulse, they observed that the bleaching rate varied linearly with power. When varying wavelength, they noted the photobleaching was maximized at wavelengths close to the absorption peak.^340^ Javed and O’Carroll have provided an extensive summary of CD emission studies in their review.^281^

A unique photophysical property of some CDs is blinking, which makes them attractive for applications like super-resolution microscopy. Das and co-workers observed multiple fluorescent intensities attributed to a multichromophoric system when observing immobilized CDs in poly(vinyl alcohol) (PVA) on glass (561 nm, 78 W cm^–2^ and 0.3 kW cm^–2^).^342^ Khan and co-workers observed blinking in CDs immobilized on coverslips in the presence of ascorbic acid or methyl viologen. In the presence of ascorbic acid, an electron donor, CDs underwent a single-step photobleaching. While in the presence of methyl viologen, an electron acceptor, CDs underwent blinking with long-lived dark states.^343^ Chizhik et al. also studied blinking using an epi-fluorescence microscope (473 nm, 500 W cm^–2^) and measured temporal fluctuations in fluorescence and off-state duration for individual particles. They observed on and off states to vary widely for individual particles. Their experimental data fit well with a power law function, something common in semiconductor nanocrystals, where blinking is caused by trapped charge on the surface or within particles.^333,344,345^

Besides blinking, some CDs are able to act as photoswitches; that is, they are able to be turned off then recover completely^346^ or partially^347^ after exposure to a wavelength of light usually shorter than the light used to turn them off. In a study by Kahn and co-workers, CDs with red emission immobilized in PVA on glass decayed after a few seconds of exposure to a 639 nm laser. The emission was then regained via excitation with a 401 nm laser. The authors explained this behavior as CDs being able to be excited by a short wavelength laser and return to ground state emitting a photon. Once in the ground state, CDs can also be excited with a longer wavelength laser, which can cause them to undergo an intersystem crossing and end up in an off state. Then, to return to ground state, they must be excited with energy that is greater than their band gap.^343,346,348^

#### 3.1.3 Carbon Nanocones (CNCs)

CNCs do not have an inherent photoemission of their own. They gain photophysical properties of interest for imaging upon conjugation to other systems (e.g., metals, dyes, aptamers, etc.).^349,350^ A common use of CNCs involves coupling to other photoactive molecules such as porphyrins^349,351−357^ or β-cyclodextran.^358^ In some of these applications, the CNC can act as an electron acceptor.^357^ Pristine CNCs have a Raman spectrum with two peaks of almost equal scattering strengths. The G-band, assigned to E_2g_-like vibrations, occurs at 1593 cm^–1^ and the D-band, assigned to A_1g_-symmetry modes, at 1341 cm^–1^.^359^

#### 3.1.4 Carbon Nanohoops (CNHs)

Cycloparaphenylene (CPP) carbon nanohoops can be conceptualized as a cross-section of a carbon nanotube that is one aryl ring thick, connected to other aryl rings in the *para* positions (i.e., 1,4 linkage), and maintain sp^2^ hybridization. CPPs are typically denoted as [*n*]CPPs where *n* is the number of phenylene units linked together.^360^ All *para*-linked CPPs share a common absorbance maximum at approximately 340 nm attributed to a symmetry-forbidden HOMO to LUMO electronic transition. Emissions range from approximately 450–600 nm with smaller ring sizes producing more red-shifted emission.^360,361^ Smaller ring sizes of *para*-linked CPPs (e.g., *n* = 5,6) are non-emissive due to their inability to break molecular orbital symmetry in their excited state (see Section 3.3. for more on electronic properties). By changing the linkage of a single aryl ring to a *meta*-linkage (i.e., 1,3 rather than 1,4) emission from smaller rings (e.g., *n* = 5,6) can be achieved.^362^ Incorporating the *meta*-linkage into a CPP ring of any size produces a brightness comparable to or brighter than its *para*-linked analog, and blue-shifts the common absorbance to approximately 328 nm.^360,361,363−368^

Having a common absorbance and a large Stokes shifts of 100–200 nm make CPPs attractive for multiplexing because a single excitation source can be used to excite multiple species.^361,369−371^ Strikingly, unlike most organic small-molecule fluorophores, CPPs retain the same bright emission in both solution and solid state,^372−374^ allowing them to be used in various flexible devices.^375,376^ Additionally, variations of CPPs, such as the water-soluble sulfonate-modified [8]CPPs, have shown constant emission intensities over a wide pH range (pH = 3–11).^377^ Another interesting photophysical property observed in solid [10]CPP, when loaded with I_2_ guest molecules, is a broadened white-light emission profile that contrasts the green-blue emission profile prior to the application of an electrical stimulus that can induce a phase transition.^378^ Moreover, modifications to CPPs can lead to emission shifts. Upon oxidation, CPP peak fluorescence is red-shifted. Some CPPs, such as [6–9]CPP^2+^, are capable of weak NIR emission (900–1300 nm).^379^

#### 3.1.5 Graphene, Graphene Oxide (GO), and Reduced Graphene Oxide (RGO)

**Figure 10:**
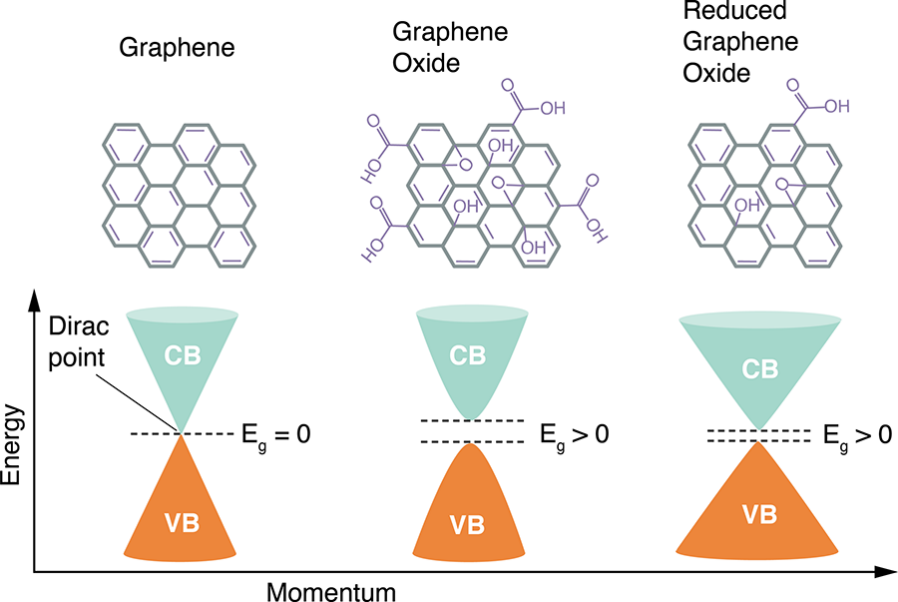
Electronic density of states for graphene, GO , and RGO. Conduction band (CB) is shown in blue and valence band (VB) is shown in orange. Notice the absence of bandgap in graphene vs graphene oxide. Adapted with permission from ref (385). Copyright 2018 Springer Nature under CC BY. http://tinyurl.com/yuh4xfa4.

Graphene is a 2D CNM that finds useful applications in many fields. Graphene is mostly transparent in the visible light spectrum,^380^ but does have intraband transitions^381,382^ and optical phonon–electron coupling,^383^ and is considered a zero-bandgap semiconductor or metal due to its 2D symmetry.^384^ This means electrons that are promoted to an excited state will relax down non-radiatively.^384^ (Figure 10) To utilize graphene in optical applications, a decrease in dimensionality is needed to form a bandgap via quantum confinement.^384^ To achieve this, while maintaining the graphitic structure, graphene nanoribbons (GNRs) and oxidized versions of graphene, GO and RGO, have been produced. The RGO is a less oxidized version of GO, in which some of the sp^2^ bonds present in the pristine graphene have been restored through reduction.

Functionalization via oxidation to form GO results in a bandgap between the valence and conduction bands. This allows GO to absorb in UV with a π → π* transition occurring at approximately 230 nm and a shoulder n → π* transition occurring at approximately 300 nm.^386−390^ Photoluminescence ranging from blue (350–450 nm),^391^ to green, to infrared (500–800 nm)^392−395^ can occur from electron–hole recombination in microscopic sp^2^ graphitic regions within the heavily oxidized GO surface.^391^ Fluorescence lifetimes can vary from picoseconds to nanoseconds^386,387,391,396^ with Chen and co-workers reporting red and blue emission in the picosecond and nanosecond range, respectively.^390^

In heavily oxidized GO with large sp^3^ regions, and thus large bandgaps, two-photon absorption is possible at high excitation energies,^397^ whereas sp^2^ domains with smaller bandgaps can only absorb one photon. Using controlled oxidation and reduction, the ratio of sp^2^ to sp^3^ regions can be adjusted, which in turn can fine-tune the absorption of the material.^398,399^ In relaxation kinetics studies, RGO has approximately 90% fast lifetime components attributed to electron–phonon interactions; a mechanism similar to graphene, giving RGO a similar carrier dynamics.^400^ Alternatively, GO has a large share of slow lifetime components and lower carrier density than RGO, suggesting defect states and oxygen-related traps control the relaxation dynamics.^384,400^

The mechanism of emission within GO remains elusive and has several competing hypotheses, as summarized by Naumov and colleagues.^384^ These mechanisms are complicated by differing approaches to generating the graphene, methods of oxidation, and then, in some cases, further reduction. Some suggest location of the emission peak is determined by the relative abundance of sp^2^ graphitic regions of a particular size and their efficiency to transfer energy to regions of larger size.^391,401−404^ Differing sizes can emit directly, or additively transfer and combine to cause a larger region to emit.^384^ Others propose that emission occurs at localized states at the oxygen containing functional groups.^386,392,393,405,406^ In this model, photoluminescence is governed by HOMO/LUMO transitions at carbon atoms adjacent to carbon–oxygen functional groups.^384^ Both of these models could be occurring independently in their respective systems, in combination in others, or through artifact debris^407^ formed during the oxidation or reduction steps.

### 3.2 Mechanical Properties

#### 3.2.1 Single-Walled Carbon Nanotubes (SWCNTs)

CNTs are among the strongest materials due to the uniform sp^2^ bonds of their graphitic lattice.^452,453^ Some structural defects, including dangling bonds at the end of a nanotube, carbon vacancy spot, sp^3^ point defects, and rotated bonds may be present, which can alter material properties.^253,454,455^ However, the density of these defects can be as low as one site per four μm in pristine nanotubes.^454^ SWCNTs have an unparalleled length-to-diameter ratios exceeding 1000:1,^453^ large surface areas,^457^ and exhibit a high degree of flexibility.^458,459^ In addition, SWCNTs have an average Young’s modulus of 0.32–1.47 TPa^459^ with bending and sheer moduli of approximately 1 TPa and 1 GPa, respectively.^458^ This allows nanotubes to bend, twist, kink, and buckle, and then return to their original shape with their properties preserved. Moreover, SWCNTs have very high thermal conductivity of up to 3500 W m·K^–1^.^460^ Strong van der Waals interactions often cause SWCNTs to cluster together into aggregate bundles^235^ and require use of surface treatments or surfactants to solubilize them for biological applications.^461^

#### 3.2.2 Carbon Dots (CDs)

CDs are small, semi-spherical nanoparticles with diameters less than 10 nm and are typically composed of carbon (approximately 50–80%), oxygen, nitrogen, and hydrogen with large surface areas.^280^ CDs consist of a core that can be crystalline or amorphous and an outer shell, which can be up to a few nanometers thick^462^ that is usually functionalized in a disordered manner with polar carboxyl, hydroxyl, or amine groups.^280,463^ Most CDs are hydrophilic due to polar surface functionality, though hydrophobic versions are possible.^464,465^ Both core and shell composition are heavily synthesis-dependent.^280^

The core can be graphitic,^307^ amorphous,^307,466^ C_3_N_4_ crystalline (β-C_3_N_4_),^467,468^ C_3_N_4_ graphitic (g-C_3_N_4_),^469,470^ or aggregated.^471−474^ Graphitic cores consist of sp^2^ carbons, while the amorphous CDs have a mixture of sp^2^ and sp^3^ carbon atoms. The C_3_N_4_ cores can be accessed through high levels of nitrogen doping during synthesis.^467−469^ The g-C_3_N_4_ is layered similarly to graphite, with hexagonal alternating sp^2^ carbons and nitrogen, while β-C_3_N_4_ core consists of sp^3^ carbon and sp^2^ nitrogen atoms.^280^ Aggregated cores can be formed during synthesis, particularly with citric acid as a starting material,^299,474^ and are held together in a sphere-like shape through π-stacking, hydrogen bonding, or van der Waals interactions.^280^ Interestingly, regardless of the core structure or connectivity, most CDs exhibit similar characteristics. This has led to the hypothesis that the core is merely a surface on which to construct an active surface layer. However, some studies have noted that the core can be as important as the surface shell^475,476^ and acts as an antenna for photon absorption and electron transfer.^280^

#### 3.2.3 Carbon Nanocones (CNCs)

CNCs can be thought of as short CNTs that are gradually reduced in size at one end until they are completely enclosed. They have an average diameter of 3 nm, a length of 40 nm, and cone angel of 20°.^350^ They aggregate together into bundles with a diameter of approximately 80 nm that can be dahlia-like if made using argon or bud-like if produced with helium.^478^ As the connected rings approach the tip of the horn, pentagons (five-membered rings) may be incorporated into the hexagonal network to form a horn.^349^ As grown, CNCs are approximately 70% tubular, 15% defective at the tip, 12% graphitic, and 2.5% amorphous carbon.^479^ When held at the base and pressed at the apex, a CNC imparts a fixed amount of elastic energy per carbon.^349^ The mechanical response can invert the cone from tip to base if the number of five-membered rings in the tip is low; however, if higher, the system is rigid, and no inversion occurs.^480,481^

CNCs have characteristically large surface areas and microporosity. The pores come in two types; open (or interstitial) pores accessible from the surface and closed (or internal) pores that are inaccessible.^350^ The size of the open pores depends on temperature, while closed pores remain intact with heat treatment. Micropores have a volume of 0.11 mL g^–1^ and a large surface area of 308 m^2^ g^–1^.^482,483^ Internal pores can be accessed through oxidizing the surface of the CNC and creating large windows.^479,484−486^ These windows can sometimes be thermally reversible, with sidewall holes being harder to close than the tip hole, and holes smaller than 0.9 nm closing more easily.^487^ Microporosity can be increased with compression at high pressures.^488^ Pores opened via oxidation that create windows can increase the surface area to 1010 m^2^ g^–1^ and increase pore volume to 0.47 mL g^–1^.^479^

#### 3.2.4 Carbon Nanohoops (CNHs)

CNHs are strained systems, and the strain of the rings arises from forcing the CPP backbone to become planar. As the size of the CPP ring is constrained with decreasing *n*, computational studies have shown that the strain increases.^364,367,489^ For a [20]CPP, strain can be as low as 29 kcal mol^–1^, while [5]CPP is significantly more strained at 119 kcal mol^–1^.^360,361,490^ Interlocked macrostructures are achievable and have interesting properties but are outside the scope of this review and are discussed elsewhere.^491,492^

#### 3.2.5 Graphene, Graphene Oxide (GO), and Reduced Graphene Oxide (RGO)

Graphene is one of the strongest materials; it is stiffer than diamond but has approximately 20% more elasticity. It can sustain up to 25% in-plane tensile and elastic strains, has higher thermal conductivity than diamond, and is impermeable even to gases as small as helium.^493^ Mechanically, graphene is more flexible and stronger than its oxidized derivatives^494^ with monolayer graphene having a Young’s modulus of approximately 1.0 TPa^494^ and GO having a modulus of 0.25 ± 0.14 TPa.^495^ Oxidation decreases the in-plane Young’s modulus and fracture strength.^496^

Like most other materials discussed in this review, graphene can have a bandgap; however, it must be induced through strain or size reduction to produce GO or GNR. Theoretical calculations predict uniaxial strains >23% are needed to open a band gap^497^ and that even with moderate deformation, properties such as resistance do not change.^498^ Gauge factors of approximately 2 are typical,^498^ though higher gauge factors, up to 150, are achievable.^499,500^ With increasing strain comes the chance of deforming the nanomaterial, which have enabled the use of rippled graphene^501^ or overlapping networks of graphene to achieve larger gauge factors on the order of 200 to 300.^502−504^

Graphene and its oxidized derivatives have exceptionally high surface areas of 2630 m^2^ g^–1^ and 2418/2391 ± 1292 m^2^ g^–1^ (theoretical/experimental), respectively^505,506^. Graphite oxide can have interlayer spacing of 0.6–1.2 nm^507^ and thickness of individual GO sheets range from 1 to 1.4 nm.^508^

### 3.3 Electronic Properties

#### 3.3.1 Single-Walled Carbon Nanotubes (SWCNTs)

Geometric differences (e.g., chirality, diameter) and density of defects or the degree of crystallinity in CNTs can all impact their electronic properties.^532,533^ CNTs can be metallic, semimetallic, or semiconducting based on their roll-up vector. They are considered chiral since different roll-up vectors produce tubes of different twists that are not superimposable images of each other (Figure 7A,B). Pristine SWCNTs are semiconducting and become p-type under most application conditions^534^ with conduction band electrons delocalized over the extended π-network.^257^ Semiconducting SWCNTs have exceptional carrier mobilities (>100,000 cm^2^ V^–1^ s^–1^),^535^ current densities (4 × 10^9^ A cm^–1^),^536,537^ room temperature ballistic electron conductivity,^536,538^ high capacitance,^539^ and exciton diffusion lengths usually in the range of 100 nm.^540^ SWCNTs can hold a voltage of up to 20 V nm^–1^ before they begin to unravel.^541^ SWCNTs can also become superconductive when cooled below 20 K.^537^ Pristine SWCNTs have an electric resistivity of 10^–6^ Ω cm. Impurities and surface defects can increase the resistivity to 1–7 × 10^–4^ Ω cm;^542,543^ with aggregation and interfacial contact resistance producing a variation in measurements.^544^ CNTs form a Schottky barrier connection with their matrix, enhancing recovery time and reducing turn-on voltage.^543,545^

Due to their small diameter of approximately 1–2 nm, SWCNTs are subject to quantum confinement effects, where electrons exist in discrete energy levels^546^ and density of states that exhibit bandgaps of approximately 1 eV.^239^ Different chiralities have different bandgaps and thus distinct excitation and emission wavelengths^547^ with decreasing bandgaps as diameter increases.^452,548^ Quantum theory predicts that SWCNT excitons are composed of 4 singlet and 12 triplet states due to the spin degeneracy^549^ and intervalley Coulombic interactions between the electron and the hole.^550^ Only one singlet transition, which happens to be higher in energy than all the other singlet and triplet dark states, is optically allowed.^263,551,552^ A bright exciton can readily decay into the lower lying dark states, where the energy is typically lost as heat, and this contributes to the intrinsic low quantum yield of SWCNTs.^553^

#### 3.3.2 Carbon Dots (CDs)

CDs have excellent charge transferability, enhanced electroconductivity, and large surface areas.^554,555^ The conductivity is enhanced in functionalized surfaces.^288,556^ When doped with heteroatoms (e.g., N, P, S, B, etc.), the electronic attributes of surface functionality can be enhanced from intramolecular charge transferability,^556−558^ where charge can be readily displaced to adjacent carbons.^558,559^ Doping also provides a means of distorting electronic configurations, tuning of local densities, and for an accelerated adsorption and desorption of substrates that interact with CDs.^556−559^

Dispersing metal cations (Hg^2+^, Cu^2+^, Fe^3+^) in solution with CDs leads to quenching of fluorescence. Photoexcited CDs can transfer electrons to metal ions, which prevents radiative recombination in excitons.^560,561^ CDs can also become photoexcited electron acceptors depending on their surface structure, and have been observed to interact with organic molecules,^562^ metal complexes,^563^ and semiconductor surfaces on which they are adsorbed.^305^ The dynamics of these transfers are extremely fast (on the scale of picosecond or faster) and require ultrafast time-resolved techniques to elucidate them.^280^

Much like their photoluminescence mechanism, the electronic properties of CDs are not well-understood. A combination of several mechanisms is likely to interact in CDs. Generally, it is accepted that the aromatic chemical structure of their cores allows for easy energy transfer throughout the conjugated system. CD absorption of short UV light (230–300 nm)^273^ has been attributed to π→π* of C=C and C=N, and longer wavelength absorption (300–400 nm) to the n→π* transition of C=O.^287,326^ Inclusion of heteroatoms can alter the electronic properties of CDs by changing the bandgaps between energy levels and red-shifting the emission.^282^ One general model used to describe these properties is the core-to-surface migration of excitations. The core acts as an antenna absorbing a photon, causing spontaneous charge separation with electrons. Holes remain trapped on the surface, where radiative recombination and fluorescence emission can occur. This model has been proposed since the inception of CDs^307^ and has been recognized by many,^302,475,476,564,565^ yet fu rther experimental findings have been elusive.^280^

A contrasting model is one based on the optical charge transfer transitions. In a system where this occurs, an exciton, localized on the surface, is directly formed when a photon is absorbed.^313^ Electron transfer from the core to these surface traps occurs simultaneously, and fluorescence occurs as a consequence of inverse recombination.^280^ This model is supported by solvatochromic and time-resolved single-molecule studies, and predicts well-defined charge transfer bands in the absorption spectra with single-exponential fluorescence decay.^313^ However, most CDs have unstructured absorption spectra and multiexponential decays^312,324,332^ and likely do not adhere to this model, unless the spectra observed in those experiments are caused by a mixture of CDs each with its own properties, leading to a convoluted spectra from a polydisperse sample.

#### 3.3.3 Carbon Nanocones (CNCs)

The overall shape of CNC facilitates flow of electrons to the pentagonal sites at the tip of the horns.^566−570^ In aggregated form, electron spin resonance has shown two decoupled electronic systems attributed to the graphene-like outer sheets and interior aggregates.^571^ NMR has supported this, showing the two distinct components as being the surface of the nanohorns, which has fast spin–lattice relation and the graphitic core exhibiting a slow relaxation.^572^ As thin films, they have low turn-on field and good long-term stability, which make them ideal for field emission applications.^573^ Pristine CNCs can exhibit semiconducting properties,^574^ and their semiconductivity can be modulated by adsorption of oxygen and carbon dioxide gases.^575,576^

#### 3.3.4 Carbon Nanohoops (CNHs)

As *n* in [*n*]CCPs increases, the energy gap between the HOMO and LUMO increases as well; hence, CPP emission red-shifts as *n* decreases.^360,361^ All CPPs share a common absorbance maximum at approximately 340 nm attributed to a symmetry-forbidden HOMO to LUMO electronic transition. This common absorbance occurs through energetically similar transitions as ring size increases (e.g., HOMO to LUMO+1/LUMO+2, and HOMO–1/HOMO–2 to LUMO).^301,302^ Tretiak and co-workers have theorized that emission is dependent on the breaking of orbital symmetry in the excited state when the CNH backbone is partially planar due to the strain of the ring system.^577^ The strain present in [5]CPP and [6]CPP, unlike larger ring systems, inhibits the planarization and thus prevents breaking the symmetry.^577^

Smaller CPPs [*n* = 5–9] have low to moderate charge mobilities, while larger CPPs [*n* = 10–12] have mobilities of more than 1.^360^ Theoretical charge transport calculations of smaller and larger CPPs indicate values comparable to C_60_ fullerene^578^ with energetic disorder and reorganization energies affecting mobilities the most.^360,579^ In addition, CPPs are easily oxidized^379,580−583^ and can produce multicharged species. These species cause drastic alteration to their electronic structure as seen in [6–9]CPP^2+^ that exhibit weak NIR emission.^379^ This phenomenon has been attributed to the in-plane aromaticity formed in the oxidized CPPs.^379,580^

#### 3.3.5 Graphene, Graphene Oxide (GO), and Reduced Graphene Oxide (RGO)

Graphene has more than 100 times higher current carrier capabilities than copper, and similarly higher intrinsic carrier mobilities than silicon.^493^ When stacked, graphene is an excellent conductor in directions parallel to the graphene sheets, but it is a poor conductor perpendicularly due to the van der Waals force between layers.^584^ Charge carriers have zero rest mass and a mean free path in the millimeter range at room temperature.^493^ These properties are imparted to the material from the conjugated sp^2^ network intrinsic to the graphitic lattice. Because graphene’s properties are highly dependent on the conjugated π-network, functionalizing graphene to produce GO or RGO often diminishes these qualities. This means that, in terms of electronic properties, graphene is better than RGO, which in turn is better than GO, as RGO has some of the sp^2^ network reconstituted when it is generated from GO.

Graphene has a low electrical noise due to its crystal lattice structure. Extremely small quantities of adsorbed material can change local carrier concentrations and thus resistance.^585−588^ Schedin and co-workers demonstrated this extreme sensitivity with a gas sensor that could detect a single molecule of NO_2_.^589^ As noted in the mechanical properties section (see Section 3.2), the bandgap can be opened on graphene via mechanical strain^497^ or it can be induced through the addition of oxygen-containing adducts that could produce quantum confinement effects. The degree and type of functionalization can turn graphene into a semiconductor or even an insulator.^590−592^

## 4 Surface Functionalization Chemistry

In order for most CNMs to be used in biological applications, they often need to be modified to induce a desired application functionality. These include tuning fluorescence properties, controlling solubility in a particular matrix (e.g., wrapping with an amphiphilic polymer for aqueous dispersion), or to generate a handle on which to build, connect, and expand for further elaboration (e.g., installing a carboxylic acid for conjugation to amines via amidation). While the focus of this review is CNM fluorescent probes, many different disciplines work with the base carbon materials in a number of research areas ranging from physical material studies of hybrid composites for energy storage to *in vivo* deployment of biosensors. This section describes the chemistries that have been performed on CNMs from various disciplines with the goal of providing the reader with a survey of what is possible. Not every functionalization will be compatible with every application.

We have organized this section of the review under two broadly defined umbrella terms: non-covalent (Table 5) and covalent (Table 6) functionalizations. Covalent approaches collectively refer to methods that break and form new bonds, whereas non-covalent approaches refer to those that do not. Generally, covalent attachments are more stable, but often come at the cost of destroying the conjugated networks and associated photochemical features. Non-covalent approaches leave the sp^2^ network that imparts most of the interesting properties of the materials intact; however, they can attenuate various properties through subtle electronic changes to the local environment. Across all CNMs reviewed, 1,3 dipolar cycloadditions and oxidations are the most common covalent modifications, and π-stacking with the aryl groups is among the most common non-covalent modifications.

**Table 5:** Summary Table of Non-covalent CNM Functionalizations

Material	Functionalization	Location	Resulting Property	Ref
SWCNTs	Surfactants	Surface	- Aqueous solubility	(617, 618)
	Oligonucleotides	Surface	- Aqueous solubility	(619, 623)
			- Molecular recognition	
	Peptides	Surface	- Aqueous solubility	(620−623, 627, 630)
			- Molecular recognition	
	Proteins	Surface	- Aqueous solubility	(624, 625)
	Polymers	Surface	- Aqueous solubility	(626, 627)
	Antibodies	Surface	- Molecular recognition	(627, 628)
	Halogen-doping	Surface/Embedded	- Increases electrical conductivity	(631)
Carbon dots	Oligonucleotides	Surface	- Quenches fluorescence	(463, 632)
	Carboxylate	Surface	- Binds metals or polar molecules	(633−637)
Carbon nanohoops	Iodine	Internalized	- Electrical stimuli-responsive multifunctional material	(372)
	C_60_	Internalized	- Quenches fluorescence	(642, 643)
	Heteroatoms	Surface from synthesis	- Modifications to how rings assemble or are spaced	(149, 650−656)
Graphene/GO/RGO	Arenes	Surface	- A stable base upon which other functionalization can be built without effecting properties of the material	(658−661)
	Oligonucleotides	Surface	- Aqueous solubility	(664, 665)
			- Nanostructure self-assembly	
	Surfactants	Surface	- Solubility and phase transfer	(663)
	Porphyrins	Surface	- Aqueous solubility	(666−668)
			- Increases electron transfer	
			- Healing of defective vacancies	
	Polymers	Surface	- Self-assembly	(501, 662, 669)
			- Dispersion	
	Chitosan	Surface	- Dispersion and pH sensitization	(670)
	Metal nanoparticles	Surface	- Directs assembly	(671−691)
			- Increases electron transfer	
			- Molecular recognition	
	Quantum dots	Surface	- Increases electron transfer	(693−695)
			- Molecular recognition	

**Table 6:** Summary Table of CNM Covalent Functionalizations

Material	Functionalization	Location	Resulting Property	Ref
SWCNTs	Halogenation	Surface	- Makes SWCNTs more insulating than conducting	(696−699, 702, 706)
			- Provides reactive handle	
	Dehalogenation	Surface	- Restores conductive properties	(704, 705)
	Nucleophilic substitution	Surface	- Installs handles	(703, 704, 710, 736, 737)
			- Diminishes electronic properties	
	[2 + 1] cycloaddition	Surface	- Installs handles	(707−712)
			- Diminishes electronic properties	
			- Preserves electronic properties	
	1,3-Dipolar cycloaddition	Surface	- Installs handles	(713−715)
			- Diminishes electronic properties	
	[4 + 2] cycloaddition	Surface	- Installs handles	(716−719)
			- Diminishes electronic properties	
	Radical addition	Surface	- Installs handles	(720−722, 724, 728−731)
			- Diminishes electronic properties	
	Birch reduction alkylation	Surface	- Installs handles	(725−727)
			- Diminishes electronic properties	
	Silylation	Surface	- Installs handles	(732)
			- Diminishes electronic properties	
	Electrophilic substitution	Surface	- Installs handles	(733, 734)
			- Diminishes electronic properties	
	Ozonolysis	Surface	- Installs hydroxyls and ethers	(738−741)
			- Diminishes electronic properties	
	Oxidation	Surface	- Installs hydroxyls, epoxides, ethers, and carbonyls	(537, 704)
			- Diminishes electronic properties	
Carbon dots	Amide/carboxylic acid coupling	Surface	- Installs handles	(463, 744)
			- Molecular recognition	
	Nucleophilic substitution	Surface	- Installs handles	(745−747)
			- Quenches fluorescence	
			- Solubility in aqueous mediums	
	Silylation	Surface	- Conjugation to nanoparticles	(748, 749)
			- Molecular recognition	
	Esterification	Surface	- Passivation	(750, 751)
			- Solubility in aqueous mediums	
			- Molecular recognition	
	Sulfonation	Surface	- Installs handles	(752−754)
			- Molecular recognition	
	Heteroatom doping	Surface	- Increases/decreases electron transfer	(556−559)
		Embedded	- Tunes fluorescence	
Carbon nanohoops	*Meta* linkage	On ring	- Tunes fluorescence	(362)
	Heteroatom inclusion	On ring	- Tunes fluorescence	(88, 767, 768)
			- Solubility in aqueous mediums	
			- Increases/decreases electron transfer	
	Fluorination	On ring	- Modulates electronic properties	(652)
			- Alters redox properties and host–guest interactions	
	Extended conjugated network inclusion	On ring	- Elongation of system	(765, 770−772)
			- Asymmetric enrichment	
			- Shifts emission	
Graphene	Radical addition	Surface	- Installs handles	(773−779)
			- Creates defect sites	
			- Increases/decreases electron transfer	
	1,3-Dipolar cycloaddition	Surface	- Installs handles	(780−789)
			- Creates defect sites	
			- Increases/decreases electron transfer	
			- Enhances dispersibility	
	Halogenation	Surface	- Installs handles	(790−794)
			- Creates defect sites	
			- Thermal and chemical stability	
			- Increases interlayer distance	
			- Enhances conductivity	
	Acidic oxidation	Surface	- Installs handles	(795−797)
			- Creates defect sites/GO	
			- Diminishes electronic properties	
			- Solubility in aqueous mediums	
	Thermal oxidation	Surface	- Installs handles	(799−803)
			- Creates defect sites/GO	
			- Diminishes electronic properties	
			- Solubility in aqueous mediums	
	Ozonolysis	Surface	- Installs handles	(798)
			- Creates defect sites/GO	
			- Diminishes electronic properties	
			- Solubility in aqueous mediums	
GO/RGO	Chemical reduction	Surface	- Reduces GO to RGO	(804−819)
			- Recovers some electronic properties	
			- Modifies surface groups to reduced forms	
	Thermal reduction	Surface	- Reduces GO to RGO	(820−823)
			- Recovers some electronic properties	
			- Modifies surface groups to reduced forms	
	UV reduction	Surface	- Reduces GO to RGO	(824−827)
			- Recovers some electronic properties	
			- Modifies surface groups to reduced forms	
	Microwave reduction	Surface	- Reduces GO to RGO	(828)
			- Recovers some electronic properties	
			- Modifies surface groups to reduced forms	
	Bacterial reduction	Surface	- Reduces GO to RGO	(829)
			- Recovers some electronic properties	
			- Modifies surface groups to reduced forms	
	Heteroatom doping	Surface	- Installs handles	(830−835)
			- Creates defect sites	
			- Increases/decreases electron transfer	
	1,3-Dipolar cycloaddition	Surface	- Installs handles	(781, 836, 837)
			- Creates defect sites	
			- Increases/decreases electron transfer	
			- Solubility	
	Radical addition	Surface	- Installs handles	(778, 838−842)
			- Creates defect sites	
			- Increases/decreases electron transfer	
			- Solubility	
	[2 + 1] cycloaddition	Surface	- Installs handles	(843, 844)
			- Creates defect sites	
			- Increase/decrease electron transfer	
			- Solubility	
	[3,3] sigmatropic rearrangement	Surface	- Installs handles	(845)
			- Creates defect sites	
			- Increases/decreases electron transfer	
	Alkylation	Surface	- Installs handles	(846)
			- Creates defect sites	
			- Increases/decreases electron transfer	
	Halogenation	Surface	- Installs handles	(847−854)
			- Creates defect sites	
			- Increases/decreases electron transfer	
	Amide couplings	Surface	- Attaches linkers	(857−864, 872−875)
			- Attaches molecular recognition groups	
			- Attaches solubilizing groups	
			- Passivation for biological applications	
	Polymerization	Surface	- Attaches solubilizing groups	(889−892)
			- Increases/decreases electron transfer	
	Esterification	Surface	- Attaches linkers	(876−879)
			- Attaches solubilizing groups	
	Etherification	Surface	- Attaches linkers	(880)
			- Attaches solubilizing groups	
	Silylation	Surface	- Attaches linkers	(881−884)
			- Attaches solubilizing groups	
			- Attaches molecular recognition groups	
	Nucleophilic addition	Surface	- Attaches linkers	(878, 885)
			- Attaches solubilizing groups	

### 4.1 Non-covalent Functionalization Chemistries

#### 4.1.1 Single-Walled Carbon Nanotubes (SWCNTs)

Non-covalent surface modifications involving π-stacking are primarily employed to solubilize CNTs without disrupting their sp^2^ lattice. These include traditional surfactants (e.g., sodium dodecyl sulfate (SDS), sodium cholate (SC), Triton X-100),^617,618^ surfactant-like amphiphilic biopolymers (e.g., DNA and RNA oligonucleotides),^619^ peptides,^620−623^ and polycyclic aryl complexes with hydrophilic appendages (e.g., proteins^624,625^ and polymers^626,627^). These approaches can impart solubility alone (e.g., SC) or solubility with sensitization to various analytes of interest (e.g., single-stranded DNA (ssDNA) used to sense catecholamines).^619^ Non-covalent approaches have also been used to conjugate nanotubes to a variety of molecular recognition motifs such as antibodies,^627,628^ peptides,^629,630^ and aptamers^623^ for specific biosensing of analytes of interest. Moreover, halogen-doping of SWCNTs via halogenated solvents has been investigated recently by Taborowska and co-workers. SWCNTs treated with these solvents were noted to exhibit an increase in electrical conductivity, with bromoforms producing the most dramatic effects.^631^

#### 4.1.2 Carbon Dots (CDs)

CDs have been non-covalently modified in manners similar to CNTs. Aryl rings can π-stack with the extended π-systems of CDs and have been used to anchor a number of quenching motifs, including ssDNA.^463,632^ The ssDNA can desorb in the presence of its complementary strand, revealing the fluorescent dot. Similar unquenching phenomena are noted when certain ssDNA base pairs bind to metal ions such as Hg^2+^ or Ag^+^.^633,634^ Most CDs have polar groups on their surface that are installed during synthesis, and these groups can participate in non-covalent interactions. Carboxyl groups can be deprotonated and used to sequester metal ions such as Na^+^,^635^ bind polar molecules like Rhodamine B,^636^ or attract larger positively charged polymers such as polyethyleneimines (PEIs).^637^

#### 4.1.3 Carbon Nanocones (CNCs)

Non-covalent modification of nanocones occurs in manners similar to CNTs and CDs. Sidewalls of the CNC can have π-stacking interactions with other aromatic systems.^350^ These non-covalent interactions have been used to maintain most of the electronic properties of the CNC, while imparting handles for attachment.^352,638,639^ Non-covalent interactions have also been used to attach porphyrins^351,640^ and can solubilize CNCs in aqueous solvents.^641^

#### 4.1.4 Carbon Nanohoops (CNHs)

Carbon nanohoops are excellent candidates for host–guest type applications with other macro- or small molecules that can non-covalently interact with them. These interactions can occur both externally on the hoop or internally within the pore at the center of the structure. Most CPPs undergo quenching upon internal guest uptake.^360,378,642,643^ This quenching can be leveraged to generate a turn-on sensor when the guest molecule is stripped from the CPP. This allows for a modular approach where the sensing mechanism is reliant on a guest modification rather than modifications to host CPP, which may already be optimized to particular wavelengths and brightness levels.

[*n*]CPPs can π-stack with other π-systems, but they are unable to do so internally with themselves. One common π-interaction involves end-capping a [10]CPP with a fullerene C_60_.^644^ In a similar manner, [*n* + 5]CPP can be used to selectively encapsulate an [*n*]CPP forming a shortened version of a DWCNTs.^645,646^ Typically, in solid-state structures, [*n*]CCPs stack in a herringbone pattern except for [6]CPP, which adopts a columnar packing.^647−649^ Columnar packing is also favored when [*n*]CCPs are fluorinated,^650−652^ carboxylated,^653,654^ heteroatom-doped,^369,655^ or reduced to anionic forms.^656^ Columnar configuration was leveraged by Ozaki and co-workers to fill CPPs with I_2_ molecules and produce electrically induced white light emission.^378^

#### 4.1.5 Graphene, Graphene Oxide (GO), and Reduced Graphene Oxide (RGO)

Like many other carbon-based systems with sp^2^ networks, non-covalent functionalization of graphene, GO, and RGO relies on π-stacking interactions or other π-system interactions.^657^ Because GO and RGO both contain isles of graphene within their structures, these interactions can be generalized to all forms of graphene with differences in binding stability being dependent on the base material being functionalized.

Non-covalent attachment of aryl complexes such as pyrene,^658,659^ naphthalene,^660^ and perylene^661,662^ can be achieved through π-stacking. Using this same approach, ssDNA,^663−665^ porphyrins,^666−668^ polyaniline,^501,662^ and polystyrene^669^ have been attached. Zwitterionic and hydrogen bonding interactions can also be utilized as demonstrated by Fang and co-workers, in which RGO was functionalized with the polymer chitosan.^670^ Nanoparticle deposition has also been extensively explored as a method of non-covalent functionalization. Various groups have deposited gold,^671−676^ palladium,^677−679^ platinum,^680−684^ cobalt,^685−687^ silicon,^688^ tin,^689−691^ and various metal oxides^501,661,687,689^ on graphene and its derivatives. These nanohybrid assemblies are understood to be stabilized by van der Waals interactions between ligand-capped metal nanoparticles and graphene, or through direct electrostatic interactions between ligand-free metals and graphene.^692^ Similar interactions have been used to immobilize carbon quantum dots (CQDs) on graphitic surfaces.^693−695^

### 4.2 Covalent Functionalization Chemistries

#### 4.2.1 Single-Walled Carbon Nanotubes (SWCNTs)

Most classic organic transformations to aryl and extended aryl systems are possible, though they often occur in an uncontrolled manner over the entire nanotube. These approaches have been used to impart functional handles for further chemical modification or to introduce bright defect sites that can shift the emission spectrum and modulate quantum yield.

Halogenations of the side walls have been used to introduce fluorine^696−699^ and cause conversion of the sp^2^ metallic or semiconducting tubes into sp^3^ insulating tubes.^700^ DFT calculations suggest 1,2-addition is more favorable than 1,4-addition^701^ though both are likely to occur under the aggressive reaction conditions. Fluorine can be substituted via Grignard or organolithium reagents^702^ and can undergo substitutions from diamines^703^ and diols.^704^ Heating at high temps of 500 °C can dealkylate the nanotube, recovering its pristine properties,^705^ yet others have contested that this rarely occurs.^704^ Chlorination and bromination are also possible, with chlorine adding more readily than bromine.^706^

Cycloadditions are another family of reactions that are commonly utilized to functionalize SWCNTs. These include [2 + 1] cyclopropanations of carbenes^707−709^ and nitrenes,^710−712^ 1,3-dipolar cycloadditions of azomethine ylides to form fused pyrrolidine rings,^713^ nitrile imines under microwave conditions to form pyrazoline derived tubes,^714^ zwitterionic cycloadditions,^715^ and Diels–Alder cycloadditions with and without microwave conditions.^716−719^

Besides halogenations and cycloadditions, radical additions are another common reaction class. Functionalization of side walls can be achieved with radicals from diazonium salts^720,721^ in a reductive manner or in an oxidative manner with aromatic amines.^722−724,^ Reductive Birch reduction–alkylation has been achieved using classic conditions of alkali metals in liquid ammonia with alkyl halides or sulfides as coupling partners.^725,726^ Reduced or hydrogenated versions have been produced in a similar manner using methanol as a hydrogen source in lieu of an alkyl halide.^727^ Alkyl and aryl peroxides have been thermally decomposed and added to SWCNTs in a radical manner.^728,729^ Photoinduced radical attachments of perfluoroalkyl groups have been achieved^730,731^ as have photoinduced silations using UV irradiation.^732^

Electrophilic additions are also achievable in CNTs. Tagmatarchis and co-workers added chloroform in the presence of a Lewis acid in order to hydrolyze the groups to produce hydroxyl functionality.^733^ Balaban and co-workers explored electrophilic additions using Friedel–Crafts conditions to generate polyacrylate nanotubes.^734^ Complementary to electrophilic additions, nucleophilic additions of functionalities like carbenes,^735^ octadecylamine in an amination reaction,^736^ or carbon dioxide after treatment with *sec*-BuLi^737^ have also been achieved.

Ozonolysis has been reported at low^738,739^ and room temperatures.^740^ Subsequent treatments with peroxide, dimethyl sulfide, or sodium borohydride afford carboxylic acids and esters, ketones and aldehydes, and hydroxyls on the surface. Banerjee and co-workers noted that sidewall ozonation occurs more readily in narrow diameter tubes due to increased strain from the curvature and a higher rehybridization energy.^741^

Oxidations using concentrated nitric or sulfuric acids, peroxides, and oxygen have all been successfully employed for covalent modification of nanotubes.^537,704^ These reactions tend to form carboxyl groups at the ends of the nanotubes and at defect sites on the side walls.^742^ While these nanotubes have better aqueous solubility, they tend to lose most of their pristine optoelectronic properties because addition often proceeds in an uncontrolled manner.^537^ While many reports of covalent CNM modifications exist, a recent study by Sanders and O’Bryan surveying select covalent modifications noted issues with the reproducibility of some of the reported reactions.^743^

#### 4.2.2 Carbon Dots (CDs)

Most covalent modifications to CDs utilize oxidation handles installed on the surface during synthesis, including carbonyl, hydroxyl, or amine functionalities. Most popular are amide coupling reactions including EDC/NHS, which couple carboxylic acids with amines and have been reviewed extensively elsewhere.^463^ This has been used to directly attach end-product functionality (e.g., coupling with an amine aptamer)^744^ or for attaching a new pendant functionality (e.g., ethylenediamine)^744^ that can be further elaborated upon. Besides amide couplings, a variety of other reactions have been demonstrated including nucleophilic acyl substitutions,^745−747^ silylations,^748,749^ esterification,^750,751^ sulfonation,^746,752−754^ and copolymerization via S_N_2 of alcohols that open epoxides.^602^ Most classic organic transformations of these pendant oxidized groups formed during the synthesis are achievable.

Surface passivation or derivatization of CDs can tune fluorescence properties and quantum yield. This can be done both through post-synthesis modifications or by introducing passivating agents during synthesis that are incorporated into the CDs.^463^ While these groups do impart new functionality that could in turn change the optoelectronics of the materials, they also impart colloidal stability to CDs allowing them to be dispersed in solutions rather than clumping as aggregates.

Different types of doping, particularly with heteroatoms, can lead to enhancement of adjacent functional sites through charge transference.^556−558^ Heteroatom doping can also distort electronic states by effectively tuning local charge densities, and can increase the adsorption and desorption of molecules.^556,557,559^ These dopants are usually added during the synthesis or as a covalent appendage post synthesis.

#### 4.2.3 Carbon Nanocones (CNCs)

Various methods of oxidation are commonly used to functionalize CNCs. These methods rupture the sp^2^ network and open up holes on the surface through insertion of hydroxyl or carbonyl functionalities. Some common approaches include high temperature treatment in the presence of O_2_^479^ or CO_2_,^755^ or through treatment with strong acids such as H_2_SO_4_, H_2_SO_4_/H_2_O_2_,^756^ or HNO_3_^486^ under heat, or through microwave irradiation.^757^ The acid treatment increases porosity by opening holes on the surface and increases internal porosity.^350^ Introduction of these groups, particularity carboxylic acids, serves as a powerful functional handle that can be easily transformed into many chemical moieties for couplings, conjugations, or direct linkages. A modified oxidative procedure using O_2_ at high temperatures and lower pressures has also been developed to selectively install carboxylic acid units at the conical-tips of the CNCs.^351^ If less oxygen functional groups are desired, a second heat treatment with H_2_ can be performed.^757^ Direct amination of CNCs using NaNH_2_ and liquid ammonia is also possible. This has produced amino-nanocones that are water-soluble without the introduction of any additional holes to the structure. Amino-nanocones are also able to be separated according to their size.^758^

Besides direct oxidation of the CNCs, 1,3-dipolar cycloadditions have proved to be very useful as well. Azomethine ylides can be generated *in situ* from a decarboxylative condensation of α-amino acids with aldehydes to yield the installation of almost any functionality desired.^759^ One of the most popular options involves N-modified α-amino acids to produce N-substituted pyrrolidines on the CNH scaffold.^760,761^ Malonate moieties can also be introduced to the surface using a Bingel cyclopropanation reaction. These include simple ones such as diethyl malonate and larger custom synthesized ones containing large anthracene, pyrene, or light-harvesting groups.^762^ Utilizing microwave-assisted irradiation allowed for solventless introduction of the malonates,^762^ as well as [2 + 1] nitrene^763^ and benzyne cycloadditions.^612^

#### 4.2.4 Carbon Nanohoops (CNHs)

Various functionalizations can be covalently introduced at different positions during the synthesis of CPPs.^764−766^ One common approach to enhance brightness is to connect a ring within an [*n*]CCP in a *meta* or 1,3 manner rather than a *para* or 1,4 connection.^362^ Installing electron withdrawing groups, such as nitrogen or benzothiadiazole, causes emission to red-shift due to a narrowing of the HOMO–LUMO gap.^370,767,768^ Incorporation of fluorine into the nanohoop, in place of a C–H bond, can impact the way nanohoops align with one another in the solid state, causing formation of tubular nanotube-like channels.^769^ Other nanohoop versions have also been achieved through incorporation of a naphthalene ring asymmetrically to form a chiral nanohoop,^765,770^ symmetrically to create extended π-networks,^771^ or through the incorporation of other fused aryl and π-systems to create extended π-networks.^766,772^ Furthermore, addition of sulfonates on an extended ether side at one of the C–H positions on an aryl ring has afforded a more water-soluble version of nanohoops.^377^ This attachment was made through a benzylic alcohol and has proved very useful as demonstrated by White and co-workers, in which the transformation was exploited for an azide click chemistry reaction for connecting the azide-CPP with alkyne-folic acid for *in vitro* studies.^377^

#### 4.2.5 Graphene, Graphene Oxide (GO), and Reduced Graphene Oxide (RGO)

Covalent functionalization of graphene shares many commonalities with GO and RGO since both contain graphene isles. These reactions focus on the sp^2^ C=C bonds present in graphene, GO, and RGO, but also include the more reactive oxygen functionalities in GO and RGO.

Additions of free radicals through diazonium salts^773−778^ and benzoyl peroxides^779^ have been achieved, as have 1,3-dipolar cycloadditions of azomethine ylides,^780−782^ nitrenes,^783−787^ and arynes.^788,789^ Halogenation is also possible through fluorination,^790^ chlorination,^791,792^ or bromination.^793,794^ In addition, covalent functionalization of graphene to GO can be achieved through strong oxidation by acids,^795−797^ ozone,^797,798^ and chemical or thermal exfoliation from graphite oxide.^799−803^ In all these methods, the sp^2^ network is oxidized and new functionalities, including hydroxyl, carbonyl, carboxylate, and epoxide, are formed on the surface. RGO can be generated from GO through an additional reduction step after the oxidation. This is done to restore some sp^2^ functionality and associated properties, such as electrical conductivity and absorption properties, and to create a lightly oxidized version as compared to GO. Common chemical approaches reduce GO with hydrazine,^799,804,805^ but the scalability of this approach is limited. To overcome this, methods using ascorbic acid,^806,807^ sodium borohydride,^808−811^ ethanol,^812−814^ H_2_,^815,816^ SO_2_,^817^ and hydroquinone^818,819^ have been developed. Besides chemical approaches, thermal reduction in the presence of inert gas is also possible,^820−823^ as is ultraviolet light reduction.^824−827^ Microwave-assisted versions of chemical and thermal reductions can also be employed.^828^ Moreover, Salas and co-workers have developed a procedure to produce RGO from GO using bacteria.^829^ Doping graphene with nitrogen is also possible through incorporation of nitrogen precursors during the reduction or annealing steps in various processes^830−833^ using ammonia^831^ or hydrazine.^681^ These dopants, and boron,^834^ can also be incorporated directly upon synthesis.^835^

GO and RGO can be modified with 1,3-dipolar cycloadditions,^781,836,837^ diazonium radical additions,^778,838−842^ carbene^843^ and nitrene^844^ additions, [3,3] sigmatropic rearrangements,^845^ alkylations,^846^ and halogenations,^847−854^ among other chemical inclusions.^657,855,856^ Additional covalent modifications can occur on the newly installed oxygen-containing groups. Utilizing the carboxylic acids, many groups have performed amide couplings with amines on small molecules,^857−860^ biomolecules,^861^ polymers^862−864^ and other compounds of interest.^857,865−871^ This approach has also been used to help further solubilize and passivate through attachment of polyethylene glycol (PEG)-amines^862,872,873^ or poly-l-lysine.^874,875^ In a similar manner, esters,^845,876−879^ ethers,^880^ and silanes^881−884^ have been attached as well.

Nucleophilic additions using epoxides can occur as demonstrated by Yu^878^ and Hsiao et al.^885^ Generation of carbamates can also occur via isocyanates reacting with both the carboxyl and hydroxyl groups on the oxidized graphene.^886−888^ Radical polymerization, grown directly from the GO surface, is also achievable as demonstrated by several groups,^889−892^ where they used a living radical polymerization after covalently attaching an initiator followed by addition of various monomers.

## 5 CNM Fluorescent Probe Applications

**Figure 11:**
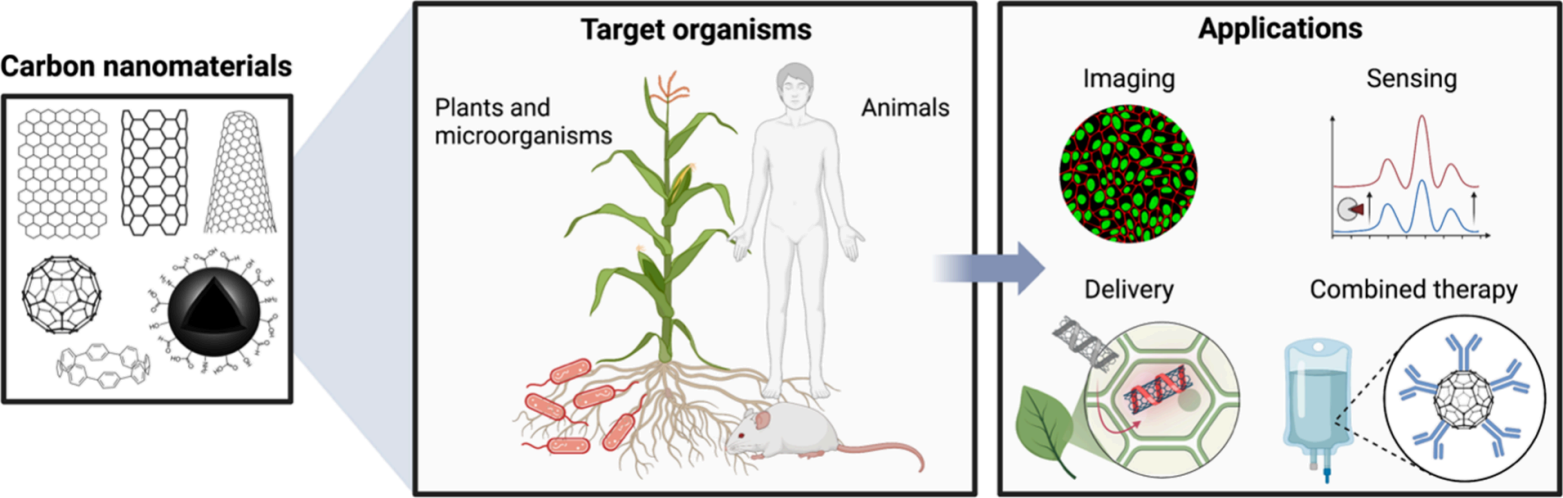
Various carbon nanomaterials have been used in microorganisms, plants, and animals for diverse applications in imaging, biosensing, biomacromolecule and drug delivery, and combined therapy. Figure prepared using BioRender.com.

In this section, we describe the use of CNMs in cells, tissues, microorganisms, plants, animals, and the environment for various applications in imaging, sensing, delivery, and therapeutics in detail (Figure 11).

### 5.1 Fluorescence Imaging in Biomedical Applications

In this section, we will discuss the use of fluorescent CNMs for imaging in biological applications, including *in vivo* imaging of the vascular system and organs in small animals, and *in vitro* imaging in cells and tissues.

#### 5.1.1 *In Vivo* Vasculature Imaging

One prominent application of fluorescent CNMs is the vasculature imaging. All biological entities rely heavily on a steady supply of nutrients. Animals utilize the blood circulatory system to transport oxygen, carbon dioxide, nutrients, heat, and hormones into and out of organs. Blood vessels are dynamic in nature and capable of undergoing changes to facilitate structural remodeling. To accommodate temporary physiological adaptations, such as during pregnancy^893^ or endurance training,^894^ transient vasculature restructuring processes can occur. Moreover, at slower temporal scales, permanent changes can occur in association with several pathological conditions. From a pathological perspective, early diagnosis of alterations in vasculature is crucial for medical interventions, especially in the cases of hypertension, atherosclerosis, diabetes, and retinopathy. Hence, vascular imaging plays an important role during diagnosis, and for monitoring treatment efficacy and disease progression. The heart, brain, and eyes are among some of the organs that are heavily monitored for changes in vasculature.

##### 5.1.1.1 Heart Vasculature

Mapping blood vessels in the heart dates back to 1950s with the introduction of angiography using iodine injected into the bloodstream and visualization with X-ray. Decades later with the inventions of multiple technologies such as ultrasound, computed tomography (CT), magnetic resonance imaging (MRI), positron emission tomography (PET)/scintigraphy, ultrasonography, and optical coherence tomography (OCT), the distribution and anomalies in blood vessels can be mapped noninvasively, and most importantly often without the need of exposure to ionizing X-ray radiation. While these techniques can report on global changes in vasculature, small changes inside blood vessels cannot be mapped due to insufficient temporal and spatial resolutions. In cases where medical interventions are required, invasive intravascular imaging is performed. In contrast, fluorescence-based techniques can provide high temporal and spatial resolutions compared to the conventional vasculature imaging techniques employed in the clinic.^895^ Fluorescence imaging can be performed over a wide range of excitation and emission wavelengths, and the NIR window has been particularly identified as the bioimaging window that optimizes photon absorption, reduces scattering of photons, and contributes negligible autofluorescence background. All these features allow deeper tissue penetration and imaging.

**Figure 12:**
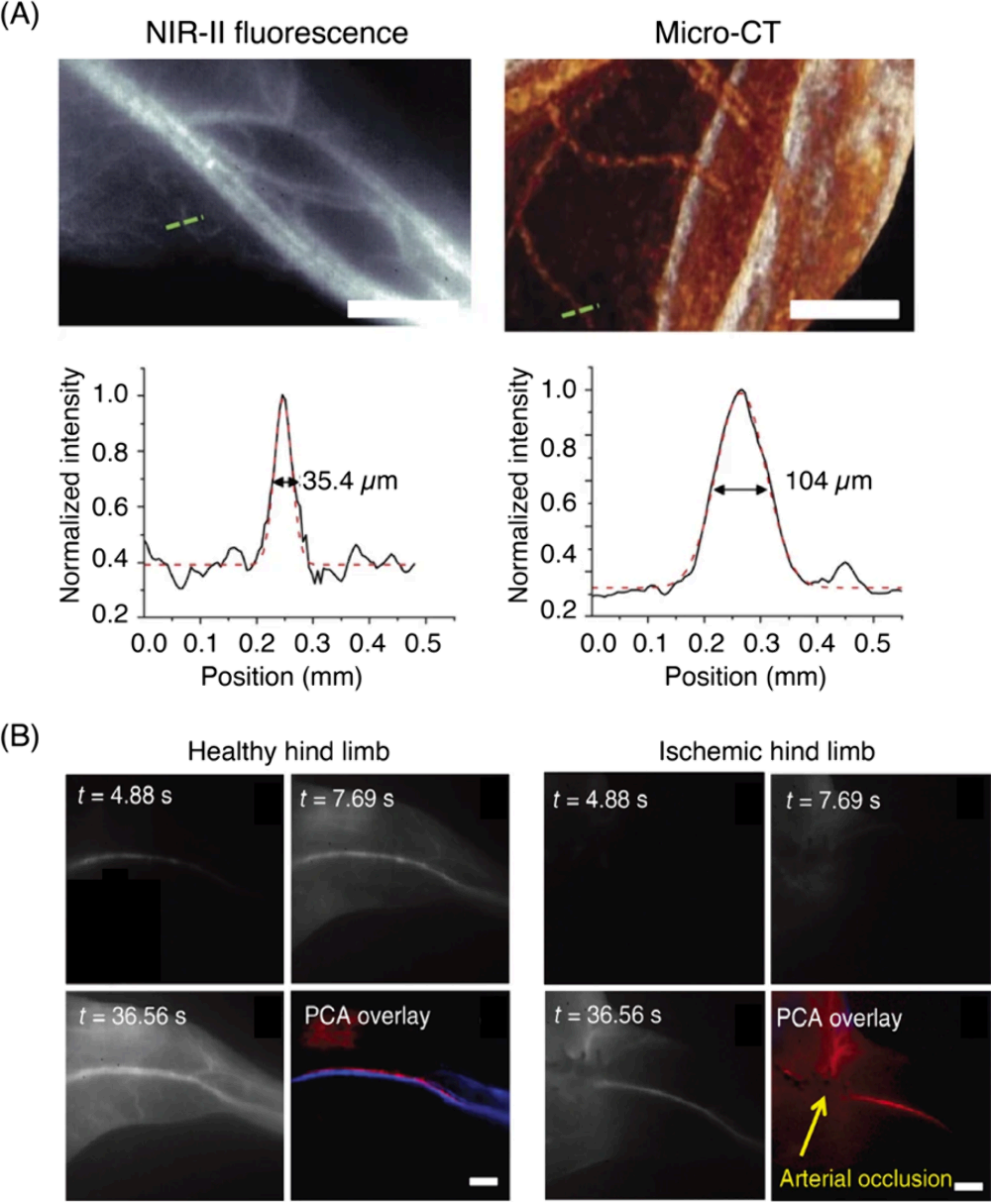
(A) NIR-II SWCNT fluorescence and micro-CT images of a mouse thigh (same area imaged in both modalities) and the cross-sectional intensity profiles measured along the green dashed lines fitted with a Gaussian distribution function (scale bar = 2 mm). (B) Time course NIR-II fluorescence images of a hind limb blood flow in a healthy vs ischemic mouse. Principal component analysis (PCA) revealed arteries and veins, color-coded in red and blue, respectively (scale bar = 2 mm). Reproduced with permission from ref (901). Copyright 2012 Springer Nature.

Among the many NIR-emissive probes that include small molecule fluorescent dyes,^897^ polymer nanoparticles,^898^ aggregation-induced emission dots, rare-earth doped nanoparticles,^899^ and semiconducting quantum dots,^900^ we will highlight carbon allotropes used for heart vasculature imaging. SWCNTs are one of the few materials whose intrinsic optical properties lie in the optimal bioimaging window (850–1350 nm). SWCNTs can be formulated to become water-soluble, and Hong et al. have studied heart vasculature using their intrinsic NIR fluorescence.^901^ A direct comparison of NIR fluorescence and commonly used microcomputed tomography (Micro-CT) revealed that the two techniques are comparable at measuring blood vessel of widths greater than 100 μm (Figure 12A). For SWCNTs, the smallest measurable vessel diameter was ∼35 μm, while Micro-CT could not discern any structure less than 100 μm (Figure 12A). Apart from measuring blood vessels diameter, gaining insights into hemodynamics (e.g., arteries vs veins) is important to assess function. Typically, Doppler measurements aided by microultrasonography provide hemodynamic information, but spatial resolution significantly attenuates at increased penetration depths. When SWCNTs were employed, based on the time difference between the inflow and outflow from veins and arteries respectively, the two types of blood vessels were successfully distinguished. To show the utility of this technology, SWCNTs were tested in mice that underwent surgically induced ischemia. As expected, a significant delay in the appearance of NIR fluorescence in ischemic limbs was noted compared to the control, suggesting that CNM fluorescence-based vasculature imaging can discern acute changes in blood flow following a pathologic condition (Figure 12B).

##### 5.1.1.2 Brain Vasculature

The brain is among the most complex organs due to its anatomical composition and function, and the demand for blood flow within cerebrovasculature is very high. By monitoring blood flow, neuroscientists can study brain regions that may be responsible for orchestrating specific actions. From a clinical perspective, fine changes in cerebrovasculature, such as vessel blockages, inflammation (vasculitis), narrowing (stenosis), vessel spasm (vasospasm), and malformations are linked to several pathological conditions. Therefore, cerebrovascular imaging-guided diagnosis could be the key for the prevention and early intervention of brain diseases. Rapid advancements in instrumentation and tools have enabled a wide variety of imaging techniques to peer into brain vasculature. Selection of an appropriate technique depends on the clinical situation and the resolution (both spatial and temporal) required for optimal diagnosis.

The gold standard for cerebrovascular imaging is angiography, where a contrast dye is introduced via a catheter placed near the arteries of the neck and the head. The contrast agent helps visualize the blood vessels in X-ray images but with limited spatial (submillimeter) and temporal (minutes long scanning times) resolutions. Fluorescence-based brain imaging offers an alternative with improved resolutions (both spatial and temporal) without the need of exposure to ionizing radiation. One of the biggest drawbacks of fluorescence imaging performed in the visible (400–700 nm) and NIR (700–900 nm) regions is reliance on craniotomy, cranial windows, and skull thinning agents. Even with invasive surgical installation of cranial windows, penetration depths of imaging are usually limited to 1–2 mm. Multiple reports from different laboratories have shown biological imaging in the NIR-II window (1000–1700 nm) can potentially enable deep brain fluorescence imaging due to greater penetration depths and reduced scattering of photons. However, there are very few materials that fluoresce in the NIR-II window and the challenge is further compounded by strict constraints including water solubility and high biocompatibility. For example, NIR-II emissive inorganic semiconducting QDs are attractive candidates due to narrow emissions and high quantum yields.^902^ However, the toxicity of heavy metal QDs is a major concern and hinders their deployment in biological applications.

**Figure 13:**
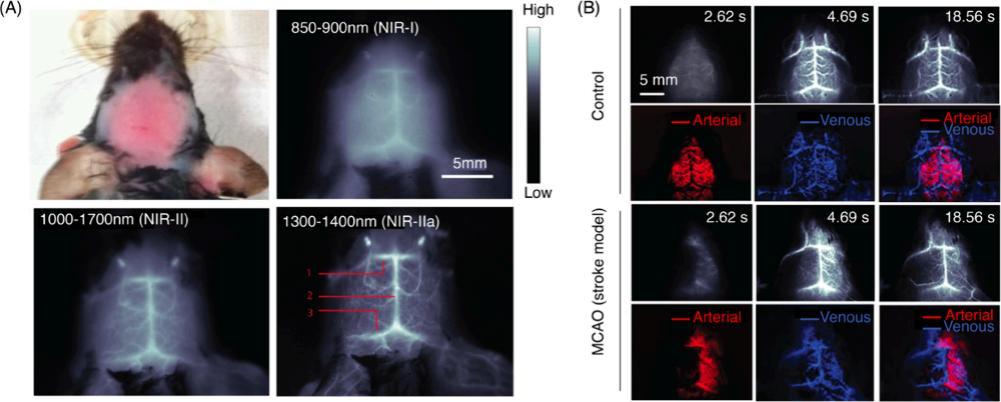
(A) Images of a head-shaved mouse and fluorescence images of the same mouse in the NIR-I, NIR-II, and NIR-IIa windows after a tail vein injection of SWCNTs. Inferior cerebral vein, superior sagittal sinus, and transverse sinus are labeled as 1, 2, and 3. (B) Time course NIR-IIa images (top rows) of a control (healthy) vs MCAO (stroke model) mouse treated with SWCNTs. PCA overlaid images (bottom rows) showing arterial (red) and venous (blue) vessels. Adapted with permission from ref (904). Copyright 2014 Springer Nature.

Highly extended π-conjugated systems, such as SWCNTs, have intrinsic emissions in the NIR-II range (900–1400 nm) when excited with NIR-I (700–900 nm) lasers. Despite low quantum efficiencies, the ability for tunable water solubility and biocompatibility sparked an interest in utilizing SWCNTs for cerebrovascular imaging. Over the years, the Dai Lab has pioneered tissue imaging in the NIR-II window using CNMs.^903^ In a report published in 2014, the group demonstrated that SWCNTs can be useful for studying brain vasculature with high spatial resolutions and great tissue depths (>2 mm in the mouse brain).^904^ Notably, this feat was achieved without the need of a surgically installed optical window in the cranium (Figure 13). Additionally, to directly compare the differences between imaging in NIR-I vs NIR-II regions of the spectrum, the authors synthesized SWCNT-IRDye800 conjugates, where IRDye800 emits in the 800–900 nm window (NIR-I) and SWCNTs emit in the 900–1400 nm window (NIR-II). After injecting a solution of SWCNT-IRDye800 through the tail of a mouse, the cerebrovasculature was imaged under 808 nm laser illumination. In comparison to NIR-I spectral window imaged by IRDye800, higher resolution images of brain blood vessels were obtained with NIR-IIa (1300–1400 nm) window using SWCNTs (Figure 13A). At the NIR-IIa window, dynamic changes in blood perfusions were recorded at sub-10 μm spatial resolutions and imaging rates of ∼5 frames/sec. Furthermore, the authors demonstrated the utility of SWCNTs to record dynamics of blood perfusions for studying the effect of strokes. Compared to WT controls, blood flow of a surgically induced middle cerebral artery occlusion (MCAO) mouse was noted to be markedly slower, thus demonstrating that CNM-based vasculature imaging can report on acute changes in hemodynamics induced by physiological perturbations (Figure 13B).

##### 5.1.1.3 Ocular Vasculature

Eye vasculature is a complex network and can be divided into three main subdivisions: hyaloid, choroid, and retinal vasculature. Generally, hyaloid vasculature system is dominant during the early development stages and disappears as retinal vessels develop and mature.^905^ The choroid is the central network of blood vessels found in the eye, which transports oxygen and nutrients into the retina via retinal pigment epithelium. The retinal vasculature is among the most studied bed of blood vessels in the body due to ease of accessibility from the front of the eye. With ever advancing non-invasive imaging modalities, patients routinely undergo retinal scans in the clinic for early detection and monitoring of disease progression, and evaluation of therapeutic efficacies for various ophthalmic diseases.^906^ For most clinical examinations, ocular vasculature is imaged by angiography, which involves an intravenous injection of a fluorescent dye, followed by imaging of retinal blood vessels via dilated eyes. Typically, fluorescein is used as the dye of choice to image retinal blood vessels with blue light excitation. As a proof-of-concept nitrogen and selenium-doped CDs have been successfully used to image retinal vasculature in mice.^907^ The spatial resolutions achieved by these CDs lag those of small molecule organic fluorophores; however, CDs can be simultaneously employed for imaging and as delivery vehicles for therapeutic agents, and are, therefore, worthy of continuous exploration in this space.^908,909^ Application of CNMs for imaging of the choroid vasculature is even less common, and to our knowledge has not yet been reported.

#### 5.1.2 *In Vivo* Whole Organ Imaging

*In vivo* fluorescence imaging presents a unique set of challenges compared to imaging in reduced preparations such as cells and tissue slices. One of the biggest challenges is the poor penetration depth, which is more pronounced when visible light is used for optical imaging. Furthermore, tissue autofluorescence is prominent in visible range of the spectrum but is considerably diminished in NIR. All of these reasons have motivated the development of fluorophores that are excitable in the NIR-I window (700–900 nm) and emit in NIR-II window (900–1400 nm). CNMs are relatively easy to synthesize and purify compared to multistep organic synthesis required for synthesizing small molecule dyes. CDs, GOs, and SWCNTs are the most widely used fluorescent CNMs for *in vivo* biological imaging, and most of the discussions in this section will focus on these materials.

**Figure 14:**
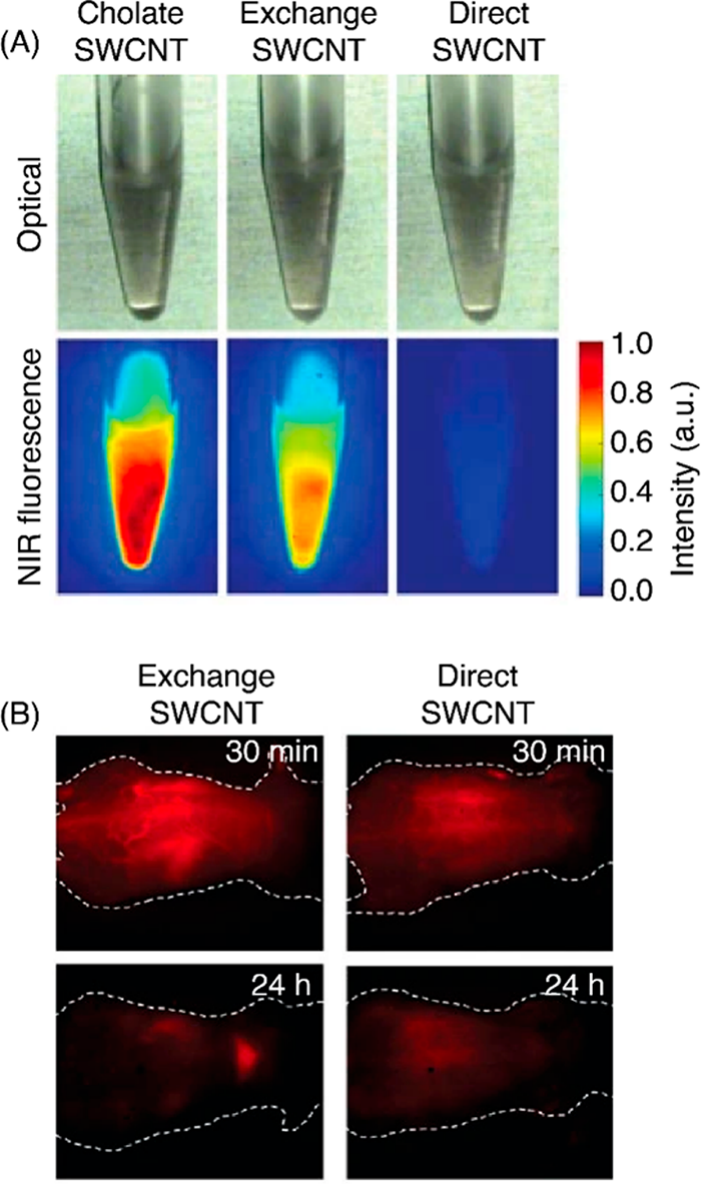
(A) Optical micrographs and NIR fluorescence images of three SWCNT preparations at equal concentrations. Emission was collected using excitation at 808 nm. (B) NIR fluorescence images (1000–1700 nm) of nude mice treated with exchange or direct-SWCNTs at 30 min and 24 h post tail vein injections. Reproduced with permission from ref (910). Copyright 2009 Springer Nature.

In order to access SWCNT fluorescence, bundled SWCNTs need to be singly exfoliated into colloidal dispersions by the use of surfactants or a wide variety of amphiphilic chemical motifs. Despite affording colloidally stable bright suspensions, surfactants used for exfoliating nanotubes are often cytotoxic to biological tissues. On the other hand, biocompatible SWCNTs dispersions, such as those obtained from the commonly used phospholipid-polyethylene glycol (PS-PEG), exhibit low brightness.^910^ To circumvent this issue, Welsher et al. devised a method to generate both bright and biocompatible SWCNTs using a two-step ligand exchange method. Bright SWCNT suspensions were first suspended with sodium cholate (SC), and the SC is subsequently replaced with PS-PEG via ligand exchange. It is thought that the ligand exchange process preserves the bright fluorescence properties afforded by SC, whereas direct passivation with PS-PEG leads to breaks in the sp^2^ lattice and generation of oxygen-induced adducts that quench the fluorescence (Figure 14A).^910^ When PS-PEG-SWCNT suspension was introduced into wild-type (WT) mice via tail vein injections, dispersions produced by the ligand exchange method showed high image contrast at very low concentrations. To generate comparable contrast in captured images, direct-sonication PS-PEG-SWCNT suspensions required more than 15-fold loading compared to exchanged PS-PEG-SWCNTs (Figure 14B).

**Figure 15:**
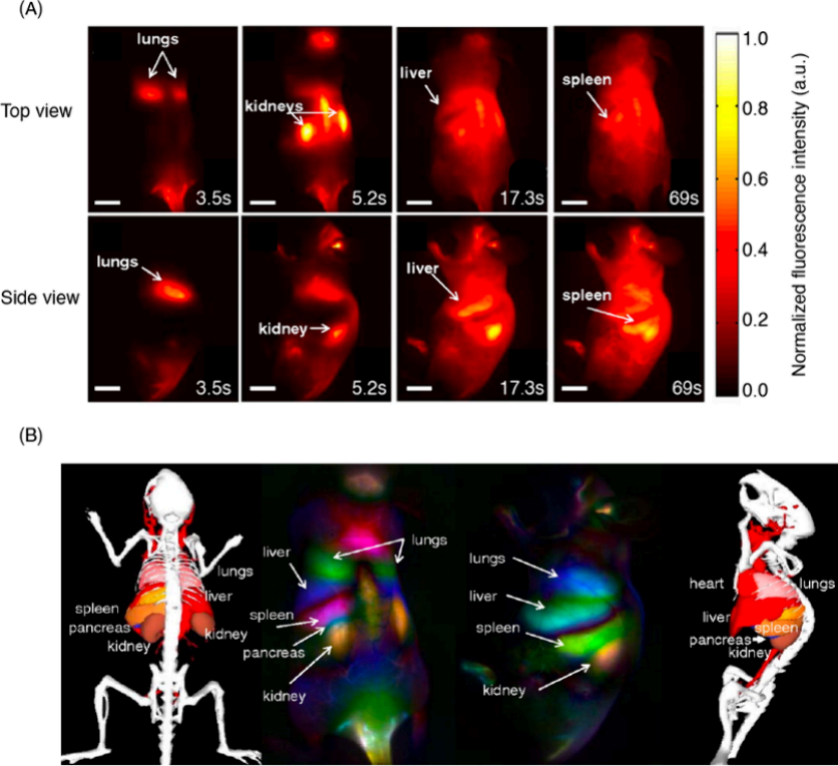
(A) Frames of video-rate imaging of a mouse following a tail vein injection with SWCNTs (scale bar = 1 cm). (B) Dynamic contrast-enhancing imaging via PCA analysis. Adapted with permission from ref (911). Copyright 2011 Proceedings of the National Academy of Sciences.

Further demonstration of SWCNTs as contrast reagents involves dynamic imaging of SWCNTs through the path of the blood circulatory system after a mouse tail vein injection.^911^ Following the injection, oxygen-deficient venous blood travels to the heart and lungs. Video rate imaging then revealed an initial spike of NIR fluorescence in the lungs, followed by a decrease in the fluorescence signal from lungs and an increase in the signal in the kidneys and the liver (Figure 15A). The temporal dynamics of SWCNT fluorescence affords imaging at anatomical resolutions through principal component analysis (PCA) of the time-variant data, which can be challenging to discern from real-time raw fluorescence images alone (Figure 15B).

**Figure 16:**
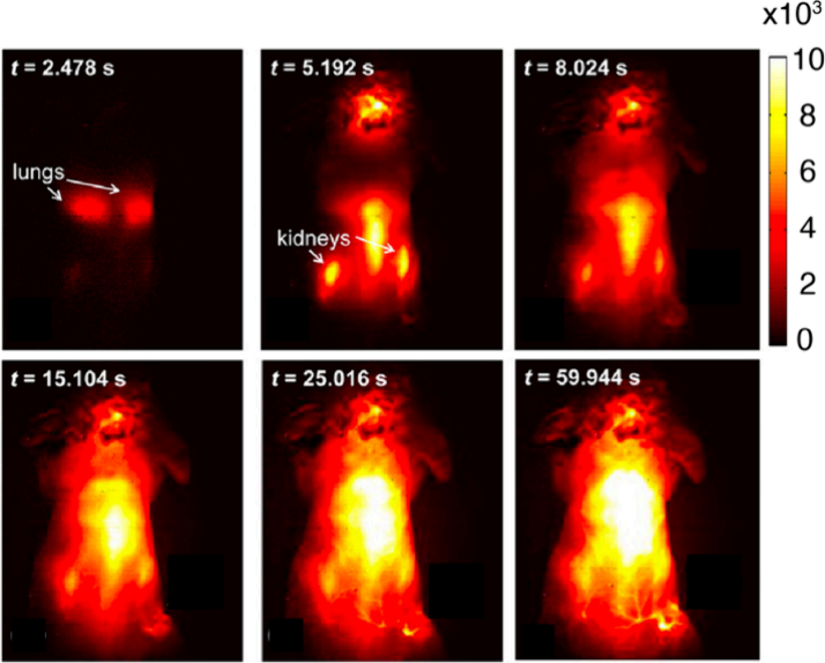
Time course NIR-II fluorescence images of a 4T1 tumor bearing mouse after injection of SWCNTs decorated with octadecene appended PEG chains. Reproduced from ref (924). Copyright 2012 American Chemical Society.

The use of functionalized SWCNTs for *in vivo* imaging extends beyond their use as whole organism-scale contrast agents to organ-specific or tumor-targeted imaging reagents.^912−915^ Tumor-homing SWCNTs, typically designed through side-wall functionalization with tumor specific antibodies or peptides, have been employed for imaging tumors in animal models. One approach employs the RGD peptide, a potent ligand for α_v_β_3_ receptor that is important for tumor angiogenesis. SWCNTs bearing the RGD ligand localize to the tumor, thus enabling imaging of the tumor and associated vasculature.^916^ A similar approach employed bifunctional SWCNTs that are decorated with tumor-specific antibodies and radioligands.^917^ The small diameters of SWCNTs (typically in the range of 1–3 nm) are thought to assist in the trafficking into tumors sites.^918,919^ Moreover, in contrast to the tight endothelial junctions found in healthy tissue, tumor sites have leaky endothelial junctions. Porous openings in vasculature associated with tumor sites facilitate nanoparticle circulating in blood to passively accumulate in tumor tissues. Once nanoparticles are accumulated, most of them are retained due to poor lymphatic drainage.^920^ This phenomenon is referred to as the enhanced permeation and retention (EPR) effect.^918^ Taking advantage of the EPR effect, the Dai lab first tested the efficacy of SWCNT accumulation in tumor-bearing mice in a proof-of-concept study.^921^ The results of the study were further confirmed by employing other imaging modalities, such as PET and Raman spectroscopy, to demonstrate the tumor targeting tendency of SWCNTs .^922,923^ Following an intravenous injection, SWCNTs accumulated predominantly in the liver, spleen, and tumor-bearing tissues. Other organs such as heart, kidney, pancreas, and lungs contained negligible amounts of SWCNTs. Although the results of this study are encouraging, a high degree of tumor targeting is generally required for early detection of tumors. Interestingly, the same group observed that functionalizing the nanotube surface with octadecene units appended to PEG chains increased blood circulation times (half-life of 30 h).^924^ The improved bioavailability in the bloodstream over an extended period, combined with continuous accumulation of SWCNTs, allowed NIR fluorescence to steadily increase in the tumor region (Figure 16).

The tumor-homing ability of SWCNTs can be leveraged not only for imaging, but also for delivery of therapeutic interventions. An interesting study used SWCNTs to image intact tissues at lower excitation powers and map the tumor region.^924^ Once the tumor map is registered, laser irradiations at high power cause SWCNTs to heat until the impacted tissue reaches the temperature necessary for thermal ablation. Such efforts can be further optimized by sorting as-synthesized SWCNT suspensions to isolate highly fluorescent chiral species. More brightly fluorescent SWCNTs afford clear tumor imaging and quickly reach tumor ablation temperatures at much lower injection doses.^925,926^ Moreover, SWCNTs can also be used to image biological tissues that are not amenable to conventional techniques. One such example is the detection of brown fat using a SWCNT reporter that is decorated with a synthetic amphiphilic polymer.^927^ Brown fat is an increasingly attractive therapeutic target and imaging it relies on expensive modalities, including PET-computed tomography.

Functionalized graphene-oxide (GO)-based nanomaterials have also been used for imaging tumor in mice *in vivo*. In one study, graphene quantum dots (GQDs) were decorated with catechol-functionalized hyaluronic acid (HA), an important biopolymer that is a component of the extracellular matrix.^928^ The catechol is understood to anchor HA through its affinity for the surface of the graphitic nanomaterials. In *in vitro* cellular assays, the authors showed that GQDs that were not decorated with HA exhibited low levels of internalization into cancerous and noncancerous cell lines. In contrast, HA-GQDs showed significant levels of uptake by A549 cancer cell lines. Remarkably, when A549-cancer cell bearing mice were injected with the nanomaterials, the HA-functionalized GQDs were more intensely localized to the tumor regions, largely recapitulating the observations made in the *in vitro* cellular assay. This study additionally demonstrated that the tumor-homing ability of HA-GQDs can be leveraged for delivering chemotherapeutic agents into tumor locations. The authors suggest endocytosis and EPR as mechanisms for cellular uptake and tumor-homing ability, with the HA motif clearly playing a facilitative role in targeting and internalization into cancerous cells.^929,930^ Other studies have used antibodies to target GO nanoparticles into cancer cells. Sun et al. showed that GOs functionalized with a lymphoma targeting antibody, Rituxan, facilitated trafficking of the nanomaterials into tumor cells *in vitro*, with the nanomaterials serving a dual purpose as imaging reagents and drug delivery vehicles.^931^

Indeed, the use of the GO-family of CNMs as dual imaging and therapeutic reagents appears to be an attribute that has been repeatedly exploited in the literature. Besides being used for drug cargo delivery, GO-based nanomaterials have also been used as agents for photothermal therapy. Yang et al. showed that the strong optical absorption of PEG-functionalized GO in the NIR can be used for efficient tumor destruction in mice models.^932^ In addition to their tumor-homing abilities, GO-based CNMs have been used for fluorescence imaging using multiphoton excitation. PEG-GO nanoparticles exhibited enhanced solubility and biocompatibility, and their two- and three-photon photoluminescence properties were exploited for imaging in cortical layers at depths of up to 300 μm^933^ and at even deeper depths in tissue phantoms.^934^

The utility of CNMs as dual imaging and therapeutic reagents extends from the GO family of nanoparticles to CDs. In one study, Ge et al. used polythiophene phenylpropionic acid as the starting precursor for CD synthesis.^935^ The resulting CD nanoparticles exhibited a broad emission with a maximum at 640 nm. When intravenously injected into tumor-bearing mice, majority of the red-emitting CDs were localized to tumor sites and the liver. The authors further demonstrated the efficacy of CDs as a treatment option for cancerous tissue via photothermal therapy.

The studies highlighted in the previous sections were conducted using mice as the model organism, consistent with the practice in many disciplines of biomedical research. Beyond mice, the use of CNMs for *in vivo* imaging has been demonstrated in drosophila (fruit flies), an important organism that is used as a model for scientific research in several disciplines of biology. In one study, SWCNTs were dispersed in buffered bovine serum albumin, concentrated, and then mixed with Baker’s yeast, which is a standard laboratory food for drosophila.^936^ Drosophila larvae fed on SWCNT–yeast paste during their normal growth phase, and then imaged in the pupal or adult phase, did not exhibit abnormal development, and had levels of survival that were comparable with those that were fed standard food. Importantly, post hoc imaging of the NIR/SWIR SWCNT photoluminescence showed that SWCNTs localized to the gut and the dorsal vessel of fruit flies. Furthermore, fluorescence signals were detected in brain tissue albeit at lower levels of intensity. While food-based nanotube delivery into model organisms could present a less invasive method of *in vivo* loading of tissue, it is not clear what new biological insights were gained from this study beyond a proof-of-concept level demonstration.^936^

**Figure 17:**
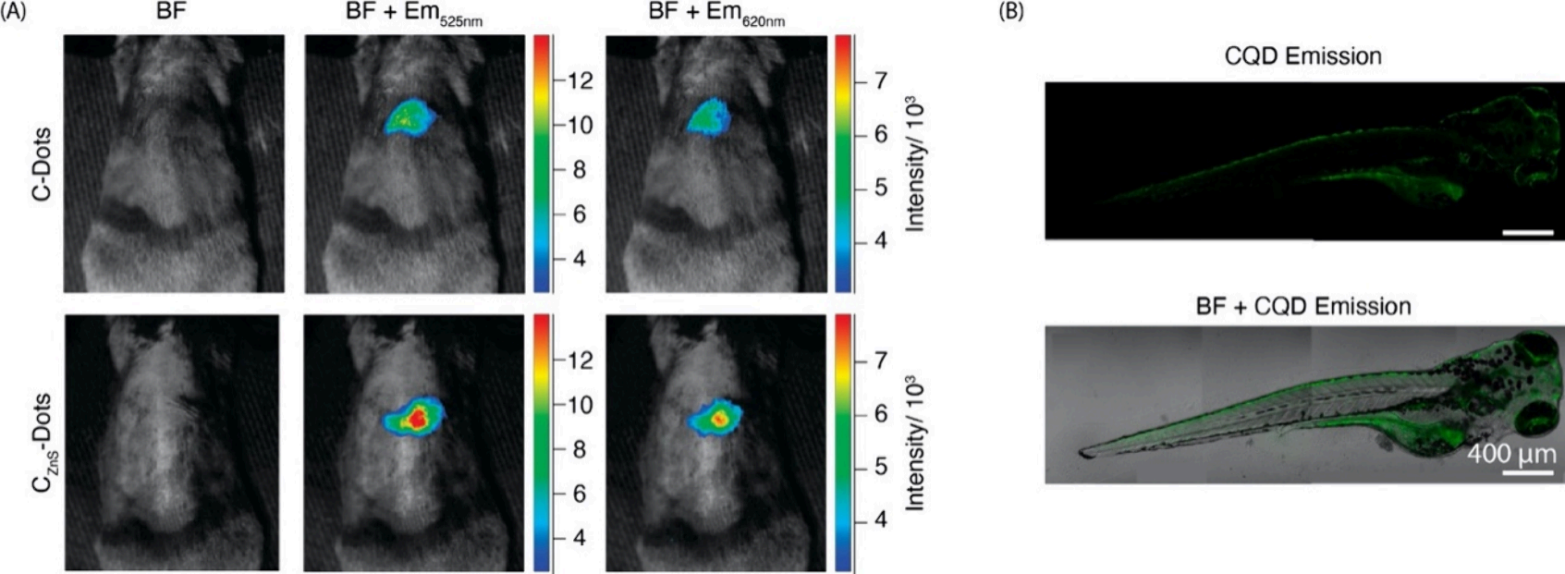
(A) Bright-field and merged fluorescence images of mice subcutaneously injected with CDs (top) and C_ZnS_-Dots (bottom). Emission at 525 and 620 nm were collected by 470 and 545 nm excitations, respectively. Adapted with permission from ref (937). Copyright 2009 American Chemical Society. (B) Fluorescence and bright-field merged images of a zebrafish incubated with CQDs at 488 nm excitation. Adapted with permission from ref (942). Copyright 2020 Dove Medical Press Limited.

Other types of CNMs have also been used for *in vivo* imaging. One of the early *in vivo* explorations of CDs involved engineering doped CDs with ZnS (C_ZnS_-Dots).^937^ Both CDs and C_ZnS_-Dots had comparable excitation and emission spectra, while the latter fluoresced brighter. These two materials were administered subcutaneously in mice and compared side-by-side to evaluate optical properties *in vivo*. As expected C_ZnS_-Dots appeared brighter in the injection area compared to undoped CDs (Figure 17A). After intravenous injection into mice, CDs were primarily excreted via urine. CD emissions were only detectable in kidneys and liver after 4 h post injection, which was consistent with the urine excretion pathway.

Several subsequent studies demonstrated the utility of CDs as contrast agents in living animals including mice and zebrafish (Figure 17B).^938−942^ In zebrafish, the biodistribution of CDs rapidly increased within the first 48 h in multiple organs including the yolk sac, intestine, stomach, and liver. The accumulation of CDs had no adverse effects on key biological processes, such as hatching rates, teratology, or mortality. To improve probe targetability, Wang et al. decorated nitrogen-doped CDs with *N*-methyl-2-pyrrolidinone (pN-CNDs) and demonstrated such two-component systems can hone in on specific cancerous tissue, such as glioma *in vivo*.^943^

The multimodal imaging and therapeutic applications observed in GOs and SWCNTs is prevalent in CDs as well. Ge et al. synthesized far-red and NIR-emissive CDs, which exhibited a broad emission profile with a maximum at 640 nm.^935^ When injected into tumor-bearing mice via intravenous injection, the majority of the red-emitting CDs were localized inside tumor site and the liver, presumably through the EPR effect. Furthermore, authors tested the efficacy of these CDs as photothermal therapeutic agents. The relative tumor volume in CD-treated mice diminished compared to control mice after phototherapeutic intervention, demonstrating the multifunctional potential of CNMs for biomedical imaging and therapy. Notably, CD-treated mice did not show signs of inflammation, necrosis, or apoptosis. In a subsequent study, Liu and co-workers synthesized red-emissive CDs from taxus leaves.^944^ After purification, the CDs displayed an excitation-independent emission maximum at 673 nm and a narrow full width at half maximum (fwhm) around 20 nm. Post-injection into mice, CD photoluminescence was detected in the liver, lungs, and kidneys. Although this method produced CDs with highly desirable narrow NIR emissions, limited water solubility and solvent-induced aggregations (which leads to fluorescence quenching) may hinder further biological applications.

#### 5.1.3 *In Vitro* Imaging in Reduced Preparations

The study of complex biological systems is often facilitated by employing reduced preparations, such as cultured cells and tissue slices. Cells in culture can retain some of the complexity of biological phenomena seen in tissues while offering a simplicity that makes them accessible for study. The advent of fluorescence microscopy has facilitated studies of cell biology *in vitro*. Fluorescence microscopy is quite advantageous because organelles, proteins, oligonucleotides can be labeled to reveal novel dynamical information that otherwise would have remained inaccessible. CNMs have advantageous properties including ease of synthesis, minimal photobleaching, and tunable emission properties. Similar to organic small molecule fluorescent probes, fluorescent CNMs can be used as either cellular or subcellular stains, or to detect biologically relevant analytes using their fluorescence modulations. In this section, we discuss examples of CNMs used as contrast agents for *in vitro* cellular imaging.

**Figure 18:**
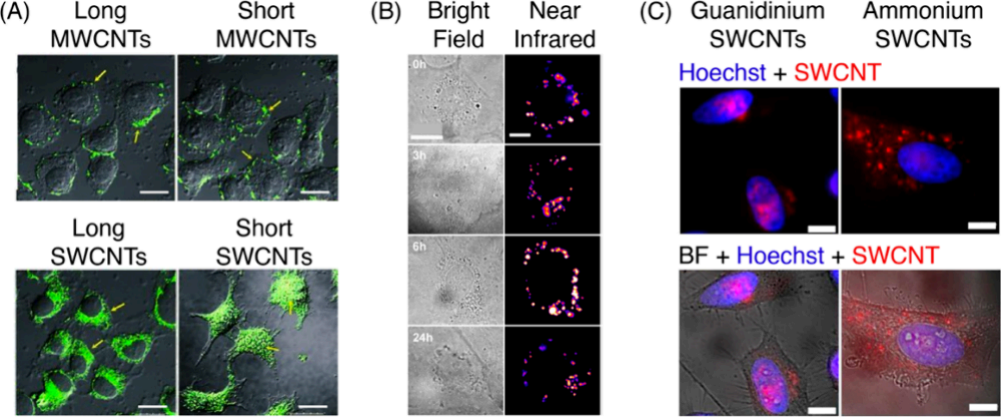
(A) Dependence of subcellular localization and cellular penetration on the type and length of the CNMs. The diameters of MWCNTs were 10–30 nm and SWCNTs were 1–3 nm. Length distributions for long MWCNT, short MWCNT, long SWCNT, and short SWCNT were 1–2 μm, 0.5–1 μm, 100–200 nm, and 50–100 nm, respectively. Nanotubes were conjugated to Alexa Fluor 488, and merged images of bright-field and fluorescence are presented. Scale bar is 20 μm. Adapted with permission from ref (960). Copyright 2010 Wiley-VCH. (B) Transmitted light and broadband NIR fluorescence (950–1350 nm) time lapse images of human umbilical vein endothelial (HUVEC) cells stained with 1 mg L^–1^ (GT)_30_-SWCNTs for 1 h. Scale bars for transmitted and NIR fluorescence images are 20 and 10 μm, respectively. Reproduced from ref (961). Copyright 2021 American Chemical Society. (C) Subcellular localization of HeLa cells costained with guanidinium- or ammonium-polymer coated SWCNTs and Hoechst 33258. Scale bar is 10 μm. Reproduced from ref (962). Copyright 2017 American Chemical Society.

Early studies of SWCNTs in biological milieu primarily employed SWCNTs as a scaffold for gene and drug delivery.^945,946^ Explorations of SWCNTs as contrast agents for fluorescence microscopy began shortly after the discovery of their intrinsic NIR fluorescence. An early report of studying SWCNT cellular uptake via imaging of their intrinsic NIR fluorescence was achieved by Pluronic F108-coated SWCNTs in mouse peritoneal macrophage cells.^947^ Such surfactant-suspended SWCNT uptake was limited to phagocytic cells. In a related study, Heller et al. decorated SWCNTs with an alternating guanine and thymine containing ssDNA (GT)_30_, which remained in live cells for up to three months.^948^ Interestingly, in cultured murine 3T3 cells, (GT)_30_-wrapped SWCNTs were internalized, and electron microscopy images revealed aggregates of nanotubes in the endosomes.^948^ This led to a series of studies related to mechanisms of ssDNA-functionalized SWCNT uptake in cells, which revealed that internalization occurs via endocytosis, which is followed by an endosomal escape, and leads to SWCNT accumulation in the cytosol (Figure 18).^949−954^

It should be noted that SWCNT uptake and final intracellular destination is influenced by many factors, including nanotube type (multi- or single-walled), purity, size/length, aggregation, and functionalization. To elucidate these parameters, Kang et al. carried out a study on intracellular uptake of SWCNTs and MWCNTs of different lengths.^955^ The study showed that MWCNTs were excluded from the cytoplasm, longer SWCNTs were internalized and resided exclusively in the cytosol, and shorter SWCNTs partitioned between both the cytoplasm and nuclei (Figure 18A). Apart from size, the properties of materials that coat SWCNTs also influence cellular uptake. For example, SWCNTs coated with bovine serum albumin (BSA), a protein often used to generate biocompatible and singly exfoliated dispersions of nanotubes, exhibit cellular loading that is primarily cytoplasmic in localization.^956^ Other studies have shown that the endocytosed nanotube’s cytosolic location can be programmed to drive preferential partitioning into various subcellular locations.^957,958^ A good example is the work of Heller and colleagues, where synthetic polymer–SWCNT hybrids were used to guide nanotube reporters to different subcellular compartments (Figure 18C).^959^ In this study, SWCNTs were functionalized with ammonium- and guanidinium-based polycarbodiimide polymers. SWCNTs with guanidium functionalization preferentially partitioned into the nucleus, while ammonium-based ones were excluded from the nucleus (Figure 18C). Initial entry into the cells was facilitated by endocytosis, and nuclear translocation for the guanidinium complex was mediated by the less common import receptor importin-β through an apparent non-canonical pathway.

Another study pursued a different strategy to direct the subcellular localization of internalized SWCNTs using canonical nuclear import pathways. Here, coating SWCNTs with the tail end of the nuclear protein lamin B1 (LB1) appeared to derive the partitioning of internalized SWCNTs from the cytoplasm into the nucleus.^958^ LB1 contains an exposed nuclear localization signal and as a result, LB1-functionalized SWCNTs were translocated into the nucleus, as evidenced by multimodal Raman and fluorescence imaging. While these studies show that controlling the SWCNT cellular distribution is possible through interfacial engineering, they also highlight the absence of generalized design principles for controlling SWCNTs' biodistribution in cells, and most results remain application- and discipline-specific.

**Figure 19:**
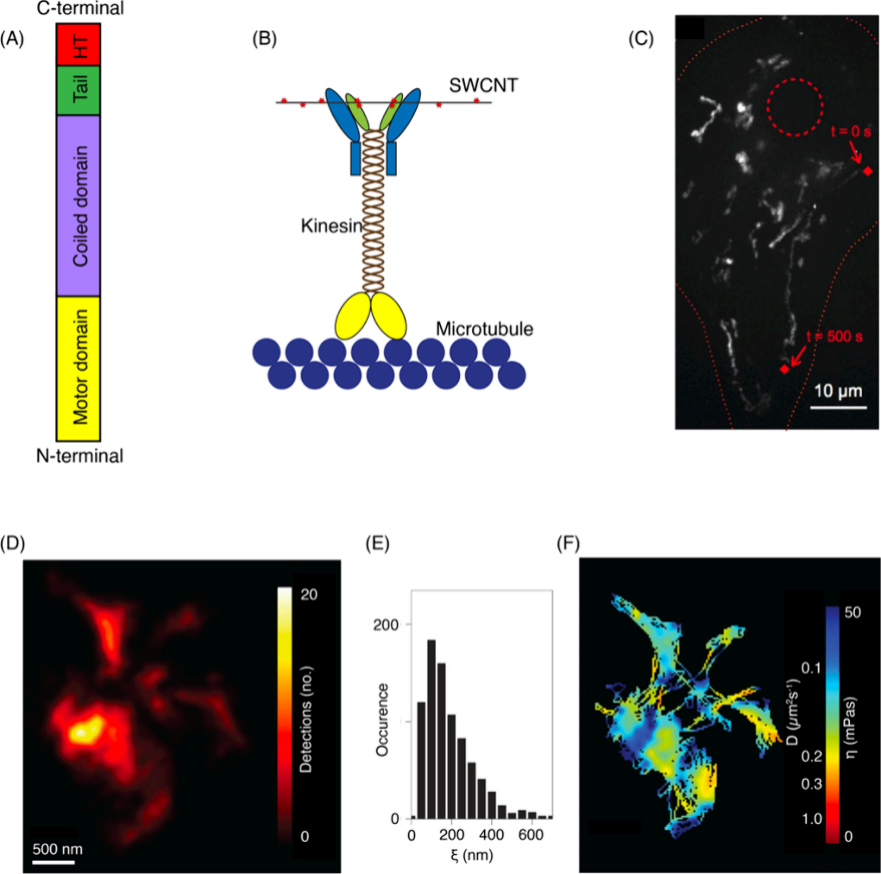
(A) Schematic of kif5c and HaloTag (HT) protein fusion. (B) SWCNTs are bound to the kinesin via their HT ligand surface motifs. (C) Movement of kinesin labeled with SWCNTs is tracked in a COS-7 cell line. Nucleus and periphery are outlined in red dashed and dotted lines, respectively. The red diamond marks beginning and end of the 500 s trajectory over 40 μm. Adapted with permission from ref (963). Copyright 1979 American Association for the Advancement of Science. (D) Super resolved image of an ECS obtained from 20,000 localizations of a diffusing SWCNT. (E) Characteristic length scales of ECS microdomains pooled from many tracking experiments. (F) Diffusion coefficients and viscosity of the ECS computed from single particle tracking experiments. Adapted with permission from ref (966). Copyright 2017 Springer Nature.

One advantage of the photophysics of SWCNTs is the ability to stably emit photons under prolonged excitations, enabling minutes to hours of continuous imaging, without the need to correct for photobleaching or blinking. This photostability can be exploited for single particle tracking experiments, as demonstrated by the use of SWCNTs for high resolution mapping the movement of kinesin along microtubules in live cells (Figure 19A–C).^963^ This strategy used Halo-tagged kinesin and SWCNTs functionalized with the HaloTag ligand. SWCNTs were delivered into cells via electroporation for kinesin labeling. Fakhri et al. utilized the stable photoluminescence of SWCNTs to track the motion of kinesins in live cells from milliseconds to hours. Based on the quantitative data collected from live cell imaging, authors show that thermal motion dominates cytoskeletal dynamics on short time scales, whereas motor protein-based transport is dominant at longer temporal scales. In another study, a green fluorescent protein (GFP)-tagged kinesin was labeled with SWCNTs bearing an anti-GFP nanobody. This afforded tracking motor protein movement in embryos of drosophila.^964^ In a related theme, single particle tracking experiments using PEG-functionalized SWCNTs have enabled super resolution mapping of the brain’s extracellular space (ECS) in brain slices.^965^ In this study, SWCNTs are non-covalently functionalized with pegylated phospholipids, which generate colloidally stable and biocompatible SWCNT dispersions. The functionalized nanotubes are delivered into mice brain by microinjection, where they localize to the ECS and allow single particle tracking experiments to be carried out (Figure 19D–F). The dynamic movement of isolated SWCNT emitters is tracked through a custom-made fluorescence microscope, enabling a high-resolution mapping of the morphology and rheology of the brain’s ECS (Figure 19D–F).^966^ In a follow up study, work from the same group demonstrated that single particle tracking of SWCNTs can reveal nanoscale differences in the morpho-rheological properties of the ECS in close proximity to synapses.^967^

Directing fluorescent probes to biologically important targets may be necessary to study the function of the target. Several strategies have been employed to direct SWCNTs to receptors, biomolecules, or organelles. Some of these include the engineering the biointerfacial properties of SWCNTs to localize them exclusively to the lumen of endolysosomal organelles,^968^ surface receptors via conjugation to antibodies^969^ and nanobodies,^970^ transmembrane receptors, such as integrin, by using peptides with integrin recognition sequences.^971^ SWCNTs have also been directed to proteins using antibodies,^972−974^ biotin–avidin interactions,^975^ or aptamers.^976^

The GO-family of CNMs have been used *in vitro* in cultured cells. Indeed, work by Al-Nahain et al. and Yang et al. utilized *in vitro* cellular assays in cancer cell lines as part of their studies that demonstrated the tumor-homing ability of GO’s *in vivo* (section 5.1.2).^977−979^ The *in vivo* dual use attributes of GO-based CNMs for imaging and therapeutics are applicable in cellular assays as well, as demonstrated by Li et al.^980^ Li and co-workers used two-photon excitation for imaging of GO-labeled cancer cells, which incidentally generated microbubbles upon laser excitation.^980^ The microbubbling caused cell death at an order of magnitude lower laser power than in non-labeled cells, making GOs an effective photothermal therapy reagent. Non-labeled cells tolerated 35 mW of power and required 40 mW to induce damage/death, while GO-labeled cells exhibited significant cell death when raster-scanned at 4 mW. Similarly, Pramanik and co-workers used two-photon imaging of methicillin-resistant *Staphylococcus aureus* (MRSA) labeled with GO.^981^ They reported excitation wavelength-dependent tunable emissions from the same GO material, which enabled multicolor imaging of bacterial cells with aptamer-functionalized GOs.

**Figure 20:**
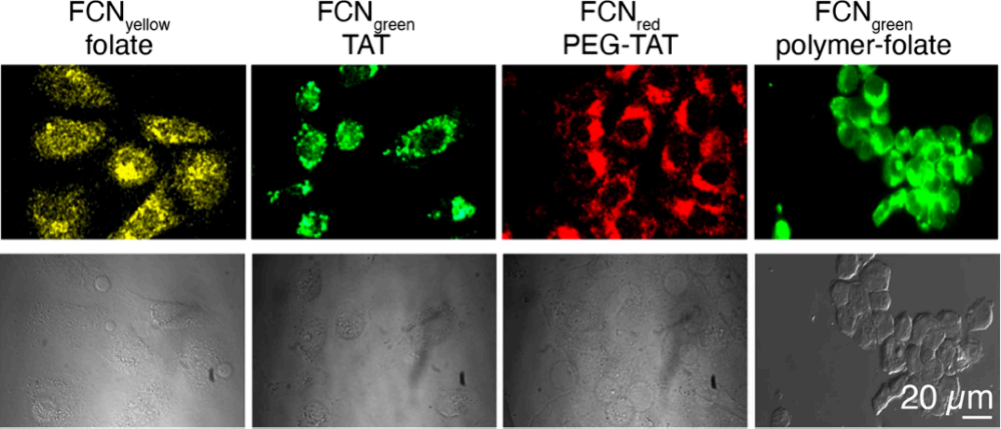
HeLa cells stained with functionalized fluorescent CDs with tunable emission profiles. Cells were imaged under fluorescence (top) and bright-field (bottom) modes. Adapted with permission from ref (983). Copyright 2013 Springer Nature. FCN stands for fluorescent carbon nanoparticles, which we collectively refer to as CDs in this review.

For *in vitro* live cell imaging, it is advantageous to have brightly fluorescent CDs. Prior to elemental doping and post-synthesis surface modifications, early synthetic methodologies produced weakly fluorescent CDs.^982^ However, recent studies, such as those by Bhunia et al. show that it is possible to synthesize highly fluorescent CDs with tunable emission profiles.^983^ As synthesized, these CDs are less than 10 nm in diameter and their optical properties exhibited dependence on the method of carbonization. For example, blue- and green-emitting nanoparticles were produced by carbonization of carbohydrates using sulfuric acid, whereas yellow- and red-emitting CDs required phosphoric acid. The quantum yield for these CDs ranged from 6 to 30% against fluoresceine standard. Synthesized CDs were hydrophobic, and surface functionalization with amphiphilic polymers afforded water solubility. Interestingly, further functionalization with affinity ligands enabled biolabeling. TAT-functionalized CDs exhibited increased cellular penetration and folate-functionalized CDs showed selective labeling of cells that express folate receptors (Figure 20).

A common strategy to improve CD fluorescence is through introduction of elemental precursors at the synthesis phase, a practice that is quite prevalent in the field of semiconductor nanoparticles to furnish brightly fluorescent emitters.^984^ Liu et al. generated smaller diameter (∼3.5 nm) multicolor fluorescent nanoparticles via nitrogen (N) and phosphorus (P) doping.^985^ These biocompatible NP-CDs were able to penetrate cell membranes effectively when tested in human cervical carcinoma SiHa cells. Indeed, heteroatom inclusion, most commonly encompassing phosphorus (P), boron (B), nitrogen (N), and sulfur (S), has been extensively employed to tune the photoluminescence properties of CDs.^986−989^ The brightness and emission spectra of CDs are sensitive to the doping agents and the method of synthesis. For example, nitrogen-doped CDs synthesized from citric acid and ethylenediamine by a hydrothermal method reported a QY of 80%,^990^ while N- and P-doped CDs synthesized using a microwave-assisted method had QY of only 17%.^991^ Cysteine is a common source of sulfur to synthesize S-doped CDs (s-CDs), and when combined with the hydrothermal method, s-CDs with QYs of 73% are reported.^992^ From these, nitrogen appears to be the most common dopant for CD photoluminescence tuning.

The highest reported QY for CDs is 94.5% and Liu et al. accomplished it by using folic acid as the nitrogen source.^993^ These blue-emitting CDs were bright and photostable when exposed to a continuous excitation light source for up to 150 min. When incubated with cancer cells presenting overexpressed folate receptors, preferential uptake of CDs was evident compared to control A549 cells. Remarkably, the source of N-precursors appears to influence the cellular permeability of CDs. For example, N-CDs synthesized by Li et al.^994^ using gelatin as a nitrogen source were able to penetrate and label the cytosol of A549 cells. In contrast, N-CDs synthesized by Liu et al. from folic acid were impermeable to A549 cells. In addition to these four common heteroatom dopants, CDs can also be doped with rare earth elements such as gadolinium and ytterbium.^995^ Incorporation of gadolinium permits MRI imaging, and ytterbium allows CT imaging due to its strong X-ray absorption coefficient. In combination with the fluorescence from the CDs, Gd/Yb@CDs afforded multimodal imaging both *in vitro* and *in vivo*.^813−816^

Similar to other CNMs, it is thought that CDs enter cells via endocytosis and escape the early endosome through energy and cell-dependent mechanisms.^996,997^ To integrate subcellular targetability into CDs, reactive ligands incorporating recognition moieties can be installed. As a proof of concept, Cheng et al. included *meta*-phenylenediamine and triethylenetetramine in the CD synthesis phase, in which the resulting CDs showed a strong affinity for cellular RNA.^998^ The affinity toward RNA was thought to be mediated by possible π-interactions of isoquinoline moieties in CDs with the major groove of RNA. However, the implementation of this sensing strategy was limited to RNA and did not extend to other analytes.

Cell biological studies often involve the study of subcellular compartments and their real-time dynamics. With the aid of high affinity chemical moieties, CDs have been directed to different subcellular targets, including nuclei,^999^ lysosomes,^1000^ mitochondria,^1001^ and components of the extracellular matrix such as hyaluronan.^1002^ However, these approaches appear to employ one-off strategies and occasionally rely on insufficiently understood intrinsic properties of CDs to mediate subcellular targeting. If rationally designed targeting motifs, such as HaloTag and SNAP tag ligands, can be incorporated into CDs, these can be coupled with genetic perturbations for a more modular targeting of subcellular structures, as described for small molecule organic fluorophores.^1003^ This approach could be compelling to biologists for experiments that leverage CDs' photostability and compatibility of their photoluminescence with commercially available microscopes. In addition to CDs, other CNMs, including GO and RGO, have been employed for *in vitro* imaging and delivery of cargo into cells.^1004,1005^

Despite the expanding application of CDs in biology, organic dyes and fluorescent proteins (FPs) remain preferred reagents for imaging. Tunability of emission could be a competitive advantage for CDs, particularly in the NIR region of the spectrum where high performing organic dyes and FPs are still hard to find. Previous attempts at red shifting the CD emission required complicated synthesis and extensive purification steps.^1006−1008^ To address this issue, Sun et al. prepared red-emissive CDs via microwave-assisted synthesis from citric acid and formamide precursor materials.^1009^ The synthesized CDs were ∼4 nm in diameter and displayed emission at 640 nm when excited using a 540 nm laser. Cell staining experiments revealed red CDs can effectively permeate past the cell membranes of MCF-7 and HeLa cells. Interestingly, the CDs passed through the nuclear pore complex and translocated to the nucleolus. Furthermore, the authors used these CDs as a vehicle to deliver cell impermeable therapeutic agents, demonstrating yet again the general suitability of CNMs to function as multimodal experimental reagents.

**Figure 21:**
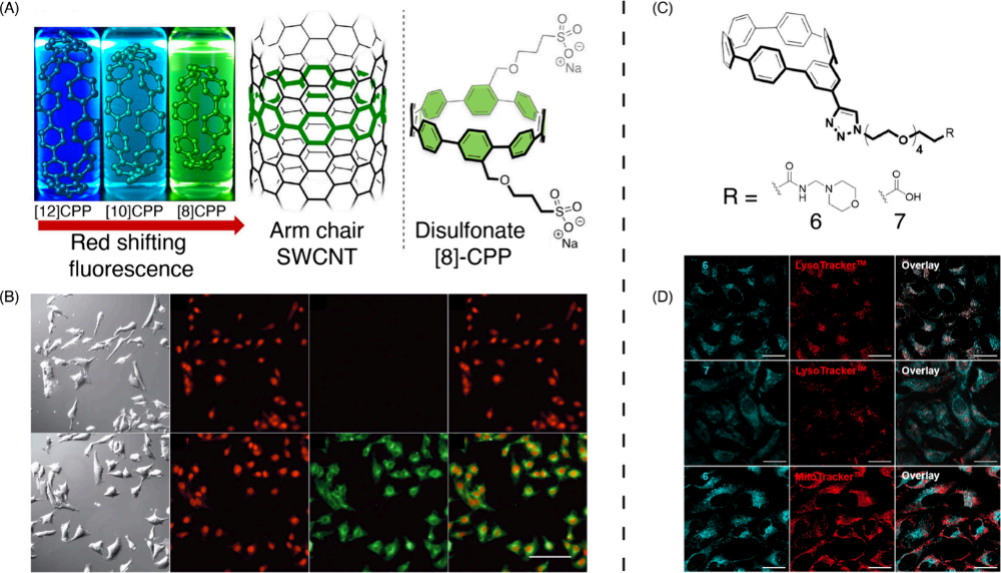
Live cell imaging using carbon nanohoops. (A) CPPs can be conceptualized as the smallest macrocyclic slices of an armchair nanotube. Notice the counter-intuitive red shifting of fluorescence as ring size decreases. Right: structure of cell permeable disulfonate [8]CPP, used for live cell imaging depicted in panel B. (B) Bright-field, nuclear (NucRed, red) and cytoplasmic (disulfonate [8]CPP, green) images of HeLa cells, and overlay between red and green channels. Top row: imaged in the absence of disulfonate [8]CPP. Reproduced from ref (1011). Copyright 2018 American Chemical Society. (C) Structure of *meta*[6]CPP with PEG chains to enhance aqueous solubility, capped with subcellular targeting ligands (R). (D) Top row: Lysosome-targeting motif enables localization of *meta*[6]CPP punctate signal to lysosome (good overlap with LysoTracker). Middle row: Nanohoop without lysosome-targeting motif exhibits diffuse labeling and poor overlap with LysoTracker. Bottom row: lysosome-targetted nanohoops show poor overlap with MitoTracker, a mitochondrial marker. Reproduced from ref (1013). Copyright 2021 American Chemical Society.

The newest and smallest class of fluorescent CNMs, carbon nanohoops, have also been leveraged for cell imaging studies. Although numerous studies and reviews have noted the promise of carbon nanohoops as biological imaging agents,^428^ applications of these materials have been slow to emerge owing to low solubility in aqueous mediums, but encouraging advances have been made to ameliorate this.^1010^ A seminal study by White and co-workers has achieved water solubility through the synthesis of sulfonate-functionalized carbon nanohoops.^1011^ Their disulfonate-[8]CPP (excitation: 328 nm, emission: 510 nm) showed no cytotoxicity at concentrations of up to 10 μM and good cell permeability when used for live cell staining and imaging in HeLa cells (Figure 21). The study showed that HeLa cells internalized the nanohoops with good colocalization towards the cytosol over mitochondria (Figure 21B). The authors further modified the reagent to include a clickable handle in place of the sulfonate group (Figure 21C). Here, the attachment of a folate group afforded successful targeting of folic acid receptors, which are known to be overexpressed in cancer cells (Figure 21D).^1013^

In a subsequent study, Lovell and co-workers used a *meta*[6]CPP (excitation: 328 nm, emission: 519 nm), and demonstrated a simpler, higher yield synthesis of nanohoops, and further explored their cytotoxicity, cellular uptake, and subcellular targeting in HeLa cells.^1013^ Their approach employed the use of clickable constructs and attachment of water-solubilizing PEG groups that are terminated with a carboxylic acid or morpholine groups (Figure 21C). When incubated with HeLa cells, the carboxylic acid-terminated CPPs were found throughout the cytosol, to a lesser extent in the nucleus, and had no colocalization with LysoTracker in the lysosomes (Figure 21D). In contrast, the morpholine-terminated CPPs were observed to be sequestered as puncta outside the nucleus and had strong colocalization with LysoTracker in lysosomes and minimal colocalization with MitoTracker in mitochondria, demonstrating a successful subcellular targeting. This study provided evidence of CNH uptake via endocytosis. Moreover, it demonstrated that these nanomaterials can be used for two-photon imaging in U2OS cells, reporting a peak two-photon response at 720 nm with a 65 GM absorption cross section. The authors demonstrate photostability at 100 mW for continuous imaging of up to three minutes.^1013^ Other studies have similarly sought to improve solubility of CPPs. Park and co-workers developed a water-soluble oxidized-[5]CPP (Oxi-[5]CPP) by exposing [5]CPP to air at room temperature for 24 h.^1014^ These Oxi-[5]CPPs were then packaged into liposomal nanoparticles via thin-film hydration and subsequently applied to HeLa cells (excitation: 335 nm, emission: 446 nm). Internalized Oxi-[5]CPP-liposomes efficiently labeled the cytosol, and no reduction in cell viability was observed at concentrations of up to 40 μM.^1014^

### 5.2 Environmental and Food Sample Imaging with CNMs

#### 5.2.1 Bacteria Imaging

Bacterial infections have become a growing concern in recent years, largely due to the emergence of antibiotic-resistant strains.^1015^ Shockingly, more than 1.2 million people died in 2019 due to antibiotic-resistant bacterial infections.^1016^ Given the circumstances, the urgency to develop innovative and real-time techniques for monitoring and mitigating bacterial pathogens in both food and environmental contexts has never been more critical. To date, there has been a noticeable gap in the availability of real-time bacterial detection methods. Existing techniques include polymerase chain reaction (PCR),^1017^ matrix-assisted laser desorption ionization time-of-flight mass spectrometry (MALDI-TOF MS),^1018^ enzyme-linked immunosorbent assays (ELISA),^1018,1019^ and traditional microbiological counting methods.^1015^ These methods have excelled in quantifying and detecting bacterial pathogens due to their high specificity and sensitivity.^1020^ Nevertheless, they harbor inherent drawbacks, such as the requirement for sophisticated preparation procedures and inability to deliver prompt, spatially, and temporally precise detection of bacteria.^1018,1019^ This has highlighted the necessity for more advanced bacterial imaging and sensing techniques. In response, CNMs have shown potential as fluorescent imaging probes for pathogenic bacteria.

In this section, we provide a comprehensive summary of the recent developments in the use of CNMs as fluorescent probes for imaging bacteria. Our focus is on literature published after 2017 with special attention to studies that have utilized these probes in analyzing real-world environmental and food samples. To gain a broader understanding of CNMs and other nanomaterials used for environmental or food sample imaging, readers are encouraged to refer to these other reviews.^1021−1024^

Among all CNMs, CDs stand out as the most utilized fluorescent probes for environmental bacterial monitoring. This preference can primarily be attributed to the facile, scalable, and affordable synthesis methods for CDs in contrast to the more intricate procedures required for other CNMs, such as chemical vapor deposition, arc discharge, or laser ablation. Additionally, CDs exhibit a distinct advantage with their intrinsic bright fluorescence in the visible light spectrum. While SWCNTs can display NIR fluorescence, their fluorescence signals are generally weaker compared to CDs and require specialized instrumentation for detection that limits feasibility in environmental settings. Lastly, other CNMs, such as MWCNTs, graphene, and fullerenes, do not possess intrinsic fluorescent properties.^1025−1028^

**Figure 22:**
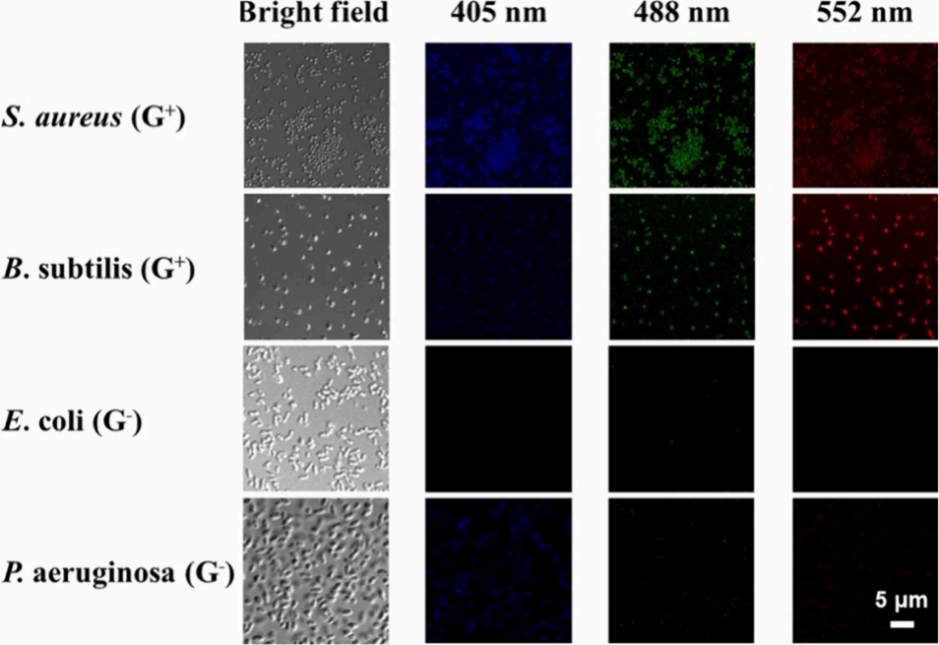
Confocal images after a 2-h treatment with CDs@MR-1, showcasing two Gram-positive bacterial strains (*S. aureus* and *B. subtilis*) and two Gram-negative bacterial strains (*E. coli* and *P. aeruginosa*). Adapted with permission from ref (1031). Copyright 2022 Elsevier.

Compared to traditional fluorescent probes based on metallic quantum dots or organic dyes, such as vancomycin and hexidium iodide,^1022,1029,1030^ CDs overcome issues of easy oxidation and photobleaching, high toxicity, and low quantum yield. For instance, CDs@MR-1 developed by Shen et al. using a one-step hydrothermal process from *Shewanella oneidensis* MR-1 bacterium has demonstrated exceptional abilities in selectively interacting with Gram-positive bacteria, effectively distinguishing them from Gram-negative bacteria.^1031^ They incubated these CDs with either Gram-positive (*S. aureus* and *B. subtilis*) or Gram-negative bacteria (*P. aeruginosa* and *E. coli*). After a two-hour treatment, CDs incubated with the two Gram-positive bacteria, but not the Gram-negative bacteria, exhibited blue, green, and red fluorescence emissions under different excitation wavelengths (405, 488, and 552 nm), respectively (Figure 22). The utility of CDs@MR-1 extends beyond bacterial imaging, as researchers also demonstrated its high sensitivity and selectivity in detecting environmental pollutants in real water samples, including Hg^2+^ ions and the antibiotic tetracycline. This broadens the scope of bacteria-sourced CDs, making them valuable tools not only in bacterial imaging studies, but also in environmental monitoring. While this work on CDs@MR-1 represents a significant advancement in the field, it falls short in elucidating the underlying reasons for the selective interaction of these CDs with Gram-positive bacteria.

**Figure 23:**
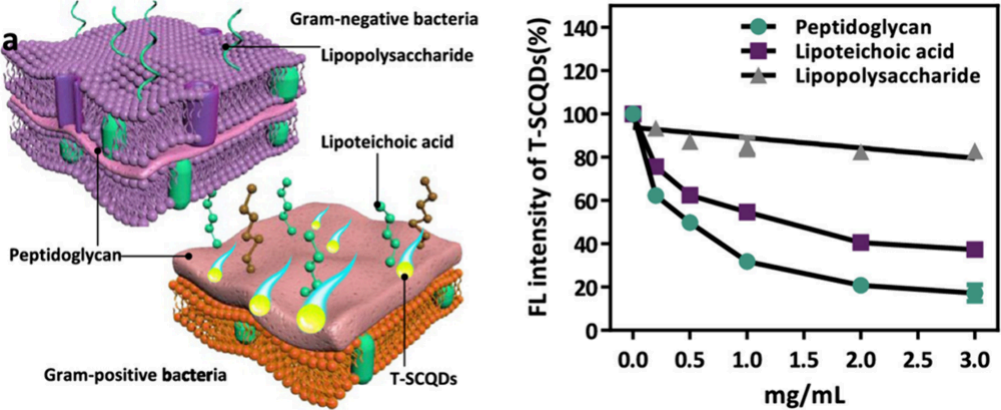
(Left) Schematics of Gram-negative and Gram-positive bacteria cell walls. (Right) Competition assay using T-SCQDs at a concentration of 500 μg/mL with peptidoglycan, lipoteichoic acid, and lipopolysaccharide to assess their binding affinity toward peptidoglycan and lipoteichoic acid. Adapted from ref (1032). Copyright 2021 American Chemical Society.

In another study, Yan et al. developed L-tryptophan modified carbon quantum dots (T-SCQDs) that also exhibited a specific staining pattern for Gram-positive bacteria.^1032^ Their experiments revealed that T-SCQDs bound more effectively to the peptidoglycan and lipoteichoic acid cell wall layers in Gram-positive bacteria compared to the lipopolysaccharides in Gram-negative bacteria (Figure 23), providing valuable insights into the specific detection mechanisms that were missing in previous studies. This specificity arises from the distinctive chemical compositions of bacterial cell walls. Specifically, lipoteichoic acids (−7.70 mV) and peptidoglycans (−12.37 mV) exhibit more negative zeta potentials compared to lipopolysaccharides (−3.84 mV). This difference grants Gram-positive bacteria an increased number of anionic sites, ultimately enhancing their electrostatic interaction with the cationic amino groups of T-SCQDs. Furthermore, peptidoglycan’s structure, comprising of N-acetylglucosamine and N-acetylmuramic acid linked by short peptides, provides ample hydrophobic regions for the benzopyrrole structure of T-SCQDs to interact. Additionally, the abundant hydrophilic groups on the peptidoglycan surface allow hydrogen bond formation with the hydroxyl groups on T-SCQDs. These interaction mechanisms together might potentially explain why, even though T-SCQDs have negative zeta potentials, they are still able to target the peptidoglycans of Gram-positive bacteria rapidly and selectively.

Although electrostatic and weak intermolecular interactions may play a crucial role in distinguishing between Gram-positive and Gram-negative bacteria, their applicability in real environmental samples can be limited by various confounding factors. In complex matrices, such as natural waters or food samples, the presence of other ions, molecules, and particulate matter can interfere with these interactions, potentially leading to false results. Moreover, although the aforementioned studies offer a general distinction between Gram-positive and Gram-negative bacteria, they do not enable the precise identification of bacterial species or strains. Considering these challenges, there is a pressing demand for more specific and robust detection methods that can target specific types and strains of bacteria with high accuracy.

The exploration of molecular recognition elements for bacterial targeting has revealed a diverse toolkit. Antibodies have been the preferred option due to their unparalleled specificity and ease of use,^1033^ but they can be expensive and impacted by environmental factors such as pH and temperature. In comparison, antibiotics and lectins, which achieve their antimicrobial action by attaching to the bacteria’s cell wall or outer membrane, are more affordable but often lack the desired selectivity. Aptamers stand out in this landscape as their binding specificity rivals that of antibodies, yet they are more versatile—easy to synthesize, resilient under various conditions, and adaptable.^1034^ All of this makes them suitable for bacterial detection in environmental samples, offering a balance of specificity, stability, and practicality.

**Figure 24:**
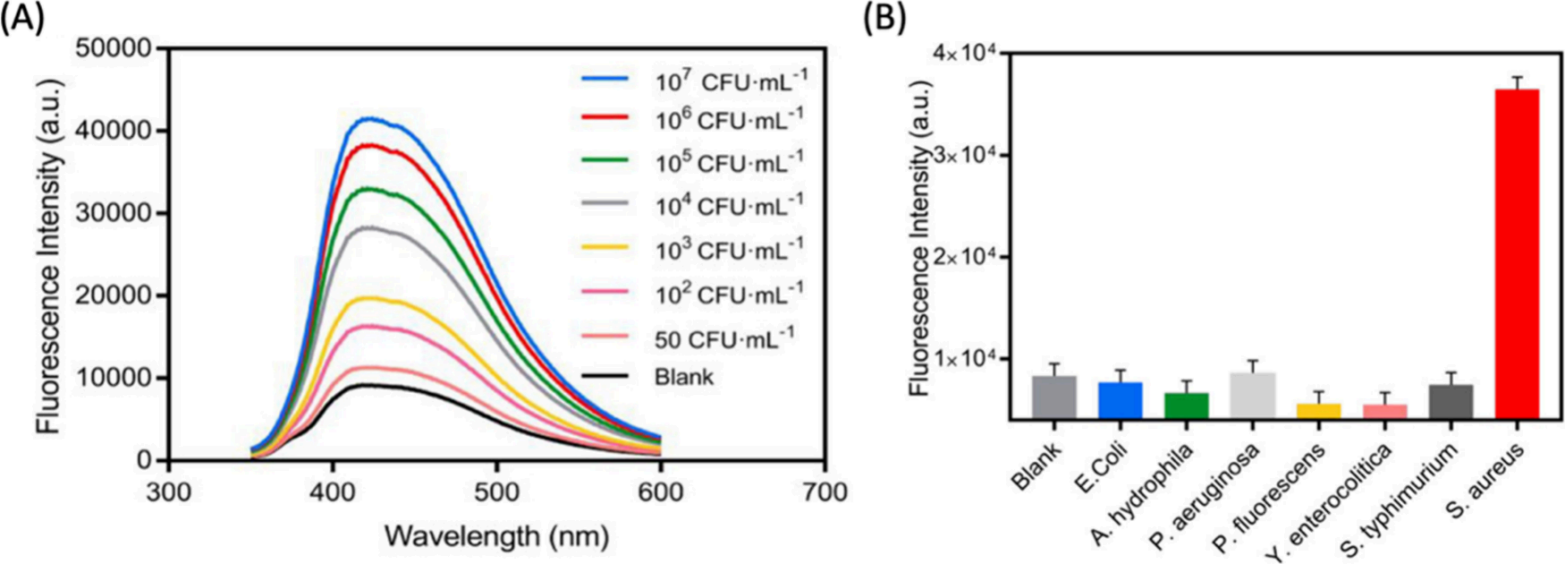
(A) Fluorescence emission spectra of Fe_3_O_4_/CD aptasensor after incubation with different concentrations of *S. aureus in vitro*. (B) An investigation into the aptasensor’s specificity for the detection of *S. aureus*, *E. coli*, *A. hydrophila*, *P. aeruginosa*, *P. fluorescens*, *Y. enterocolitica*, and *S. typhimurium*, each at a concentration of 10^5^ CFU·mL^–1^. Adapted from ref (1035). Copyright 2019 American Chemical Society.

One application of this technology is the aptasensor developed by Cui et al., which employs a combination of CDs and Fe_3_O_4_ nanoparticles, both modified with DNA sequences.^1035^ The Fe_3_O_4_ nanoparticles are coated with DNA aptamers specific to *Staphylococcus aureus*, while the CDs are modified with complementary DNA (cDNA). In the absence of *S. aureus*, these components interact, leading to the quenching of CD fluorescence via fluorescence resonance energy transfer (FRET). However, when *S. aureus* is present, the aptamer binds to the bacteria instead of the cDNA, causing the disassembly of the Fe_3_O_4_/CD complex. This leads to a recovery in CD fluorescence, clearly signaling the presence of *S. aureus*. The sensor’s outstanding sensitivity and specificity are evident in its impressively low detection limit of 8 colony forming units per milliliter (CFU/mL), along with a noticeable surge in fluorescence intensity when compared to six other bacterial types, as depicted in Figure 24. Its feasibility is further established through the accurate detection of *S. aureus* in food samples, such as milk and juice, achieving recovery rates (defined as the bacteria amount measured by the sensor divided by the actual amount spiked in the samples) of 95% to 106%. These results, comparable to traditional plate counting methods, underscore the sensor’s potential for rapid, reliable, and specific bacteria detection across various samples.

**Figure 25:**
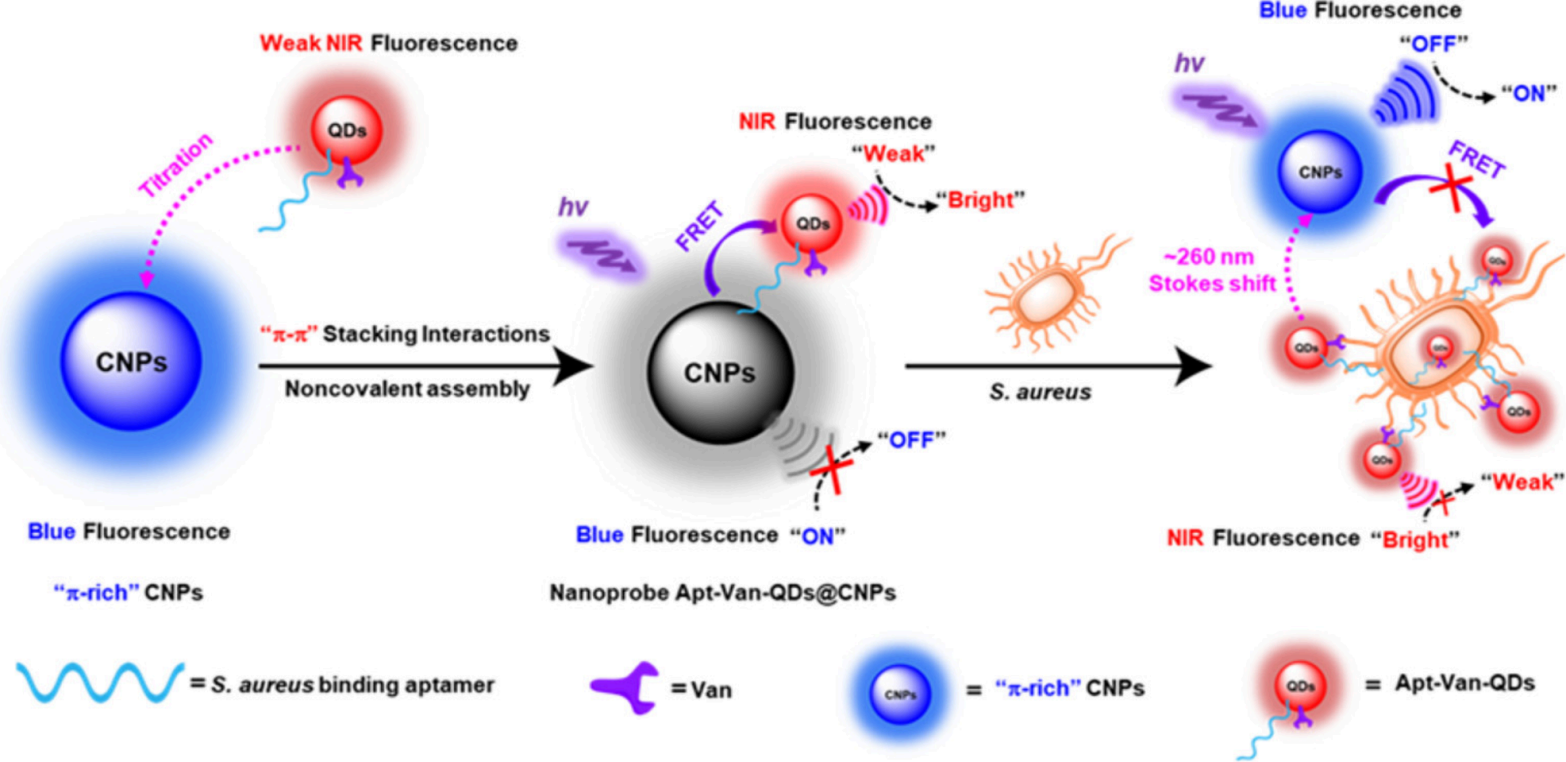
Ratiometric fluorescent nanoprobe, which utilizes both vancomycin and aptamer dual-recognition elements, offers an extensive Stokes shift. Adapted from ref (1036). Copyright 2020 American Chemical Society.

Despite these advances, the aptasensor faces challenges due to the susceptibility of base sequence-assembled aptamers to degradation in microbial environments. This susceptibility can lead to premature breakdown, risking unreliable outcomes and false positives in pathogen detection. To address this limitation, researchers developed a novel dual-recognition ratiometric fluorescent nanoprobe, named Apt-Van-QDs@CNPs,^1036^ to achieve rapid and sensitive detection for *S. aureus* at the single-cell level. This innovative probe combines NIR-fluorescent Apt-Van-QDs and blue-fluorescent π-rich electronic carbon nanoparticles (CNPs) to create a mechanism that relies on both an *S. aureus*-specific aptamer and vancomycin, a broad-spectrum antibiotic, for precise and rapid bacterial targeting. The proximity of CNPs (energy donors) and Apt-Van-QDs (energy acceptors) facilitates FRET, leading to an observable change in fluorescence. In the absence of *S. aureus*, the FRET process occurs smoothly, resulting in blue fluorescence quenching of CNPs and enhanced NIR fluorescence from the Apt-Van-QDs. However, when *S. aureus* is present, it binds to the Apt-Van-QDs, disrupting the FRET process and causing a significant fluorescence shift, characterized by increased blue fluorescence from CNPs and reduced NIR fluorescence from Apt-Van-QDs (Figure 25). Due to its dual fluorescence property, the sensor can operate ratiometrically, offering self-calibration and heightened sensitivity with a detection limit of 1 CFU/mL. Moreover, further studies have confirmed the feasibility of this nanoprobe in real-world conditions. Apt-Van-QDs@CNPs were successfully tested in complex media such as commercial milk, orange juice, and riverine water without any pretreatment, resulting in highly accurate detection of *S. aureus*, with recovery rates ranging from 97.00% to 103.00%. These findings underscore the effectiveness of the probe in actual samples and bolster its potential in food safety and environmental monitoring applications.

**Figure 26:**
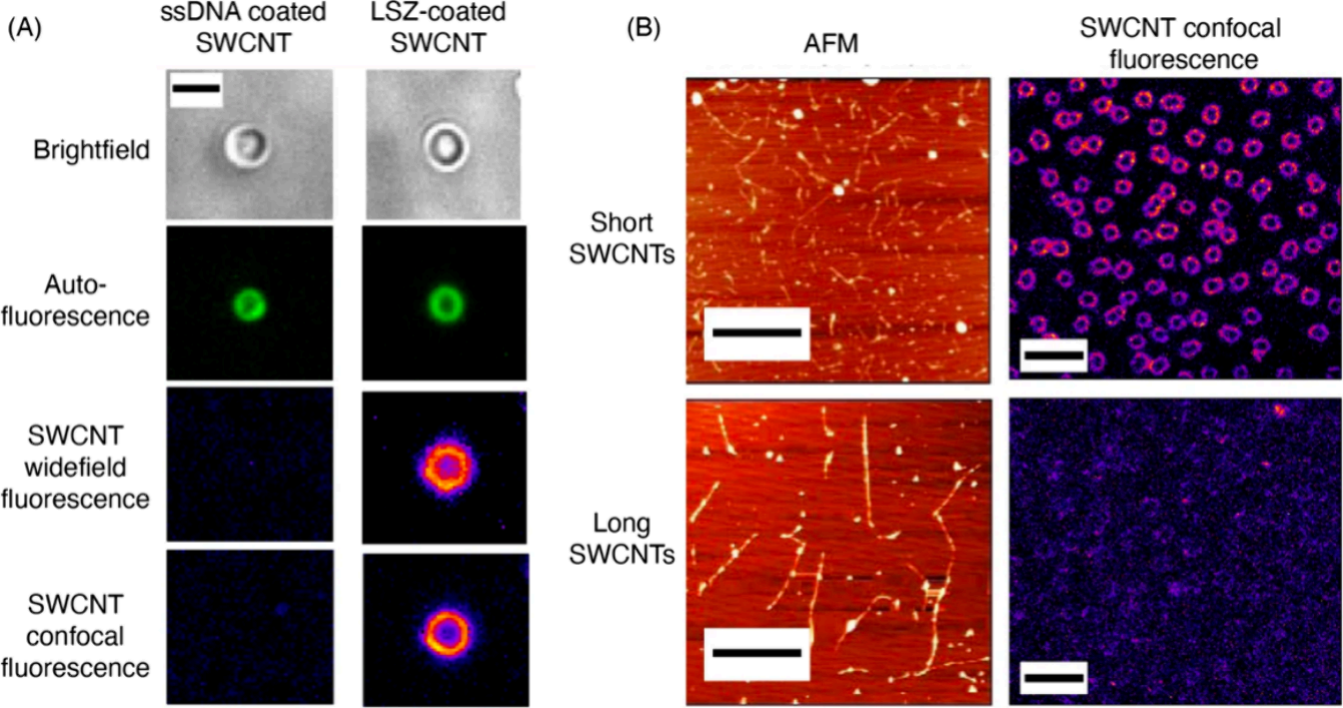
(A) Bright-field, autofluorescence, and NIR fluorescence (both under wide field and confocal modes) of *Synechocystis* cells incubated with ssDNA- or LSZ-wrapped SWCNTs. Note LSZ-coated SWCNTs efficiently label bacterial cells, while ssDNA-coated SWCNTs do not (scale bar = 3 μm). (B) AFM images of short and long SWCNTs (scale bar = 1 μm) and NIR fluorescence images of *Synechocystis* cells incubated with short and long SWCNTs (scale bar = 10 μm). Adapted with permission from ref (1037). Copyright 2022 Springer Nature.

Furthermore, alongside advancements in the utilization of CDs, notable strides have been made in the application of SWCNTs for bacterial imaging purposes. In a recent publication, Boghossian and colleagues explored the uptake of SWCNTs in Gram-negative cyanobacteria.^1037^ In this study, the authors first decorated SWCNTs with different amphiphilic polymers carrying varying degrees of electrostatic charges, leading to SWCNT dispersions of varying zeta potentials. When tested in intact unicellular cyanobacteria (*Synechocystis*), (AT)_15_ coated-SWCNTs were unable to penetrate the outer peptidoglycan coat. On the other hand, lysozyme (LSZ)-coated SWCNTs (LSZ-SWCNT) showed colocalization in the same bacterial cell lines (Figure 26A). To verify internalization, a strong SWCNT fluorescence quencher (K_3_[Fe(CN)_6_]) was introduced into cells that were pre-incubated with LSZ-SWCNT conjugates. NIR fluorescence near periphery of cells were strongly quenched by K_3_[Fe(CN)_6_]. However, inside the cytosol, SWCNT NIR-emissions were retained. This showed LSZ-decorated SWCNTs can efficiently enter bacterial cells, whereas (AT)_15_-decorated SWCNTs cannot. Furthermore, similar to observations found in eukaryotic cells, short LSZ-SWCNTs were found to be better at penetrating cells compared to long LSZ-SWCNTs (Figure 26B). Since LSZ can hydrolyze the peptidoglycan network, the authors used thermally inactivated LSZ to explore if the enzymatic activity of LSZ is responsible for facilitating nanoparticle cellular entry. Notably, they found that the inherent physicochemical characteristics of LSZ are responsible for internalization rather than their enzymatic activity. Indeed, the study revealed that internalization was higher for SWCNT suspensions with positive zeta potentials, while negatively-charged dispersions of SWCNTs were unable to cross the cyanobacteria cell membrane. Remarkably, the internalized CNMs did not inhibit the photosynthetic activity of the cyanobacteria, but rather appeared to facilitate exoelectrogenicity (i.e., transfer of electrons from the bacteria into the extracellular space), opening a nascent and tantalizing role for CNM-bacterial hybrids as living photovoltaic devices.

In the field of CNM-mediated bacterial imaging for environmental and food samples, research is still relatively scarce compared to medical applications. Beyond our current discussions, there remain numerous unexplored avenues that researchers can potentially delve into. An important example includes the development of probes for biofilm detection. In natural settings, microbial communities frequently form biofilms, characterized by robust and adhesive extracellular polymeric substances. These biofilms present notable challenges for fluorescent imaging by restricting dye penetration into the biofilm structure, and the dyes can cause damage to biofilms.^1038−1040^ Although we have not seen any studies conducted in actual environmental or food samples, one study did investigate a type of carbon dots (CD-605) capable of directly and specifically labeling microorganisms within biofilms,^1041^ bypassing the need for incubation, protection, or washing steps. CD-605 features negatively-charged carboxyl groups that facilitate strong electrostatic repulsion against similarly charged microorganisms. This repulsion is further enhanced by its hydrophilic nature, which is marked by the presence of −COOH and −OH groups, inhibiting hydrophobic interactions with microorganisms. Due to its highly negative and hydrophilic surface, CD-605 is prevented from penetrating live, planktonic microorganisms. However, its remarkably small size enables it to infiltrate dead microorganisms that have permeable cell walls and membranes. This unique characteristic allows for the selective staining of dead cells within a biofilm. Importantly, these CDs do not disrupt the biofilm structure and show greater photostability compared to the commercial dye SYTO 9, ensuring reliable imaging of biofilm-associated microorganisms.

Besides biofilm imaging, live/dead staining techniques are valuable in environmental and food sample analysis, particularly for quality control and assessing environmental impacts. In the food industry, these techniques can help determine shelf life by monitoring bacterial load, setting expiration dates, and maintaining product quality. In environmental research, they can be used to evaluate the impact of pollutants and ecological changes on microbial life, which is crucial for ecosystem conservation and management. Recent advancements include the use of CNMs as probes for live/dead staining. For instance, nitrogen and phosphorus co-doped carbon dots (NPCDs), synthesized through a one-step hydrothermal reaction of ethylenediamine and yeast extract,^1042,1043^ possess an enhanced negative charge distribution on their surface, enabling them to selectively stain dead bacteria due to electrostatic repulsion from the negative charges on intact bacterial cell walls. NPCDs can stain dead bacteria within two hours, providing detection accuracy on par with traditional plate counting methods but more rapidly. Similarly, Hua et al. developed CDs with low cytotoxicity and multicolor fluorescence emission properties, suitable for live–dead bacterial staining.^1044^ In their comparative studies with the conventional viability dye propidium iodide, they found that CDs showed a higher efficacy in differentiating dead from live cells. Similar to previous studies, this paper suggests that the selective staining by CDs is due to their negatively charged surface, which leads to electrostatic repulsion and selective permeability. This feature prevents the CDs from penetrating live cells with intact membranes, thus enhancing their specificity for dead cells. Furthermore, CDs offer several advantages over propidium iodide, including multicolor imaging, superior photostability, and notably, significantly lower cytotoxicity. These findings collectively highlight the potential of CNMs to be used as live/dead fluorescence probes in real samples.

#### 5.2.2 Plant Imaging

In plant biology research, imaging is essential for studying plant internal structures, from organelles to entire organisms.^1045^ This process is crucial for understanding plant development and the relationships between structure and function. However, imaging plant cells is challenging due to their distinct structural characteristics. The cell walls, composed mainly of cellulose, hemicellulose, and pectin, create a dense and rigid barrier that hinders the penetration of imaging probes. Furthermore, chlorophyll in plant leaves and lignin in cell walls lead to high levels of autofluorescence, complicating the imaging process and leaving much to the researcher’s interpretation.^1046,1047^

To address these challenges, scientists have been exploring alternative approaches. Microinjection, for instance, involves directly inserting imaging reagents into the cells, bypassing the cell wall. However, this technique must contend with the limited space in the cytoplasm and avoid disrupting the structure and function of organelles and cytoskeletal structures.^1048^ Engineered CNMs, especially CDs and SWCNTs, have emerged as promising alternatives to traditional bioimaging agents for use in plants.^1049−1052^ This is primarily due to their small size, ability to enter plant cells efficiently, bright intrinsic fluorescence, resistance to photobleaching, and the ability to fine-tune their emission range. This section delves into the recent applications of these CNMs in plant cell imaging, highlighting their role in overcoming traditional barriers in plant biology research. Here, we explore how the unique characteristics of CDs and SWCNTs enable more effective and detailed visualization of plant structures, from cell walls to organelles such as chloroplasts and mitochondria.

In a pioneering study, Giraldo et al. successfully utilized SWCNTs for bioimaging in plants focusing on exploring various plant components, including the lamina, veins, and chloroplasts, allowing for the detailed visualization of these structures deep within plant tissues.^1053^ A key aspect of their approach was leveraging the unique fluorescence emission properties of SWCNTs in the NIR spectrum, specifically beyond 1100 nm. This wavelength is particularly effective because it minimizes interference from chlorophyll autofluorescence, thereby facilitating clear imaging of plant chloroplasts. Previously, plant researchers had no means of visualizing these structures via fluorescence microscopy, as typical probes cannot reach chloroplasts and the chlorophyll autofluorescence often overlaps with that of probes. Furthermore, photobleaching resistance of SWCNTs marked a remarkable advancement in plant imaging, introducing a novel tool for conducting in-depth biological studies over long-time frames to study plant development and effects of environmental changes on plants.

Since then, numerous research teams have made substantial strides in developing novel CNM fluorescent probes designed to target different organelles and subunits in plants, which are detectable through various fluorescent imaging modalities. An intriguing imaging target is the plant cell wall. This crucial biosurface, pivotal in interactions with nanomaterials in both terrestrial plants and aquatic algae, remains a significant barrier to the delivery of genetic materials, nutrients, and pesticides.^1054,1055^ A deeper understanding of nanoparticle–cell wall interactions could revolutionize agricultural nanotechnology, enhancing crop productivity and sustainability with minimal environmental impact.

**Figure 27:**
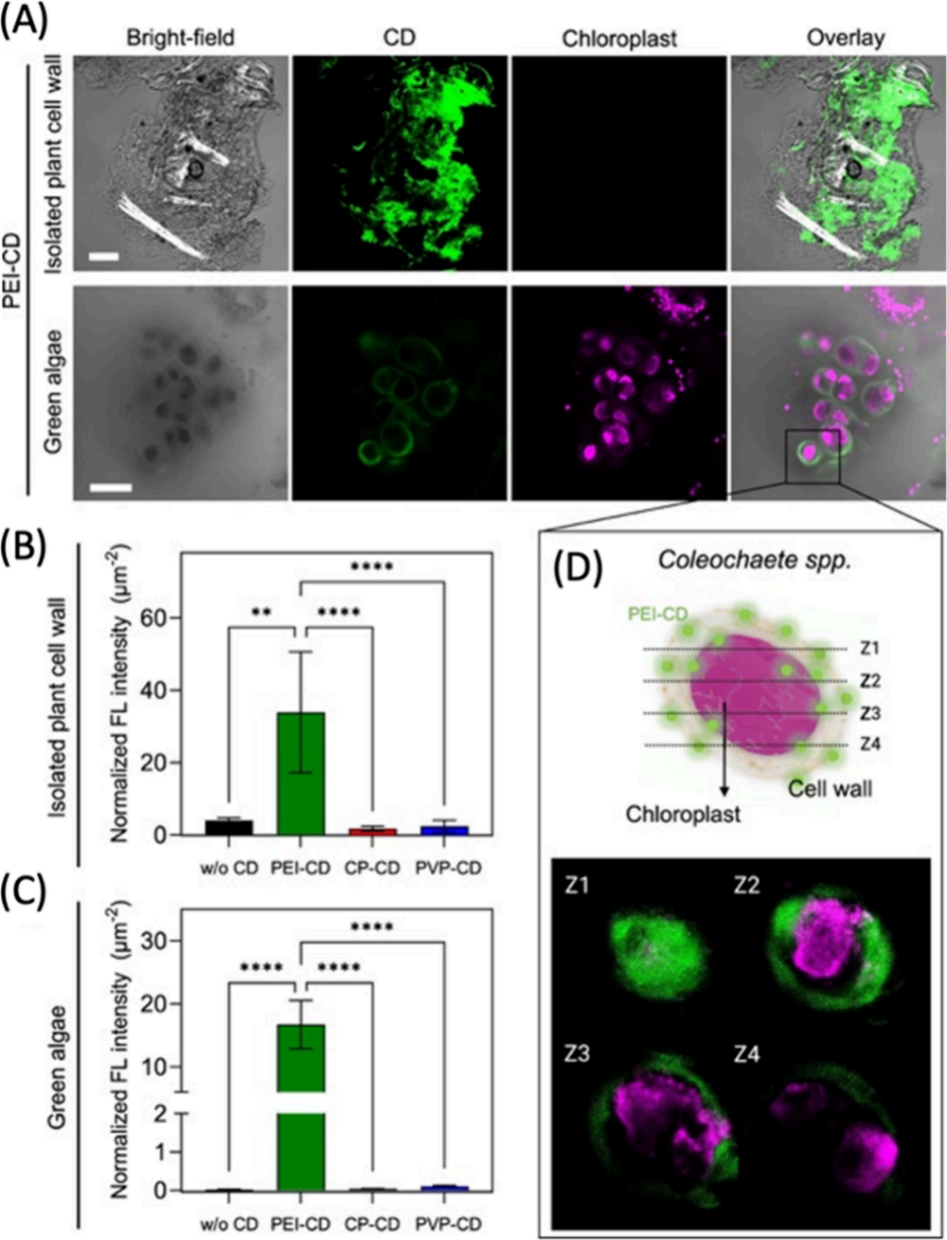
Interactions between carbon dots and the cell walls of native plants and algae. (A) Confocal images showing the cell walls isolated from *Arabidopsis* plant leaves and live green algae (*Coleochaete*). The scale bar in the images is 100 μm. (B) Comparative analysis of CD fluorescence intensity, which was normalized by the imaging area, for the cell walls of native plants and algae based on multiple confocal images (*n* = 3–9). Different letters in the graph represent statistically significant differences, as determined by one-way ANOVA and Tukey test (** *p* < 0.01, **** *p* < 0.0001). (C) The z-stack images depict the binding of PEI-CDs to the cell wall and membrane of algae, with chloroplasts shown in magenta. Adapted from ref (1056). Copyright 2023 American Chemical Society.

In a recent article, Jeon et al. explored this interaction by creating CDs with varying surface charges, specifically designed to target the cell walls of plants and algae.^1056^ These included CDs coated with polyethyleneimine (PEI-CD), carboxylated polyethyleneimine (CP-CD), and polyvinylpyrrolidone (PVP-CD). Using the intrinsic fluorescence properties of CDs as fluorescent probes, this study delved into how these different CDs interact with both model and native cell walls of plants and algae. Interestingly, only the positively charged PEI-CDs had a strong affinity for the native cell walls of plants and algae, in stark contrast to the negligible interaction observed with CDs that were either neutral or negatively charged (Figure 27). This finding was further elaborated through chemical interaction studies, where PEI-CDs demonstrated a significantly stronger binding affinity to pectin compared to cellulose in model cell walls, given the ionic interactions between the charged groups of the CDs and the pectin’s carboxylic acid groups. Further analysis showed that increasing the surface charge density of the positively charged CDs enhanced their interactions with plant cell walls. This research not only underscores the potential of using specifically designed CNM fluorescent probes for imaging cell walls, but also sheds light on the importance of charge and electrostatics in nanoparticle–plant interactions.

Besides cell walls, chloroplast stands out as a key focus in plant imaging due to its vital role in numerous plant metabolic pathways, including the conversion of light energy into sugars that power plant growth and the regulation of plant yields.^1057,1058^ Additionally, chloroplasts are becoming increasingly recognized as promising targets for gene editing. Given the high polyploidy of the plastid genome, which is the condition of having multiple sets of chromosomes, chloroplast transformation can lead to exceptionally high levels of protein production by introducing thousands of foreign gene copies per cell.^1059^ This potential, coupled with a reduced risk of mammalian viral contamination and the chloroplasts’ ability to correctly fold human proteins, positions chloroplasts as ideal candidates for synthesizing human therapeutics.^1060^ The convergence of these unique attributes makes plant chloroplasts a particularly intriguing subject for detailed study and imaging.

**Figure 28:**
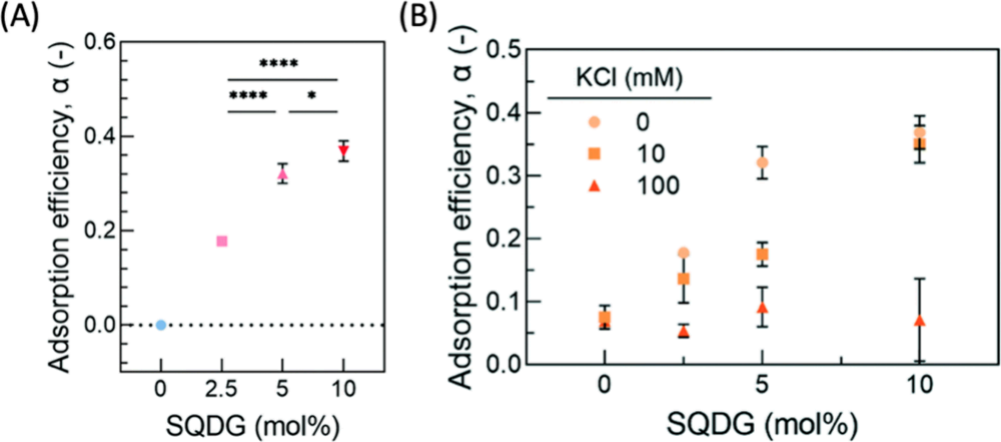
(A) Evaluation of the adsorption efficiency of PEI-CNDs on bilayers containing 0–10% SQDG. The adsorption efficiency was determined as zero for the 0% SQDG bilayer since the exposure to PEI-CNDs did not result in detectable frequency changes. (B) Adsorption efficiencies of PEI-CNDs on 0–10% SQDG containing bilayers under 0–100 mM KCl, showing effects of increasing ionic conditions. Adapted from ref (1061). Copyright 2022 American Chemical Society.

To investigate the interactions of nanomaterials with chloroplasts, Kim et al. utilized CDs as fluorescent probes, focusing on positively charged PEI-CDs and negatively charged CP-CDs and PVP-CDs.^1061^ This study revealed the essential role of sulfolipid (SQDG), a negatively charged lipid in chloroplast membranes, in influencing these interactions. The presence of higher SQDG levels in chloroplast membranes significantly increased the electrostatic attraction with positively charged PEI-CDs (Figure 28A). This novel imaging tool also demonstrated that the ionic strength of the environment plays a crucial role in modulating this attraction, with increased ionic conditions leading to reduced CD adsorption on SQDG model membranes (Figure 28B). Furthermore, strong CD–chloroplast membrane interactions could inhibit further adsorption due to particle–particle repulsion, a phenomenon known as the surface exclusion effect. These findings elucidate the complex interplay of forces in nanomaterial–biological membrane interactions, and underscore the utility of CDs as versatile fluorescent probes to image these interactions.

In another study, instead of using isolated chloroplasts as a model system, Kwak et al. utilized mature *E. sativa*, *N. officinale*, *N. tabacum*, and *S. oleracea* plants, as well as *A. thaliana* mesophyll protoplasts (plant cells in solution with their cell walls digested), and employed SWCNTs both as carriers for plasmid DNA and as fluorescent probes for chloroplast imaging.^1062^ These SWCNTs, designed using the lipid exchange envelope penetration (LEEP) mechanism, effectively penetrated plant cell walls and membranes, including the double lipid bilayers of chloroplasts,^1063^ enabling the direct delivery of genetic material to these organelles in various plant species. The fluorescent properties of SWCNTs facilitated real-time tracking and visualization of this gene delivery process, providing critical evidence of their localization within chloroplasts and the subsequent gene expression. The dual functionality of SWCNTs marks a substantial advancement in plant biotechnology, offering a more precise and efficient method for studying chloroplast functions and enhancing capabilities in genetic engineering for crop improvement.

While the probes discussed above have been promising plant bioimaging agents, their interaction with plant structures largely depends on size, electrostatic forces, and intermolecular interactions. This dependence, though beneficial in certain scenarios, can also be a source of variability and limitation. Factors such as pH fluctuations and variations in salt concentration within the plant’s internal environment can largely alter the efficacy of these probes. As the ultimate goal of bioimaging is to achieve precise, reliable, and consistent visualization of cellular processes, overcoming these limitations is important. To enhance the specificity and robustness of these probes under varying environmental conditions, a promising approach lies in the functionalization of CNMs with molecular recognition elements.^1064,1065^ This strategy involves the incorporation of specific biomolecules or chemical groups onto the surface of the nanomaterials, transforming them into smart probes capable of selectively targeting and binding the specific imaging targets within plant cells.

**Figure 29:**
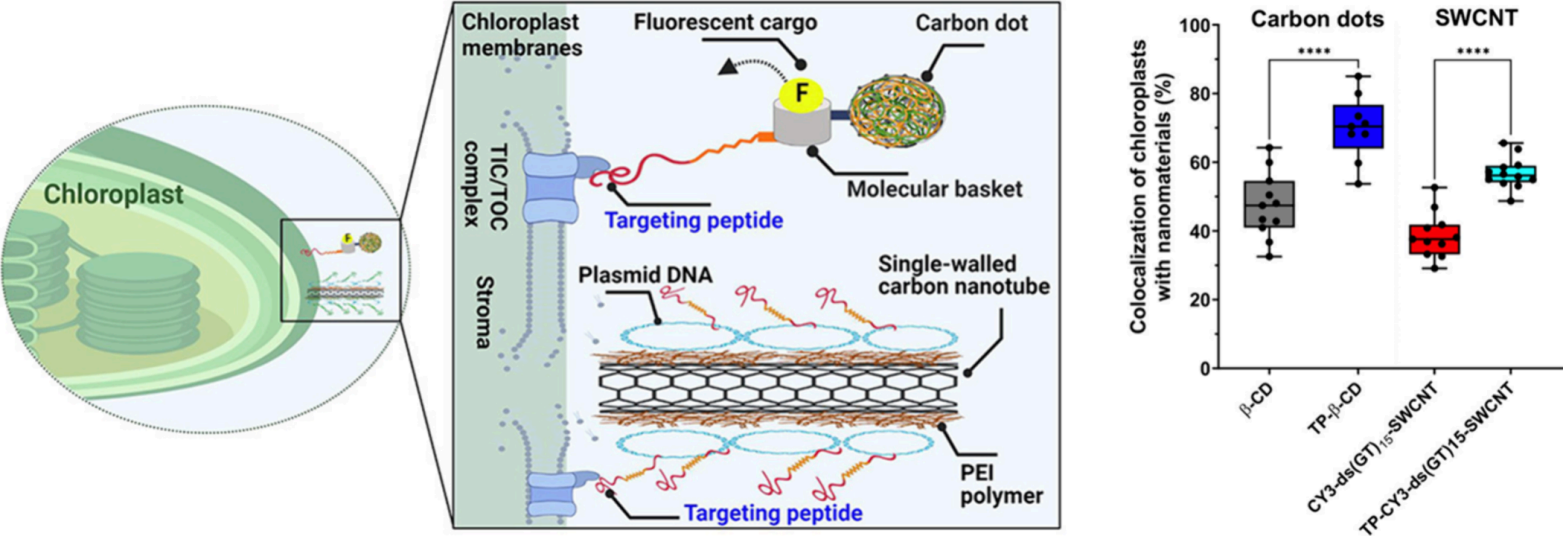
(Left) Schematics of CNM targeting to plant chloroplasts. (Right) The analysis of colocalization in nanostructures revealed a notably increased proportion of chloroplasts containing targeted nanomaterials, in contrast to the control group lacking TPs. Statistical evaluation was performed using one-way ANOVA and post hoc Tukey’s test, with a sample size of 7 to 12, yielding a highly significant result with *p* < 0.0001. Adapted from ref (1066) Copyright 2022 American Chemical Society.

For this, Santana et al. developed targeted CNM probes for chloroplasts.^1066^ Their design includes β-cyclodextrin-CDs and DNA-coated SWCNTs functionalized with a chloroplast targeting peptide (TP). This peptide, derived from the Rubisco small subunit 1A, selectively binds to protein translocation outer channels (TOC) on the chloroplast membrane, enhancing the efficiency of targeted delivery into chloroplasts. Using these probes, the quality and specificity of fluorescent probes for imaging chloroplasts have been substantially improved. This enhancement is evidenced by the notable increase in colocalization rates. For TP-β-CDs with chloroplasts (Figure 29), the colocalization rates have significantly risen to 70.0 ± 9.46%, compared to 47.4 ± 9.57% for β-CDs that are not targeted. In a similar vein, the colocalization rates for TP-SWCNTs have escalated to 56.9 ± 4.58%, a marked improvement from the 38.7 ± 6.69% observed for untargeted SWCNTs.

Besides targeted probes for chloroplasts, targeted fluorescent probes for plant mitochondria are also impactful, since mitochondria are involved in key cellular processes, such as energy production and regulation of metabolic pathways.^1067^ Developing specific fluorescent probes for plant mitochondria can provide deeper insights into their unique functions and dynamics within plant cells.

An example here is the innovative use of SWCNT-polymer hybrids for imaging plant mitochondria.^1068^ These hybrids, specifically designed for mitochondrial targeting, consist of a polymethacrylate maleimide (PM) layer noncovalently adsorbed on the SWCNT surface. This layer offers high adaptability for functionalization and can undergo covalent conjugation with thiol-rich compounds, including the Cytcox peptide (Cyt) that targets mitochondria, and the KH9 cationic peptide that enhances electrostatic interactions for binding. Law et al. evaluated the effectiveness of these SWCNT nanocarriers in delivering to plant mitochondria.^1068^ They created a fluorescently labeled variant by conjugating SWCNT-PM-CytKH9 with the DyLight488 dye. This complex was then applied to the root cells of *Arabidopsis thaliana* through a vacuum/pressure infiltration process. Their observations showed a distinct colocalization of the DyLight488-labeled SWCNT-PM-CytKH9 with MitoTracker-stained mitochondria in the infiltrated cells. This contrasted with the control groups: samples without vacuum infiltration displayed the SWCNT-PM-CytKH9 primarily on the root surface, while those treated with only DyLight488-labeled KH9 exhibited minimal fluorescence, highlighting the targeted delivery and localization of the SWCNT-PM-CytKH9 probes within the mitochondria, enabled by the imaging capabilities of SWCNTs in plant tissues.

### 5.3 Biosensing Applications of Fluorescent CNMs

#### 5.3.1 Biomedically Relevant Sensing

Sensing in biology can facilitate breakthrough insights into the inner workings of numerous biological processes, spanning broad fields including cancer research, metabolomics, and neurobiology. Classically, biosensing relies on genetically encoded proteins that operate by modulating the intensities or fluorescence lifetimes of engineered fluorescent transgenes, expressed in cells, tissues, or organisms via genetic approaches. While genetically encoded sensors remain the predominant technology in biology, synthetic, nonprotein-based probes have made important contributions. In this regard, CNMs have made contributions for sensing of biomolecules, including those that target small signaling molecules, such as neurotransmitters and metabolites,^619,1069−1071^ as well as large macromolecular complexes,^1072,1073^ including proteins, oligonucleotides, and organelles^1074^ and whole cellular complexes, such as viruses and bacteria.^1075,1076^ In this section, we review utilization of CNMs for biosensing within the context of applications in biological research which include, among others, their utility in advancing basic biomedical research, as well as translational uses in analytics and diagnostics.

Optical biosensors are an amalgam of two primary components: the fluorophore and the molecular recognition motif. Biosensor development is concerned with identifying or rationally synthesizing molecular recognition motifs, and successfully conjugating these motifs to the fluorescent molecule. In most designs, conformational changes, or perturbations of local chemical environment elicited by the recognition motif modulate the fluorescence of the reporter, which can serve as a readout for molecular recognition. CNM photoluminescence can be sensitized to its local chemical environment through strategies that employ covalent or noncovalent conjugation of the nanomaterials to molecular recognition motifs. In principle, a molecular recognition event in proximity to the nanomaterial can induce local perturbations in the electrical, chemical, or photophysical properties of the nanomaterial that can be read out for sensing. Here, we emphasize modulation of photophysical properties that facilitate sensing within the context of fluorescence imaging (i.e., optical biosensing) and do not discuss literature related to sensing through other modalities (e.g., electrochemical). From among the family of CNMs included in this review, SWCNTs are the predominant materials employed for optical biosensing because their photoluminescence emanates from superficial excitons that can be easily perturbed by proximally located molecular recognition motifs. Fortuitously, SWCNTs fluoresce in the NIR region of the spectrum has properties that facilitate imaging in biological specimens. Some applications of GO-related CNMs as sensors involve the use of these materials in conjunction with other optical probes through which the sensing occurs and are beyond the scope of this review. In such cases, the graphene nanomaterials are used as a platform to bind and quench fluorescently labeled oligonucleotides^1077−1079^ or probes,^1080^ which are then turned on upon binding their target analytes. Similarly, GO-type CNMs have also been used to enhance other forms of fluorescent nanomaterials, such as up-conversion nanoparticles; however, the fluorescent output here is from the up-conversion nanoparticle and not the GO nanomaterial itself.^1081^

In subsequent sections, we review applications of SWCNTs, and when appropriate other CNMs, for sensing in biological research. For additional material on SWCNT-based sensors, readers are invited to refer to a recent review by Ackermann et al.^1082^

##### 5.3.1.1 Neurotransmitters and Neuromodulators

One of the most productive uses of CNMs for sensing has been in the field of neuroscience, where SWCNT biosensors have made significant contributions to our understanding of the molecular and cellular neurobiology of a class of neurons that release catecholamines. ssDNA-functionalized SWCNTs (ssDNA@SWNCT) have been particularly fruitful in this field. To understand their utility, it is important to appreciate the basics of the biology toward which they are applied and how several features of the biological systems to which they are targeted have contributed to their success.

**Figure 30:**
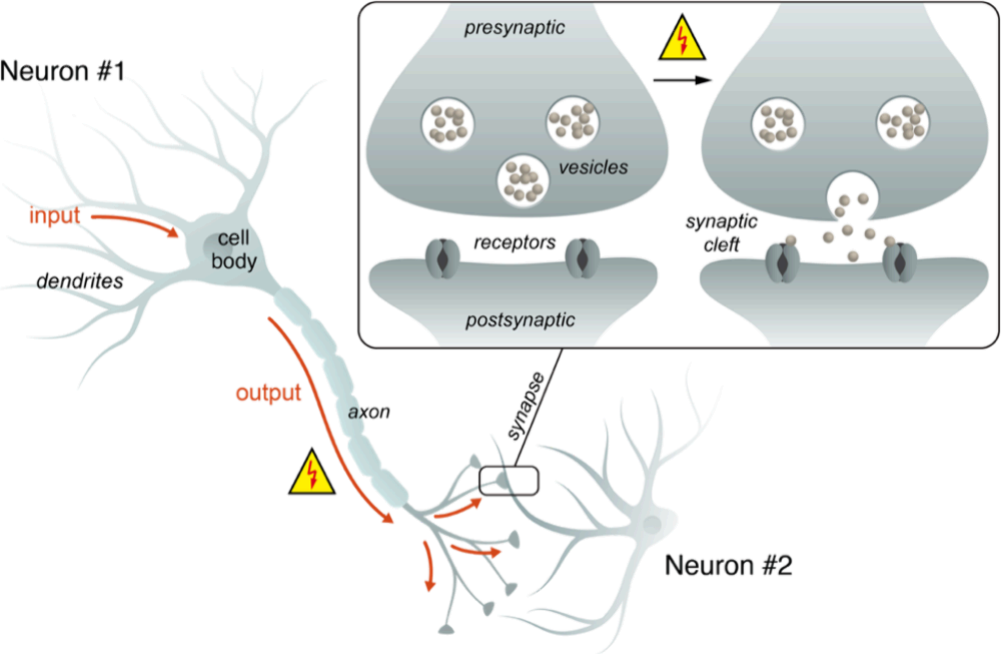
Neurons receive input through dendrites, integrate this input at the cell body, and send information out to neighboring neurons through their axons. Communication occurs via interfaces known as chemical synapses that convert electrical signals in axons to chemical signals that are released via vesicular exocytosis (inset).

Nerve cells (neurons) are the building blocks of the nervous system. One of the hallmark properties of neurons that underpins their function is their ability to communicate with each other. This communication is, in most instances, mediated by chemical signaling molecules that are released from one neuron and diffuse through extracellular space (ECS) to travel to and activate receptors on neighboring neurons (Figure 30). Neurons use several dozen types of chemicals to derive this communication, with each neuron typically releasing just one type of neurochemical. Regardless of the diversity in chemical matter, most of the signaling between neurons is highly stereotypical. Each neuron synthesizes and packages one or just a few of these chemical signaling molecules into small packets (quanta) of vesicles, which are aggregated into cellular locations called synapses (Figure 30). A sophisticated protein machinery then orchestrates the release of these chemicals from synapses in response to neuronal electrical activity, where the molecules diffuse in the ECS to activate receptors on postsynaptic partners. An array of complex molecular processes ensure that the release of these chemical cues is highly confined both spatially and temporally. The spatial and temporal precision of these chemical cues makes them amenable for detection with a fluorescent turn-on sensor because neurochemical signals go from low-to-high in a short period of time, within a relatively small spatial domain. In other words, they are both spatially and temporally specific. As a consequence, a biosensor that is strategically localized to the ECS inside synaptic clefts or in close proximity to them is likely to detect this signal if it has the appropriate kinetics and sensitivity.

**Figure 31:**
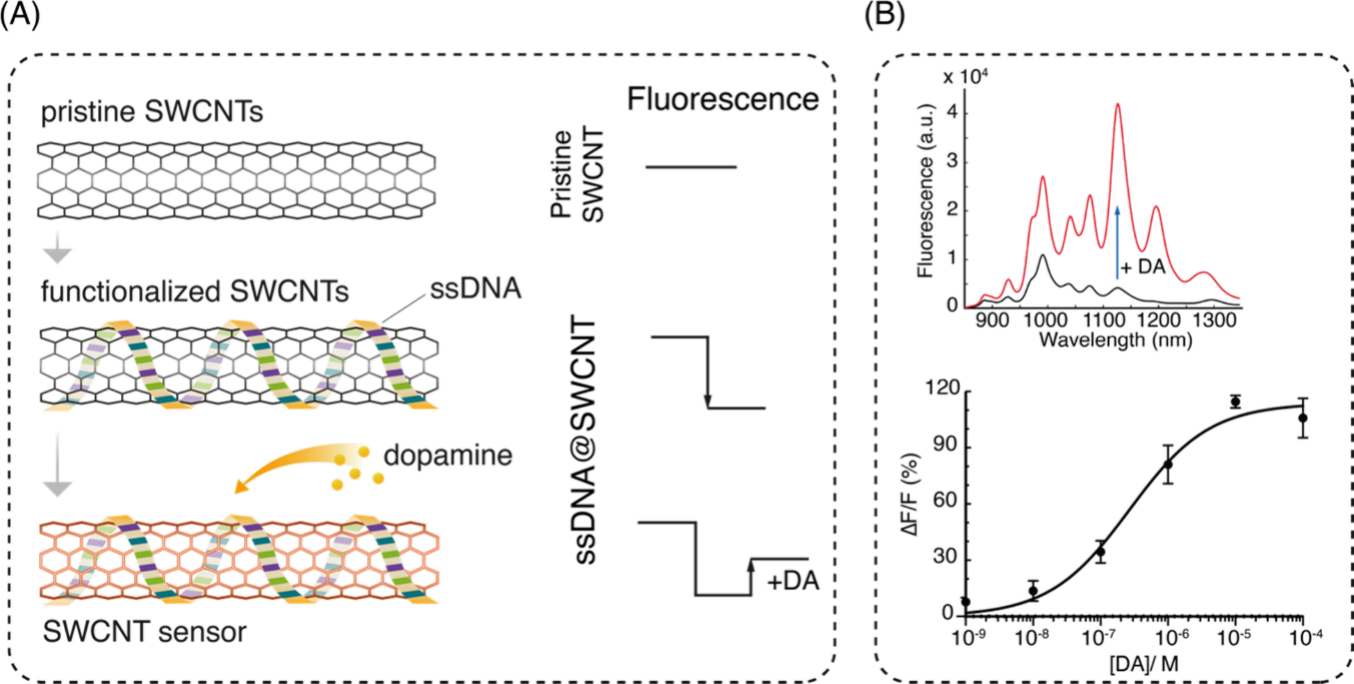
SWCNT-based sensors for the catecholamine dopamine. (A) Pristine nanotubes surface functionalized with a short, 12-mer oligonucleotide sequence (GT)_6_ exhibits a strong turn-on sensitivity to dopamine. The ssDNA coat affords colloidal stability and tiles the surface of nanotubes, creating binding pockets for dopamine molecular recognition. Ligand binding is transduced via modulation of the nanotube’s fluorescence emission. To the right: current model of how the sensor is thought to work. Dispersions of ssDNA@SWCNT exhibit quenched fluorescence, which is partially rescued by the addition of dopamine (+DA). (B) Top: Fluorescence spectra of a polydisperse nanotube colloid before (black) and after (red) addition of 10 μM dopamine (+DA) in solution. Bottom: Dose response curve for surface immobilized nanotubes show half maximal response (EC_50_) of ∼250 nM.

One class of chemicals that neurons employ for communication are referred to as catecholamines. Catecholamines fall within the family of monoamine neuromodulatory neurotransmitters and include molecules such as dopamine and norepinephrine that play important roles in learning, motivation, and motor control. SWCNT-based catecholamine sensors have so far been some of the most successful and well-studied.^619,1083^ These sensors employ a conjugation between ssDNA and SWCNT, which create ssDNA@SWCNT bionanomaterials that exhibit strong fluorescence turn-on response to catecholamines. Short (GT)*_n_* (*n* = 6–15) sequences are typically used for sensor assembly. Conjugation of the ssDNA to the SWCNT is achieved through noncovalent self-assembly, in which π-stacking of ssDNA nucleobases on the graphitic lattice of SWCNTs electrostatically pins the DNA to the nanotube surface. The negative charge of the phosphodiester backbone imparts solubility in aqueous media and is the main source of colloidal stability for ssDNA-solubilized SWCNTs.^1084^ In addition to offering a stable colloid, the DNA tiles the surface of SWNCT, quenching the baseline fluorescence of the SWCNT and creating a rich surface topology with unique binding pockets for the molecular recognition of catecholamines (Figure 31). The binding of catecholamines to recognition sites on ssDNA@SWCNT surfaces results in a strong turn-on of the quenched baseline fluorescence of the nanotube. The mechanism behind the fluorescence turn-on is still being investigated. One proposed mechanism posits that molecular interaction between catechol’s diol motif and phosphate groups on ssDNA lead to ssDNA conformational change.^1085^ Another study proposes that catechols perturb the electrochemical surface potential imprinted on the SWCNTs by the adsorbed ssDNA.^1086^ (GT)_*n*_-rich ssDNA sequences appear to provide the best signal-to-noise for catecholamine detection and molecular dynamic simulations have offered insights into the role that hydrogen bonding and π-stacking play in analyte binding and molecular recognition.^1085,1086^ Catecholamine binding is thought to be followed by facilitation of radiative recombination of excitons on nanotube surfaces.

One of the first successful demonstrations of the use of ssDNA@SWCNT for sensing and imaging catecholamine release from cells involved the detection of dopamine release from pheochromocytoma (PC12) cells.^1087^ Although non-neuronal, PC12 cells package and release catecholamines (primarily dopamine) from dense core vesicles. In this study, pheochromocytoma cells were cultured on a nanofilm layer made from (GT)_15_ ssDNA functionalized SWCNTs ((GT)_15_@SWCNT) deposited on a glass coverslip and then passivated with polylysine. (GT)_15_@SWCNT exhibited good turn-on sensitivity to dopamine in solution phase, and this sensitivity is retained when the sensors are deposited onto a solid substrate on which cells can be cultured. Cultured PC12 cells were stimulated with a high concentration potassium chloride (KCl) solution to evoke dopamine release. This allowed detection of dopamine released from cells, in which the turn-on signal was localized to the cellular periphery of the cells being imaged. This study presented an important conceptual advance, particularly in employing fluorescent CNMs as solid-state substrates on which cells can be cultured, and with which biochemical activity from the same cells can be detected. However, reliance on KCl for cellular stimulation suggested that the study lacked the appropriate signal-to-noise ratio to report catecholamine release with better temporal and spatial specificity.

**Figure 32:**
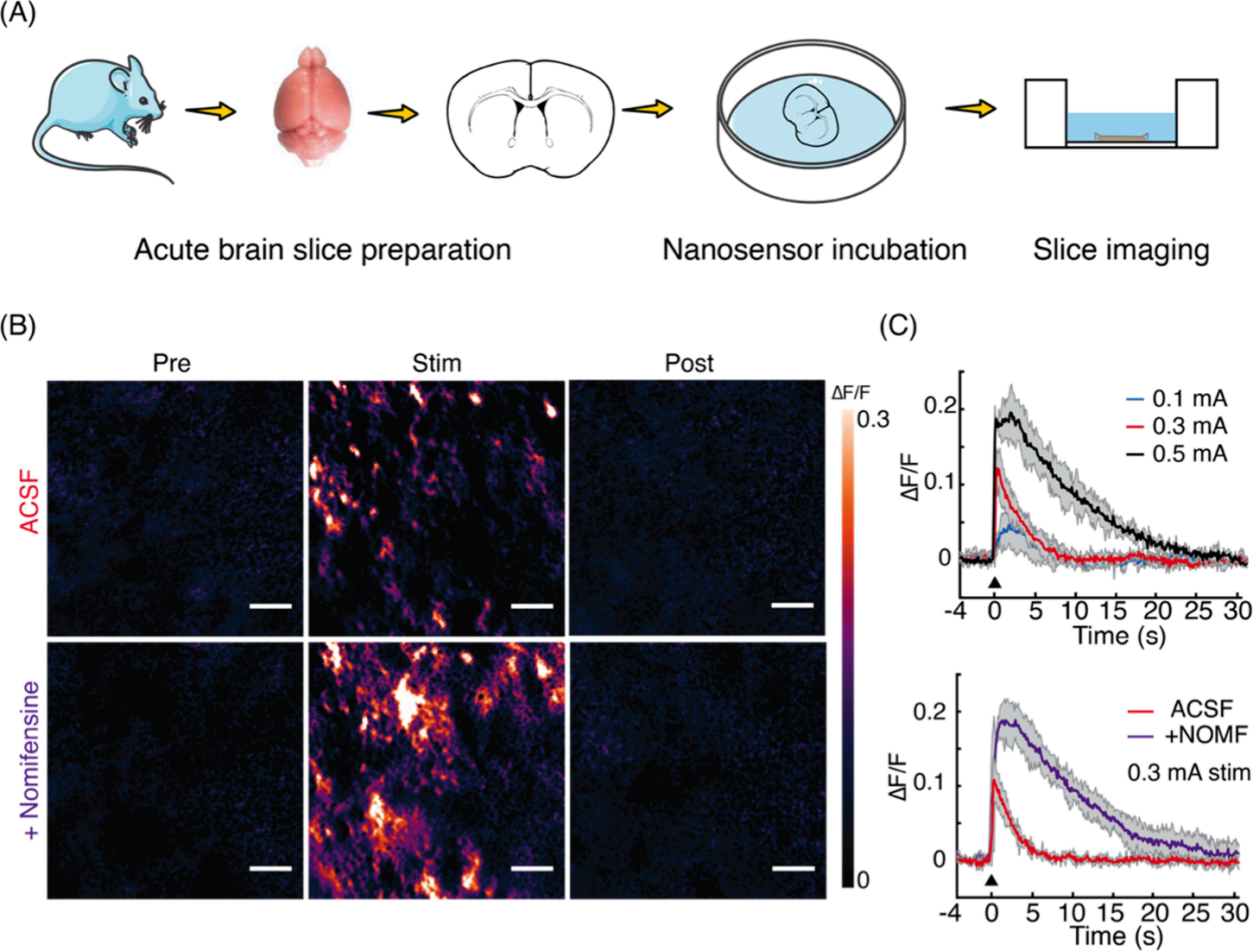
Imaging of dopamine release from brain slice tissue. (A) Brain slices are incubated in solutions that contain dopamine nanosensors. This process delivers the nanosensors into the brain slice through passive diffusion. (B) Electrical stimulation derives dopamine release, which are detected as hotspots by dopamine nanosensors. Application of nomifensine (bottom row) delays the clearance kinetics of dopamine and increases spatial extent of dopamine diffusion relative to standard imaging buffer (ACSF) (scale bar = 10 μm). (C) Spatially averaged traces of nanosensor fluorescence transients under various stimulation paradigms (top) and pharmacological perturbations (bottom with nomifensine, +NOMF). Reproduced with permission from ref (1088). Copyright 2019 American Association for the Advancement of Science.

The first application of SWCNT-based catecholamine sensors in neurons involved imaging dopamine release in striatum in acute brain slice preparations obtained from mice.^1088^ The striatum is an important anatomical brain region that receives strong innervation by dopamine neuron axons. In this study, (GT)_6_ functionalized SWCNTs ((GT)_6_@SWCNT) were applied to 300 μm slices of brain tissue by incubating the tissue in a dilute concentration of the nanosensors (Figure 32A). The nanosensors diffused into the tissue, forming a layer of uniform coat on the slice at penetration depths of up to 20 μm. Dopamine release from brain tissue was elicited via electrical stimulation, which enabled sub-second scale temporal precision for signal detection. In addition, optogenetic stimulation was also employed, which involves the use of light sensitive proteins to stimulate dopamine release, offering both temporal and cellular specificity for evoking dopamine release. In both stimulation paradigms, the SWCNT sensors were able to record the release and diffusion of dopamine from axonal terminals at video frame rates (Figure 32B,C). This study demonstrated that dopamine release is organized as hotspots in the striatum, the brain region where imaging was being performed. The authors demonstrate that application of pharmacology that delays clearance of dopamine from tissue also delays the temporal profile of the signal decay from the nanosensors, demonstrating that ssDNA@SWCNT conjugates possess favorable kinetic properties for fast imaging of neurochemical release and can recapitulate pharmacologic perturbations. Although this study enabled temporally precise imaging of dopamine from brain tissue, it lacked the spatial resolution to assign the visualized dopamine release hotspots to specific chemical synapses.

**Figure 33:**
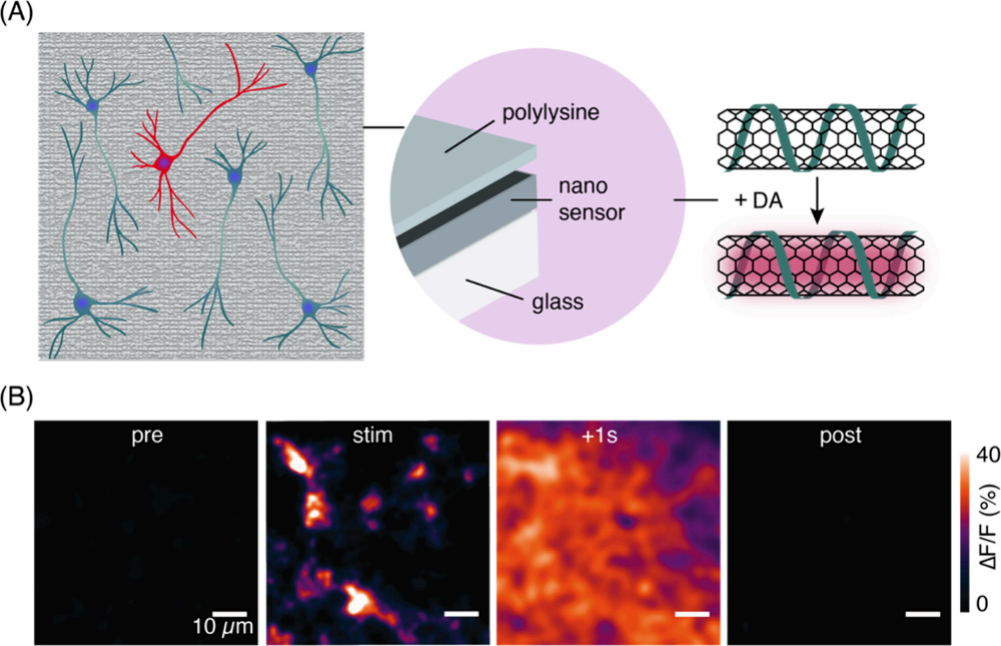
(A) Composite nanofilm strategy for culturing primary dopaminergic neurons. Dopamine neurons are cultured on fluorescent and dopamine sensitive substrate produced from drop casting a solution of ssDNA@SWCNT conjugates on glass surfaces. (B) Dopamine release evoked by field stimulation modulates the fluorescence of the nanosensor layer, which is recorded as a “hotspot” of activity (a cluster of pixels that exhibit highly correlated temporal behavior). Images show temporal evolution of signal. Reproduced with permission from ref (1090). Copyright 2022 *Elife* under CC BY 4.0.

Two additional recent studies have demonstrated the utility of SWCNT-based sensors for imaging dopamine release with the spatial specificity of a single chemical synapse. These studies involve the use of composite nanofilm substrates made from fluorescent and dopamine sensitive ssDNA@SWCNT conjugates, and employ primary dopaminergic neurons that are grown on such composite nanofilms (Figure 33). Using this method, Elizarova et al. demonstrate an important role for a protein called MUNC13 in organizing dopamine release by using KCl and electrical field stimulation of neurons to evoke dopamine release.^1089^ Using optogenetic stimulation, Bulumulla et al. show spatiotemporal dopamine release and diffusion from single chemical synapses with the sensitivity of single release events, a feat that had not been achieved with any type of biosensor before (Figure 33).^1090^ With this tool, the authors explore various facets of dopamine neurobiology that elude conventional methods of inquiry, including exploring molecular determinants of release, and elucidating less well understood phenomena such as release of dopamine from dendritic process. SWCNT-based catecholamine sensors for dopamine can also be used for investigating norepinephrine, another important molecule, although demonstration of this in real biological contexts is still lacking. SWCNT-based optical sensors for catecholamines remain some of the most well studied and extensively used sensors from the CNM family. Importantly, catecholamines play critical roles in various facets of brain function, and their aberrations are implicated in several neurological and psychiatric diseases. Therefore, ssDNA@SWCNT catecholamine optical nanosensors are poised to be an important tool within the neuroscientist’s toolkit for continued explorative research in these fields.

Serotonin (5-hydroxytryptamine, 5-HT) is an important modulatory neurotransmitter in the brain and the enteric nervous system. Cell bodies of serotonergic neurons are located in the raphe nuclei, a region in the brain stem, and project to innervate all the major regions of the brain, including the cortex. Consequently, serotonin regulates a wide range of brain functions that affect broad facets of behavior, including mood, learning, and cognition. SWCNT-based nanosensors for 5-HT have been developed and their utility have been demonstrated in brain tissue and in cells in two studies. In the first study, Jeong et al. developed a sensor for 5-HT from conjugation of ssDNA on SWCNTs in a manner similar to catecholamine sensors.^1091^ These sensors were developed by high-throughput screening coupled to a selective enrichment strategy, in which a large random library of initial oligonucleotide sequences was conjugated to SWCNTs, and selectively enriched for their ability to bind to 5-HT. The technique, named SELEC by the authors, made an important advancement in its approach to identify ssDNA sequences that can serve as recognition motifs for a specific analyte in a semirational manner and with an improved throughput, which contrasts sharply against the low throughput screening approaches that have generally been the norm in the field. Using this method, the authors identify a sequence with improved selectivity for 5-HT over catecholamines. To demonstrate the utility of the sensors, the authors use them for detecting exogenously applied 5-HT in acute brain slices prepared from mice. In the second study, Dinarvand et al. noncovalently conjugated a previously reported DNA aptamer to SWCNTs as a molecular recognition motif.^1092^ These 5-HT aptamers, when conjugated to SWCNTs, serve both as the recognition element and keep the nanotubes in stable colloidal dispersions. The study demonstrates the use of these sensors for detecting endogenous 5-HT release from platelets.

We highlight these two studies here because both seek to address a general challenge in the development of biosensors for molecules of interest using CNM scaffolds. Development of such biosensors has historically relied on serendipitous discoveries or low throughout screens, and a rational design strategy has been sorely lacking. Jeong et al. and Dinarvand et al. sought to ameliorate this drawback by using two different strategies: high-throughput screening or relying on a pre-existing molecular recognition motif (an aptamer in this case). Although important as conceptual advances, the broader applicability of both approaches remains to be seen. For example, it is still not clear if the SELEC strategy reported by Jeong et al. can reliably generate selective sensors for a broader class of analytes. On the other hand, while aptamers have often been touted as molecular recognition motifs that can be modularly coupled to CNMs for biosensor development, the study by Dinarvand et al. fails to fully explore alternative aptamer-CNM conjugation strategies beyond a simple sonication step. It is therefore not clear to what extent the aptamer’s solution-phase secondary structure is retained once it is coupled to SWCNTs via probe tip sonication, making it difficult to assess if the reported molecular recognition indeed arises from the aptamer’s secondary structure. Critically, the selectivity of the sensors for 5-HT over catecholamines from both of the studies we highlighted here needs improvement. However, with carefully designed experiments and appropriate controls, both sensors have the potential to become useful tools for the study of serotonin neurobiology. Importantly, imaging of endogenous 5-HT release from neurons with these probes has not been demonstrated, and this remains an important next use case for these technologies.

A recent report has expanded the use of SWCNT-based neurochemical sensing from small molecules to neuropeptides by employing a rational approach for sensor synthesis.^1093^ This approach eschews the low throughput and tedious screening strategies that are used to identify molecular recognition motifs for target analytes, and instead uses a rational design strategy for developing a sensor for a neuromodulator called oxytocin. Oxytocin is an important neuropeptidergic hormone that is released from a small cluster of neurons. In the mammalian brain, oxytocin plays an evolutionarily conserved role in regulating important aspects of social behavior, particularly in establishing and maintaining social bonds, and regulating maternal care. In the study, an oxytocin receptor peptide fragment is used as the molecular recognition motif, where it is covalently linked to the surface of SWCNT.^1093^ The covalently functionalized SWCNT scaffold is further passivated and solubilized noncovalently with ssDNA. The hybrid covalent and noncovalent approach offers a unique strategy that scaffolds the nanotube surface with the molecular recognition and solubilizing motifs serially. This sensor was demonstrated to be selective for oxytocin over vasopressin, a close molecular analogue of oxytocin that is often difficult to distinguish from oxytocin using commonly available assays. The probe was further validated in *in vitro* assays and demonstrated to have affinities for oxytocin (reported as *K*_d_) of ∼6 μM. When employed in mice acute brain slice experiments, the probe enabled visualization of oxytocin dynamics evoked by the electrical stimulation of the paraventricular nucleus of the hypothalamus, a brain region that is enriched in oxytocin releasing neurons.

##### 5.3.1.2 Proteins, Oligonucleotides, and Other Biomolecules

SWCNTs can be conceptualized as cylindrical rolled sheets of graphene, and exhibit photoemission that is highly sensitive to the direction and length of the rollup vector that maps the nanocrystalline lattice of the SWCNT onto an imaginary 2D graphitic sheet (Figure 7). SWCNT photoemission therefore displays complex spectra that are a convolution of fluorescent spectra from several unique emitting species, a reflection of the geometric diversity with which flat a graphitic lattice can be rolled into cylindrical tubes. While this convoluted photoemission spectra can often be a disadvantage when pure, single emitter species of nanotubes are sought, the diversity and spectral complexity can also be leveraged for biosensor development. In particular, the location of fluorescence peaks (peak position) for each SWCNT emitter is sensitive to the dielectric environment of the surface of the nanotube. Therefore, solvatochromic shifts driven by modulations in surface dielectric properties can be leveraged for the development of sensors for biomolecules, including oligonucleotides and proteins.^1094−1097^

One such biosensor that has been developed for protein targets is for albumin, one of the most abundant proteins in the human body. A high concentration of albumin in urine is indicative of a pathologic state. In one study, polycarbodiimide (PCD) polymers with carboxylic acid functional groups, which have an affinity for albumin, were used in conjugation with SWCNTs.^1098^ SWCNTs were noncovalently functionalized with PCD polymers. The collapse of PCD on the surface of nanotubes offered colloidal stability and served as a molecular recognition motif. When exposed to albumin, PCD functionalized-SWCNTs undergo a hypsochromic (blue) shift in their emission spectra, which served as a readout for albumin biosensing. It is thought that the PCD headgroup mimics albumin binding fatty acids and serves as the basis for molecular recognition. In contrast, nonspecific protein binding by transferrin and γ-globulins drives a bathochromic shift in nanotube fluorescence or no shift at all. This is offered as evidence of specificity of the response to albumin. The choice of transferrin and γ-globulins as the only potentially interfering proteins limits the validation of this sensor and constrains the scope of its use to urine samples. Therefore, the applicability of this sensor would require a more rigorous test of specificity that is tailored toward the biological samples under investigation. Moreover, the sensor exhibits a maximal response of ∼1.5 nm over time scales of ∼20 min, suggesting that detection of smaller signals that occur over faster time scales under dynamic conditions could be challenging. The authors use a hydrogel encapsulation strategy to demonstrate the ease and utility for biosensing albumin, and propose the method for diagnosing microalbuminuria in low resource settings.

SWCNT emission wavelength-shift is not the only sensing modality of proteins, and intensiometric sensing of proteins has also been demonstrated.^1097,1099^ In two studies, Bisker and colleagues used PEG-phospholipid functionalized SWCNTs for sensing the blood protein fibrinogen. *In vitro*, the sensor exhibited a dose-dependent fluorescence quenching in response to fibrinogen. Fragment based assays revealed the binding emanates from an interaction between the D-domain of the fibrinogen protein and the PEG-phospholipid corona, and not the E-domain nor fibrinopeptide sequences. In a subsequent study, the authors used the sensor for monitoring the dynamics of the coagulation cascade to visualize fibrin and thrombin-mediated blood clot formation demonstrating the ability of the probe for monitoring active biochemical processes.^1099^

Early diagnosis of cancer is a critical factor in the longer-term survival of patients, and several SWCNT-based biosensors have been developed that target cancer biomarkers. In particular, several studies from Heller and colleagues have made important contributions to detecting cancer biomarkers from patient derived biofluids. In one such study, Williams et al. conjugate an antibody for HE4 (an ovarian cancer biomarker) to SWCNTs.^1100^ Exposure of the SWCNT-HE4 antibody conjugate to patient derived HE4 biofluids leads to a solvatochromic shift in SWCNT photoluminescence, which is used as a readout for molecular recognition. Similar strategies were employed for the detection of amyloid-beta,^1101^ the main component of amyloid plaques found in the brains of people with Alzheimer’s disease, and urokinase plasminogen activator (uPA), a prostate cancer biomarker.^1102^

During the development of optical biosensors, each analyte of interest for which an optical sensor is sought requires a unique molecular recognition motif. Consequently, developing new biosensors is a low throughput and arduous effort that relies on an iterative process of identification and synthesis of unique molecule recognition elements and their conjugation to the fluorescent material for each target of interest. Two recent reports leverage SWCNT photoemission diversity and develop an optical sensing strategy that does not rely on a one-to-one correspondence between the analyte and the recognition motif, hence seeking to improve the throughput of biosensor development.

In the first study, a library of short ssDNA oligonucleotides of varying length and sequence chemistry are conjugated to polydisperse SWCNT starting materials to generate stable colloidal dispersions.^1103^ The optical response of each ssDNA@SWCNT conjugate pair to a panel of disease biomarkers for gynecologic cancer (HE-4, CA-125 and YKL-40) is then measured. Although the optical response of any one of the ssDNA@SWCNT conjugates to HE-4, CA-125, and YKL-40 did not exhibit reliable specificity, a machine learning-based parsing of the ensemble optical modulation of the library of ssDNA@SWCNTs to patient derived samples was determined to be highly predictive for cancer relative to laboratory-generated control samples. The authors refer to this technology as a “molecular perceptron” and compare it to the ability of the olfactory system to “perceive” odors with high specificity based on a combinatorial input from many relatively nonspecific receptors. A subsequent study from the same group extends the use of “perception” strategy of sensing to SWCNTs with covalently installed color centers.^1104^ The utility of both strategies is demonstrated in solution-phase spectroscopic assays, in which both the intensities and peak positions of individual SWCNT species can be tracked. However, applications of such a “molecular perceptron” strategy in an imaging setup for the detection of spatiotemporally resolved dynamics of molecules would require combined spectroscopy and microscopy, and is yet to be demonstrated for biosensing purposes. Additionally, it is not clear the extent to which the number of starting oligonucleotide sequence in the library correlates with specificity of the sensor or the signal-to-noise ratio of detection, making direct comparisons with single oligonucleotide sensors difficult. Finally, the specificity of the strategy against a protein biomarker ensemble for a different but closely related disease is not demonstrated. Coupled with the fact that a sophisticated data analysis and parsing is a required component of the molecular perceptron pipeline, how broadly this strategy will be adopted by the broader scientific community remains to be seen.

In addition to fluorescence intensity and wavelength modulations, SWCNTs can have a stereoselective interaction with their immediate chemical environment, which can be leveraged for biomolecular sensing. Chiral species of SWCNTs exhibit handedness that is often denoted by (+) or (−) and reflects their interaction with circularly polarized light. In a recent study, enantiopure SWCNTs functionalized with resolving ssDNA sequences were demonstrated to exhibit a stereoselective modulation of their fluorescence toward amino acid enantiomers.^1105^ This difference in modulation was noted to be a consequence of the chiral nanotube species interacting in a specific way with chiral amino acid compounds. This study opens up a less explored modality of molecular recognition using SWCNT optical properties as transduction elements.

##### 5.3.1.3 Sugars, Lipids, and Other Metabolites

Besides disease markers, CNMs have been used to sense a broad range of analytes *in vivo* or in biological fluids *ex vivo.*(1106,1107) CNM-based optical recognition strategies for various important classes of metabolites, such as sugars,^1108−1111^ lipids,^1112−1114^ ascorbic acid,^1115−1117^ uric acid and urea,^1118−1120^ and mycotoxins^1121^ have been developed and improved over the past decade. Below, we discuss some of the pioneering and recent advances that have been achieved on this topic.

##### 5.3.1.3.1 Sugars

**Figure 34:**
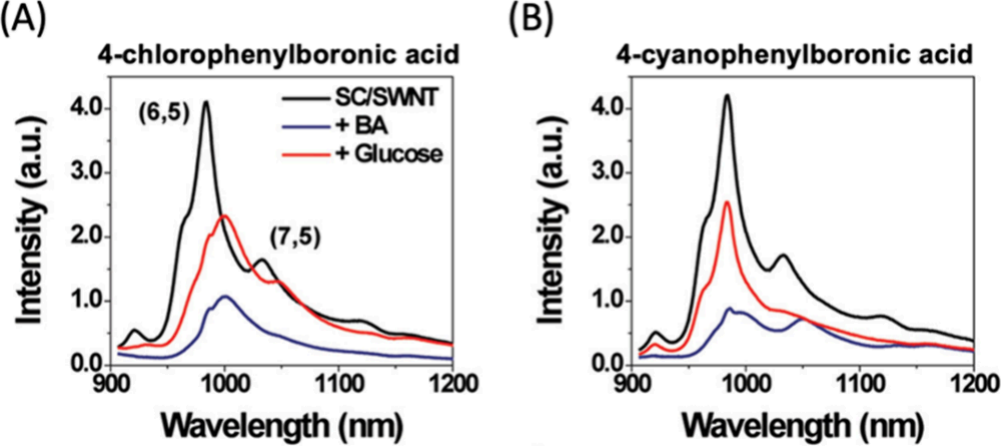
Fluorescence spectra that compare the original spectrum of SWCNTs (black), the spectrum after adding 50 mM boronic acid (blue), and the spectrum after adding 50 mM glucose (red). (A) The BA-SWCNT complexes were prepared with 4-chlorophenylboronic acid and (B) 4-cyanophenylboronic acid. Adapted from ref (1125). Copyright 2011 American Chemical Society.

Sugars are important metabolic targets that can be detected by CNMs. There have been many enzymatic and non-enzymatic efforts since the early 2000s in optical sugar sensing as described in several reviews.^1122−1124^ Earlier non-enzymatic efforts focused on the non-covalent and covalent attachment of boronic acid varieties to SWCNTs for glucose-specific fluorescence intensity and wavelength modulation with a detection limit of around 5 mM.^1109,1110^ Here, boronic acid is used to quench the fluorescence of nanotubes, which sets a low baseline brightness. Complexation affinity of saccharides with boronic acids is then used for molecular recognition of sugars. For instance, glucose partially rescued the quenched SWCNT fluorescence functionalized with 4-chlorophenylboronic acid, whereas glucose also caused a wavelength red-shift in SWCNT emission when it was functionalized with 4-cyanophenylboronic acid (Figure 34).^1125^ The fluorescence modulation mechanism in both cases was claimed to be a photoinduced excited-state electron transfer that is disrupted by boronate formation when glucose binds boronic acid. A subsequent study from the same group extended the use of boronic acids for recognition of pentoses such as arabinose, ribose, and xylose.^1126^

These studies represent important conceptual advances for molecular sensing of glucose, but their applications remain at a proof-of-concept level with most demonstrations being carried out in *in vitro* experiments using buffered solutions. Their efficacy for sensing glucose in biological fluids, or in cells and tissues, remains to be demonstrated. Therefore, whether these SWCNT sugar sensors could retain their solution-phase properties in realistic biological specimens, and importantly, the extent to which the kinetics of sensor turn-on and reversibility are compatible with the biochemical processes that dictate the spatiotemporal profiles of endogenous biological events remain unexplored.

In addition to SWCNTs, phenyl boronic acid was also used as a glucose recognition moiety on other CNMs. For instance, graphene quantum dots (GQDs) functionalized with phenyl boronic acid were employed as a non-enzymatic glucose sensor with a sensitivity of 3 mM.^1127^ These ∼10 nm GQDs in PBS solution were excited at 350 nm and had an emission peak at 426 nm. Glucose addition both reduced the photoluminescence intensity and also caused a 9 nm red-shift. The authors hypothesized that the sensing is based on the surface quenching states (SQS) induced mechanism, where glucose molecules bound to PBS groups form negatively charged boronate complexes, stretching the interfaces of the PBS-GQDs to form surface states for efficient fluorescent quenching. This mechanism was specific to glucose as the sensor did not respond to other sugars, such as fructose, galactose, sucrose, or lactose. The authors have also demonstrated the utility of this GQD sensor in real blood serum samples, achieving 93.6–98% recovery rates.^1127^

**Figure 35:**
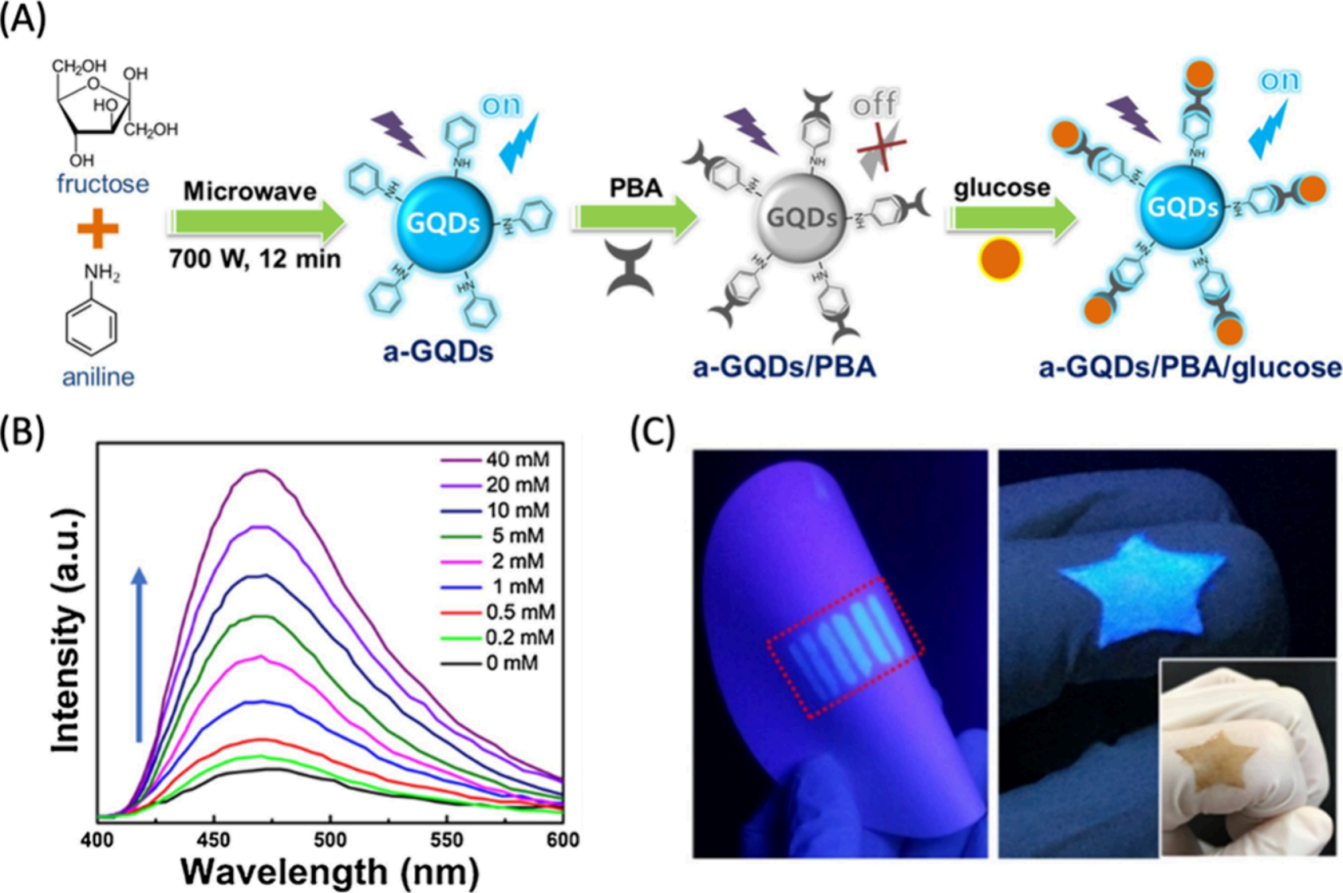
(A) Schematic illustration of a-GQDs synthesis and its glucose sensing mechanism. (B) Fluorescence spectra of a-GQDs/PBA with different glucose concentrations showing the turn-on sensor response. (C) Portable paper-based printed sensor and a wearable composite thin-film sensor responding to patient glucose levels. Adapted with permission from ref (1128). Copyright 2021 Elsevier.

In another non-enzymatic detection study, aniline-functionalized GQDs modified with phenyl boronic acid responded to glucose as a fluorescence turn-on sensor with a remarkable 2 μM sensitivity (Figure 35A).^1128^ Aniline-functionalized GQDs (a-GQDs) have an absorption peak at around 328 nm corresponding to n→π* electronic transitions related to the conversion of −COOH groups to O=C–N–H bond after aniline functionalization, and emission peak around 460 nm. Presence of the phenyl boric acid (PBA) quenches this GQD fluorescence as the amine groups of a-GQDs attract the boronic acid groups by electrostatic interaction, resulting in a close spatial orientation between PBA and a-GQDs. PBA forms π–π stacking interactions with the aniline molecules, allowing for electron transfer from a-GQDs to PBA and quenching the fluorescence. Addition of the analyte glucose then recovers the fluorescence (Figure 35B) by disassembling the PBA linker from a-GQDs as the boronic acid groups form negatively charged boronic ester complexes with the cis-diols of glucose. This sensor was also employed as a portable paper-based printed sensor and a wearable composite thin-film sensor, and was able to detect glucose in human blood serum and tear samples demonstrating its promise in real life biomedical applications (Figure 35C).

Enzymatic SWCNT-based saccharide sensors have also been developed. In one study, Barone et al. non-covalently functionalized SWCNTs with glucose oxidase (GOx).^1129^ Before GOx functionalization, the nanotube is oxidized with the application of ferricyanide anion (Fe(CN)_6_^3^)^*–*^, which adsorbs on SWCNT surfaces and abstracts electrons from the nanotube’s valence band and bleaches optical transitions, lowering the nanotube’s Fermi level in the process. In this manner, the oxidized nanotubes experience a quenched baseline fluorescence. In the presence of glucose, GOx produces H_2_O_2_ as a catalytic byproduct, which is observed to increase the fluorescence of the quenched nanotube. The liberated H_2_O_2_ is hypothesized to partially reduce the Fe^3+^ core of ferricyanide to Fe^2+^ (ferrocyanide), lowering its oxidative effect and ameliorating transition bleaching in nanotubes. This is detected as a partial rescue of the quenched nanotube’s fluorescence. The authors demonstrate the efficacy of the sensor in *in vitro* solution-phase experiments.

Following up on this work, two recent reports demonstrate that GOx-functionalized SWCNTs can directly sense glucose without the need for an electroactive intermediary to facilitate charge transfer reactions between the nanotube and GOx.^1130,1131^ In one study, SWCNTs were directly functionalized with GOx through a two-step ligand exchange process as described in Section 5.1.2.^1130^ Addition of 30 mM glucose generated a maximal sensor response of ∼35% ΔF/F. The positive sensor response suggested that the catalytic product of GOx-glucose reaction, H_2_O_2_, is unlikely to be the cause of the fluorescence modulation. The authors suggested direct reversible doping of nanotube surface defects, mediated by molecularly adsorbed oxygen, as a mechanism for the observed optical modulation. A subsequent study from the same group used bioconjugation reactions to outfit GOx with pyrene moieties that can anchor the enzyme to the nanotube surface through π-stacking interactions.^1131^ Several recombinant GOx variants were anchored on nanotube surfaces and screened for their optical responses to glucose, which led to the identification of a variant that produced optical modulations with single milli-molar sensitivity, a feat the authors attribute to the oriented enzyme loading facilitated by pyrene binding.

##### 5.3.1.3.2 Lipids

**Figure 36:**
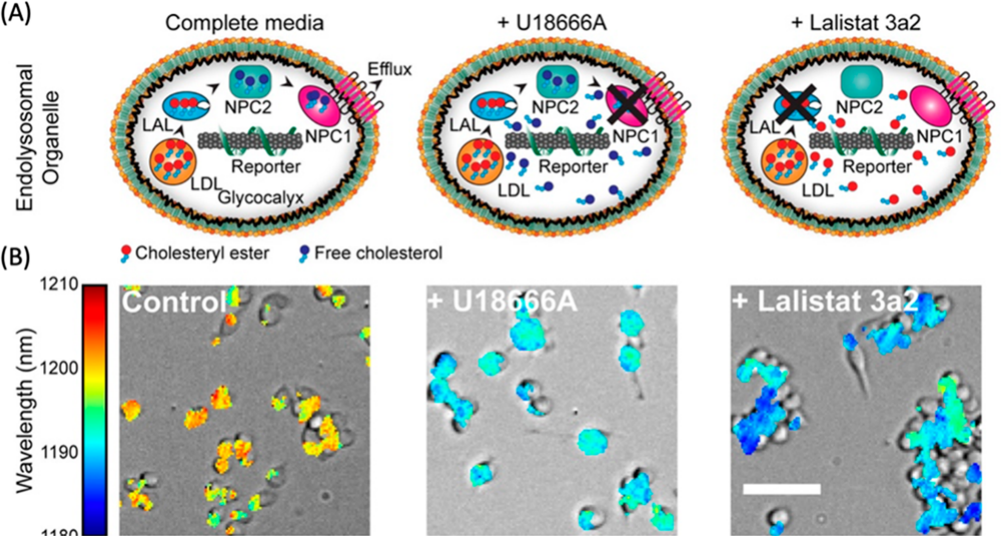
Detection of endolysosomal lipid accumulation in live cells. (A) Schematics of the ss(GT)_6_-(8,6) SWCNTs in macrophages treated with compounds that accumulate lipids in cells (U18666A or Lalistat 3a2). (B) Overlay of brightfield and hyperspectral images of macrophages incubated with sensors under the specified treatments. Color legend maps to nanotube emission peak wavelength. Scale bar = 50 μm. Adapted from ref (1132). Copyright 2017 American Chemical Society.

Beyond sugars, CNM-based optical biosensors for lipids have been reported. Two studies have demonstrated the utility of SWCNT-based lipid biosensors in live cell experiments and *in vivo.*(1112,1113) In the first study,^1132^ Jena et al. leveraged classical works of Zheng and colleagues that show that oligonucleotides of specific length and sequence chemistry can be used as molecular tweezers to purify specific nanotube chiralities.^1133^ One oligonucleotide sequence that enriches the (8,6) nanotube chirality was also shown to exhibit a hypsochromic shift in the emission peak of the nanotube in response to soluble lipids and lipid analogues (Figure 36). The authors demonstrate that when incubated with macrophages, these lipid nanosensors localize to lysosomal compartments, which afforded sensing of lipid accumulation in these organelles. Using these sensors, the authors demonstrate that biochemical perturbations that lead to accumulation of lipids in lysosomes recapitulate the hypsochromic shift observed in solution phase experiments, suggesting the probes retain their sensing efficacy inside cells.

In a subsequent study,^1134^ Galassi et al. employ a similar approach of using ssDNA-purified single-color emitters of SWCNTs for sensing lysosomal lipid accumulation *in vivo* in liver macrophage cells. Tail vain injection of the probes into mice led to sequestration of the sensors in the liver. Perturbations that induce accumulation of lipids in the liver, such as a high fat diet, generated sensor response profiles similar to those seen *in vitro*, suggesting that the probes can be used to study a variety of lipid storage disorders in animal models of disease. While the successful application of these sensor responses in cells and *in vivo* is important, the selectivity of the probes for specific types of lipids over a broader range of lipid and nonlipid biomolecules has not been sufficiently demonstrated. Moreover, sensor kinetics and the feasibility of using these sensors to track fast changing dynamical biological processes has not been fully explored.

Lipid droplets (LD) are cellular organelles consisting of a phospholipid layer and a hydrophobic lipid core and serve as important lipid storage locations inside cells. Small molecule fluorescent probes are often used for visualizing and studying dynamic biochemical processes involving LDs, but a recent report shows that CNM-derived probes could be employed for studying LDs. Boron and nitrogen co-doped CDs (BNCDs) were employed for selective and intrinsic staining of LDs in cells.^1114^ Using a BODIPY-based LD probe as a positive control, the authors showed that BNCDs exhibited a high degree of overlap with LDs, and low levels of overlap with lysosomes and endoplasmic reticulum, demonstrating a specificity that is on par with fluorescent organic probes. The authors further demonstrated the robustness of the probe by testing LD-labeling in five different cell lines. Importantly, whereas most organic fluorescent probes require targeting ligands for LD labeling, a process that entails complex organic synthesis and purification, the CDs reported in this study appear to be straightforward in their synthesis and do not require introduction of LD targeting ligands for their function. The authors also demonstrated that BNCDs can be used for studying biochemical processes that control the dynamics of LDs inside cells, including lipophagy.^1135^

##### 5.3.1.3.3 Other Metabolites

Ascorbic acid is another important metabolite of interest for sensing oxidative stress and related diseases. Wei et al. used CD fluorescence quenching for reversible detection of ascorbic acid in the concentration range of 1–30 μM in a selective manner, though authors did not provide a discussion of what endowed this selectivity.^1116^ Similarly, N-doped CDs were recently used as in-solution fluorescence turn-off sensors for ascorbic acid with a detection limit of 2.6 μM.^1117^ Besides CDs, single-chirality SWCNTs wrapped with oligonucleotides were also developed as ascorbic acid sensors; however, these were not specific to the analyte and also responded to dopamine and riboflavin.^1115^

CDs have also been used for sensing uric acid and urea with demonstrated potential for human health diagnostic applications. For instance, N and P co-doped CDs were used to detect uric acid in human fluids.^1120^ Zhang et al. furthered this study by improving uric acid detection limit to 0.14 μM in human fluid samples using iron and nitrogen co-doped CDs.^1118^ For urea sensing, which is important for health monitoring, an integrated fluorescent CD nanosensors with pH-responsive plasmonic silver nanoparticles have been reported.^1119^ The urea increases the pH and generates plasmonic Ag NPs *in situ*, which quenches the CD fluorescence with a linear range of detection between 100 nM and 1 mM.

Mycotoxins are synthesized by a variety of fungi, and they exhibit many toxic effects in humans, therefore, their detection is an important pursuit. Numerous CNM-based biosensors have been developed for sensing mycotoxins using recognition moieties of antibodies, aptamers, and molecularly imprinted polymers on metal-doped GO sheets, SWCNTs, GQDs, CDs, among others. For a comprehensive discussion of mycotoxin detection via CNMs, readers are referred to a recent review by Ma et al.^1121^

##### 5.3.1.4 Reactive Oxygen and Nitrogen Species (ROS/RNS)

Reactive oxygen species (ROS) and reactive nitrogen species (RNS) are highly reactive species primarily composed of oxygen and nitrogen, respectively, and can be produced under normal physiological processes in humans and many other organisms. Common ROS species include hydrogen peroxide (H_2_O_2_), hypochlorite (ClO^–^), hydroxyl radicals (OH^–^), superoxide anion radicals (O_2_^–^), and singlet oxygen (^1^O_2_), and common biological RNS molecules are nitric oxide (NO), nitroxyl (HNO), nitrogen dioxide (NO_2_), and peroxynitrite (ONOO^–^). These molecules play a role in oxidative stress linked to various pathologies, including Parkinson’s and Alzheimer’s diseases, inflammation, diabetes, and cancer. As such, ROS and RNS are important targets of biosensing by CNMs. Developments in the area are discussed in a comprehensive review by Kwon et al.^1136^ ROS/RNS sensing in the context of plant biology is additionally discussed in Section 5.3.2 of this review.

##### 5.3.1.4.1 ROS

H_2_O_2_ is one classical ROS that has been successfully studied using fluorescent biosensors developed from SWCNTs. The sensitivity of SWCNT fluorescence emission to H_2_O_2_ is well-characterized, and has been attributed to reversible charge transfer from the valence band of the nanotube to the lowest unoccupied molecular orbital of H_2_O_2_.^1137^ This makes it possible to study biochemical processes in which the release of H_2_O_2_ plays an important role, or can be employed for the study of enzymatic reactions that produce H_2_O_2_ as a byproduct (see discussions on glucose sensing in preceding sections of this review). These reactions between nanotubes and H_2_O_2_ generally lead to reduction in the intensity of nanotube’s fluorescence emission and have been leveraged to sense the release of H_2_O_2_ generated by biochemical processes. In one such study, the dynamics of H_2_O_2_ release from A431 human epidermal carcinoma cells is investigated.^1138^ Activation of a receptor expressed in these cells is thought to induce the release of H_2_O_2_, but the spatiotemporal profile of the H_2_O_2_ release had remained mostly unknown. A431 cells grown on a surface immobilized array of collagen passivated-SWCNTs and subsequently stimulated to evoke biochemical release of H_2_O_2_ induced localized quenching of SWCNT emission with putative single molecule sensitivity. The study relies on the statistical signal aggregation from extended imaging frames, likey due to small signal-to-noise ratio of detection, which makes it difficult to assess the extent to which the signal faithfully recapitulates the purported underlying biological phenomena. Nonetheless, the study made an important demonstration of the utility of the approach for detection of endogenously produced ROS. A related study from the same group used the spectral diversity of SWCNTs for multimodal detection of singlet oxygen and hydroxyl radicals in addition to H_2_O_2_.^1139^ Interestingly, the study demonstrates that not all ROS affect SWCNT photoluminescence similarly: for example, H_2_O_2_ was noted to induce fluorescence reduction of lower bandgap SWCNTs (e.g., (7,5) nanotubes) more vigorously than higher bandgap nanotubes (e.g., (6,5) nanotubes). On the other hand, singlet oxygen and hydroxyl radicals produced a unique combination of spectral shifts and intensity attenuations that the authors use for multimodal ROS signal detection. The utility of this approach was demonstrated in cultured 3T3 cells that are exposed to perfusions of exogenously prepared ROS. However, the efficacy of this approach for multimodal sensing of biochemically generated ROS, where the potential impact of successful demonstration would have been the highest, remains unknown.

More recently, other CNM platforms have been reported for indirect and direct optical detection of ROS/RNS. Indirectly, iron and nitrogen co-doped CDs (Fe/N-CDs) have been used together with o-phenylenediamine (OPD) to detect H_2_O_2_ generation in human serum and urine samples.^1118^ Fe/N-CDs emit strong fluorescence at 449 nm under UV excitation. The presence/generation of H_2_O_2_ oxidizes OPD, which then causes CD fluorescence quenching via FRET as the oxidation product, 2,3-diaminophenazine (oxOPD), absorbs at 420 nm and emits at 555 nm. Since the amount of product generated is linearly correlated with the amount of H_2_O_2_ consumed, the detection of H_2_O_2_ can be achieved by monitoring both ratiometric fluorescence at 555 nm/449 nm and colorimetric absorption at 420 nm. This dual detection platform exhibits notable selectivity and sensitivity toward H_2_O_2_ with a detection limit of 70 nM.

**Figure 37:**
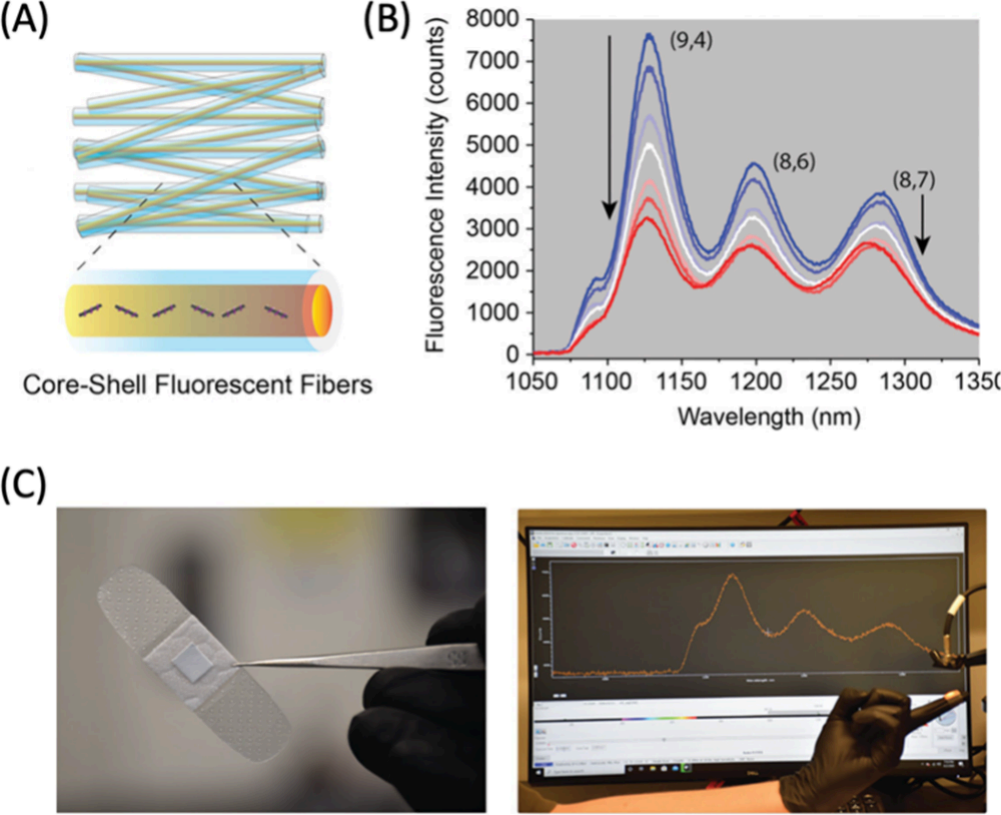
(A) Schematic of the ss(GT)_15_-SWCNT sensors encapsulated in PCL polymers. (B) The fluorescence spectra of the microfibrous samples exposed to various H_2_O_2_ concentrations ranging from 0 to 5 mM. (C) Optical fibrous samples that are integrated into a commercial wound bandage still responds to exogenously applied H_2_O_2_. Adapted with permission from ref (1140). Copyright 2021 Wiley.

On the other hand, direct sensing of H_2_O_2_ was achieved through the use of core–polycaprolactone (PCL) shell microfibrous textiles incorporating SWCNTs for the real-time optical monitoring of H_2_O_2_ in *in vitro* wounds within a physiologically relevant range (1–250 μM).^1140^ This study used (GT)_15_-wrapped SWCNTs that are responsive to H_2_O_2_ and employed an electrospinning process to encapsulate them in poly(ethylene oxide) (PEO) and PCL polymers is a fiber format (Figure 37A). Hyperspectral fluorescence imaging revealed that all (9,4), (8,6), and (8,7)-SWCNT chiralities quench upon exposure to H_2_O_2_; however, the extent of quenching varies among the chiralities, creating a ratiometric sensor (Figure 37B). For real-life applications, attaching the fibrous sensors onto a commercial wound bandage enabled real-time wireless wound screening over 7 days (Figure 37C).

Besides H_2_O_2_, CDs for the detection of hypochlorous acid (HClO) at fresh wounds of zebrafish larvae have been reported.^1141^ These CDs have a continuous absorption ranging from UV to more than 500 nm, displaying light yellow color under room light. Their excitation and emission peaks locate at 496 and 537 nm, respectively. Because these CDs are very bright and possess abundant phenolic hydroxyl groups on their surface, authors tested their use for ROS sensing, and demonstrated that HClO causes a distinct blue emission after a 10 min reaction with a detection limit of 8.60 nM. These experiments were also successfully replicated in A549 cells and 7-day old zebrafish larvae, noting the potential for real life applications, even though the mechanisms behind sensing and its specificity are not explored or discussed in this study.

##### 5.3.1.4.2 RNS

NO is a gaseous messenger molecule that is ubiquitously employed in living systems, including the cardiovascular system, nervous system, and immune system. The small and labile nature, and high reactivity of NO make development of optical probes for NO a challenge. The importance of NO signaling has led to the development of several classes of genetically encoded probes.^1142^ Similarly, CNMs have been successfully employed for the development of sensing technologies for NO.^1143,1144^ SWCNTs in particular have been successfully exploited for biosensing of NO *in vitro* and *in vivo*.

The first such report relied on 3,4-daminophenyl-functionalized dextran (DAP-dex) as a non-covalent functional motif for SWCNTs.^1145^ DAP-dex enables colloidally stable dispersions to be prepared from SWCNTs and covers the surface of SWCNTs to enable NO-specific analyte binding and optical perturbation. In this report, NO induces a fast and reversible photobleaching of the SWCNT photoluminescence, which is attributed to the electron transfer from SWCNT to NO. In addition to the *in vitro* spectroscopy of the sensor, the study demonstrates that the sensor retains its NO sensitivity in exogenous NO wash experiments, as well as endogenous NO release from stimulated cultured cells. In a follow up study, Zhang et al. demonstrated a ssDNA@SWCNT-based sensor for *in vitro* NO detection.^1146^ In subsequent studies, Iverson et al. demonstrated that PEG-functionalized versions of ssDNA@SWCNT-based NO sensors can be injected into mice, in which the sensors localized in the liver and responded to endogenous NO levels in a mouse model of inflammation.^1147^ Additionally, hydrogel-embedded NO nanosensors in that study were investigated for their long-term use *in vivo*.

Besides SWCNTs, other CNMs such as CDs, have been used for RNS detection. For instance, benzylamine-passivated CDs (B-CDs) were developed to detect NO and NO_2_^–^ under different pH conditions in aqueous media with a limit of detection of as low as 43 nM and 0.65 μM, respectively.^1148^ Here, 2.5 nm B-CDs absorb at 264 and 333 nm, which are attributed to the π→π* transition of aromatic sp^2^ domains from the carbon core and the n→π* transition of C=O, respectively. When excited at 375 nm, B-CDs have an emission peak at 460 nm. The presence of NO was shown to decrease the B-CD fluorescence linearly within the concertation range of 0–180 μM. Interestingly, this sensor showed no response toward ^1^O_2_, ·OH, ONOO^–^, NO_2_^–^, NO_3_^–^, HNO, H_2_O_2_, ClO^–^, ascorbic acid, cysteine, dehydroascorbic acid, or methylglyoxal. Authors verified that the fluorescence quenching mechanism of NO is static quenching, which occurs when a non-fluorescent ground-state complex or a weakly fluorescent complex is formed by the interaction between CDs and quenchers.^1148^

Moreover, folic acid-functionalized CDs (CdotsFA) were able to selectively sense NO levels down to the 10 nM range via fluorescence quenching.^1149^ Authors selected folic acid (FA) functionalization as FA has a redox regulator role, and showed that the presence of 10^–10^ M NO· effectively quenched the fluorescence intensity of the CdotsFA and caused an 8.3 nm red-shift in CD emission. The sensing mechanism was not revealed in this study, but a strong binding constant between CdotsFA and NO was shown.

##### 5.3.1.5 Bacteria and Viruses

Detection of viral or bacterial pathogens is important for early diagnosis and treatment of many human diseases. For a comprehensive review of viral pathogen detection using CNMs, readers are encouraged to refer to Bardhan et al.^1150^ for pre-2021 literature. Similarly, for comprehensive reviews of bacterial pathogen detection, readers are invited to refer to Alafeef et al.^1151^ and Cui et al.^1152^ for pre-2020 literature. More recently, there have been several exciting advancements in the field of virus and bacteria detection. Pinals et al. constructed a SWCNT nanosensor that is functionalized with ACE2, a protein that binds SARS-CoV-2 spike protein.^1153^ SARS-CoV-2 spike protein causes a 2-fold fluorescence increase of this nanosensor 90 min after exposure in solution. However, the surface-immobilized version was able to detect 35 mg/L SARS-CoV-2 virus-like particles within 5 sec in human saliva.

For pathogenic bacteria sensing, Nißler et al. developed a set of SWCNT nanosensors functionalized with molecules that can specifically detect the metabolites released by bacteria and specific virulence factors such as lipopolysaccharides, DNases, and proteases.^1154^ Using this approach, they were able to sense and differentiate clinical isolates of six important bacteria. More recently, a unique approach was taken for optical sensing of odors specific to certain bacterial infections. In this study, Shumeiko et al. functionalized SWCNTs with artificial olfactory sensors consisting of peptides for specific detection of *Escherichia coli* and *Klebsiella pneumoniae.*(1155) Lastly, a label-free fluorescent carbon nanosensor embedded in agarose was developed for detection based on pH changes that can rapidly discriminate pathogens in real time.^1156^ The detection relies on the pH-triggered aggregation-induced emission quenching of nanosensors in a physiologically relevant pH range and demonstrated single cell resolution with rapid response time.

##### 5.3.1.6 Metal Ions

Concentration of metal ions in organisms or in the environment can affect health and wellness. CNMs have been used for electrochemical sensing of transition metal ions.^1157^ In addition, optical detection of metal ions has also been demonstrated, as reported in a recent study.^1158^ SWCNTs coated with a melanin-like supramolecular complex were observed to exhibit dose-dependent modulation of their fluorescence in response to divalent transition metal ions, including Cu^2+^, Hg^2+^, Mn^2+^, and Fe^2+^. Interestingly, in addition to detecting these ions, nanotube–polymer complexes exhibited a remarkable ability to chelate these metal ion species and remove them from solution, suggesting a potential role as scavengers of free metal ions from solution. A more extensive review of metal ion sensing with relevance to environmental applications is summarized in Table 7.

**Table 7:** Advances in CNM Fluorescence Sensors for Environmental Monitoring

	Target	Materials	System	Limit of Detection	Ref
Pathogens	Polyphenols	SWCNTs	Soybean and leaf tissue of *Tococa spp.*	–	(1159)
	*E. coli*	CDs-microspheres	Milk	240 CFU/mL	(1160)
	*E. coli* and *S. aureus*	CsWO_3_-CDs	*In vitro*	70 CFU/mL	(1161)
	*P. aeruginosa* and *S. aureus*	SWCNTs in hydrogel	*In vitro*	–	(1163)
	Acyl-homoserine lactones from Gram-negative bacteria	Citric acid and glycine CDs	Fish, juice, and milk	<7 × 10^–5^ μM	(1164)
	*E. coli*, *S. sciuri*, *D. desulfuricans*, *L. monocytogenes*, *S. aureus*, and *P. aeruginosa*	Boronic acid, polymixin, and vancomycin functionalized CDs	*In vitro*	–	(1165)
	*E. coli* and *S. aureus*	Ag-CDs	River water	0.1 mg/mL	(1166)
	*S. aureus*	Fe_3_O_4_-CDs	Milk and juice	8 CFU/mL	(1035)
	*P. aeruginosa*	NiFe_2_O_4_-CDs	*In vitro*	–	(1167)
ROS/RNS	H_2_O_2_	Hemin-aptamer-SWCNTs	*A. thaliana* leaves	–	(1225)
	H_2_O_2_, dopamine, riboflavin, ascorbic acid, pH	ssDNA-SWCNTs	*In vitro*	–	(1226)
	H_2_O_2_	SWCNTs	Lettuce, arugula, spinach, strawberry blite, sorrel, and *A. thaliana* leaves	–	(1227)
	H_2_O_2_/NO	SWCNTs	*A.thaliana* leaves	–	(1228)
	ClO^–^	N-CDs	Pool and tap water	0.03 μM	(1229)
Plant hormones	Gibberellins	SWCNTs	*Arabidopsis*, lettuce, and basil roots	GA_3_ (542 nM) and GA_4_ (2.96 μM)	(1220)
	Zeatin	Dye-labeled aptamers-GO	Plant tissue culture	60 nM	(1221)
	Jasmonic acid	NCQDs@Co metal organic frameworks, molecularly imprinted polymers	Rice leaves	0.35 ng/mL	(1222)
	Abscisic acid	CQDs@ZIF-8/Apt-AuNPs	Rice seeds	30.0 ng/mL	(1246)
	NAA (1-naphthalene acetic acid) and 2,4-D (2,4-dichlorophenoxyacetic acid)	Cationic polymer wrapped-SWCNTs	Spinach, *A. thaliana*, bok choy, and rice	NAA (8.2 μM) and 2,4-D (0.35 μM)	(1224)
Contaminants and explosives	TNP	CD+wood	*In vitro*	0.27 μM	(1168)
	Picric acid	S-CDs	*In vitro*	3.2 μM	(1169)
	Picric acid	N-CDs	Industrial effluent water	1.8 nM	(1170)
	TNT	N-CDs	Sandy soil	30.0 nM	(1171)
	Hg^2+^ and thiophanate methyl	Thioctic acid-CDs	Tap water, grape juice, and Citri Reticulatae Pericarpium water	Hg^2+^ (33.3 nM) and TM (7.6 nM)	(1172)
	Malathion	Au-CDs	Entire cabbage	1 nM	(1173)
	Methyl orange, rhodamine 6G, and bromophenol blue	N-Oxidized CDs	*In vitro*	38 nM	(1174)
	Carbendazim	Serine and histidine functionalized GQDs	Tomatoes	6.1 × 10^–17^ M	(1175)
	Pyrene	GO-GQDs	Lake water	0.325 μM	(1176)
	Tetracycline hydrochloride	Poly(diallyldimethylammonium) chloride functionalized graphene	Milk	0.9284 nM	(1177)
	TNP	CDs	Lake and tap water	1.31 μM (tap water) and 0.99 μM (lake water)	(1178)
	Tetracycline	Alginate-CDs	*In vitro*	2 μM	(1179)
	PNP (4-nitrophenol), DNP (1,3-dinitrophenol), TNP (1,3,5-trinitrophenol), MET (metronidazole), DCNA (2,6-dichloro-4-nitroaniline)	NiFe_2_O_4_-CDs	River water	PNP: 65 nM	(1180)
				DNP: 74 nM	
				TNP: 78 nM	
				MET: 57 nM	
				DCNA: 100 nM	
	TNP	ZnSe-CDs	River water	12.4 μM	(1181)
	F^–^	Pyrene-boronic acid-based CDs	*In vitro*	0.59 μM	(1182)
	Glyphosate	N-CDs@SiO_2_	Malt	3.4 ng/mL	(1183)
	Methotrexate	N,S-CDs	*In vitro*	12 ng/mL	(1184)
	Malaoxon	GO sheet	Agricultural surplus water, carrot, and grape juice	1 nM	(1185)
Metals	As^3+^	SWCNTs	Spinach, Cretan brake fern, and indica rice	0.6 and 0.2 ppb of As after 7 and 14 d	(1186)
	Ag^+^	N-CDs	Lake water	0.5 nM	(1187)
	Cr^6+^	Chitosan-based hydrogel/titanate/cellulose nanofibers-CDs	*In vitro*	8.5 mg/L	(1188)
	Hg^2+^	Eu-CDs	Drinking water and milk	0.2 nM	(1189)
	Pb^2+^	Thiolated-CDs	Onion cell walls	–	(1190)
	Fe^3+^	CDs	*In vitro*	355 nM	(1191)
	Hg^2+^, Pb^2+^, and Cu^2+^	CDs	Pearl river water	Hg^2+^ (5.8 nM), Pb^2+^ (0.12 μM), and Cu^2+^ (0.076 μM)	(1192)
	Cr^6+^	EDTA-CDs	River water	0.8 μM	(1193)
	Fe^2+^	CDs	*In vitro*	0.62 ppm	(1194)
	Cr^6+^	Benzalkonium chloride-CDs	Tap water	0.03 μM	(1195)
	Cu^2+^	N,S-CDs	Tap water	0.3 μg/mL	(1196)
	Pb^2+^	CDs	*In vitro*	58.63 μM	(1197)
	Hg^2+^ and Pb^2+^	Nanofiber/Fe-CDs	*In vitro*	–	(1198)
	Co^2+^, Fe^3+^, Hg^2+^, and Pb^2+^	CDs	*In vitro*	Co^2+^ (96.8 nM), Fe^3+^ (61.7 nM), Hg^2+^ (39.5 nM), and Pb^2+^ (37.1 nM)	(1199)
	Pb^2+^ and Cu^2+^	AuCNs/N-CDs	River water	Pb^2+^ (0.5 μM) and Cu^2+^ (0.15 μM)	(1200)
	Hg^2+^	CDs	River water	1.26 ng/mL	(1201)
	Pb^2+^, Cu^2+^, and Ni^2+^	CDs	*In vitro*	Pb^2+^ (0.01 μM), Cu^2+^ (0.1 μM), and Ni^2+^ (0.1 μM)	(1202)
	Cu^2+^	CDs	*In vitro*	10 nM	(1203)
	Cr^3+^ and Pb^2+^	CDs	River water	Cr^3+^ (27 nM) and Pb^2+^ (34 nM)	(1204)
	Pb^2+^ and Hg^2+^	CDs	River water	Pb^2+^ (0.14 nM) and Hg^2+^ (0.22 nM)	(1205)
	Fe^3+^	CDs	*In vitro*	18 mg/L	(1206)
	Hg^2+^	N-CDs	Seafood	37 nM	(1207)
	Ag^+^	CDs	River water	1.4 nM	(1208)
	Hg^2+^	Graphitic carbon nitride quantum dots	Tap and rainwater	–	(1209)
	Fe^3+^, Cu^2+^, and Hg^2+^	N-GQDs	Tap water	Fe^3+^ (66.7 nM), Cu^2+^ (146.5 nM), and Hg^2+^ (47.9 nM)	(1210)
	Hg^2+^ and Fe^3+^	N-GQDs	Drinking water	0.1 μM	(1211)
	Hg^2+^ and F^–^	B,N-GQDs	*In vitro*	Hg^2+^ (0.16 μM) and F^–^ (0.18 mM)	(1212)
	Hg^2+^	B,N-GQDs	River water	6.4 nM	(1213)
	Fe^3+^	B-GQDs	River water	31.2 nM	(1214)
	Hg^2+^, Pb^2+^, Cr^2+^, and Mn^2+^	SWCNTs	Fish tissue extract	33 nM for Hg^2+^	(1264)
	Cu^2+^, Cd ^2+^, Hg^2+^, and Pb^2+^	DNA-wrapped SWCNTs	*In vitro*	100 μM	(1265)
VOC (volatile organic compounds)	Diisopropylamine (DIPA) and dioxane	Carbon nano-onion (CNO)	*In vitro*	200 nM	(1216)
	Acetone	CDs	*In vitro*	1.5% v/v of acetone to water	(1217)
	Cr(VI), nitrobenzene (NB), m-nitroaniline (m-NA), and p-nitroaniline (p-NA)	MO-CNTs	*In vitro*	Cr(VI) (19 nM), NB (2 nM), m-NA (2 nM), and p-NA (2 nM)	(1218)
	Chloroform	CDs	*In vitro*	3 ppb	(1219)

#### 5.3.2 Environmentally Relevant Sensing

CNMs have gained significant attention in recent years as fluorescent sensors for environmentally relevant molecules. This section presents a review of the research surrounding the application of CNMs in environmental optical sensing, focusing on post-2019 literature (Table 7). Key focal areas encompass sensing of pathogens,^1035,1159−1167^ explosives and contaminants,^1168−1185^ metal ions,^1186−1215^ volatile organic compounds (VOCs),^1216−1219^ plant hormones,^1220−1224^ and ROS/RNS.^1225−1229^ For a more detailed overview of the broad use of CNMs as fluorescence sensors, readers may refer to the other reviews.^1230−1239^

##### 5.3.2.1 Pathogen Sensing

Pathogen detection is vital in environmental monitoring and food safety, playing a key role in protecting ecosystems, preventing disease spread through environmental means, and ensuring the safety of our food. This important practice helps prevent foodborne illnesses, strengthens public health, and supports the sustainable future of the food industry. However, traditional methods like PCR, ELISA, and various culture-based techniques, while effective, can be slow and complex, often taking days to produce results. Therefore, there is an urgent need to develop and adopt faster, more efficient, and reliable technologies for pathogen detection to improve our ability to quickly and accurately detect pathogens.

In a recent study, Roh et al. introduced PVP@Ag:FCD, a novel material that merges photoluminescence-tunable fluorescent carbon dots (FCDs) with silver nanoparticles (AgNPs).^1166^ This combination yields a dual-function tool adept at both detecting and eliminating bacteria. The material operates through a two-pronged mechanism. The first aspect involves bacterial detection, where the FCDs are pivotal. Upon encountering bacteria, these FCDs experience a quenching of their fluorescence, triggered by aggregation-induced quenching stemming from the material’s cationic nature that allows it to electrostatically adhere to the negatively charged bacterial cell surfaces. Simultaneously, the AgNPs within the material unleash their antibacterial properties,^1240^ disrupting bacterial cell walls and ultimately causing bacterial death. This innovative probe has demonstrated significant sensitivity in detecting bacteria, with the ability to identify concentrations as low as 10 CFU/mL for both *E. coli* and *S. aureus*. Furthermore, researchers also demonstrated the material’s efficacy in real-life situations, such as in contaminated river water, where it successfully detects and eradicates bacteria. Even though this versatile tool demonstrates the ability to detect and kill bacteria effectively, the method’s reliance on electrostatic and weak intermolecular interactions poses challenges for real-world sensing applications. Factors such as pH changes, and the presence of other ions, molecules, and particulate matter could interfere with these interactions, potentially resulting in false outcomes. Hence, technologies that utilize molecular recognition elements, such as aptamers and antibodies, are preferred.

**Figure 38:**
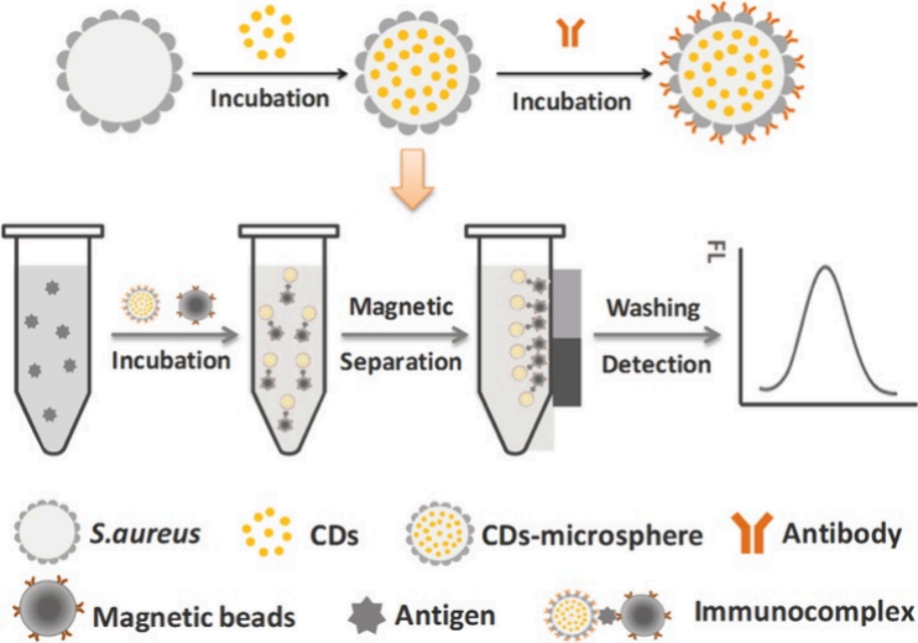
A method for creating cell-based CDs-microspheres utilizing *S. aureus* cells as carriers to encapsulate CDs particles. These inactivated cells can subsequently bind to antibody molecules via SPA proteins present on their surfaces. The development of the CDs-microsphere immunoassay involves the integration of immunomagnetic separation and CDs-microsphere fluorescence detection for pathogen detection. Adapted with permission from ref (1160). Copyright 2021 Elsevier.

Addressing this issue, Zhao et al. present an innovative approach through the development of a cell-based fluorescent microsphere immunosensor for the detection of *E. coli O157:H7* in milk.^1160^ This sensor integrates CDs within nonviable, inactive *S. aureus* cells (Figure 38), utilizing the cells not only as carriers of CDs but also exploiting the inherent binding affinity of Staphylococcal Protein A (SPA) on their surface to antibodies specific to *E. coli O157:H7.* The fluorescence signal generated by the CDs is the key to detection, which occurs when the *E. coli O157:H7* is captured between immunomagnetic beads and the antibody-coated CD-microspheres, forming a sandwich-like structure. This design yields a sensor with exceptional sensitivity, capable of selectively detecting *E. coli* over five other bacteria types, and features a moderate detection limit of 2.4 × 10^2^ CFU/mL. It can detect in roughly 30 min, outperforming PCR^1241^ (2 h) and ELISA^1242^ (2.5 h) in speed, and surpassing the lateral-flow assay in detection limit (4 × 10^3^ CFU/mL). Notably, the sensor’s effectiveness extends to practical scenarios, as evidenced by its successful identification of *E. coli O157:H7* in contaminated milk samples. With its rapid, sensitive, and cost-effective detection capabilities, this sensor represents a notable leap forward in pathogen detection within the realm of food safety.

Regarding pathogen detection, another method, besides directly sensing the pathogens themselves, involves monitoring their metabolites. This approach offers a valuable alternative for evaluating pathogen activity, as these metabolites can be indicative of the presence and intensity of an infection. By analyzing the specific compounds produced by pathogens, it is possible to gain insights into their metabolic processes and potentially identify their presence even when direct detection of the pathogen is challenging. For example, N-acyl homoserine lactones (AHLs) are key molecules in the communication system, known as quorum sensing, of Gram-negative bacteria. This system enables bacteria to gauge their population size and adjust their behavior accordingly, including the production of factors that contribute to virulence, spoilage of food, and formation of biofilms. Common foodborne pathogens, such as *Aeromonas hydrophila*, *Pseudomonas aeruginosa*, and *Hafnia alvei*, are known to produce AHLs during their growth phases, playing a crucial role in the spoilage of food and the onset of foodborne illnesses. Consequently, the detection of AHLs in food items could serve as a reliable indicator of contamination by Gram-negative bacteria.

In a recent study, Cui et al. introduced a novel magnetic fluorescence probe, combining Fe_3_O_4_ particles with CQDs-doped molecularly imprinted polymers (MIPs) for the precise detection of AHLs in food samples.^1164^ This probe operates on a unique mechanism: the Fe_3_O_4_ particles provide a magnetic base, facilitating easy separation and manipulation in complex matrices like fish juice and milk. The MIPs, tailored with specific molecular cavities, selectively bind to AHL molecules. CDs embedded within these MIPs emit a fluorescence signal, which is quenched upon the binding of AHLs, indicating their presence through a decrease in fluorescence intensity. This mechanism allows for a sensitive detection range for AHLs, from 3.65 × 10^–3^ to 0.96 × 10^–1^μmol/L, and boasts a detection limit of 2.99 × 10^–5^ μmol/L. The selectivity of the probe is demonstrated by its ability to distinguish AHLs from six similar compounds (e.g., C_4_-HSL, C_6_-HSL, C_8_-HSL, etc.), showing significant fluorescence quenching with AHLs while exhibiting minimal response to others. The probe’s capability to accurately detect AHLs in real food samples, with recovery rates from 83.10% to 90.74%, further demonstrates its practical applicability. This innovative tool, with its specificity, sensitivity, and magnetic separation features, represents a significant advancement in food safety monitoring, offering a new avenue for rapid and reliable detection of bacterial signals in food products.

**Figure 39:**
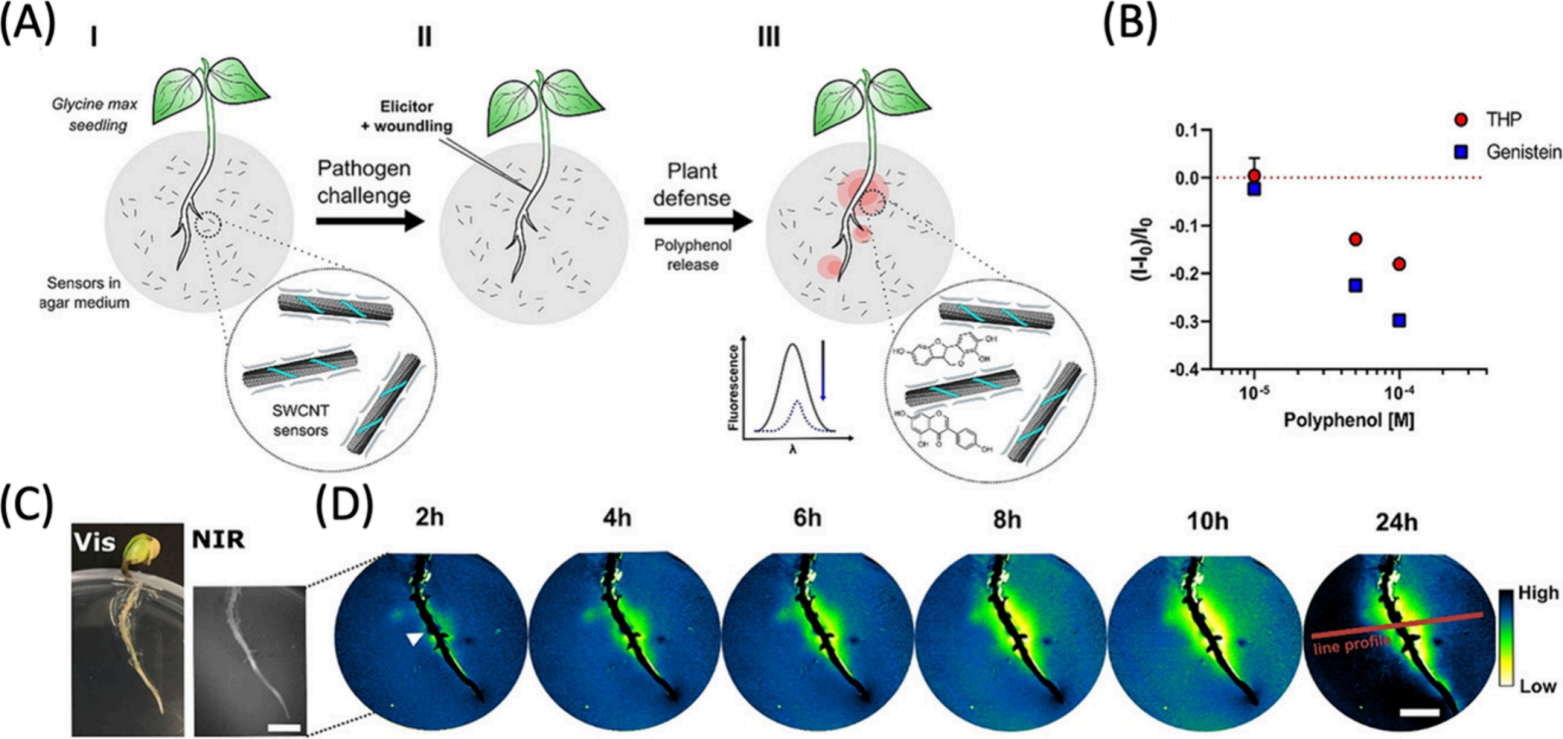
(A) The use of SWCNT-based fluorescent sensors integrated into the agar culture medium. A soybean seedling (*G. max*) grows through the agar, and when the plant encounters a pathogenic elicitor, its response in terms of polyphenol secretion is monitored through NIR fluorescence imaging from a distance of more than 20 cm. (B) Genistein and THP, which are significant components of soybean (*G. max*) polyphenols, reduce the fluorescence of PEG-PL-SWCNTs in the agar (mean ± SD, *n* = 3). (C) Visible and NIR images of the soybean seedling with a scale bar of 1 cm. (D) The NIR fluorescence of the sensors (*I*/*I*_0_) in the plant’s environment (rhizosphere) decreases over time near the challenged root area (where root tissue is indicated by black overlay; the white triangle represents the position for elicitor induction, and the red line shows the line profile position, with a scale bar of 1 cm). Adapted with permission from ref (1159). Copyright 2022 Wiley.

The field of pathogen biosensing has also expanded its applications to agriculture, offering valuable opportunities to enhance our understanding of plant–pathogen interactions.^1243,1244^ By closely monitoring dynamic physiological processes, such as plant’s defense response against pathogenic attacks, we could advance the cultivation of plants with enhanced tolerance to biotic stress. Nißler et al. have made significant progress in this domain through extensive modifications of SWCNT sensors for the detection of plant polyphenols, which are released when the plant is under pathogen attack.^1159^ These modifications entail the incorporation of nucleotides, PEG, and phospholipid (PS) macromolecules onto the surface of SWCNTs, leading to favorable interactions with hydroxy-rich compounds. Consequently, these interactions induce fluorescence quenching or solvatochromic shifting.^1245^ Expanding upon these interactions, the PEG-PL-SWCNT sensors show excellent capability in detecting soybean defensive polyphenols, notably genistein, and trihydroxypterocarpan (Figure 39). These compounds, crucial in the plant’s defense mechanism against pathogens, can be accurately detected and quantified using the developed sensors. The study showcases the effectiveness of these sensors in different plant-based experiments, including their application in real-time monitoring and analysis in agricultural and botanical research. Overall, the study provides a significant advancement in the field of agricultural monitoring by enabling the sensitive and specific detection of plant polyphenols.

##### 5.3.2.2 Plant Hormone Sensing

Detecting plant hormones is a critical aspect of agricultural science and plant biology, offering profound insights into plant growth, development, and responses to environmental stimuli. By accurately identifying and quantifying these hormones, researchers and farmers can better understand how plants adapt to stress, optimize nutrient uptake, and regulate their life cycles. Traditional methods for detecting and analyzing plant hormones include HPLC, gas or liquid chromatography/mass spectrometry (GC or LC/MS), capillary electrophoresis (CE), and ELISA. These techniques, although effective, are not optimal because they require complex sample preparation, expensive equipment, and skilled operators. Additionally, they are destructive methods and fail to offer prompt spatiotemporal results. CNMs have emerged as promising alternatives to traditional technologies, owing to their capacity to enter plant cells and their tunable surface functionalities. These characteristics enable them to target a diverse range of molecules with high specificity.

Shi et al. developed a ratiometric fluorescence aptasensor, specifically designed to detect abscisic acid (ABA),^1246^ a crucial phytohormone that is instrumental in regulating plant growth, seed germination, and environmental stress response. This sensor integrates CQDs within a 2-Methylimidazole zinc salt framework (CQDs@ZIF-8) and couples them with aptamer-functionalized gold nanoparticles (Apt-AuNPs). This unique configuration grants the CQDs@ZIF-8 dual-emission properties, with the ZIF-8 component serving as both a stabilizing anchor and a modulation agent. The sensor’s operation is based on the specific interaction between the ABA and aptamers on the Apt-AuNPs. When ABA binds to these aptamers, it alters the configuration of the Apt-AuNPs, thereby disrupting the FRET between CQDs@ZIF-8 and Apt-AuNPs. This disruption manifests as a change in fluorescence intensity at two distinct wavelengths (increase in 490 nm and decrease in 657 nm). Remarkably, the sensor is capable of detecting a wide range of ABA concentrations, with the relationship between the fluorescence signal and the ABA concentration estimated by two linear regression equations for the respective ranges of 0.100–10.0 ng/mL and 10.0–150 ng/mL. The detection limit of the assay is calculated to be 0.03 ng/mL, based on the principle of triple signal-to-noise ratio (S/N = 3). Its practicality was further validated through successful applications in measuring ABA levels in rice seeds, delivering results that are comparable to those obtained using the standard LC/MS method. In addition to its sensitivity and broad detection range, the sensor is selective, reliably distinguishing ABA even amidst various potential interfering substances, including but not limited to Na^+^, Ca^2+^, and K^+^. This high specificity is complemented by the sensor’s long-term stability, maintaining its signal integrity with minimal decay over a 30-day storage period at 4 °C. Despite these significant advantages, the article did not test the probe’s reversibility or its long-term monitoring capabilities in plant tissues. These aspects represent limitations that would need further investigation.

In addition to their work on the ABA sensor, Shi and colleagues expanded their research to develop a sensor for jasmonic acid (JA),^1247^ a plant hormone essential for growth, reproduction, and defense mechanisms. They engineered a ratiometric fluorescent probe by synergizing three components: nitrogen-doped carbon quantum dots (NCQDs), cobalt-based metal–organic frameworks (Co-MOFs), and molecularly imprinted polymers (MIPs). The detection mechanism begins with the NCQDs, which are fine-tuned by nitrogen doping to emit fluorescence at two distinct wavelengths. These NCQDs are initially insensitive to JA due to their surface charge and resulting repulsion between NCQDs and JA. However, the incorporation of Co-MOFs alters this charge and reduces the electrostatic repulsion, hence priming the NCQDs for interaction with JA. MIPs are then strategically attached to the Co-MOFs. These polymers are engineered with specific cavities that mimic the shape of the JA molecule, ensuring that the probe selectively binds to JA with high fidelity, akin to a lock and key mechanism. Upon encountering JA, the NCQD undergoes a photoinduced electron transfer (PET) process. This interaction prompts a dual-wavelength fluorescent response: the fluorescence at 367 nm diminishes, while that at 442 nm intensifies. The JA detection range of the probe is between 1 and 800 ng/mL. This sensitivity was evidenced in tests conducted on various rice strains, demonstrating the probe’s ability to accurately quantify endogenous JA levels. Specifically, higher levels of JA were observed in the disease-resistant variety (Nanjing 46) compared to the infected varieties (Yongyou 9 and Jiaheyou 218), highlighting the role of JA in plant defense. Furthermore, the probe’s measurements correlated strongly with those obtained from LC/MS, affirming its accuracy. The sensors developed by Shi and colleagues are significant advancements in agricultural research, offering a more effective means for detecting critical hormones like ABA and JA.

**Figure 40:**
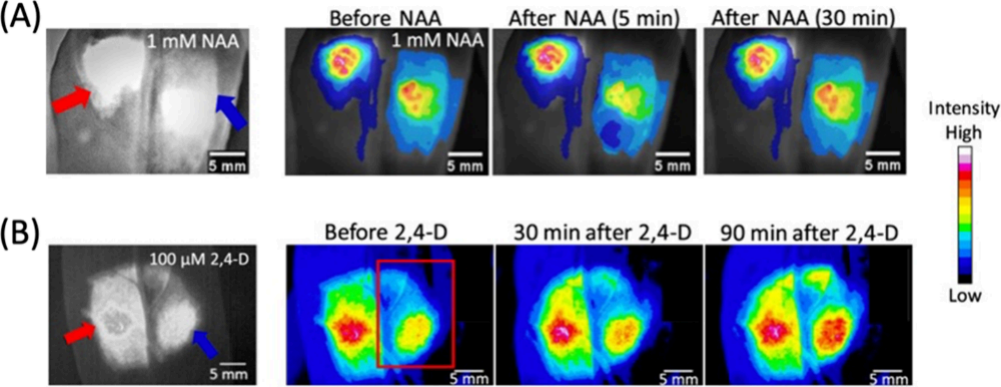
Spatial and temporal patterns of NAA and 2,4-D in spinach leaves. (A) Bright-field and false-color fluorescent images of a spinach leaf from a whole plant, showing infiltration with reference (red) and 1 mM NAA (blue) sensors under 785 nm laser light after 5 and 30 min. (B) Bright-field and false-color fluorescent images of a spinach leaf with reference (red) and 100 μM 2,4-D (blue) sensors under 785 nm laser light after 30 and 90 min. Adapted from ref (1248). Copyright 2021 American Chemical Society.

Besides ABA and JA, Ang et al. developed a SWCNT-based sensors used in detecting synthetic auxins 1-naphthalene acetic acid (NAA) and 2,4-dichlorophenoxyacetic acid (2,4-D), where NAA is commonly used as a rooting hormone powder and as a plant spray to prevent premature flowering and 2,4-D is used as a herbicide that selectively kills broadleaf dicotyledonous weeds while being generally tolerated by monocotyledonous crops.^1248^ The SWCNT-based sensors used for their detection employ the corona phase molecular recognition (CoPhMoRe) approach,^1249,1250^ where amphiphilic polymers wrap SWCNTs to form distinct molecular recognition sites for various types of small molecules. Specifically, to selectively detect 2,4-D, the researchers utilized a range of cationic fluorene copolymers, which were copolymerized with Py (pyridine), Pz (pyrazine), Pm (pyrimidine), or 13-P (1,3-phenyl). On the other hand, for the specific detection of NAA, they employed specialized polymers featuring poly(N-vinylimidazole) (PVI) and poly(4-vinylpyridine) (PVP) as their backbones. This method allows the nanosensors to detect fluctuations in fluorescence intensity, characterized by a quenching response to NAA and an activation response to 2,4-D. A pivotal aspect of this research is the detailed examination of the spatiotemporal distribution of NAA and 2,4-D in spinach leaves (Figure 39). It demonstrates how varying concentrations of NAA, from 1 mM to 100 μM, result in corresponding changes in fluorescence quenching. This indicates the nanosensors’ sensitivity and ability to monitor the uptake and metabolism of NAA within plant tissues. Contrasting this, 2,4-D infiltration showcases a more gradual fluorescence turn-on response over 90 min, highlighting a different uptake and metabolic pattern compared to NAA (Figure 40). These measurements were not limited to laboratory conditions but extended to *in planta* testing across a variety of plant species, including spinach, *A. thaliana*, bok choy, and rice, in diverse environments of soil, hydroponics, and plant tissue culture media. A key finding from these tests was the sensors’ ability to detect the accumulation of 2,4-D in bok choy leaves—a dicotyledonous plant susceptible to 2,4-D—while showing no such uptake in the tolerant monocotyledonous rice leaves. This ability to differentiate between susceptible and tolerant plants is crucial for understanding the transport mechanisms and herbicide resistance in crops. Moreover, these sensors have proved to be effective tools for rapid herbicide susceptibility testing, marking a significant advancement in agricultural technology.

**Figure 41:**
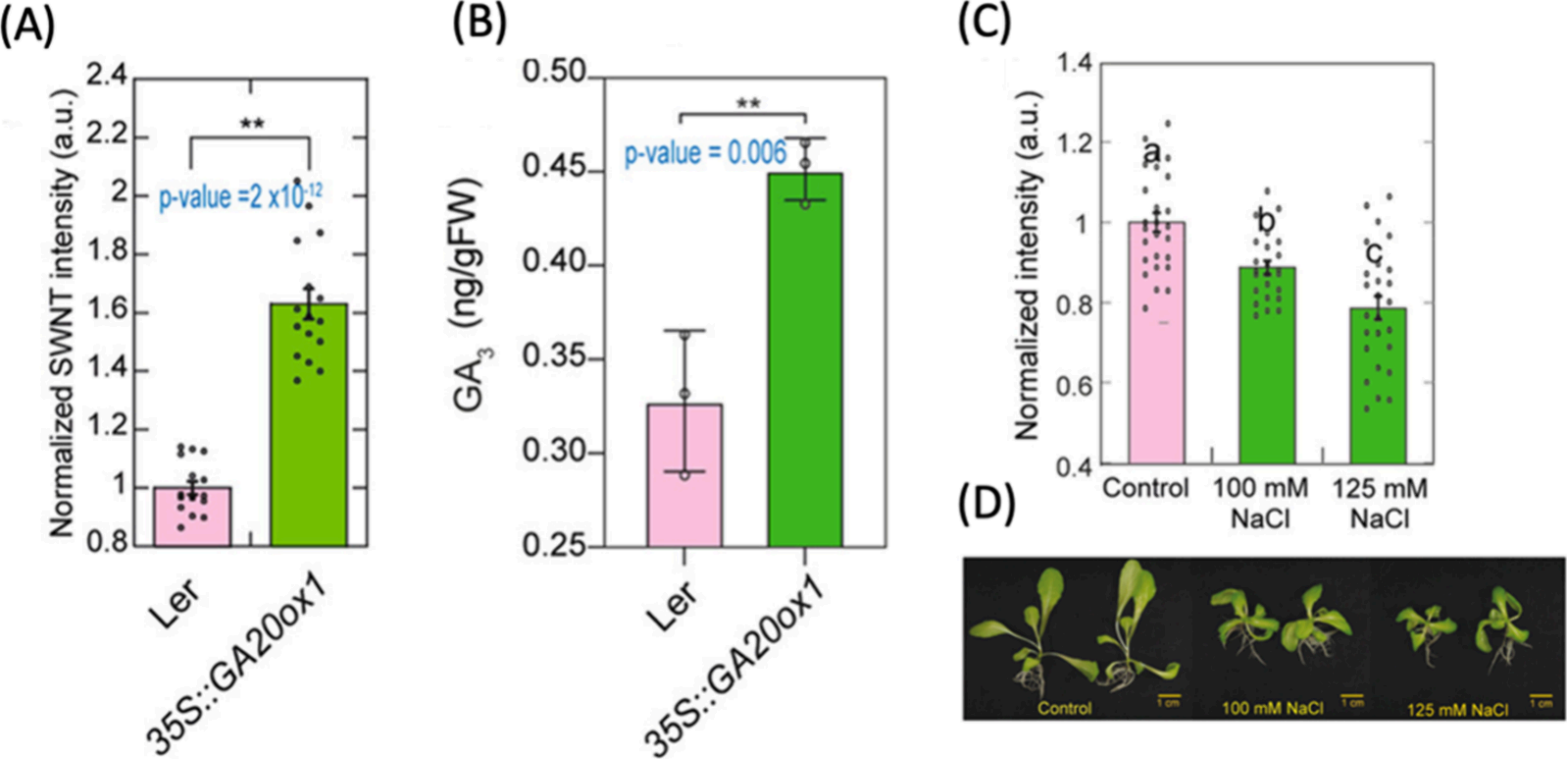
(A) The integrated fluorescence intensity was quantified for both Ler and GA20ox1 seedlings, and then standardized against the fluorescence intensity of Ler seedlings. The resulting graphs display the average standardized fluorescence intensities along with their standard deviations, with each data point represented by dots. (B) Biochemical determination of GA_3_ levels in wild-type (Ler) and 35S::GA20ox1 overexpression lines was conducted. Gibberellins were extracted from seedlings aged 10 days and their concentrations were measured using LC-MS/MS analysis. (C) Normalized fluorescence intensity of GA_3_-SWNT in the roots of lettuce for various NaCl treatments, based on 21–25 data points gathered from six seedlings across two independent experiments. (D) Lettuces at the age of 10 days, treated with either no NaCl or with 100 or 125 mM NaCl for an additional 10 days. Adapted from ref (1251). Copyright 2023 American Chemical Society.

Another plant hormone of interest for detection has been gibberellins (GAs), particularly GA_3_ and GA_4_. These hormones are vital for plant growth and development. Boonyaves et al. employed the CoPhMoRe platform to wrap SWCNTs with polymers composed of styrene-based monomers, namely sodium-styrenesulfonate (S) and vinyl benzyl trimethylammonium chloride (N).^1251^ These monomers were specifically chosen for their interaction with GAs, particularly targeting the carboxyl groups of the GAs as key interaction sites. Using CoPhMoRe SWCNT sensors, researchers were able to detect changes in GA levels.^1251^ The sensors responded to GA_3_ and GA_4_ concentrations ranging from 0 to 150 μM, with detection limits estimated at 542 nM for GA_3_ and 2.96 μM for GA_4_, respectively. In *Arabidopsis* seedlings, these sensors exhibited a pronounced increase in fluorescence intensity following GA_3_ application. Additionally, the study revealed that *Arabidopsis* mutants overexpressing GA20ox1, a key enzyme in GA biosynthesis, displayed higher GA levels than wild-type plants, as detected by the sensors (Figure 41). This differentiation demonstrated the sensors’ capabilities in gauging varying endogenous GA levels in genetically diverse plant lines. This study further explored the impact of environmental stress, specifically salinity, on GA dynamics in *Arabidopsis*. The sensors indicated a decrease in GA levels under high salinity conditions, a change that correlated with reduced lateral root growth (Figure 41). This finding linked GA dynamics directly to the plant’s response to environmental stress. Moreover, the spatial analysis of GA distribution in *Arabidopsis* roots revealed higher concentrations at critical growth points, such as the lateral root bud, emphasizing GA’s role in root development. The study also ventured beyond model plants, with experiments in lettuce showing similar trends in GA reduction under salinity stress, correlating with stunted growth. These findings highlight the substantial potential of CNM sensors in real-time, nondestructive monitoring of GA distribution and dynamics in plants. The ability to provide detailed insights under both normal and stress conditions open up new avenues for agricultural research and crop management.

The continuous advancement of sensor technologies in plant biology research holds the potential to revolutionize agricultural practices, leading to more precise crop management, and fostering a deeper understanding of plant biology. The exploration of a broader range of plant hormones, including cytokines, ethylene, and brassinosteroids, which are pivotal in cell division, stress response, and overall plant development, could unveil a more comprehensive understanding of plant hormone networks and their complex interactions. Such investigations have the potential to pave the way for innovative agricultural strategies and enhance crop resilience. However, despite these promising advancements in this field, there remain critical aspects that warrant further investigation. For instance, the sensors for ABA and JA, while demonstrating excellent sensitivity, are reliant on tissue homogenization, an inherently destructive process. This methodological constraint limits our ability to obtain immediate, spatially resolved insights within the plant tissues, which are essential for understanding dynamic physiological processes and hormone distribution patterns in situ. The reliance on destructive sampling techniques, therefore, poses a significant barrier to fully elucidating the intricate spatial and temporal dynamics of hormone activity within plant structures. Furthermore, other important factors such as the reversibility of the sensors, their long-term stability in plant tissues, their ability to provide spatiotemporal information, and their specificity amidst various environmental factors and other biological molecules also merit further. Addressing these challenges is crucial for advancing our understanding and application of these sensor technologies.

##### 5.3.2.3 ROS/RNS Sensing

There is also an emerging field of study centered around the detection of reactive oxygen and nitrogen species (ROS/RNS) in plants,^1225−1229^ with a particular focus on H_2_O_2_ and NO. ROS play a critical role in plant physiology and their response to stress. For instance, H_2_O_2_ production and accumulation have been observed in plants experiencing various stresses, such as light, heat, salinity, wounding, and pathogen infection.^1252−1254^

In response to the growing demand, Wu et al. have devised a highly sensitive H_2_O_2_ sensor utilizing SWCNTs functionalized with a DNA aptamer that specifically binds to hemin (HeAptDNA-SWCNT).^1225^ This sensor operates through a Fenton-like reaction,^1255^ wherein H_2_O_2_ reacts with hemin, generating hydroxyl radicals that subsequently quench the SWCNT fluorescence. To ensure the sensor’s specificity for H_2_O_2_, tests were conducted to assess its response to stress-associated plant ions, sugars, and hormones, including Ca^2+^, sucrose, glucose, methyl salicylate, abscisic acid, and jasmonate. The results showed that the NIR fluorescence responses of the sensor were largely unaffected by these off-target molecules. Furthermore, the H_2_O_2_ sensing performance of the HeAptDNA-SWCNTs remained robust in the presence of these stress-related molecules, underscoring their selectivity and potential for *in vivo* applications.

**Figure 42:**
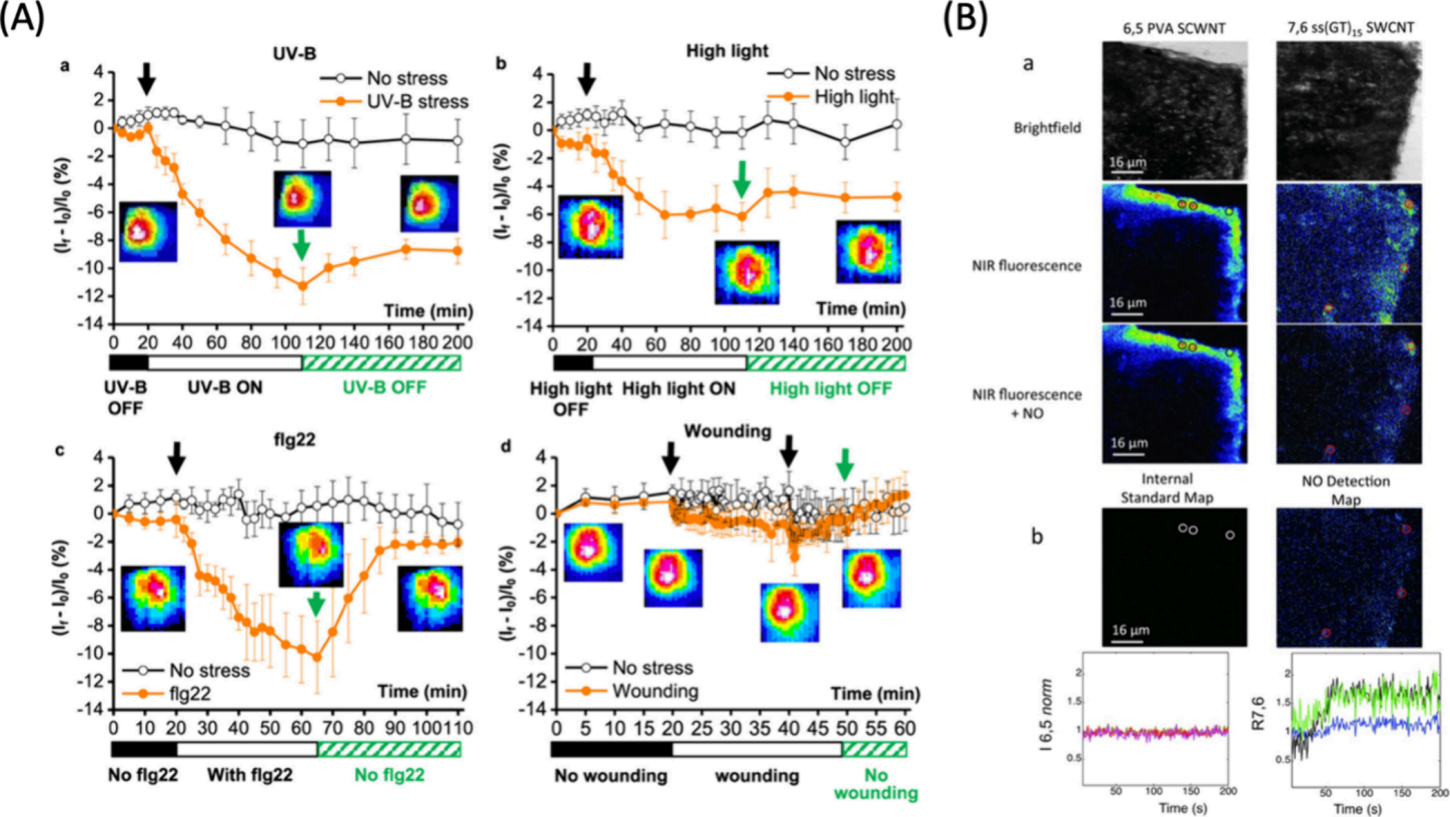
(A) Plant health is monitored real-time using optical techniques by employing H_2_O_2_ nanosensors. The changes in NIR fluorescence intensity of HeAptDNA-SWCNT sensors in leaves (as shown in color map insets) provide information about the initiation of various environmental stresses, such as UV-B radiation, intense light exposure, and stress caused by pathogen-associated peptides like flg22. Adapted from ref (1225) with permission. Copyright 2020 American Chemical Society. (B) The response of a ratiometric sensor to H_2_O_2_ inside leaves is observed *in vivo*. Leaf sections are infiltrated with a ratiometric sensor consisting of a 6,5 ss(AT)_15_ strand and a 7,6 ss(GT)_15_ strand, each with different chiralities. These chiralities are independently imaged using a 785 nm excitation source. The internal standard and H_2_O_2_ detection are represented in maps based on the change in NIR intensity within the leaf section. Adapted from ref (1228). Copyright 2020 American Chemical Society.

Using the HeAptDNA-SWCNTs, they were able to selectively monitor physiological H_2_O_2_ levels (10–100 μM) in *A. thaliana* leaves^1225^ (Figure 42A) in response to several stressors, such as UV-B light (reduction of 11%), high light (reduction of 6%), and a pathogen-related peptide (reduction of 10%), although it does not detect leaf wounding.^1225^

In a complementary study, Lew et al. developed highly sensitive and selective sensors using functionalized SWCNTs for real-time monitoring of H_2_O_2_ caused by wounding.^1227^ This study employed two types of DNA-wrapped SWCNTs: G-SWNTs, which are wrapped in ss(GT) oligonucleotides and show quenched fluorescence in the presence of H_2_O_2_, and A-SWCNTs, wrapped in ss(AT) oligonucleotides, whose fluorescence remains invariant toward H_2_O_2_. This dual system forms a ratiometric platform for *in vivo* H_2_O_2_ detection. Using these sensors, researchers investigated the mechanism and characteristics of the H_2_O_2_ signaling pathway in various plant cultivars and genetic variants. Their findings suggest a significant interaction between H_2_O_2_, electrical, and calcium signaling pathways, which plays a crucial role in regulating plant defense responses following wounding.

The focus on RNS, including NO, has grown alongside the study of ROS. The remarkable capacity of RNS to modulate plant growth, enhance nutrient absorption, and activate disease and stress tolerance mechanisms has attracted significant attention across a wide range of plant species.^1256,1257^ Giraldo et al. developed ratiometric reversible sensors using NIR-SWCNTs coated with corona phases consisting of specific oligonucleotides that selectively recognize NO, H_2_O_2_, or no analyte in *A. thaliana* leaves.^1228^ (Figure 42B). The integration of these nanosensors with plants, facilitated by the unique optical properties of single chirality SWCNTs, holds immense potential for establishing a robust platform for biochemical monitoring, particularly in dynamic field conditions. For example, leveraging the multiplexing capability of the NIR signal emitted by nanosensor-equipped plants could enable the detection of SWCNT fluorescence from remote locations using standoff devices such as NIR cameras, even amidst challenging environmental, chemical, and optical complexities.

The advances in plant nanobionics represent a significant leap in our understanding of plant responses. Researchers’ innovative use of SWCNTs to monitor vital indicators offers new prospects for real-time, remote plant health assessment. However, it is not just H_2_O_2_ and NO that are crucial; other ROS and RNS, such as superoxide radicals, peroxynitrite, and hydroxyl radicals also play an important role in plant processes, as they are respectively produced during photosynthesis and respiration in plant cells. For a more comprehensive study of plant stress, it is essential to develop probes that can detect these additional types of reactive species. Given that these ROS have shorter half-lives and are less able to travel through cellular membranes, sensors designed for this purpose will need to be highly efficient in penetrating plant cells and possess rapid response times. While these technologies using CNMs are still evolving, their development underscores the transformative potential of nanobionics in shaping a more resilient future for agricultural systems.

##### 5.3.2.4 Other Sensing Applications

CNMs are also employed for detecting heavy metals and contaminants, covering a broad spectrum of chemicals. These include, but are not limited to, As^3+^, Ag^+^, Cr^4+^, Hg^2+^, Fe^3+^, Pb^2+^, Cu^2+^, Co^2+^, trinitrotoluene (TNT), trinitrophenol (TNP), malathion, and glyphosate. Table 7 presents the CNMs utilized for environmental monitoring in various settings such as rivers, lakes, and land. However, due to the extensive research in this field and the scope of this review, our discussion focuses solely on CNMs used in biological samples, such as plants and animals.

**Figure 43:**
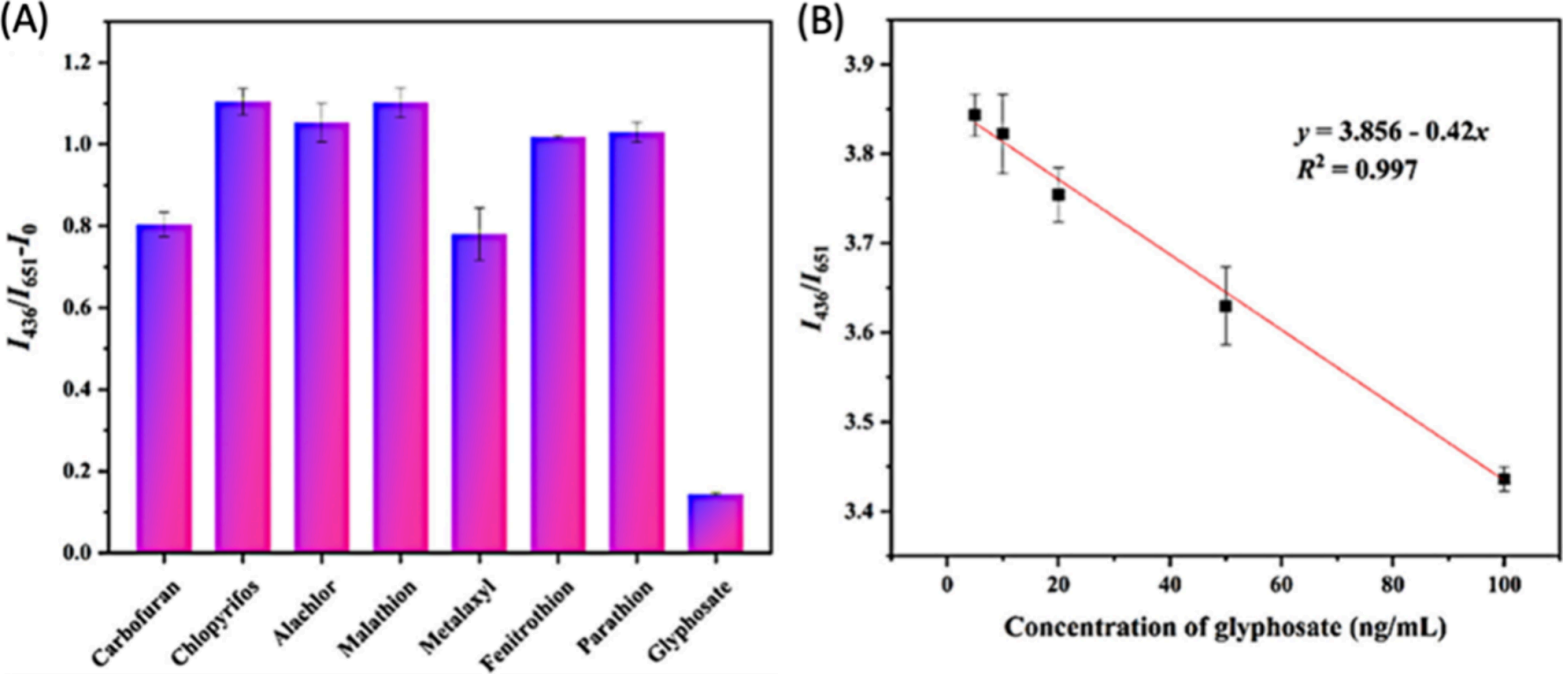
(A) Ratiometric fluorescence responses of N-CDs@SiO_2_@BSA-AuNCs with Cu^2+^ in response to glyphosate and seven other pesticides at a concentration of 100 ng/mL, and (B) the linear correlation between the *I*_436_/*I*_651_ intensity ratio and glyphosate concentrations ranging from 5 to 100 ng/mL. Adapted from ref (1258). Copyright 2023 American Chemical Society.

In the case of detecting contaminants, Ma et al. developed a ratiometric fluorescent nanosensor aimed at detecting glyphosate,^1258^ a widely used herbicide known for its nonselective, broad-spectrum weed control properties. This sensor integrates nitrogen-doped CDs enveloped in mesoporous silica spheres (N-CDs@SiO_2_) with bovine serum albumin-stabilized gold nanoclusters (BSA-AuNCs). The core–satellite configuration of this sensor facilitates dual-emission fluorescence at 436 and 651 nm using a single excitation wavelength of 360 nm. This mechanism operates on a “signal on–off–on” principle, where the fluorescence of BSA-AuNCs is initially quenched by addition of Cu^2+^ and then restored when glyphosate forms complexes with Cu^2+^, altering the fluorescence intensity ratio at these wavelengths. Notably, the sensor is highly selective in distinguishing glyphosate in the presence of other pesticides, including carbofuran and alachlor (Figure 43). The probe can detect glyphosate across a wide concentration range of 5–100 ng/mL (Figure 43), with the lowest detection limit of 3.4 ng/mL. Its selectivity and sensitivity make it particularly effective in complex environmental samples. Using malt as the tested system, the researchers achieved glyphosate recoveries ranging from 94.81% to 101.61%. The sensor’s innovative design, combined with its validated efficacy in real-world samples, positions it as a tool for environmental monitoring and food safety.

Another example of CNM application in contaminant detection involves carbendazim, a widely used fungicide from the benzimidazole chemical class. This substance effectively controls a wide range of fungal diseases in crops and serves as a preservative in paints, textiles, and paper products. Ruiyi et al. developed a sensitive fluorescence probe, with a detection limit of 6.1 × 10^17^ M.^1259^ This system utilizes serine and histidine-functionalized graphene quantum dots (Ser-GQD-His), which display blue and yellow fluorescence under varying excitation wavelengths. The mechanism is based on the interaction between carbendazim and a specially designed aptamer. When the aptamer binds with carbendazim, it prompts the release of an assistant strand (AS), initiating a DNA recycling amplification process. This process leads to the formation of numerous G-quadruplex/hemin (G4/hemin) DNAzyme composites. These composites catalyze the transformation of externally introduced o-phenylenediamine (OPD) into the fluorescent molecule 2,3-diaminophenazine (DAP). The presence of DAP then quenches the fluorescence of Ser-GQD-His and simultaneously enhances its own fluorescence, thus generating a strong and measurable fluorescence signal. The practical application of this probe has been successfully demonstrated in the detection of carbendazim in tomato plants, achieving a recovery rate between 95 to 105%.

Recently, a different type of quantum dots has been used for detecting malathion, which is an organophosphate insecticide that is commonly applied to control mosquitoes and a variety of insects. Liang et al. developed a dual-mode sensor consisting of CQDs and gold nanoparticles (GNPs) for the efficient detection of malathion in cabbage.^1260^ This sensor’s construction leverages the fluorescence properties of CQDs and the colorimetric response of GNPs, enabling it to visually detect malathion through changes in both fluorescence intensity and color. The mechanism by which the presence of malathion enhances the fluorescence of the CQDs-GNPs and causes a color shift from red to blue is due to a specific interaction between malathion and the nanocomposite sensor. When malathion is present, it induces the aggregation of the GNPs within the sensor. This aggregation disrupts the fluorescence quenching normally caused by the close proximity of the GNPs to the CQDs. As the GNPs aggregate, they move away from the CQDs, which restores the fluorescence of the CQDs. The aggregation of the GNPs also leads to a visible colorimetric change; the solution’s color changes from red to blue, which can be observed with the naked eye. Importantly, the probe exhibits high specificity: when tested against various other compounds typically found in cabbage, such as isocarbophos, dimethoate, and dichlorvos, the sensor’s response to malathion was distinctly stronger, indicating selective detection capability even when the interfering substances were present at higher concentrations than malathion. Furthermore, the sensor demonstrates high accuracy in real-world applications, where its effectiveness was validated in cabbage samples by detecting varying concentrations of malathion. The sensor’s recovery rates range from 89.9% to 103.4% in fluorescence detection and 88.7% to 107.6% in colorimetric detection.

Besides detecting pesticides, sensing toxic heavy-metal pollutants is also crucial because these pollutants can cause severe environmental damage and pose significant health risks to humans and wildlife. Timely and accurate detection helps in implementing effective remediation strategies and preventing long-term ecological and health impacts.

**Figure 44:**
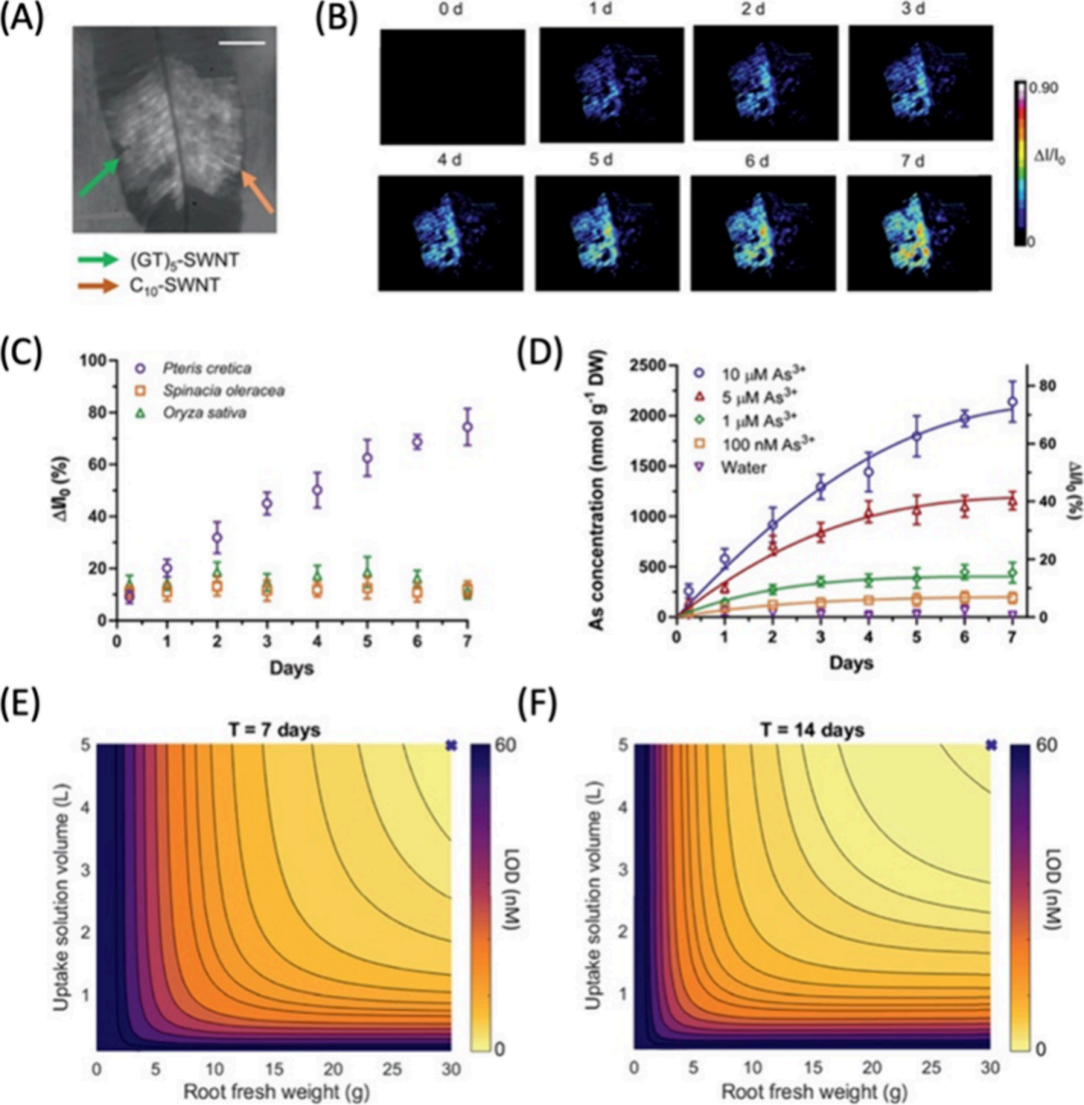
(A) Bright-field visualization of *Pteris cretica* leaf with (GT)_5_-SWCNT and C_10_-SWCNT, excited at 785 nm. Scale bar = 0.5 mm. (B) Sequential images depicting intensity variation in nanosensors following arsenite exposure, with timestamps postarsenite application via roots. (C) Comparison of fluorescence intensity shifts in SWCNT nanosensors within spinach, rice, and *Pteris cretica* under 10 μM arsenite-treated root medium. (D) Arsenite levels in *Pteris cretica* leaves subjected to varying arsenite concentrations (10, 5, 1, 0.1 μM) and deionized water in the root medium. (E) Contour plot of sensor’s detection limit after 7 days. Cross indicates the detection limit of 4.7 nM (0.6 ppb). (f) Contour plot of sensor’s detection limit after 14 days. Cross indicates the detection limit of 1.6 nM (0.2 ppb). Adapted with permission from ref (1261). Copyright 2021 Wiley.

For example, in a recent study, Lew et al. utilized plant nanobionic sensors for the real-time detection of arsenite, which is an arsenic form predominantly found in anaerobic conditions such as paddy soils of crops.^1261^ Their approach involved embedding NIR fluorescent nanosensors, specifically SWCNTs, into plant tissues. These nanosensors, wrapped in single-stranded DNA rich in guanine and thymine nucleotides, selectively respond to arsenite, leveraging the unique binding properties of these nucleotides that form strong hydrogen bonds with arsenite’s hydroxy groups. Their study underscores the efficacy of these sensors in the Cretan brake fern (*Pteris cretica*), a species known for its arsenic hyperaccumulating abilities. The ratiometric sensor response for *P. cretica* was consistently higher than that observed in other plant species like spinach or rice. This heightened response in the fern, shown in a significant increase of 74% relative to the initial level, is indicative of its superior arsenite accumulation capability (Figure 44). Experiments demonstrated that upon exposure to arsenite, the ferns exhibited a steady increase in the fluorescence intensity of the embedded SWCNTs over a seven-day period. This increase was relative to the initial values, providing a dynamic quantitative measure of arsenite uptake. Further, the study explored a kinetic model to describe the nanosensor response to arsenite uptake in *P. cretica.* This model helped translating the changes in sensor fluorescence intensity into actual concentrations of arsenite within the plant tissues. Using the natural ability of *P. cretica* ferns to hyperaccumulate and tolerate exceptionally high levels of arsenic, the sensors can integrate arsenite signals over extended periods, which in turn help detect arsenic levels as low as 0.6 parts per billion (ppb) after 7 days, and 0.2 ppb after 14 days (Figure 44). The research affirmed the potential of integrating optical nanosensors with living plants, enabling them to function as sensitive and selective detectors for environmental monitoring.

Beyond detecting heavy metals in plants, CNM research extends to the important area of environmental monitoring, focusing on the sensing of divalent metal cations in aquaculture. In one study, Gong et al. uses SWCNTs functionalized with DNA corona phases (CPs).^1262^ These optical sensors are designed to identify harmful metal ions such as mercury, lead, chromium, and manganese, which are notable contaminants in aquaculture environments. The sensor operates on the principle of photoluminescence changes, triggered by the interaction of DNA-SWCNTs with specific metal ions. The DNA CPs serve as molecular recognition elements, allowing the SWCNTs to selectively bind to different divalent metal cations and induce changes in photoluminescence intensity and wavelength. The researchers conducted a series of experiments to understand the interactions between a range of divalent metal cations and various DNA CPs. Critical experimental conditions, including the ionic strength, buffer dilution kinetics, laser excitation power, and analyte response kinetics were optimized to maximize the sensor’s accuracy and reliability. A notable finding was the sensor’s ability to operate in two distinct sensing states, achieved by adjusting the pH levels of the solution. This pH-dependent response is crucial for differentiating among various metal ions, enhancing the sensor’s versatility. Furthermore, the researchers developed a portable version of the sensor for aquaculture applications, where it detected mercury levels in fish tissue extracts, a crucial test given the prevalence of mercury contamination in aquaculture products. The sensitivity of this DNA- SWCNT sensor was 33 nM. Given that the typical mercury concentration in cod, post-extraction, is around 1.1 μM, this suggests the method could be highly effective for monitoring and ensuring safety of seafood products at a commercial scale.

He et al. also introduced a ratiometric fluorescence sensor for detecting mercury in seafood,^1263^ based on nitrogen-doped carbon dots (N-CDs) sensitized with Terbium(III) and 2,6-pyridinedicarboxylic acid (DPA). The sensor operates on a mechanism where N-CDs are coordinated with DPA-modified Terbium ions (Tb-DPA). This coordination results in a sensor that exhibits dual-emission fluorescence: one at 436 nm from the N-CDs and another at 543 nm from the Tb-DPA complex. The detection mechanism is centered on the interaction between mercury ions and the N-CDs. When mercury ions are present, they interact with the oxygen-containing functional groups on the N-CDs, facilitating an electron transfer process. This interaction leads to the quenching of the N-CDs’ fluorescence at 436 nm without affecting the fluorescence emission of the Tb-DPA complex at 543 nm. The ratiometric measurement is based on the relative change in the intensity of these two emissions, providing a reliable indication of the presence and concentration of mercury ions. One of the most remarkable features of this sensor is its selectivity. The sensor shows the ability to selectively detect Hg^2+^ ions in the presence of a wide range of other metal ions, including Ag^+^, Cu^2+^, Mn^2+^, among others. The selectivity is crucial for applications in complex matrices like seafood, where various metal ions can be present. In addition to its specificity, the sensor has a low detection limit of approximately 37 nM. In practical applications, it has successfully quantified mercury levels in different seafood samples (e.g.,prawn, seaweed, octopus, and large yellow croaker).

### 5.4 Tracking of Delivery with CNM Fluorescent Probes

Delivery of biologics using CNMs has been a popular application in nanomedicine and bioengineering. Advances in this area are summarized in numerous comprehensive reviews.^1266−1272^ Even though majority of the delivery applications of CNMs use non-fluorescent nanomaterials, unique properties of CNMs enable novel ways of cell entry and cargo delivery that are not achievable by other approaches. Given the focus of this review is on the CNM fluorescent probes, this section is kept brief.

#### 5.4.1 Delivery of Drugs in Nanomedicine

Recently, novel fluorescent CNMs were established for dual intracellular imaging and drug delivery. For instance, gluten was used as a carbon source to synthesize highly fluorescent carbon nanorings with a quantum yield of 47.0% that when loaded with the model drug doxorubicin showed high level of apoptosis in many cancer cells lines.^1273^ Similarly, nitrogen and sulfur co-doped photoluminescent CDs synthesized from κ-carrageenan and folic acid were targeted to cancer cells using folic acid receptors to effectively deliver an anticancer drug capecitabine.^1274^

Besides imaging and drug delivery, more modalities have recently been added to CNMs, such as leveraging photothermal effects of certain CNMs by heating them with a NIR light resulting in an on-demand drug release.^1275^ Another combination therapy example is the usage of CNMs that inherently have antimicrobial properties for the delivery of antibiotics to address the increasing antibiotic resistance issue. For this, carboxylic acid-functionalized MWCNTs were developed for the delivery of kanamycin and streptomycin to treat *Mycobacterium fortuitum* infection.^1276^ Similarly, isoniazid and fluoxetine-conjugated MWCNTs showed effective delivery of the drug fluoxetine for combined therapy against *Tuberculosis.*(1277)

Recent years have also seen a surge of studies investigating the drug–CNM interactions at the theoretical and modeling levels using the quantum theory of atoms in a molecule (QTAIM) method, electron localization function (ELF) calculations,^1278^ DFT,^1279^ and molecular dynamics.^1280,1281^ These studies provide fundamental understanding of drug-CNM adsorption/desorption dynamics and complex stability, which are crucial for the future successful design of CNM medicines.

#### 5.4.2 Gene and Protein Delivery

The delivery of genes and proteins is the first step for gene therapy and genetic engineering of multicellular organisms, such as animals and plants.^1282−1290^ For an overview of CNM applications in biomolecule delivery to plants, refer to the Landry et al.^1291^ and to mammalian systems, refer to Hossein et al.^1292^ Below, we discuss the advancements developed after the publication of these reviews. It is important to note that some CNM delivery vectors that are covered in this section are not fluorescent due to their chemical modifications for cargo loading.

**Figure 45:**
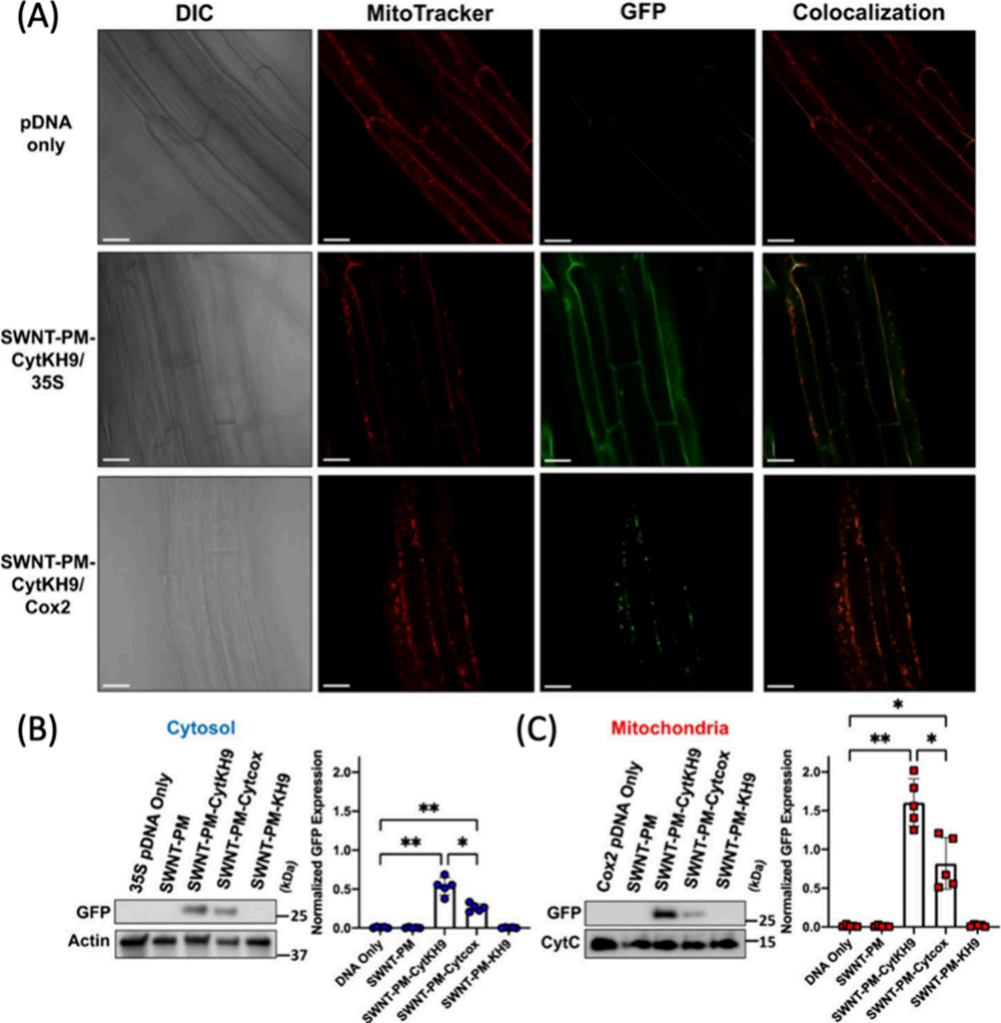
(A) Confocal laser scanning microscopy is utilized to determine GFP expression 18 h postinfiltration of two different plasmid constructs *pDONR-35S-GFP* with a 35S (nuclear) promoter and *pDONR-Cox2-GFP* with a cox2 (mitochondrial) promoter delivered with SWCNT-PM-CytKH9. (B, C) Quantification of protein expression via SWCNT-cytKH9 in plants by Western blotting shows GFP protein presence in both the cytosol (b) and mitochondria (c) 18 h postinfiltration. Adapted with permission from ref (1293). Copyright 2022 Nature.

In case of plant gene delivery, Law et al. decorated SWCNTs with cytochrome c oxidase subunit IV (cytcox) and cationic lysine and histidine repeat cell penetrating peptides for the targeted delivery of DNA into the mitochondria of *A. thaliana* seedlings.^1293^ These modified SWCNTs did not have fluorescence, so their uptake to mitochondria was verified via Raman microscopy. Introduction of genetic material using SWCNTs resulted in a 30-fold increase in the expression of delivered DNA when compared with prior work using cell penetrating peptides only, as well as efficient homologous recombination into mitochondrial genome with no cytotoxicity. Controlled expression of GFP from the delivered plasmid was observed in *A. thaliana* by utilizing either a constitutive promoter (35S) or a mitochondria specific promoter (cox2), shown via both confocal imaging (Figure 45A) and Western Blot (Figure 45B,C).

Other methods using biorecognition peptides on CDs and CNTs have been shown to allow access to cellular organelles, such as chloroplasts. For example, Santana et al. developed PEI-coated SWNCTs and cyclodextrin-coated CDs, both of which had chloroplast targeting peptides for the selective targeting of *A. thaliana* chloroplasts.^1294^ When compared to CNMs without chloroplast targeting peptides, the efficiencies of modified CNMs improved from 47% to 70% and 39% to 57% for CD and SWCNTs, respectively.

**Figure 46:**
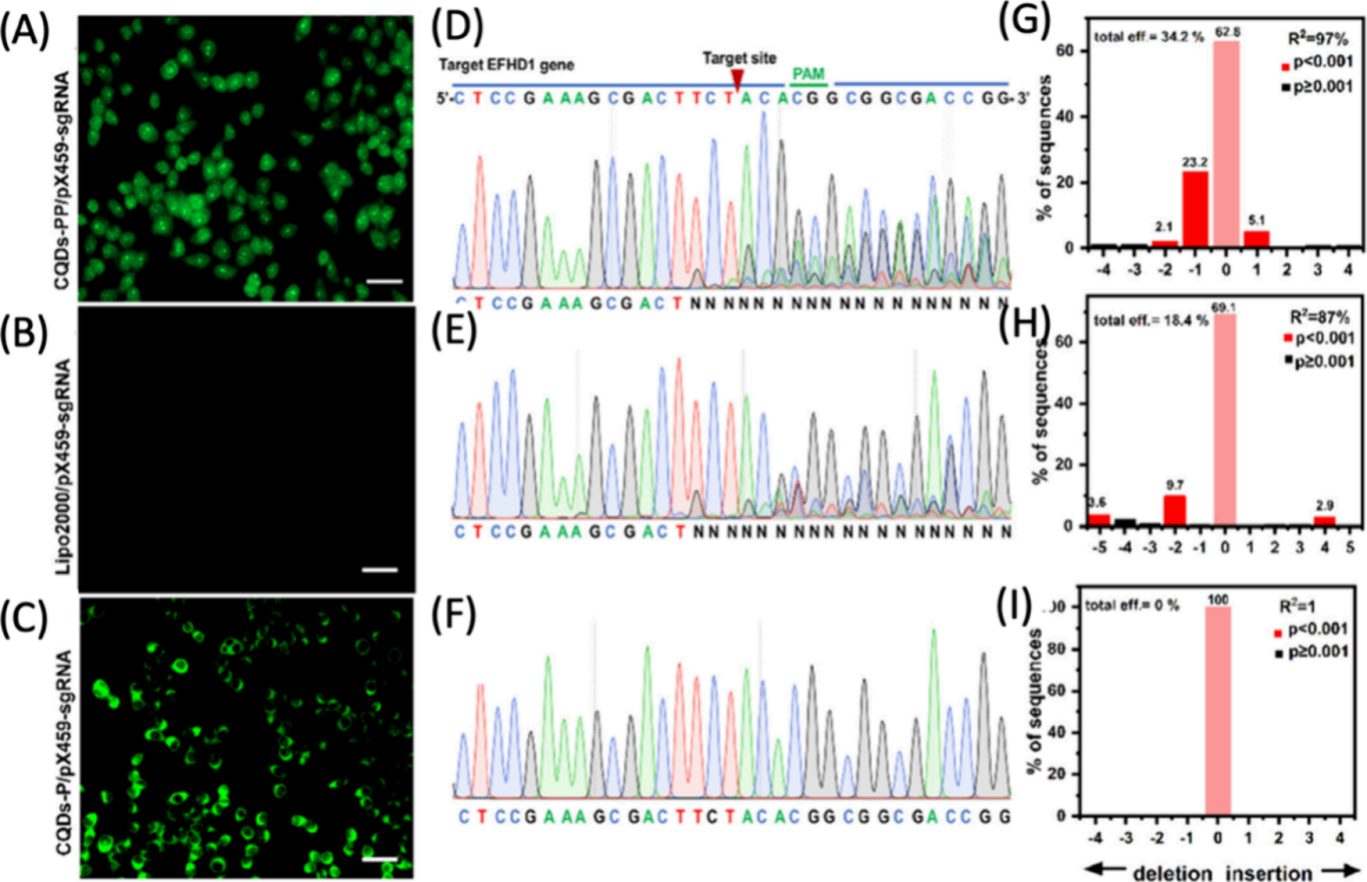
(A–C) The tracking of CQD-PP (A), Lipo2000 (B), and CQD-P (C) through fluorescence microscopy. (D–F) Insertion/deletion of nucleotides utilizing CQD-PP/pX459-sgRNA, Lipo2000/pX459-sgRNA, and CQD-P/pX459-sgR, respectively. (G–I) The quantification of gene editing efficiencies for CQD-PP/pX459-sgRNA (34.2%), Lipo2000/pX459-sgRNA (18.4%), and CQD-P/pX459-sgR(0%) of *EFHD1* gene in HeLa cells. Adapted with permission from ref (1296). Copyright 2022 Royal Society of Chemistry.

In the case of mammalian delivery, CDs have been recently used to deliver genes into *in vitro* mammalian cells, where Hashemzadeh et al. developed cationic PEI and arginine-functionalized CDs for the delivery of CRISPR plasmids into the HEK 293T-GFP cells to effectively knock out the GFP gene as a proof-of-concept study.^1295^ CDs offer significant benefits due to their inherent photoluminescent properties and their ability to function both as nanocarriers and as a fluorescent probe. Zhai et al. used CDs for the delivery and tracking of a CRISPR plasmid into HeLa cells,^1296^ where the plasmid targeted the *EFHD1* gene that is associated with various diseases. CDs that were synthesized from PEI, PEG, and citric acid (CQDs-PP) demonstrated an efficient nuclear uptake (Figure 46A) and editing efficiency of 34.2% (Figure 46D,G) as compared to a common transfection agent Lipo2000 with editing efficiency 18.4% (Figure 46B,E,H). Authors have also compared the CQDs-PPs with other CDs made from the PEI and citric acid without PEG, which were unsuccessful in causing editing given their inability to enter cell nucleus (Figure 46C,F,I).

**Figure 47:**
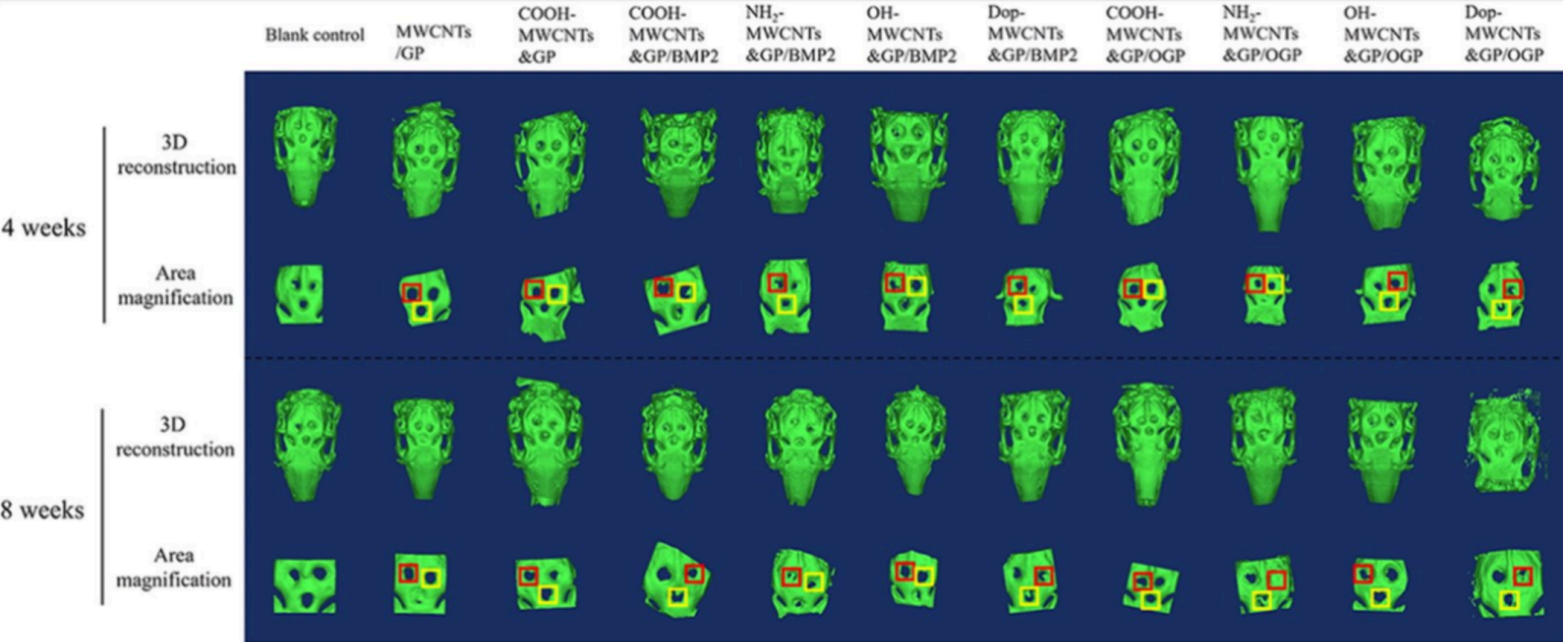
A 3D reconstruction of the rabbit calvarial defects conferred to measure the regeneration of bone for chemically distinct (neat, −COOH, −NH_2_,–OH, dopamine) MWCNTs (red) and graphene (yellow) particles decorated with BMP2 and/or OGP peptides at 4 and 8 weeks. The reconstruction is utilized to measure the growth of new bone as demonstrated by all the CNM conjugate implantations. Adapted with permission from ref (1297). Copyright 2022 Elsevier.

Compared to the DNA delivery, there are only a few reports of protein delivery using CNMs. Du et al. assessed the efficacy of catechol, carboxyl, amino, and hydroxyl-modified MWCNTs and GO for the delivery of morphogenic protein-2 (BMP-2) and osteogenic growth peptides (OGP) into both mouse and rabbit models to track induced osteogenesis.^1297^ BMP-2 and OGP-modified MWCNTs induced osteogenesis more effectively in the ectopic osteogenesis rat models when compared to GO derivates (Figure 47). Similarly, osteogenesis in the calvarial defect rabbit models was greater with MWCNTs compared to GO. Moreover, Silvestre et al. used MWCNTs for the delivery of rMSP1a proteins to Holstein cattle and measured their immune response against *Anaplasma marginale.*(1298) Results demonstrated that vaccinated cows with MWCNT-rMSP1a presented an increase in Natural Killer (NK), CD4^+^, and CD8^+^ cells. In addition to the elevated immune cells, an increase in IgG, IgG1, and IgG2 anti-rMSP1a were present after two rounds of vaccinations. This study utilized CNMs for the delivery of rMSP1a in place of conventional vaccines and was able to elicit a strong immunogenic response *in vivo* in cattle.

## 6 Biomedical and Environmental Translation of CNMs

With the rising prominence of CNMs both in the biomedical and environmental fields, it is crucial to gain a comprehensive understanding of their *in vitro* and *in vivo* cytotoxicity effects (Section 6.1), along with their environmental accumulation and fate (Section 6.2). The translation of CNMs from the lab to the clinic and fields will also depend on scale-up, economical, and regulatory considerations (Section 6.3).

### 6.1 Cytotoxicity of CNMs

#### 6.1.1 *In Vitro* Cytotoxicity

Common methods of studying the *in vitro* cytotoxic effects of CNMs include tracking of mitochondrial function, mitochondrial membrane permeability, cellular membrane breakdown, uptake of neutral red dye by lysosomes, and the generation of ROS in cultured animal cells.^1299^ Below, we describe these methods, discuss how they provide valuable information to help evaluate the cytotoxicity of CNMs, and summarize recent *in vitro* cytotoxicity study results of CNMs. For a more extensive review on *in vitro* CNM cytotoxicity, readers are encouraged to refer to Yuan et al.^1300^ and for a CNT-specific review on toxicity and regulation, readers should refer to Heller et al.^1301^

##### 6.1.1.1 Methods Used for Determining Cytotoxicity

Tracking mitochondrial function is often used to measure both the metabolic activity and viability of cells. Since live cells depend on mitochondrial ATP synthesis for many cellular functions, mitochondrial function is commonly tracked via [3-(4,5-dimethylthiazol-2-yl)-2,5-diphenyltetrazolium bromide] (MTT) assays to determine cellular viability. This assay depends on the reduction of water-soluble MTT to water insoluble formazan via viable mitochondrial dehydrogenases. Consequently, by measuring the production of formazan through UV–vis spectroscopy, mitochondrial metabolic activity and viability of cells can be determined. Tracking mitochondrial permeability via monitoring the mitochondrial membrane potential can be used to determine cellular viability, since changes in mitochondria membrane potential are indicative of the start of apoptosis. This is commonly monitored via a fluorescent reporter, 5,5,6,6′-tetrachloro-1,1′,3,3′ tetraethylbenzimi-dazoylcarbocyanine iodide (JC-1).^1302^ When cationic JC-1 molecules are present in high concentrations in the inner negatively charged mitochondria, they fluoresce red due to the aggregation of JC-1. However, when the concentration of JC-1 aggregates is low in mitochondria, the monomeric JC-1 accumulates in the cytosol and fluoresces green. Inhibition of JC-1 transport into mitochondria is a sign of compromised mitochondria.

Neutral red dye absorption in live cell lysosomes provides an estimate of live cells present. This assay utilizes the selective trapping of neutral red dye in viable cell lysosomes. After extraction of dye from lysosomes, UV–vis spectroscopy can be utilized to determine neutral red amount which is proportional to live cells. On the other hand, Trypan Blue is utilized for the staining of dead cells. Plasma membrane impermeable Trypan Blue is only internalized by cells when their membranes are compromised, and thus dead cells can be quantified to understand cytotoxicity *in vitro*. Similarly, tracking cellular membrane permeability is conducted via lactate dehydrogenases (LDH) assays. This assay utilizes the LDHs typically found in the cytoplasm as a reporter for lysed cell membranes. By conversion of lactase to pyruvates and the reduction of NAD+ to NADH, NADH is then able to activate the inactive luciferin to a luminescent luciferin probe. Luciferin can then be measured to quantify the luminescence caused by compromised cellular membranes. Lastly, the Cell Counting Kit (CKK-8) similarly utilizes the conversion of 2-(2-methoxy-4-nitrophenyl)-3-(4-nitrophenyl)-5-(2,4-disulfophenyl)-2H-tetrazolium salts to a water-soluble formazan dye that can be quantified to determine cellular viability.

The detection of excess ROS is observed by 2′,7′-dichlorodihydrofluorescein diacetate (DCFH-DA) assay. This assay utilizes the conversion of DCFH-DA to 2′,7′-dichlorofluorescein (DCF) via oxidation caused by excess ROS. DCF can then be measured via UV–vis to determine the relative concentration of ROS present in the treated cell. Measuring ROS is important because excess ROS cause oxidative stress in cells and can ultimately lead to the damage of nucleic acids (DNA), lipids (cell membrane), and proteins (enzymes), all of which are crucial for cellular viability.^1303^

The following sections summarize some of the recent *in vitro* cytotoxicity results of several CNM types that either performed a systematic and comprehensive study, or revealed an interesting finding. However, there exists a large breadth of old and newer literature in this area, which will not be possible for us to discuss all here. Nevertheless, in summary, some of these findings are contradictory because most studies do not control or report all the needed information on CNMs, biological systems, and treatment conditions. Therefore, going forward in the field, it is crucial to develop better, more comprehensive, and standardized CNM characterization and treatment strategies for accurate understanding of CNM cytotoxicity.

##### 6.1.1.2 Carbon Nanotubes (CNTs), Carbon Nanocones (CNCs), and Carbon Nanohoops (CNHs)

**Figure 48:**
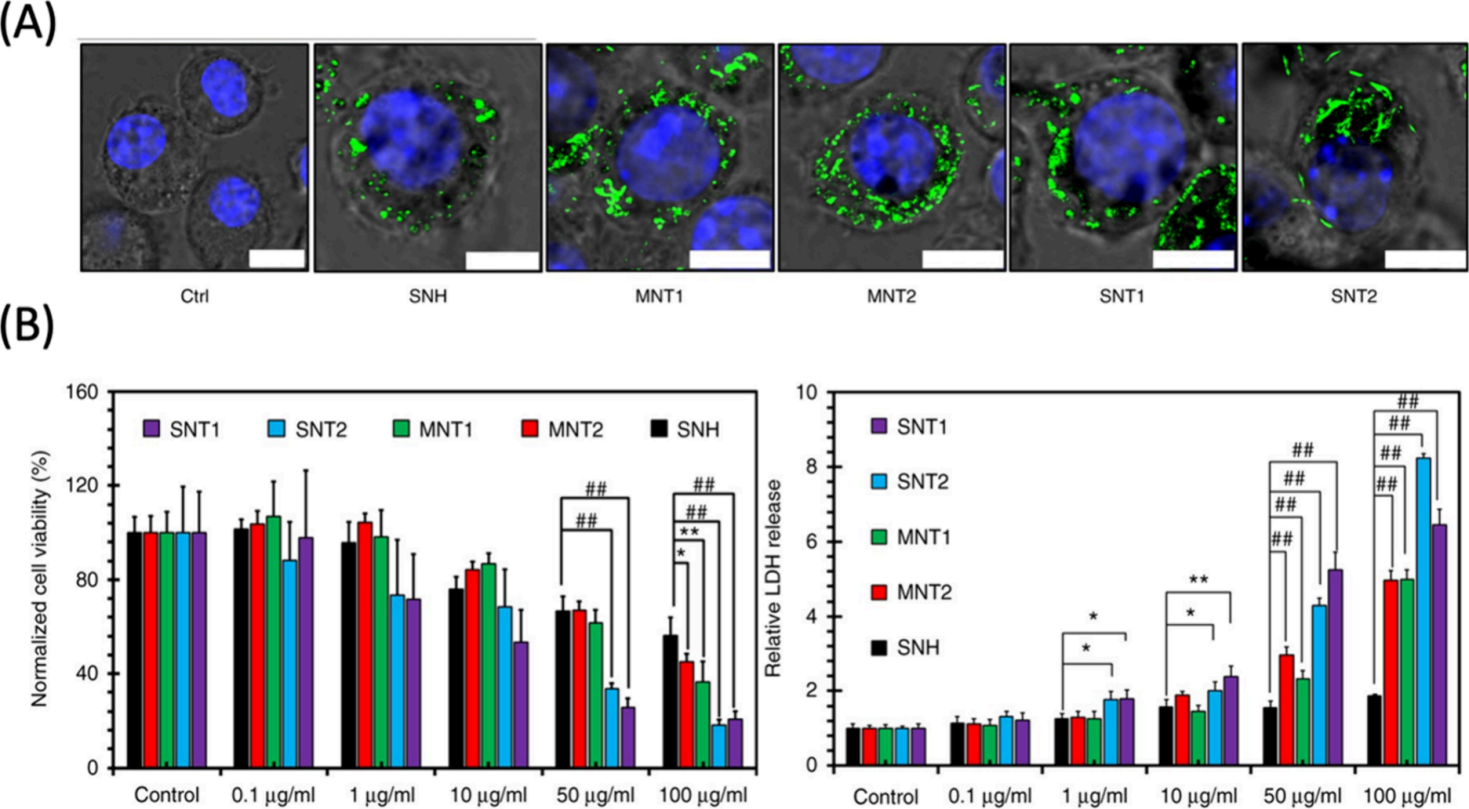
(A) Confocal images of cells after nanocarbon incubations. Intracellular nanocarbons were detected by laser reflection (LR) technology and shown with pseudo green color showing less cell entry of SNHs. Scale bar = 10 μm. (B) Cellular viability comparisons of different nanocarbons detected by a MTT assay (left) and LDH release investigations (right) (*n* = 5). Adapted with permission from ref (1304). Copyright 2018 Nature.

He et al. performed a systematic study to compare the cytotoxicity of five nanocarbons: two SWCNTs, two MWCNTs, and one single-walled carbon nanohorn (SNH).^1304^ They conducted a careful characterization of many CNM properties and their effects on macrophages. SNHs had monomer diameter of 2–5 nm and length of 10–20 nm. SWCNTs had a diameter of 1–2 nm and lengths of 0.5–2 μm for one batch and 2–5 μm for the second batch. MWCNTs had a diameter of 20–30 nm and lengths of 0.5–2 μm for one batch and 2–5 μm for the second batch. The pristine dry CNMs were characterized with Raman, FTIR, thermogravimetric analysis (TGA), XPS, and inductively coupled plasma mass spectrometry (ICP-MS). Then, they were all dispersed in 0.5% (w/v) BSA containing PBS, and further characterized with DLS, TEM, and SEM. The results showed that SNH exhibited unique a cone-like structure and an extremely small aspect ratio, which are significantly different from the rod shapes of high-aspect-ratio nanotubes. Macrophages internalized 2-fold lower amounts of SNH by phagocytosis compared to CNTs (Figure 48A). Third, MTT and LDH assays revealed that SNH caused lower cytotoxicity than four types of CNTs (Figure 48B). It was further observed that necrosis was the main mode of dead cells, tested by propidium iodide (PI) staining resulting in a ∼23% death in SNH-treated samples, compared to the >40% cell death observed in CNT-treated samples. He et al. further elucidated the molecular mechanism behind the CNM cytotoxicity, revealing that CNTs cause cleavage of PARP and CASP-3, which are two hallmarks of apoptosis. This study also investigated many other cytotoxicity metrics that are not discussed here,^1304^ but in summary, it represents an important advancement in the field of studying CNM cytotoxicity as it performs a thorough characterization of many material properties and associated biological responses aiming to eliminate nuanced and skewed toxicity conclusions of insufficiently characterized nanomaterials.

Beyond pristine CNMs in the above study, other research has compared the cytotoxicity of carboxylic acid-functionalized CNTs.^1305−1308^ One example of this is done by Aminzadeh et al.,^1308^ where they studied the reproductive toxicity of COOH-SWCNTs and COOH-MWCNTs in an *in vitro* setup on human spermatozoa. The results showed that neither CNTs had caused increased cell death at a concentration range of 0.1–100 μg/mL when incubated with sperms at 37 °C for 5 h as shown by the MTT test. However, sperm motility was significantly attenuated in a dose-dependent manner. Also, measuring the ROS/RNS amounts revealed increased levels of ROS production in human spermatozoa with both CNT types. This study reveals the importance of how surface functionalization can affect cytotoxicity behavior of CNMs, and that measuring only cell death is not a sufficient metric for comprehensive understanding of CNM effects on biological systems. Studies are highly limited on the cytotoxicity of the newer CNM type carbon nanohoops (CNHs). White et al.^88^ demonstrated that water-soluble disulfonated [8]CPP at the working concentration of ≤10 μM was not toxic to the HeLa cells. Future studies are needed to determine cytotoxicity of different size CPPs, heteroatomic CPPs, and their other chemical modifications on diverse biological systems, especially including noncancerous cell lines.

##### 6.1.1.3 Carbon Dots

**Figure 49:**
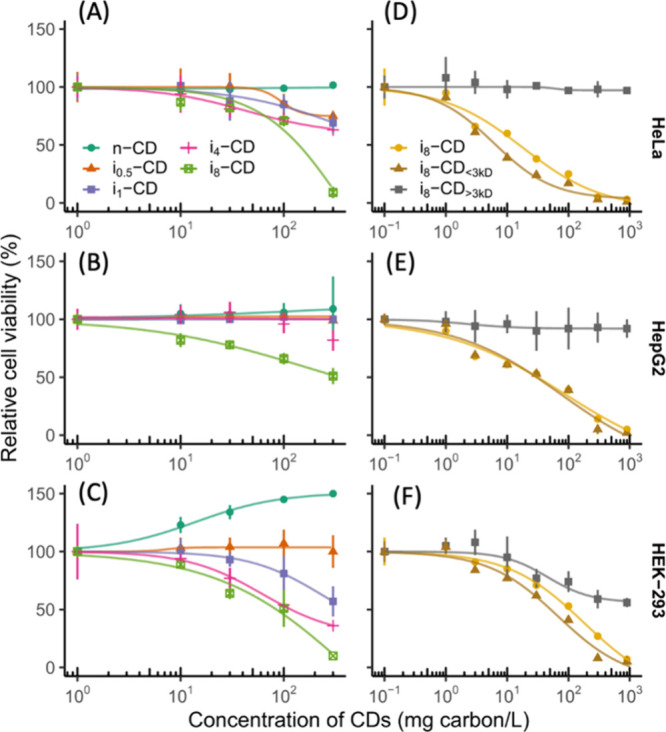
(A–C) Relative cell viability of three cell types (HeLa [a], HepG2 [b], HEK-293 [c]) at 24 h postincubation with 5 samples (n-CD, i_0.5_-CD, i_1_-CD, i_4_-CD, i_8_-CD) individually at different concentrations (0, 10, 30, 100, and 300 mg carbon/L), where n-CD is the nonirradiated CDs and *n* in i_*n*_-CD indicates the number of days the CDs were irradiated with 60 μmol photons/m^2^/s. Trends depict decreased cell viability with longer irradiation times. (D–F) The relative cell viability of three cell types (HeLa [d], HepG2 [e], HEK-293 [f]) at 24 h postincubation of three CD samples (i_8_-CD, i_8_-CD_>3kD_, i_8_-CD_<3kD_). Similarly, i8-CD demonstrates CDs irradiated with 60 μmol photons/m2/s for 8 days and where i8-CD_>3kD_ and i_8_-CD_<3kD_ indicate the molecular size of the fraction tested from an original i_8_-CD sample. Trends depict the increase cytotoxicity with i_8_-CD_<3kD_ and i8-CD indicative of increased cytotoxicity with photolyzed carbon dot products which have a size of <3kD. Adapted with permission from ref (1309). Copyright 2021 Nature.

The investigations of cytotoxic concentrations of CDs can be misleading. This comes as a surprise since advantageous properties of CDs include their biocompatibility and low cellular toxicity. However, in a recent study conducted by Lui et al., it was demonstrated that the photodegradation of several CDs samples synthesized from different compounds can result in the generation of smaller CDs fragments upon exposure to light, which can notably increase their cytotoxicity *in vitro.*(1309) This degradation process was found to be driven by the production of alkyl and hydroxyl radicals when CDs were exposed to light. Specifically, CD samples were subjected to light irradiation in an aqueous solution at a rate of 60 μmol photons/m^2^/sec for varying durations (0.5, 1, 4, 8 days) and concentrations (0, 10, 30, 100, and 300 mg carbon/L) of CDs. The impact on the treatment of three cell lines (HeLa, Hep-G2, and HEK-293) from glucose pyrolysis-derived CDs were evaluated illustrated in Figure 49. Notably, prolonged light exposure of CDs led to a significant decrease in cell viability, and similar cytotoxic effects were observed when the cells were treated with photodegradation products of <3 kDa filtrate solution from CD treated with light and CDs that had been irradiated for 8 days. Moreover, to determine the composition of CD degradation byproducts, mass spectrometry was utilized, and it was elucidated that chemical structures observed in degradation products were glucose-PEG linkage, aldehydes, poly aromatic rings, pentose, and tetrose conjugated PEG. Similar degradation patterns were observed from nitrogen containing CDs, commercially available CDs, and silicon-containing CDs, suggesting any type of CDs has the propensity to undergo similar decomposition under light, and have potential to be cytotoxic. Given this recent and novel insight, light exposure should be taken into consideration when utilizing CDs for biological applications. This is especially true since the previously reported cytotoxicities of CDs were often contradictory ranging from very biocompatible to mildly biocompatible.^1310−1315^ It may be possible that sample handling may have been influencing these ranges of biocompatibilities in prior studies.

##### 6.1.1.4 Graphene Oxide (GO)

Studies have recently shown that GO size has a major implication on its cytotoxicity. Specifically, in a study conducted by Gurunathan et al., they found that GO smaller than 100 nm made the TM3 and TM4 cell lines susceptible to higher levels of leakage of lactate dehydrogenase (LDH), and caused generation of more ROS compared to GO samples with 100 nm diameter.^1316^ This suggests that the formation of ROS is more prevalent for smaller CNMs. However, it must be noted that size is likely not the only contributing factor to GO’s cytotoxicity. In fact, other key factors such as surface structure, functionalization, charge, aggregation, and impurities all play substantial roles in determining cytotoxicity. For more information on graphene family nanomaterial cytotoxicity, readers can refer to a review published by Ou et al.^1317^

#### 6.1.2 *In Vivo* Cytotoxicity

The *in vivo* assessment of cytotoxicity of CNMs involves a comprehensive investigation into their potential adverse effects on living organisms. Through carefully designed experiments, researchers administer various doses of CNMs to animal models, allowing for the observation of their impact over specific time periods. Tissues and organs are sampled and subjected to histopathological examination, revealing any morphological changes indicative of cellular damage, inflammation, or necrosis. For a comprehensive analysis of the *in vivo* CNM cytotoxicity, readers are encouraged to refer to recent reviews, as this is a heavily examined topic in previous reviews.^1318,1319^

There is a recent study performed to determine the cytotoxicity of CDs *in vivo*, where researchers found that CDs synthesized from sugar cane molasses at concentrations lower than 150 μg/mL had no impact on embryonic toxicity nor showed impediment on embryo development in zebrafish.^1320^ Conversely, concentrations higher than 200 μg/mL greatly increased cytotoxicity, and demonstrated an impact on the dopamine levels, and a decrease in TH^+^ neuronal cells. Additionally, upon oral administration of 5000 μg/mL CDs, they were localized to the brain, gills, heart, liver, and intestines with a gradual decrease over a course of 5 h. Model organisms, such as zebrafish and mice, have been instrumental in testing the cytotoxicity of CNMs, embryonic development, and localization of CNMs *in vivo*.

#### 6.1.3 *In Planta* Cytotoxicity

In the realm of plant research, assessing cytotoxicity takes on a unique perspective distinct from that applied to mammalian cells. The evaluation of the quantum yields of photosystem II, gene expression, and, in certain instances, cell viability assays stand as pivotal approaches to gauge the effects of CNMs on plants.

Monitoring the quantum yield of photosystem II is commonly used to determine physiological stress on plants induced by CNMs.^1294,1321,1322^ By selectively quantifying the maximal fluorescence (*F*_M_) and variable fluorescence (*F*_v_), both of which are quantum yields, a ratio of the two (*F*_v_/*F*_m_) can be used to compare quantum yields of plants that are infiltrated with CNMs to untreated plants. A decrease in quantum yield can be indicative of lower levels of photosynthesis, stress, and thus overall cytotoxicity.

Expression of reporter stress genes activated under stress conditions can also serve as an indicator of cytotoxicity in plant cells. Plant NADPH oxidases, also known as respiratory burst oxidase homologues (rboh), are enzymes that catalyze the formation of a superoxide anion and are often upregulated to combat biotic and abiotic stressors.^1323^ The quantification of the expression of these genes, as a stress and cytotoxicity proxy, can be performed via reverse transcriptase quantitative polymerase chain reaction (RT-qPCR) or at the protein level via Western blot.^27^ Moreover, plant response to CNMs can also be characterized at the whole plant transcriptome level using RNA-seq.^1324^

Similar to determining cellular viability in mammalian cells, plants can be subjected to dyes that provide information on cellular viability. A common dye used to determine cytotoxicity in plants is propidium iodide (PI).^1294^ This impermeable florescent probe, which is only internalized when cell membranes are compromised, can be visualized by confocal microscopy and can offer modes of detection for compromised cells. Additionally, other dyes like Evans blue have been utilized to determine cellular viability in plants.^1293^ Like PI, Evans blue can be used to determine compromised cells by binding to proteins and nucleic acids.

A recent study led by González-Grandío et al. delved into the investigation of the toxic effects of SWCNTs in plants using RNA-seq.^1325^ The SWCNTs that were modified with PEI, when used at high concentrations (50 mg/L), triggered upregulation of genes associated with programmed cell death in leaves of *A. thaliana.* However, at lower concentrations (1 mg/L), stress gene expression levels returned to baseline values 6 h after infiltration. Interestingly, carboxylated SWCNTs did not cause cytotoxicity even at the higher concentration of 50 mg/L. In *Nicotiana benthamiana*, higher molecular weight polymer–SWCNT conjugates were more toxic than the low molecular weight and linear polymer–SWCNTs.^1326^ An altered version of PEI with a low degree of hydrophobic modification was then used to functionalize SWCNTs, which showed significantly reduced stress response and less nonspecific protein adsorption in plant cells.

*In planta* cytotoxicities of other CNMs have also been evaluated. CDs synthesized from citric acid and urea and modified with β-cyclodextrin molecular baskets showed best biocompatibility at 20 mg/mL in *Arabidopsis* model systems.^1294^ In another study, CDs that are electrochemically synthesized from graphene rods showed little to no toxicity at 0.8 mg/mL and interestingly increased the growth rate of plants, specifically eudicots. The authors hypothesized that several factors might have caused this, including the decomposition of CDs to CO_2_ and plant like hormone analogs.^1327^ Although certain CD and SWCNT formulations appear to be noncytotoxic in plants, additional investigations into the long-term and prolonged exposure effects are needed.

### 6.2 Environmental Accumulation and Fate of CNMs

The expanding production and utilization of CNMs necessitates careful consideration and evaluation of their environmental impacts. Projections estimate that by 2030, 20–40 tons of CNTs will annually be released to soil, resulting in a concentration range of 0.01–3 μg per kg soil.^1328−1330^ Moreover, it is expected that within the next half-century, the concentration of CNTs in surface waters and sediments will reach 5 μg per L and 970 mg per kg, respectively.^1331,1332^ As such, this section discusses the accumulation and fate of CNMs in the environment.

CNMs have the potential to be released into the environment through a variety of pathways, including industrial processes, waste disposal, accidents, and consumer products.^1333^ The specific mechanisms responsible for their release involve biodegradation, mechanical means (e.g., abrasion, scratching, or sanding), washing, diffusion, matrix degradation (which can include photo-, thermo-, or hydrolytic degradation), and incineration.^1333^ The exact pathway and mechanism of release may vary depending on the context and properties of the materials.^1334−1336^ For instance, when CNMs are present in landfill settings, they are likely to enter the environment through hydrolytic degradation or photodegradation processes.^1333,1337^ Conversely, in aquatic settings, CNMs are typically released into the environment through rainwater and runoff from contaminated air and soil, deposition from aerial and tire sources, or emissions from wastewater treatment plants.^1338,1339^

Once introduced into the environment, the fate of CNMs varies depending on many factors, including but not limited to the particle exposure concentration, size, surface properties, and solubility/degradability. Consequently, the behavior and impacts of CNMs have been the focus of numerous investigations, with particular attention paid to their effects on either aquatic or soil environments. Table 8 presents a comprehensive summary of all studies since 2017 that investigated the distribution and ultimate destiny of diverse CNMs in the natural environment.

**Table 8:** Accumulation and Fate of Diverse CNMs in Environmental Settings

Materials	Testing Methods/Conditions	Major Outcomes	Ref
CDs	CD application to lettuce and tomato in a hydroponic nutrient solution	• CDs penetrate aerial parts of plants	(1361)
		• Enhanced seed germination, seedling growth, and overall plant development	
		• Improved absorption of mineral elements and enhanced photosynthesis	
Various kinds of SWCNTs	Factors influencing uptake of nanoparticles within plant chloroplasts	• SWCNTs can passively traverse the chloroplast membrane	(1364)
		• Inside the chloroplast, SWCNTs exhibit confined diffusion and convection	
		• Eventually, SWCNTs reach a state where they become irreversibly trapped	
SWCNTs	Investigated the interaction between SWCNTs and plant organelles to determine their potential for enabling novel or improved functions	• SWCNTs passively transport and permanently localize within the lipid envelope of isolated plant chloroplasts	(1053)
		• 3-fold increase in photosynthetic activity compared to control	
		• SWCNTs enhance maximum electron transport rates within the chloroplasts	
0.72% MWCNT/amine-cured epoxy nanocomposite	Accelerated aging using intense UV radiation at elevated temperature and humidity	• Photodegradation of the nanocomposite matrix by UV radiation	(1337)
		• Formation of a dense MWCNT network on the surface, no MWCNT release	
Oxidized multiwalled carbon nanotubes (O-MWCNTs)	Evaluated the developmental toxicity of O-MWCNTs using *A. salina* cysts and larvae at various developmental stages	• O-MWCNT distribution in *A. salina’s* phagocytes, lipid vesicles, and intestine	(1338)
		• After 72 h, most of the accumulated O-MWCNTs were excreted	
		• Gradual increase of O-MWCNT from 1 to 48 h, and rapid decrease from 48 to 72 h	
Pristine and carboxyl-functionalized MWCNTs	Hydroponically grown lettuce with varying concentrations of pristine or carboxyl-functionalized MWCNTs	• Localization in lettuce roots, stems, and leaves	(1328)
		• Carboxylation enhances the absorption and distribution of MWCNTs in lettuce	
MWCNTs, GO, rGO	Fate and transport of MWCNTs, GO and rGO in four aquatic ecosystems in the southeastern US	• 99% of nanomaterials undergoes transportation via systems with varying residence times, no heteroaggregation or sediment deposition	(1331)
		• Substantial sediment accumulation does occur over extended time	
MWCNTs	Fate of MWCNTs by considering the presence of coexisting metals and NOM	• Humic substances and changes in chemical structure contribute to toxicity	(1343)
		• Accelerated sedimentation and immobilization of Cd in a dose-dependent manner at the water-sediment interface	
MWCNTs	Absorption of MWCNTs in tomato fruits and accumulation in the organs of mice that consume these fruits	• Feeding mice CNT-contaminated tomatoes showed no toxicity	(1365)
		• Minimal CNT accumulation in mice’s organs, indicating that nanofertilization does not lead to toxicity	
O-MWCNTs	Impact of two different coexposure protocols on the interaction between O-MWCNTs and Cd in a zebrafish liver cell line	• Decreased activity of catalase, glutathione peroxidase, and glutathione S-transferase	(1332)
		• Alterations in the cell cycle, reducing the number of cells in the G2/M phase	
MWCNTs	Mechanisms behind the phytotoxicity of MWCNTs on *Arabidopsis*	• Hindered root and leaf growth, oxidative stress, impeded photosynthesis, and suppressed auxin signaling at high concentrations	(1362)
MWCNTs	Growth and reproduction toxicity of MWCNTs on sexually mature *Xenopus tropicalis*	• Hindered growth and development of *Xenopus tropicalis*	(1349)
		• Inhalation of MWCNTs led to their accumulation in lungs and development of lesions	
C_60_	C_60_ stability and transformation under UV exposure	• Pseudo-first order degradation profile	(1354)
		• 0.8 to 13.1 days half-life	
		• Transformation of fullerenes mediated by light is likely to occur in the environment	
C_60_	Effects of short- and long-term exposure of sediment-associated fullerenes on *Chironomus riparius*	• Oxidative stress reactions in tissues adjacent to microvilli and exoskeleton	(1353)
		• Swift uptake of fullerenes	
		• Decrease in body residues following chronic exposure to fullerenes	
C_60_	Synergistic impact of C_60_ and benzo(α)pyrene (B(α)P) on zebrafish embryos	• Interaction between C_60_ and B(α)P led to elevated accumulation of pollutants within the tissues, inducing genotoxicity and oxidative stress	(1366)
C_60_	Uptake, transportation, and bioaccumulation of C_60_ fullerenes and selected heavy metal ions (Cd, Pb, and Cu) in four rice cultivars	• C_60_ nanoparticles taken up by rice roots and distributed to stems and panicles, which is influenced by rice cultivar, soil heavy metal concentration, and C60 exposure duration/concentration	(1367)
GO	Bioaccumulation of GO in wheat seedlings	• 15 days of GO exposure led to significant accumulation (112 μg/g) in wheat roots	(1368)
		• Hindered plant development, growth, disrupted root structure, oxidative stress	
		• Minimal translocation of GO from roots to stems and leaves	
GO and amine-functionalized GO (G-NH2)	Phytotoxic effects of unfunctionalized GO and G-NH2 on wheat plants	• Significant morphological damage and reduced root and shoot length, and biomass compared to the control at high GO concentrations (>1000 μg/mL)	(1369)
		• G-NH2 exposure enhanced plant growth: ∼19.% increase in root and stem length at 2000 μg/mL G-NH2	
GO	Impact of GO on copper (Cu) stress in duckweed	• GO alleviates Cu stress by adsorbing Cu, protecting plants from harmful impacts of elevated Cu concentrations	(1336)
GO	Exposed young apple plants grown in tissue culture to varying concentrations of GO	• Applying 0.1 mg/L GO to apple plants had a favorable impact on root development, while adversely affecting root growth	(1370)
GO	Effects of GO on the freshwater cladoceran *Ceriodaphnia dubia*	• Immediate and long-term impacts affecting feeding and reproduction	(1351)
		• GO accumulation within the gut tract of the organisms	
GO	Toxic effects of GO on wheat seeds by subjecting wheat caryopses to various concentrations of GO	• Germination and root elongation were negatively affected by higher doses of GO	(1363)
		• Accumulation of aberrant cells, primarily near the plumules’ intercellular space	

**Figure 50:**
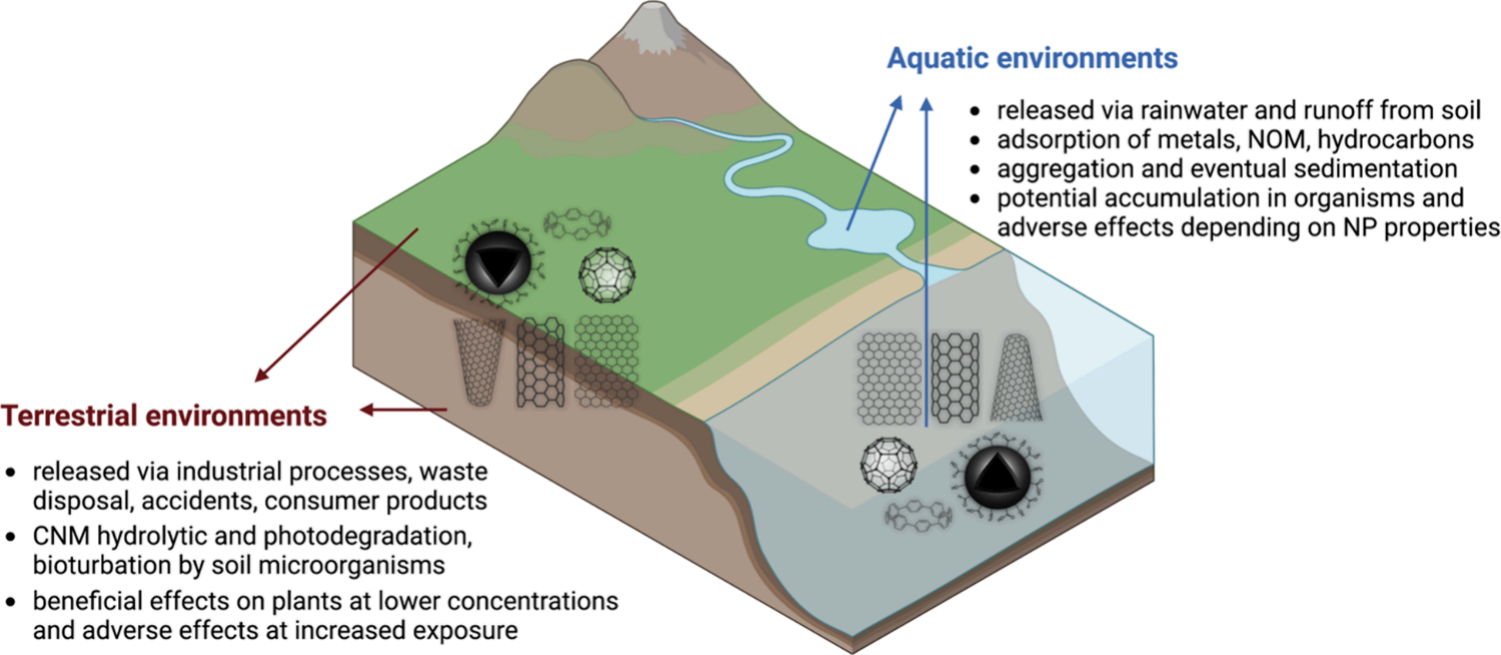
Accumulation and fate of CNMs in aquatic and terrestrial environments. Figure prepared using BioRender.com.

In aquatic environments, CNMs undergo a range of processes (Figure 50). In environments with high ionic strength or natural organic matter (NOM), the extensive surface area of certain CNMs allows for the adsorption of components such as metals, polycyclic aromatic hydrocarbons (PAHs), NOM, and other compounds onto their surfaces. These adsorbed CNMs may subsequently undergo homoaggregation with other CNMs or heteroaggregation with other particles, leading to their sedimentation.^1340−1342^ To better understand these interactions, Xu et al. conducted a study on the fate of MWCNTs considering coexisting metal Cd and NOM.^1343^ Their results indicated that upon discharge into the environment, MWCNTs promoted Cd sedimentation and immobilization. However, it is worth noting that in natural settings, sedimentation is not an instantaneous process; it can span over long time periods. Owing to their size, charge, the stabilizing agents used to deter aggregation, or due to environmental transformation and sediment resuspension, CNMs might linger in water sources.^1344^ Corroborating this, Avant et al. found that more than 99% of CNM mass traverses through natural water systems without undergoing heteroaggregation or being deposited into the sediments.^1331^ This implies that concentrations may accumulate downstream in river systems, all the way to reservoir or ocean end points, and can persist in the water column’s free phase for protracted periods. Eventually, CNMs will accumulate in sediments, and with prolonged exposure, the sediment concentrations may see a substantial increase. Predictive models estimate recovery periods to reduce sediment CNM concentrations by half to be over 37 years for lakes and 1 to 4 years for rivers.^1331^

In addition to their accumulation in the environment, certain CNMs can potentially elicit harmful effects on neighboring organisms through several mechanisms, including the induction of oxidative stress, inflammatory responses, DNA damage, and mutations. Moreover, CNMs can interfere with electron-mediated transfer processes between themselves and biological membranes.^1345−1349^ Notably, studies by Zhao et al. and Cano et al. have reported the toxicity and accumulation of MWCNTs in *Xenopus tropicalis*,^1349^*D. magna*, and *Fathead Minnow.*(1350) These studies have revealed that MWCNTs can accumulate within aquatic organisms and induce adverse effects. For instance, MWCNTs accumulated in the lungs of *X. tropicalis*, resulting in lesions and inhibited growth and development. Moreover, it has been established that both fullerene and GO can induce toxicity in aquatic organisms. Specifically, unfunctionalized GO has demonstrated a notable mean effective concentration of 1.25 mg per L when *C. dubia* is acutely exposed to it.^1351^ This exposure leads to the ingestion and subsequent accumulation of GO within the gut tract of these organisms. Consequently, the accumulation of GO prompts *C. dubia* to allocate energy toward maintaining defense mechanisms, such as antioxidant processes. As a result, this diversion of energy may potentially reduce the availability of energy for crucial physiological processes, including reproduction and foraging activities.^1352^ Another study examined the impacts of both acute (12 and 24 h) and chronic exposures (10, 15, 28 days) to sediment-bound fullerenes (at concentrations of 0.025, 0.18, and 0.48 mg/cm^2^) on the benthic invertebrate *Chironomus riparius.*(1353) The presence of C_60_ within the upper sediment layer leads to the swift assimilation of C_60_ aggregates in their gut, consequently inducing oxidative stress in tissues adjoining the microvilli and exoskeleton.

Similar to the aquatic environments, the destiny of CNMs in terrestrial environment is subject to various factors (Figure 50). To explore the impact of environmentally relevant conditions on fullerenes, Carboni and colleagues conducted an incubation experiment with C_60_ under ultraviolet A (UVA) irradiation for 28 days.^1354^ Their findings indicate that UVA irradiation alone can induce considerable degradation of C_60_. However, when C_60_ was introduced into quartz sand or sandy soil samples, degradation occurred at a significantly faster rate. This suggests that once CNMs are deposited in soil, their fate is likely determined by a complex interplay of interactions with soil materials and microbiota. These interactions involve the physical disturbance and mixing of soil through bioturbation, which encompasses the activities of living organisms that alter soil structure and nutrient distribution. Additionally, the fate of CNMs is influenced by biotransformation, which encompasses the chemical transformations occurring within organisms, leading to the conversion of CNMs and other substances into different forms. The scientific literature contains numerous investigations of the biodegradation of CNMs by soil bacteria.^1355−1358^ For example, Chouhan and colleagues identified a bacterium named *T. guamensis* that is capable of oxidizing and partially catalyzing the degradation of MWCNTs.^1355^ Furthermore, Berry and colleagues discovered that in organic-rich clay, the presence of agricultural soil bacteria significantly enhances the mineralization degradation rate of C_60_, with over 50% of C_60_ being mineralized in 65 days.^1359^

Certain CNMs could demonstrate beneficial effects by boosting various functions in plants, such as water uptake, water transport, seed germination, nitrogenase, photosystem, and antioxidant activities. Additionally, they stimulate water channel proteins and enhance nutrition absorption.^1051,1360,1361^ However, an excess of these CNMs may not be favorable. At higher concentrations, CNMs could accumulate within plants, thereby inhibiting growth.^1051^ For instance, when *Arabidopsis* is exposed to elevated levels of MWCNTs, root elongation is impaired in a dose-dependent manner. This overaccumulation can even extend to leaves, affecting their growth.^1362^ The adverse effects of CNMs on plants are generally attributed to their ability to trigger oxidative stress. Further research has identified an increase in the activity of antioxidant enzymes, including catalase, peroxidase, and superoxide dismutase, when GO is introduced. This heightened enzymatic activity points to the oxidative stress that arises from GO treatment.^1336,1363^

In summary, the escalating production and use of CNMs in multiple domains pose a growing risk to the environment and ecological balance. Upon release, these nanomaterials undergo complex interactions within aquatic and terrestrial ecosystems, leading to potential accumulation and consequent adverse impacts on local flora and fauna. Understanding the fate and influence of these materials is paramount to establishing effective risk assessment and mitigation strategies. It is crucial to continue extensive research in this area with a particular emphasis on developing environmentally benign alternatives, containment strategies, and waste management approaches. The findings reviewed herein underscore the pressing need for stringent guidelines to manage the environmental impact of CNMs and safeguard ecosystems (see Section 6.3.3).

### 6.3 Scale-Up, Economical, and Regulatory Considerations of CNMs

Given the considerable potential exhibited by CNMs discussed in prior sections, it becomes imperative to explore the feasibility and ramifications of their mass production. To expand their applications and fully realize their capabilities, three pivotal factors necessitate thorough examination: scaling up production, assessing economic feasibility, and considering regulatory constraints.

#### 6.3.1 Scalability of CNM Synthesis

The scalability of CNMs revolves around two essential aspects: production consistency and process efficiency. Ensuring uniformity in synthesized probes is crucial for obtaining reliable and reproducible outcomes. However, achieving this consistency across large batches poses a challenge^1371^ due to structural inhomogeneity and imprecise fabrication arising from the synthesis process.^1372^

Presently, the three most commonly employed methods for CNM production include arc-discharge, laser ablation, and CVD. While the arc-discharge method excels in generating high-quality CNMs, it is resource-intensive and requires expensive equipment, rendering it less suitable for large-scale production.^1373−1375^ In contrast, laser ablation operates at relatively lower temperatures compared to arc-discharge and employs solid carbon sources.^1376−1380^ However, it still requires high energy consumption and has the potential for product entanglement, as well as inadequate product purification, thereby presenting obstacles to scalable production of high-quality materials.^1381^ Therefore, CVD has emerged as a prominent choice for mass production.^1382,1383^ CVD involves the vaporization of carbon-containing precursors onto a substrate, where CNMs decompose and form. This method has garnered substantial attention due to its capacity to achieve relatively low-cost production, high yield, and less stringent reaction conditions.^1382,1384^ However, an inherent challenge with all these methods is the batch-to-batch variation, both in terms of nanoparticle properties (size, charge etc.) and impurity levels. These variations can impact the utility of the synthesized CNMs for specific applications and can create difficulties in achieving standardization.^1385^

Despite this, extensive research dedicated to employing CVD in the large-scale synthesis of diverse CNMs has seen significant advances.^1382^ These advancements encompass the successful large-scale production of notable materials such as SWCNTs,^1386,1387^ MWCNTs,^1388^ fullerene,^1389^ graphene,^1390^ along with other noteworthy CNMs.^1382^ Moreover, the harnessing of CVD for the mass manufacture of CNMs has stimulated the creation of various innovative techniques. Key developments in this realm include techniques like hot-filament CVD,^1391^ ultra-high vacuum plasma-enhanced CVD,^1382^ and floating catalyst CVD.^1392^ These techniques have proven pivotal in enhancing both the efficiency and quality of mass production. floating catalyst CVD, for instance, employs gaseous or liquid precursors to facilitate high productivity, superior quality, and controlled growth. It also ensures excellent uniformity while easing the controlled doping of CNMs with heteroatoms.^1393,1394^ This represents just one example of how these advancements in CVD technology are transforming the production of CNMs.

#### 6.3.2 Economic Feasibility of CNM Technologies

In addition to direct production costs, broader economic considerations encompass market potential, competition, and cost-effectiveness. As the potential applications for CNMs continue to expand, so does their market potential. However, to effectively compete with established fluorescence technologies like organic dyes, it is crucial to lower production costs and demonstrate superior long-term cost-effectiveness. The selection of precursors plays a vital role in managing production costs.

Traditional precursors such as coal, mesophase carbon, benzene, *n*-hexane, methane, ethylene, and acetylene are predominantly derived from petroleum sources. This reliance on petroleum presents a range of challenges, including environmental concerns, depletion of fossil fuel resources, and uncertainty in crude oil prices.^1395−1397^ To address these issues, the exploration of alternative precursors, such as biomass-derived materials, emerges as a more sustainable and cost-effective option. Biomass-derived materials offer a diverse range of alternatives, including algae, crop stalks, husks, cellulose, starch, chitosan, and lignin, among others.^1398−1400^ These renewable sources present the potential for sustainable CNM production. Notably, some of these materials have already been demonstrated to be suitable for manufacturing CNMs with high quality and sensitivity, as showcased in previous sections.^1401^ Although biomass-derived materials show promise as alternative precursors for CNM production, they present specific challenges related to their high oxygen content and the need to maintain consistency. In CNM synthesis, low oxygen content in the starting material is generally preferred,^1381^ which is commonly found in fossil-based hydrocarbon precursors like methane and benzene. The relatively high oxygen content of biomass, particularly in carbohydrates, may complicate its suitability as a precursor.^1381,1402^

#### 6.3.3 Regulation of CNM Usage

In the context of CNMs’ regulatory considerations, it is essential to understand that regulatory agencies worldwide are paying close attention to CNMs. In an era where nanotechnology is making strides, ensuring safety and compliance with environmental, health, and safety (EHS) regulations is fundamental.^1403^ Nanomaterials, presently governed by conventional chemical regulations, fall under the jurisdiction of various international entities. For instance, the United States’ Environmental Protection Agency (EPA) enforces the Toxic Substances Control Act (TSCA) to supervise nanomaterials.^1404^ This regulation necessitates a premanufacturing notification for any new chemical substances, including nanomaterials, before they can be produced or imported. Meanwhile, in Europe, the European Chemicals Agency (ECHA) regulates nanomaterials through the REACH (Registration, Evaluation, Authorization, and Restriction of Chemicals) and CLP (Classification, Labeling and Packaging) regulations.^1405^

Despite these regulations, concerns persist that conventional chemical laws may not adequately consider the distinctive properties and behaviors of nanomaterials. For example, the International Chemical Secretariat (ChemSec) has classified CNTs on the SIN (Substitute It Now) list, which is a comprehensive catalog of chemicals that, according to ChemSec, should be restricted or prohibited within the EU.^1406^ However, categorizing all CNTs under a single material type is simplistic, given their diverse structures, physical dimensions, modifications, and wide-ranging applications.^1407^ This diversity can lead to significantly varying levels of toxicity and environmental impact.

Under existing procedures, safety data sheets offer details about potential hazards, as well as guidelines for safe handling, storage, and emergency response for specific substances or products.^1408^ Nonetheless, safety data sheets for nanomaterials present a key issue: they often outline different toxicity profiles than those of their bulk material equivalents.^1403^ It is reported that approximately 35% of these nanomaterial-focused safety data sheets could be unreliable.^1409,1410^ Addressing this gap requires a focused effort in streamlining research in the field of CNMs and their environmental effects. To start, establishing standardized practices and protocols is crucial. Central to this is the creation of a universal set of guidelines for characterizing CNM properties, such as size, shape, surface area, stiffness, and functional groups, as these factors are critical in determining their fate and toxicity in environmental and health contexts. For instance, studies show that longer CNTs, exceeding 15–20 μm, may induce “frustrated” phagocytosis, where immune cells cannot effectively absorb and break down these CNTs, leading to potential harm.^1411^ Conversely, shorter CNTs seem to be less hazardous. Moreover, the stiffness of CNMs significantly influences their interactions with biological systems. Rigid CNMs can damage lysosomes, vital cell organelles, and such stiffness is linked to both acute and chronic inflammatory responses.^1412^ Therefore, having a uniform characterization protocol is essential for achieving global consistency in CNM research. This uniformity facilitates easier comparison and analysis of results from different studies, enhancing the overall understanding of CNMs and their effects on the environment and health.

In parallel, standardizing experimental conditions and protocols is equally important. This involves accounting for variables such as organism, tissue, or cell type, and their respective ages and health statuses. Given that CNMs’ impacts can vary across biological systems and conditions, standardizing these factors is essential. This includes documenting and standardizing application conditions like concentration, exposure method, and duration. Establishing common guidelines for these variables will help reduce variability and conflicting findings in current research. Finally, emphasizing comprehensive reporting and transparency in research findings is critical. Encouraging or mandating researchers to report all relevant information, including both positive and negative results, under specific conditions, can enhance the reliability of data. Peer-reviewed journals can enforce these standards by requiring detailed methodological information for publication. Promoting open data and science practices can further accelerate discovery and understanding. In summary, while CNMs and their fluorescent probe derivatives exhibit enormous potential, their journey from the lab to the market is subject to a host of regulatory considerations. As the regulatory landscape for nanomaterials continues to evolve, it is essential to maintain active engagement with regulatory agencies to ensure the responsible and sustainable development and commercialization of CNMs. Future research should aim not only to improve the production efficiency and cost-effectiveness of these materials but also to better understand and mitigate their potential environmental and health impacts.

## 7 Perspectives and Outlook

As we highlight in this review, CNMs have enabled important advancements in the fields of physiological imaging, biosensing, cargo delivery, and therapeutics. Although their uses were initially demonstrated *in vitro* and *in vivo* in animals and animal derived cells and tissues, CNM usage has been successfully translated to microbes, plants, and various other organisms for a plethora of applications, which we explored extensively in this review.

In the biomedical imaging field, fluorescent CNMs have found fruitful niche applications that are enabled by several unique attributes. First, their photoluminescence properties exhibit a high degree of tunability, and between the family of CNMs reviewed in this paper, it is possible to find probes that emit stably across a wide swath of the electromagnetic spectrum, including UV, visible, NIR, and SWIR. Use cases in NIR and SWIR are particularly tantalizing given the advantages associated with imaging in the so-called “tissue-transparency” window, which we discussed broadly in this review. The synthetic nature of CNMs affords flexible deployment to enable applications where genetically encoded probes may be unavailable or are not feasible, as we highlight in several examples where these materials are used as implantable probes, composite films and as bionanohybrids in conjunction with other biological matter. Another common theme in the application of CNMs relates to the dual role that most CNMs can play, in which imaging and environmental applications are often seamlessly coupled to, and sometimes facilitate, their use as therapeutics agents or as cargo delivery vehicles. This feature appears to be common among most CNMs reviewed in this paper and could be an important competitive edge for these materials. Compared to small molecule organic probes, synthesis of CNM probes is cheaper and less reliant on sophisticated synthesis and purification techniques. In this sense, the relative accessibility of these materials could contribute to their appeal to a broad segment of the scientific enterprise.

However, despite these advantageous attributes, there are several areas where the field needs to focus for improvement. First, the relative ease in the synthesis of CNMs can make keeping track of advancements in this field challenging, and the literature in this field is considerably vast and growing. Adding to the challenge, most studies appear to rely on discipline specific, and sometimes even lab-specific practices for purification and characterization of these materials, which makes comparative analysis across studies difficult. The lack of a consistent approach also makes replicating specific claims challenging and contributes to inconsistencies. For example, the literature on cytotoxic effects of CNMs has led to findings that appear contradictory, with claims ranging from complete biocompatibility to acute cytotoxicity, as we highlight in our review. This can make interpretation of results in biological settings difficult, and in some cases can lead to ill-advised policy decisions on regulatory frameworks that govern the eventual deployment of CNM-based reagents in biomedical settings. Even some fundamental properties, such as the precise mechanism of fluorescence in CDs, or the set of biointerfacial properties that govern CNM cellular entry, remains surprisingly enigmatic. Therefore, the field needs to focus on harmonizing, and if necessary, inventing techniques for synthesis, characterization, functionalization, and deployment of these materials.

Ultimately, these gaps in our knowledge contribute to some critical challenges for CNM based technology. In the area of biosensors, for example, a facile and modular approach for developing a sensor for a given analyte of interest does not exist. The optoelectronic properties of SWCNTs offer a good example. The mechanism of SWCNT photoluminescence is well understood and emanates from quantum confined surface excitons that are sensitive to the immediate chemical environment that the nanoparticle is exposed to. The material is relatively better characterized, has a large surface area, and has well described chemistries to functionalize the surface. Despite these attributes, no unified principle exists that govern the conjugation of SWCNTs to molecular recognition motifs for biosensor synthesis. Some of the better characterized sensors, such as those for catecholamines, still lack mechanistic descriptions of how analyte binding leads to massive modulations in fluorescence intensity. Working out the molecular mechanisms that underpin these ssDNA@SWCNT bionano conjugates could open a beachhead in turning these CNMs into a platform technology that can be modularly tuned as a sensor for a broader class of analytes. Currently, there are CNM sensors for many classes of metabolites, hormones, proteins, lipids, metals, ROS/NOS, pathogens, among many other molecules, both for biomedical and environmental applications, which we reviewed extensively in this paper. However, the process of biosensor development for most of these targets relies on serendipitous discoveries or low-throughput screens, and typically does not involve rational use of molecular recognition motifs, necessitating time- and resource-intensive screens. Some recent progress has been made in this field with higher-throughput enrichment-based screens and AI/ML-based selection strategies. These types of explorations should be highly encouraged. Future studies focusing on this issue will greatly benefit the CNM biosensing field.

Improvements in CNM imaging modalities and hardware constitute another opportunity for the field. In the plant imaging field for example, CNMs have been used to track intracellular cargo delivery and enabled a rapid way of testing nanoparticle-cargo formulations that are effective in such systems. However, the strong autofluorescence of plant tissues, especially in leaves due to the chlorophyll, can make imaging of CNM fluorescence difficult. In addition to chlorophyll fluorescence, plant tissues demonstrate a high level of background fluorescence when damaged, which makes it challenging to detect CNM uptake. New imaging modalities, such as those based on FRET or fluorescence complementation, have not been sufficiently explored for CNM imaging and are therefore an area of opportunity. Moreover, imaging CNMs that fluoresce in the NIR and SWIR regions of the spectrum have often relied on custom-made microscopy solutions that have wildly varying performance. This is largely a consequence of the fact that most fluorescent reagents used in experimental biology fluoresce in the visible region of the spectrum. The dearth of reagents that fluoresce in NIR and SWIR, including proteins and synthetic small molecule dyes, has led to a concurrent lag in the performance of hardware in NIR/SWIR. Microscopes, cameras, PMTs and related instrumentation for NIR/SWIR do poorly relative to equivalent technologies in the visible range of the spectrum. Therefore, the field should make a concerted effort to incentivize manufactures to make improvements in hardware, and work to standardize practices through open-source collaboratives, as has been done in various fields of the scientific enterprise that have faced similar challenges.

The eventual translation of these CNM technologies to the clinic or environment will depend on many factors, including but not limited to the CNM cytotoxicity, environmental accumulation and fate, and their scale-up, economical, and regulatory considerations. There have been many studies of CNM cytotoxicity. However, these studies tend to take a generalist approach and attempt to extend result from one nanoparticle formulation to another, which is likely to be inaccurate as different size and surface properties of CNMs will affect their final cytotoxicity levels. The technical and economic feasibility of scale-up is yet another critical factor in translation. Future studies should focus on developing sustainable and affordable synthesis approaches that will result in highly uniform, robust, and replicable batches of CNMs. Last but not least, the regulation of CNMs both in medicine and environment needs to focus on each exact CNM formulation for safety and efficacy, and carefully weigh the benefit-risk ratio for a given application. Standardized approaches are needed not only for CNM testing, but also for enabling regulations that are universally agreed between different stakeholders and countries.
